# Mapping the Binding Energy of Layered Crystals to Macroscopic Observables

**DOI:** 10.1002/advs.202204001

**Published:** 2022-10-17

**Authors:** Mohsen Moazzami Gudarzi, Seyed Hamed Aboutalebi

**Affiliations:** ^1^ National Graphene Institute University of Manchester Manchester M13 9PL UK; ^2^ Department of Materials School of Natural Sciences The University of Manchester Manchester M13 9PL UK; ^3^ Condensed Matter National Laboratory Institute for Research in Fundamental Sciences Tehran 19395‐5531 Iran

**Keywords:** 2D materials, dielectric function, graphene, van der Waals interactions

## Abstract

Van der Waals (vdW) integration of two dimensional (2D) crystals into functional heterostructures emerges as a powerful tool to design new materials with fine‐tuned physical properties at an unprecedented precision. The intermolecular forces governing the assembly of vdW heterostructures are investigated by first‐principles models, yet translating the outcome of these models to macroscopic observables in layered crystals is missing. Establishing this connection is, therefore, crucial for ultimately designing advanced materials of choice‐tailoring the composition to functional device properties. Herein, components from both vdW and non‐vdW forces are integrated to build a comprehensive framework that can quantitatively describe the dynamics of these forces in action. Specifically, it is shown that the optical band gap of layered crystals possesses a peculiar ionic character that works as a quantitative indicator of non‐vdW forces. Using these two components, it is then described why only a narrow range of exfoliation energies for this class of materials is observed. These findings unlock the microscopic origin of universal binding energy in layered crystals and provide a general protocol to identify and synthesize new crystals to regulate vdW coupling in the next generation of heterostructures.

## Graphical abstract

A framework to probe and quantify binding energies of layered crystals is introduced and this binding energy is mapped to macroscopic observables by analyzing their optical properties with the goal of constructing reliable dielectric functions. Then, a unifying methodology bridging the gap between fundamental characteristics of materials and their binding energies for the entire class of vdW solids, is presented.

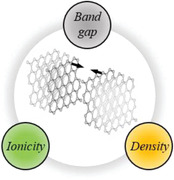

## 1 Introduction

The successful commercialization and integration of two dimensional (2D) materials into real‐world applications and devices requires advancing our understanding of the governing factors controlling the rational design, engineer, and manufacture of hierarchical hybrid van der Waals (vdW) 2D materials‐based architectures at both nano‐ and macro‐scale.^[^ ^1^ ^]^ Ubiquitous and long‐ranged intermolecular forces arising from quantum charge fluctuations, collectively known as Casimir–vdW forces,^[^ ^2^ ^]^ regulate many seemingly diverse fundamental phenomena from wetting and adhesion,^[^ ^3^ ^]^ to cell membranes’ conformation and functionality.^[^ ^4^ ^]^ Similarly, vdW forces are the key ingredient in modulating the fabrication and engineering of emergent novel hybrid vdW heterostructures with ensuing previously unexplored functionalities originating from vdW coupling,^[^ ^5^ ^]^ from simple energy harvesting and storage,^[^ ^6^ ^]^ to more sophisticated bio‐applications,^[^ ^7^ ^]^ and spintronics, opto‐valleytronics,^[^ ^8^ ^]^ and prototype quantum devices.^[^ ^9^ ^]^ However, the realization of such technologies, in practice, first requires the understanding of the nature of the binding energy in parent layered crystals which is unequivocally thought as the main factor determining the exfoliation of layered crystals into 2D materials and their self‐assembly into vdW heterostructures thereafter.^[^ ^1^, ^5^ ^]^ Nevertheless, precise measurement and theoretical description of the binding energy have proved to be nontrivial due to the ever‐expanding nature of 2D materials family members which puts the use of experimental methods for each 2D material, as the first step, out of the question.^[^ ^10^ ^]^ Nevertheless, the experimental data on binding energy of well‐studied crystals such as graphite over the past decades are not converging to a unique value.^[^ ^11^ ^]^ This holds true for other layered crystals such as MoS_2_, leaving the precise and reliable measurement of binding energy a challenging task.^[^ ^11^, ^12^ ^]^

To this end, many computationally prohibitively expensive methods such as the adiabatic connection fluctuation dissipation theorem (ACFDT) approach have been successfully applied to determine the binding energy of relatively simple crystals, from mono‐atomic to bi‐atomic layered crystals.^[^ ^13^ ^]^ The outcome of these computational methods points to the existence of a universal range of vdW bonding strength within a narrow range of 15–25 meV∙Å^−2,^ regardless of their electronic nature.^[^ ^13a^ ^]^ This is puzzling; however, as the magnitude of the vdW interactions among two bodies is closely related to their response to a perturbation of the electric field, that is, dielectric functions.^[^ ^14^ ^]^ These results led to the prediction of surprisingly similar binding energies for metals, semiconductors, or insulators,^[^ ^10^, ^13^ ^]^ in contradiction with classical picture for Casimir–vdW theories, where strongest attraction is expected for (ideal) metals.^[^ ^15^ ^]^

The main challenge in computation of vdW forces comes from the fact that the input dielectric functions, needed for the precise treatment of vdW interactions, in contact region, are not exact.^[^ ^14a^ ^]^ In the realm of colloids and surface science, these shortcomings have been circumvented by a set of empirical approximations where adhesion and cohesion energies of interfaces in contact region are estimated by additive long‐ranged vdW attractions (quantified by Hamaker constant) and by introducing a universal molecular length scale.^[^ ^16^ ^]^ These approximations have formed the current mainstream understanding of adhesion forces between solids and liquids over the course of the past 60 years, despite their limitations.^[^ ^17^ ^]^

On the other hand, accurate implementation of vdW forces in calculation of intermolecular forces has been the subject of an intense line of research specially to include these forces in density functional theory (DFT).^[^ ^14^ ^]^ An intermediate goal of such theoretical studies is the computation of the energy profile from which the binding (or cohesion) energies are obtained.^[^ ^13a^ ^]^ As such, finding the precise vdW energy profile at atomic distances, especially between solid interfaces, has been subject of debate in many theoretical studies and a challenge to overcome.^[^ ^14a^ ^]^ Here, we do not aim to obtain such energy profiles, but instead we show that the long‐range tail of the vdW energy profile quantified by the Hamaker constant is strongly correlated to binding energies obtained from benchmark calculations. Furthermore, we demonstrate, using fundamental limitations caused by causality and f‐sum rule, that the Hamaker constant is in turn controlled by two characteristic frequencies of crystals, namely effective plasma frequency and optical band gap. These findings combined with the renowned correlation of optical band gap and ionicity of crystals provide an explanation why the binding energy of layered crystals is confined to a limited range.^[^ ^18^ ^]^

To achieve this goal, we analyzed the optical properties measured for over 100 layered crystals with the goal of constructing reliable dielectric functions. These functions were then transformed to vdW energy profiles using macroscopic Lifshitz theory.^[^ ^15^, ^19^ ^]^ We then applied an empirical correction to compensate for breakdown of macroscopic vdW theory at atomic distances. Using our analysis, we can link the magnitude of the binding energy of the layered crystals to measurable macroscopic properties of this class of crystals, making it possible to distinguish or even possibly synthesize easily exfoliable crystals. Equipped with this knowledge, we propose that easily exfoliable crystals should be mainly made of light elements, possess low polarity, and have low valence electron density.

## 2 Results and Discussion

**Figure 1:**
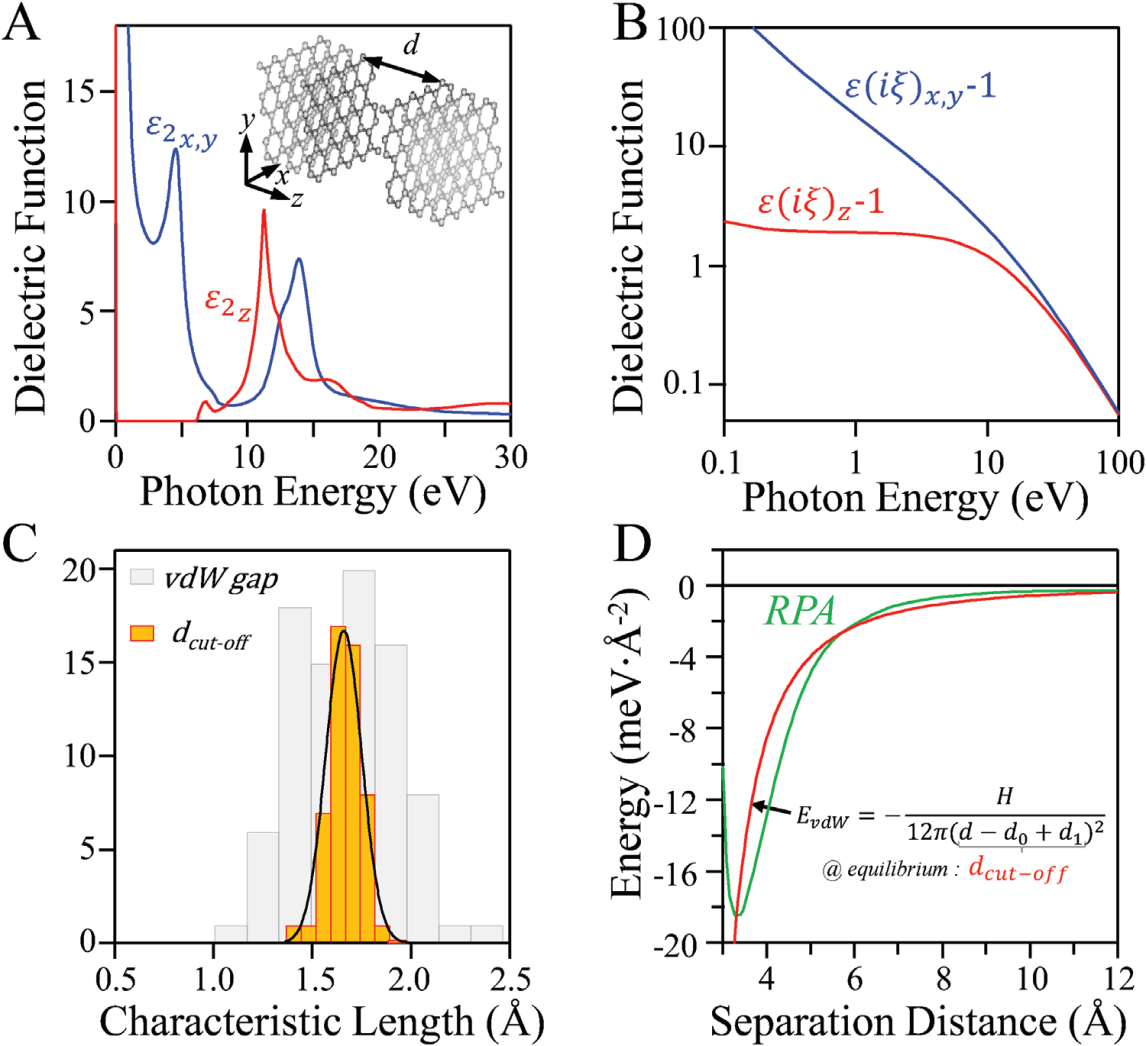
Computation of van der Waals attraction in various materials. Panel A) shows the imaginary part of the dielectric function of graphite along the crystallographic axes shown in inset by the respective vectors.^[^ ^19a^ ^]^ Panel B) is the Kramers–Kronig transformation of data in (A) to imaginary frequencies which was then transformed to vdW energy profile using Equations (1) and (2).^[^ ^16^, ^19^ ^]^ In Panel C) histograms of vdW gap for 86 elements and cut‐off distance computed for 52 different materials are shown. Details are given in Sections S2 and S15 of Supporting Information. Panel D) illustrates the empirical approximation to compute the binding energy. The green line shows the energy profile computed by the adiabatic connection fluctuation dissipation theorem‐within random phase approach (ACFDT‐RPA).^[^ ^13b^ ^]^ The energy profile predicted by Equation (1) should be corrected by two distance shifts. The empirical approximation is that at equilibrium, the difference of the equilibrium distance and the distance shift is a universal value, shown in (C). See Section S1, Supporting Information for more details.

The vdW energy potential between two slabs of aligned anisotropic crystals at distances, *d*, much larger than the interlayer spacing, follows the Lifshitz theory by the given equations (Equation (1) and (2)):^[^ ^19b^ ^]^

(1) $$\begin{array}{c} \begin{array}{c} E_{\mathrm{vdW}}=-\frac{H}{12\pi d^{2}}=-\frac{\mathrm{kT}}{16\pi^{2}d^{2}}\sum_{n=0}^{'\infty }\sum_{j=1}^{\infty }\frac{1}{j^{3}}\int_{0}^{2\pi }{\left(\frac{\varepsilon_{z}\left(i\xi_{n}\right)g\left(\psi \right)-1}{\varepsilon_{z}\left(i\xi_{n}\right)g\left(\psi \right)+1}\right)}^{2j}d\psi \end{array} \end{array}$$

(2) $$g^{2}\left(\psi \right)=\frac{\varepsilon_{x}\left(i\xi_{n}\right)}{\varepsilon_{z}\left(i\xi_{n}\right)}+\frac{\left(\varepsilon_{y}\left(i\xi_{n}\right)-\varepsilon_{x}\left(i\xi_{n}\right)\right)}{\varepsilon_{z}\left(i\xi_{n}\right)}\sin^{2}\left(\psi \right)$$

where *H* is the Hamaker constant, *kT* is the thermal energy, and *ε*_*i*_ are the components of the dielectric function tensor along different axes (**Figure** **1**A,B) in imaginary frequencies evaluated at bosonic Matsubara's frequencies, $\xi_{n}=\frac{2\pi nkT}{\hbar }$.^[^ ^19b^ ^]^ The prime in the first summation indicates the *n* = 0 term should be halved.^[^ ^19b^ ^]^ Provided that the input dielectric functions are exact, the Lifshitz theory prediction of both the magnitude and the power law falls in very precise agreement with experimental values down to separation distances of only a nanometre.^[^ ^19^, ^20^ ^]^ However, close to the contact region (when separation distance approaches the interatomic spacing), neither the magnitude nor the power law (*d* ^−2^) given by Equation (1) are valid (see Figure 1D; Section S1, Supporting Information).^[^ ^14a^ ^]^ As the magnitude of the Hamaker constant can reflect on the strength of the vdW forces at large distances,^[^ ^19a^ ^]^ we hypothesise that the Hamaker constant might be able to show a very strong correlation to the binding energy values. To test this hypothesis, first, we extend the empirical relation between Hamaker constant and vdW (or dispersive) surface energy to layered crystals. Fowkes,^[^ ^21^ ^]^ and later Israelachvili,^[^ ^22^ ^]^ in the search for quantifying the adhesion energy of liquid–solid interfaces, found that the ratio of Hamaker constant to dispersive (or vdW) binding energy, *E*_vdW_ (twice the surface energy) is nearly a universal value (≈1.58 ± 0.08 Å) for many liquids and solids.^[^ ^17b^ ^]^ An empirical cut‐off distance, *d*_cut − off_, can be defined as (Equation (3)):^[^ ^16^ ^]^

(3) $$d_{\mathrm{cut}-\mathrm{off}}=\sqrt{\frac{H}{12\pi E_{\mathrm{vdW}}}}$$

Our detailed analysis of experimental surface energy and optical properties of 52 various solids and liquids indeed confirms the universality in *d*_cut − off_, which is mostly ≈1.66 ± 0.09 Å (Figure 1C; Section S15, Supporting Information).

However, this implies that the vdW energy profile predicted by Lifshitz theory is extrapolated to a very short distances (a distance with no immediate physical meaning as it is much smaller than d‐spacing) and repulsion is modeled as a rigid step function. This notion is certainly not physical. Yet, the strikingly persistent observation of the universal *d*_cut − off_ in various materials ranging from rare gas solids to metal oxides and even liquid metals led us to propose a possible mechanism for this empirical observation (See Section S1, Supporting Information). To compensate for higher order dispersion terms,^[^ ^14a^ ^]^ we define a new term which we call the distance shift, *d*_0_. Therefore, we can rewrite Equation (1) in the following form proposed by Equation (4) (Figure 1D):

(4) $$E_{\mathrm{vdW}}=-\frac{H}{12\pi {\left(d-d_{0}+d_{1}\right)}^{2}}$$

Defining *d*_0_ term enables the proposed equation to follow the vdW energy profile. *d*_1_ also accounts for the repulsive term, which then allows to approximate the binding energy at equilibrium distance (Figure 1D). Therefore, in our opinion, *d*_cut − off_ is an effective distance controlled by vdW thickness and Pauli repulsion and as such is very close to the so‐called vdW gap distance of atoms, the difference of covalent and vdW radius (Figure 1C). Equation (4), then, allows for establishing the connection between the widely used approximation of vdW adhesion energy of interfaces to the first‐principles models for vdW interactions.

However, for the proper determination of *E*_vdW_, Hamaker constant, *H*, should be accurately computed. Intending to do that requires addressing one major obstacle, mainly the lack of full spectrum of dielectric function along all optical axes which is necessary for the computation of H (Figure 1A). Inaccuracy in the approximation of dielectric function can lead to an order of magnitude different values for H of layered crystals.^[^ ^23^ ^]^ To avoid this, we developed a model based on self‐consistent dielectric functions of crystals using Kramers–Kronig relations to accurately calculate H.^[^ ^19a^ ^]^ Knowing the chemical formula, density, optical band gap (*E*_g_), and electronic dielectric constant (*ε*_∞_), one can compute the dielectric functions over the entire spectrum using our modified harmonic oscillator model.^[^ ^19a^ ^]^ These data were collected for 107 crystals measured over the past century. Details are given for each crystal in Supporting Information and in ref. [19a]. Our analysis affirms that the error of this approach is better than 8% (Figure S6, Supporting Information).

Constructing the whole dielectric tensor, we can now back‐calculate *E*_vdW_ of the layered crystals through Equation (1) using an empirical cut‐off distance of 1.66 Å discussed above (Figure 1C). For simple layered crystals (bi‐atomic crystals) with low polarity such as *MX*_2_ (*M* = Mo, W and *X* = S, Se, Te), the binding energies are found to be ≈19.5 ± 1.3 meV∙Å^−2^, with a maximum difference of 9% compared to the output of benchmark ACFDT‐RPA calculations.^[^ ^13a^ ^]^ For metal iodides such as PbI_2_ or SbI_3_, we found *E*_vdW_ to be 11.3 and 10.1 meV∙Å^−2^, respectively, which are comparable to the outcome of rescaled DFT calculations, 10.4 and 11.8 meV∙Å^−2^, respectively (See Experimental Section for details of benchmarking).^[^ ^10^ ^]^ In contrast, for crystals with large ionicity such as hexagonal boron nitride (hBN), HfS_2_ and ZrS_2_, the computed *E*_vdW_ from Lifshitz theory is noticeably smaller than the benchmark calculations (for instance, the computed *E*_vdW_ of hBN based on Lifshitz theory is 31% smaller compared to the values reported from ACFDT‐RPA calculations). Note that the Lifshitz theory only considers interactions induced by charge fluctuations (*E*_vdW_), while ACFDT‐RPA accounts for the total energy (*E*_total_). This difference serves as evidence for the contribution of non‐vdW interactions (mainly electrostatic interactions) in polar crystals. The contribution of non‐vdW interactions to the total binding energy depends on the ionicity and charge distribution throughout the crystals. Therefore, we postulate that the ratio of the *E*_vdW_ to the total binding energy, *E*_total_, should inversely correlate with the ionicity of the layered crystals. Although different approaches are available to quantify the ionicity of crystalline solids (See Section S4, Supporting Information), we found that Pauling's ionicity (*f*) scale provides a reasonably accurate polarity scale for the layered crystals.

**Figure 2:**
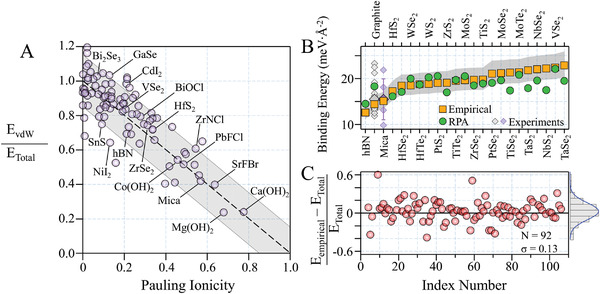
Impact of non‐vdW interactions on the binding energy of layered crystals. Panel A) shows the ratio of the binding energies computed from the Lifshitz theory to those obtained from the first‐principles calculations,^[^ ^10^, ^13^ ^]^ versus the Pauling ionicity of 92 layered crystals. See Section S16, Supporting Information, for details. The dashed line shows the prediction by Equation (5) and the grey region illustrates an error region of ±15% where 66 of the materials lie within. Spearman correlation factor is −0.74.^[^ ^26^ ^]^ Panel B) compares the binding energy for 23 crystals (mostly TMDs) obtained from the empirical relations in this work (orange squares) with the benchmark calculations using the ACFDT‐RPA (green circles). For both graphite and mica, the experimental binding energies are shown as well (Table S2, Supporting Information).^[^ ^13a^ ^]^ The grey area specifies the error which originates from the uncertainty in the cut‐off distance (≈ ±11%). The mean relative error and the mean absolute relative error are 4.8% and 9.2%, respectively. Note that for graphite and mica, we used the average value of experimental data for error calculations. Panel C) compares the relative error in empirical binding energies compared to first principles calculations for a wider range of crystals along with the distribution of the error. The average error is 3.0% and standard deviation is 13%. The index number refers to the number list of the crystals (listed in the Supporting Information).

**Figure** **2**A presents the correlation of the $\frac{E_{\mathrm{vdW}}}{E_{\mathrm{total}}}$ and the *f* of 92 layered crystals. We found a linear correlation exists between *E*_vdW_ and *E*_total_ following the relationship presented in Equation (5):

(5) $$\frac{E_{\mathrm{vdW}}}{E_{\mathrm{total}}}\approx 1-f$$

Therefore, for highly polar crystals such as Ca(OH)_2_ (*f* = 0.774), only ≈25% of the interlayer interaction is vdW in nature. This is the case for many oxides and halogenated crystals (the contribution of non‐vdW binding energy is mostly controlled by ionicity). Whereas, relatively low electronegativity of tellurium, for instance, causes low polar bonding with metals, and therefore, in most of tellurides, nearly all the binding energy is vdW in nature.^[^ ^18^ ^]^ We estimated the total binding energy of 107 layered crystals using this empirical relation. Figure 2b compares the empirically computed binding energies with those derived from ACFDT‐RPA, which shows a mean absolute relative error of 9.2%. Interestingly, the agreement of our approach with ACFDT‐RPA is better than almost all vdW‐DFT approaches for similar materials.^[^ ^24^ ^]^ The agreement between the empirical binding energies and the re‐scaled DFT calculations is inferior in some cases (Figure 2C),^[^ ^10^ ^]^ partly due to the inaccuracy of defined ionicity for complex crystals. Clearly, it is not just the ionicity of the bond that controls the contribution of the non‐vdW, as both charge distribution and the stacking configuration of the layers actively contribute to non‐vdW interactions. For instance, in the case of hBN, AA´ stacking causes larger electrostatic attraction compared to AB stacking.^[^ ^25^ ^]^ Therefore, in case of polar crystals, larger error is expected.

### 2.1 The Origin of Universal Binding Energy

**Figure 3:**
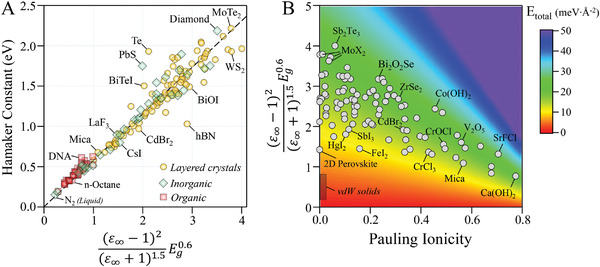
Origin of the universal binding energy in layered crystals. Panel A) shows Hamaker constants for 169 materials computed using Equation (1). They follow the modified Tabor–Winterton model Equation (6). For 56 materials, self‐consistent dielectric functions (at least for one of the optical axes) compiled from the experimental data were used.^[^ ^19a^ ^]^ The dielectric functions of the rest of the materials are approximated using the modified harmonic oscillator introduced by us in our recent work.^[^ ^19a^ ^]^ The list contains 96 layered crystals (orange circles), 35 organic (red squares), and 38 inorganic (green diamonds) insulators and semiconductors. Full list of materials along with their optical band gap, electronic dielectric constant, and the calculated Hamaker constant are given in Table S1, Supporting Information. Panel B) Two indices, namely Pauling ionicity and $\frac{{\left(\varepsilon_{\infty }-1\right)}^{2}}{{\left(\varepsilon_{\infty }+1\right)}^{1.5}}E_{g}^{0.6}$ are plotted against each other for 96 layered crystals. Unit of optical band gap is in electron volt. The binding energies are approximated from Equations (3), (5), and (6). This map identifies crystals with very large or very low binding energies which can be used to distinguish easily exfoliable crystals. Interestingly, vdW solids, such as Teflon, exhibit the lowest level of *E*_total_, indicative of very low *E*_vdW_, which explains why such materials exhibit very low surface energy levels. Mo*X*_2_ refers to molybdenum dichalcogenides (*X* = S, Se, and Te).

Our empirical approach allows us to approximate the binding energy of layered crystals from macroscopic vdW theory and $E_{\mathrm{total}}=-\frac{H}{12\pi d_{\mathrm{cut}-\mathrm{off}}^{2}\left(1-f\right)}$. We can then use this expression to understand what physical properties of crystals control the binding energy and why this energy is limited to a narrow range. The two main factors controlling the binding energy are the Hamaker constant and the polarity of the crystal. Hamaker constant, according to Equation (1), only depends on the optical constants of the material. We used our modified harmonic oscillator model for dielectric function as an input to Lifshitz theory and found (**Figure** **3**A; see Section S8, Supporting Information for derivation):^[^ ^19a^ ^]^

(6) $$H=0.577\frac{{\left(\varepsilon_{\infty }-1\right)}^{2}}{{\left(\varepsilon_{\infty }+1\right)}^{1.5}}E_{g}^{0.6}$$

The units of H and *E*_g_ are electron volt.^[^ ^27^ ^]^ Equation (6) is a modified version of the Tabor–Winterton model for Hamaker constant.^[^ ^28^ ^]^ While mathematically there is no bounds to a Hamaker constant according to the above equation, causality and optical sum rules impose a bound on *ε*_∞_ for a given *E*_g_. A passive material is causal and must obey the Kramers–Kronig relations.^[^ ^29^ ^]^ If dielectric function is approximated as the sum of lossless Drude–Lorentz oscillator, then *ε*_∞_ can be given as Equation (7):^[^ ^30^ ^]^

(7) $$\varepsilon_{\infty }=1+\sum_{i,\;\;\omega_{Ti}\geq E_{g}}^{\infty }S_{i}{\left(\frac{\omega_{p}}{\omega_{Ti}}\right)}^{2}$$

where, *ω*_p_ is plasma frequency and $\omega_{p}=\sqrt{\frac{e^{2}N}{\varepsilon_{0}m_{e}}}$;

Where, *e*, *m*_e_, *ε*_0_, and *N* are electron charge, mass of electron, permittivity of vacuum, and atom density, respectively. *ω*_*Ti*_ is the energy of *i*th excited transition with oscillator strength of *S_i_*. f‐sum rule mandates that $\sum_{i}^{\infty }S_{i}$ is equal to the total number of electrons.^[^ ^30^ ^]^ At large photon energies, the term $\frac{\omega_{p}}{\omega_{Ti}}$ diminishes, and therefore, the summation needs to be taken over the conduction band states and consequently $\sum_{i}S_{i}$ only accounts for valence electrons. This is however only true when the core states are well‐separated from the valence band.^[^ ^30^ ^]^ When a material contains heavy elements, the contribution of core states is not negligible. One can define a cut‐off energy below which most of (e.g., 99%) the transitions dominating *ε*_∞_ are located. An effective number of valence electron, *N*_eff_, can be then defined as the sum of oscillator strength up to this cut‐off energy, which can be computed from the dielectric function using f‐sum rule.^[^ ^31^ ^]^ For instance, we found *N_eff_* is 61% larger than the nominal value in CdBr_2_ (*E_g_* = 4.6 eV) whereas for hBN (*E_g_* = 5.8 eV), *N_eff_* is 22% smaller than the nominal value (See Section S9, Supporting Information for details). For narrow band gap semiconductors, despite containing heavy elements, *N*_eff_ is comparable to nominal valence electrons, because the cut‐off energy is small. Overall, *N*_eff_ directly correlates with the band gap and atomic number of elements in a crystal. We can now rewrite Equation (7) using only one effective term which turns into a modified version of Penn's model, Equation (8):^[^ ^32^ ^]^

(8) $$\varepsilon_{\infty }=1+N_{\mathrm{eff}}{\left(\frac{\omega_{p}}{E_{P}}\right)}^{2}$$

Where, *E*_P_ is Penn's band gap which represents the average position of electronic polarization bands. Applying this presentation of dielectric constant to Lifshitz theory, Hamaker constant can be approximated as follows (Equation (9), see Section S10, Supporting Information for derivation):

(9) $$H=\frac{3}{64\sqrt{2}}\;N_{\mathrm{eff}}\;\frac{\omega_{p}^{2}}{E_{P}}=\frac{3}{64\sqrt{2}}\;\frac{{\bar{\omega }}_{p}^{2}}{E_{P}}$$

Where, ${\bar{\omega }}_{p}$ represents the effective valence electron plasma frequency. This characteristic frequency is controlled by the type of constituting elements, bond length (density), and band gap (which depends on ionicity).^[^ ^18^ ^]^ In addition, *E*_P_ depends on the band gap and is very close to the characteristic frequency introduced in our modified harmonic oscillator model.^[^ ^19a^ ^]^ We note that the above approximation is of paramount importance to understanding of factors controlling *E*_vdW_ of materials.

The variation in magnitude of *E*_vdW_ in layered crystals can now be explained using Equation (9) as well. For instance, for two semiconductors with an identical band gap of ≈1.3 eV, that is, 2H‐MoS_2_ and InSe, the much larger valence electron density of MoS_2_ leads to an ≈55% bigger *E*_vdW_ compared to InSe (see Section S16, Supporting Information). In the cases of BiOCl and CrCl_3_, with similar band gap and valence electron density, the fact that the former contains bismuth, leads to an *E*_vdW_ value of more than twice compared to the *E*_vdW_ of CrCl_3_. Crystals containing light elements, with large band gap and low valence electron density, show the lowest vdW binding energy, such as Ca(OH)_2_, whereas semiconductors containing heavy elements and large valence electron density exhibit the largest *E*_vdW_ such as MoTe_2_.

The above findings shed light on the origin of the observation of a narrow range of binding energies in layered crystals (mostly between 15 and 25 meV∙Å^−2^). To understand this concept, direct correlation of ionicity and *E*_g_ needs to be considered.^[^ ^18^, ^33^ ^]^ In molecular vdW solids, such as rare gas solids, extremely large *E*_g_ (>20 eV) is observed due to the non‐covalent bonding in 3D despite negligible ionicity.^[^ ^34^ ^]^ However, in‐plane covalent bonding in vdW layered crystals dictates the large ionicity observed for wide band gap layered insulators (Figure S9, Supporting Information). In principle, layered crystals with larger *E*_g_ contain lighter elements and exhibit larger ionicity. Therefore, according to Equation (9), *E*_vdW_ is small but the contribution of non‐vdW is large as stated by Equation (5). On the other hand, semiconductors with small *E*_g_ are regularly less polar and they likely contain heavier elements (as electronegativity contrast between metals and non‐metals fades in higher period of periodic table), meaning they exhibit larger *E*_vdW_ which accounts for almost all the interlayer binding energy. It is therefore the counterbalance of factors that control the *E*_g_ and ionicity of the layered crystals that results in a narrow range of interlayer binding energies observed for this class of materials.

Exceptional cases can be spotted based on the above general rules where the binding energy is weaker than the typical layered crystals such as MoS_2_, about 20 meV∙Å^−2^. Such cases typically have low ionicity, relatively large band gap, and more importantly, small valence electron density. We can name layered Ruddlesden–Popper perovskites, namely (C_4_H_9_NH_3_)_2_(CH_3_NH_3_)_3_Pb_4_I_13_ as a typical example. This crystal has an *E*_g_ of ≈2 eV, a large portion of light elements, and very low polarity.^[^ ^35^ ^]^ More importantly, its valence electron density is ≈0.164 Å^−3^ which is nearly half of the value calculated for WS_2_ or MoS_2_ (See Supporting Information for details). This translates to a binding energy of 7.2 meV∙Å^−2^, which is almost a third of the typical value for TMDs (Figure 2A).^[^ ^10^, ^13^, ^24^ ^]^ Such a low binding energy manifests itself in the form of facile exfoliation of this crystal to very large monolayers.^[^ ^35^ ^]^ Other layered crystals can be evaluated using the same logic which makes it possible to distinguish easily exfoliable crystals. Examples are many CdI_2_‐type metal diiodides, BiI_3_, InTeI, and CrCl_3_ which mostly have low valence electron density and ionicity, but their band gaps are located around visible region. Figure 3B illustrates the criterion discussed above in a more quantitative way, providing a clear picture on why many layered crystals have very similar binding energies regardless of their electronic nature.

## 3 Conclusion

In conclusion, our findings present a coherent framework explaining that three fundamental characteristics of layered crystals, namely their effective valence electron plasma frequency, band gap, and ionicity, control their binding energy. While vdW binding energies of condensed matters span ≈three orders of magnitude across different materials,^[^ ^36^ ^]^ binding energies of layered crystals are constrained to a very narrow range. This range is dictated by the inverse correlation of optical band gap and ionicity of this class of crystals and fundamental limitations imposed by causality and f‐sum rules on dielectric response of crystals. Our findings provide an unambiguous methodology to search for easily exfoliable crystals based on their macroscopic properties, ultimately leading to a universal platform to design crystals with low binding energies. Very low exfoliation energy is; therefore, expected for crystals made of light elements with low polarity and density but with a large band gap, such as organic 2D polymers,^[^ ^37^ ^]^ or non‐covalently bonded molecular crystals.^[^ ^38^ ^]^ Binding energy at heterointerfaces of layered materials can also be quantified from our results assuming that additivity is valid for polar and dispersive interactions, an approach which has been successfully applied for liquid–solid heterointerfaces.^[^ ^21^ ^]^ Moreover, the accrued understanding here can be extended to other class of materials to decouple contribution of vdW interactions from their binding energies.^[^ ^39^ ^]^

## 4 Experimental Section

Experimental data was compiled on crystal structure, density (calculated from the X‐ray diffraction [XRD] data if possible), optical band gap, and dielectric tensor (both electronic and static) of 107 layered crystals. Note that not for all these crystals, the whole list mentioned above was available (details are given in Section S16, Supporting Information). From these data, Hamaker constants were calculated.^[^ ^19a^ ^]^ Pauling ionicity was estimated from the tabulated electronegativity of the elements.^[^ ^40^ ^]^ Theoretical binding energies listed in Section 16, Supporting Information, are referring to the calculations by Björkman et al.^[^ ^24^ ^]^ or Mounet et al.^[^ ^10^, ^41^ ^]^ The empirical binding energy of each material is computed using the Hamaker constant and the Pauling ionicity employing Equations (1) and (5) from the main text.

Data regarding space group, crystal structure, cell parameters, and calculated density were extracted from crystallographic information file (CIF) as defined by the International Union of Crystallography given by the open‐access crystallography open database (COD) using Match! software. Only validated CIFs were used. The CIFs were also validated to check whether all necessary items such as cell parameters, space groups, and crystal structures were consistent with each other. For materials for which the CIFs were not reported by COD and could not pass the checkCIF procedure, it was decided not to include the crystallography information; although, in some cases many files were available for such materials. The density was then calculated using the parameters reported in CIFs. To visualize the packed crystal structure and generating packing diagrams, Mercury software offered by The Cambridge Crystallographic Data Centre combined with POV‐Ray was employed to render high quality ray‐traced images.^[^ ^42^ ^]^

Optical band gap of insulators and semiconductors was usually quantified from the absorption spectra at the fundamental absorption edge of the crystals. For each crystal, the reference(s) from which the band gap was taken were cited. The optical band gap perpendicular to C‐axis was reported. The uncertainty in optical band gap varies for different crystals. Efforts were made to cite a value which had been reported more frequently by different researcher groups in cases where inconsistencies were observed. For some cases, however it was necessary to rely on the limited number of references available.

Dielectric constants of the layered crystals were presented in tensor form to address the optical anisotropy. The off‐diagonal components of the tensor were always considered to be zero for simplicity. The diagonal components represented the electronic (or static) dielectric constants in‐plane and out‐of‐plane. Often, the layered crystals were considered to be of isotropic nature in‐plane, unless otherwise mentioned. Regardless of the method of the measurements, the source of the dielectric tensor listed for each material was cited. Again, efforts were made to cite more frequently reported values if there were any inconsistencies. It should be noted that for a significant number of crystals, data for out‐of‐plane dielectric constant is not reported in the literature. Therefore, the anisotropy is ignored in such cases. The impact of this assumption is discussed in Section S4, Supporting Information. In addition, for some crystals, the electronic dielectric constant was calculated using the developed empirical formula. The contribution of the infra‐red absorption bands was included wherever it was possible; however, the impact of these bands was negligible.^[^ ^19a^ ^]^ Therefore, for those crystals for which these data are not available, a notable change was not expected in Hamaker constant. For metallic layered crystals, the full spectrum of the dielectric function if such data were available (see Section S12, Supporting Information) was used. In addition, it was assumed that all crystals were non‐magnetic. In the frequency ranges which has the highest contribution to vdW forces (typically visible and UV range), magnetic permeability is always close to unity, thus ignoring the magnetism seems to be reasonable.^[^ ^43^ ^]^ Details of computation of Hamaker constant are given in Section S3, Supporting Information.

The ionicity of the crystals was quantified using the Pauling's scale. For monoatomic crystals (graphite and tellurium), the ionicity was assumed to be zero. For bi‐atomic crystals, it was trivial to use Equation (S8), Supporting Information. For more complex crystals, the average ionicity of the metal and non‐metal bonds was often used. The details are given for each material in Section S16, Supporting Information.

As mentioned above, the theoretical binding energies were taken from the work of Björkman et al.^[^ ^13a^ ^]^ or Mounet et al.^[^ ^10^, ^41^ ^]^ The former reference was used, for those crystals for which their binding energy was computed by ACFDT‐RPA approach. For other crystals, the values computed using density functional theory (rVV10 vdW functional) were used. In these cases, the data were re‐scaled by a factor of 0.66 for better accuracy as proposed by Björkman et al.^[^ ^13a^ ^]^ See Section S6, Supporting Information, for details of benchmarking.

## Conflict of Interest

The authors declare no conflict of interest.

## Supporting information

Supporting Information

## Data Availability

The data that support the findings of this study are available from the corresponding author upon reasonable request.
