# Supplementary material for: Mapping the Binding Energy of Layered Crystals to Macroscopic Observables
Source: Adv Sci (Weinh). 2022 Oct 17;9(33):2204001. doi: 10.1002/advs.202204001 (PMC9685473; doi:10.1002/advs.202204001)
Supplement: Supplementary file 1 — Supporting Information [file ADVS-9-2204001-s001.pdf]

ADVANCED SCIENCE
Open Access

# Supporting Information

for *Adv. Sci.*, DOI 10.1002/advs.202204001

Mapping the Binding Energy of Layered Crystals to Macroscopic Observables

*Mohsen Moazzami Gudarzi* and Seyed Hamed Aboutalebi*

# Supporting Information

## Mapping the Binding Energy of Layered Crystals to Macroscopic Observables

*Mohsen Moazzami Gudarzi*, and Seyed Hamed Aboutalebi*

M. M. Gudarzi

National Graphene Institute; University of Manchester, Manchester, UK

Department of Materials, School of Natural Sciences; The University of Manchester, Manchester, UK.

E-mail: mohsen.moazzamigudarzi@manchester.ac.uk

S. H. Aboutalebi

Condensed Matter National Laboratory; Institute for Research in Fundamental Sciences, Tehran, 19395-5531, Iran.

E-mail: sha942@uowmail.edu.au

**This file includes:**

Supplementary Text
Sections S1 to S16
Figs. S1 to S130
Table S1 to S3
References (1 to 412)

**Supplementary Text**

**Section 1- On the origin of the universal cut-off distance:**

The cut-off distance we used to compute the vdW binding energy is purely empirical.[1] This distance is found from analysis of the experimental dispersive surface energies and Hamaker constants of wide ranges of solids and liquids. The Hamaker constant is computed from experimental optical constants and densities. Therefore, there is no adjustable parameter to arrive at a cut-off distance ( $d_{cut-off}$ ) of 1.66 ± 0.09 Å for 52 different materials (Details are given in section 15). This empirical observation can be traced back to 1960's.[2, 3] Even though flaws behind the extrapolation of Lifshitz's theory to this length scale was known [4], remarkable agreement with experimental data made this approximation a basis for estimating adhesion forces at the heterointerfaces which are critical in analysing wetting and spreading of liquids on solids.[5, 6] This cut-off distance is often referred to as molecular length[5], or minimum vdW separation distance[6] or interatomic distance[2], yet there is no reason to relate this cut-off distance to interatomic distances. Parsegian stated that '*The "interfacial energy" between two surfaces that are only ~1Å apart is an illusion.*' (See page 35 of ref.[7]).

One can in principle keep this analysis entirely an empirical observation and use Hamaker constants of materials to estimate their dispersive surface energy. Here, we however present a potential mechanism why for many materials the ratio of Hamaker constant to dispersion surface energy is nearly a universal value.

As discussed in the main text, eq. 1 (derived from Lifshitz theory[7]) is not valid near the contact region (See ref.[8] for details). The complexities associated with non-additivity and many body effects have made accurate estimation of vdW energy profile in this range of intermolecular distances very challenging. A popular microscopic approach to tackle this problem is atom pairwise additive approach with inclusion of damped higher order dispersion terms rather than just the leading $C_6 \cdot d^{-6}$ term (as in Hamaker approach).[9, 10] It still remains a challenging task to apply this approach to solids due to various forms of non-additivity. Yet, the major outcome of inclusion of higher order dispersion terms is enhancement of vdW interactions at short distances compared to leading $C_6 \cdot d^{-6}$ term. This also results in deviation of power laws of vdW interactions between atoms at short distances from $d^{-6}$.[8] Figure S1 shows vdW interactions between a pair of Krypton atoms using only $C_6 \cdot d^{-6}$ term and higher order terms (up to $C_{20} \cdot d^{-20}$ term). At distances below 7 Å, the leading dispersion term fails to follow the *ab initio* energy potential[11] whereas inclusion of higher-order terms accounts accurately for vdW interactions. On top of computation of attractive vdW component of energy profile, one needs to include the Pauli repulsion to capture the final energy profile from which the binding energy and the equilibrium distance can be inferred.

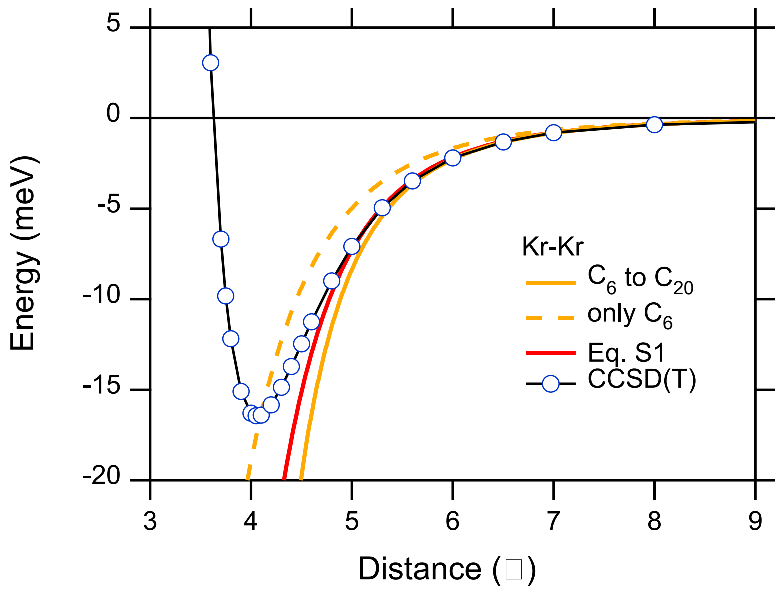


**Fig. S1. Interaction energy profile for a pair of Krypton atoms.** *The blue circles show the ab initio calculations by Slavíček et al. using the coupled-cluster method covering single- and double-excitations iteratively and triple excitations perturbatively, CCSD(T).*[11] *The orange dashed line shows the* $C_6 \cdot d^{-6}$ *term and the orange line shows the sum of* $C_{2n+4} \cdot d^{-2n+4}$ *terms for n = 1 to 8. The red line shows the prediction of eq. S1. For* $C_6$ *the reference value given in ref.*[9] *is used. The dispersion coefficients* $C_8$ *and* $C_{10}$ *are the reference values given in ref.*[12]. *The higher order coefficients are calculated as suggested in ref.*[9]

The vdW interactions between two half-spaced solids become further complicated as the ‘effective’ dispersion coefficients of atoms in solids are different compared to free atoms.[13] In the absence of other short range forces, finally the counterbalance of vdW attraction and Pauli repulsion controls the equilibrium distance. At large distances (few times of interatomic distances), however, the scaling law given by eq. 1, *i.e.* $d^{-2}$, recovers (see the main text). This power law behaviour can be simply obtained by pairwise summation of $C_6 \cdot d^{-6}$ term (Hamaker approach)[10], which is also captured by Lifshitz theory[14] and state-of-the-art adiabatic-connection fluctuation-dissipation theorem (ACFDT)[15]. A large number of direct force measurements has confirmed this scaling law (through the proximity approximation) and in our recent work we showed that once accurate self-consistent dielectric functions of materials are used for computation of vdW forces, experimental data and Lifshitz theory agree[16]. Therefore, it is expected that the vdW energy profile between ideally smooth interfaces follows eq. 1 up to distances close to 10 Å. In analogy to interatomic vdW energy, we expect that eq. 1 underestimate the vdW energy at short distances. We now discuss how this likely additional attractive contribution might be the origin of observation of universal cut-off distance for various liquids and solids.

Let’s recall the vdW energy profile of Krypton pair (Fig. S1). One way to account for higher order dispersion terms is to shift the interatomic distance as follow (eq. 8.10 in ref.[8]):

$$E_{vdW}(d) = -\frac{C_6}{(d^2 - \frac{C_8}{3C_6})^3}. \qquad \text{(S1)}$$

This can be interpreted as an effective distance if only $C_6 \cdot d^{-6}$ term is being used. The distance $\sqrt{\frac{C_8}{3C_6}}$ progressively increases as the size of atoms (*i.e.* the vdW radius) grows.[9] Therefore, it can be inferred the denominator of above equation changes less significantly as the vdW equilibrium distance increases. Similarly, Lifshitz-Zaremba-Kohn theory[17] for vdW

interaction between an atom and a solid surface introduces a reference plane position, $Z_0$, where vdW energy profile follows:

$$E_{vdW}(d) = -\frac{C_3}{(d-Z_0)^3}. \qquad \text{(S2)}$$

The distance $Z_0$ is also related to interlayer distance in solid [17, 18]. However, extrapolating these analyses to interactions between two solids is not trivial.

A more sensible analysis would be to compare the '*accurate*' vdW energy profile between two parallel half-spaced solids with the prediction of eq. 1. Glould *et al.*[19] has explicitly computed the vdW interactions between two slabs of graphite and hexagonal boron nitride (hBN) using ACFDT within the random phase approximation (ACFDT-RPA). Figure S2 compares their computed vdW energy with the energy profile given by eq. 1. At large distances (above 8 Å), two profiles nearly overlap. Indeed, same authors have shown that in the limit of $d \to \infty$ , one arrives to[15]:

$$E_{vdW}(d \to \infty) = -\frac{C_2}{d^2}, \qquad \text{(S3)}$$

Therefore, the power law predicted by eq. 1 is also recovered at large distances.

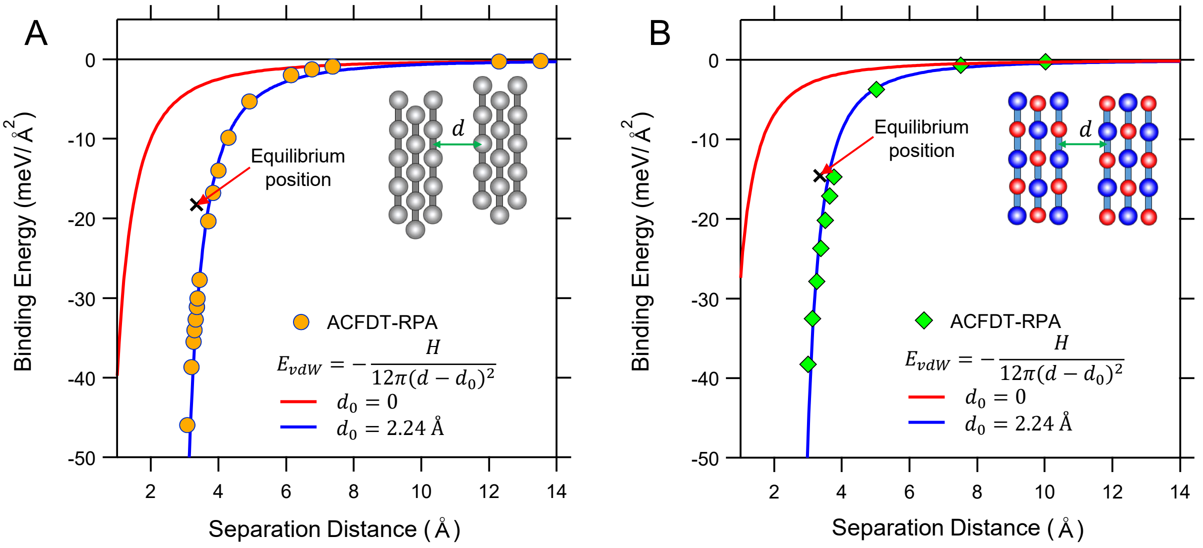


**Fig. S2. vdW interaction energy between two slabs of graphite and hexagonal boron nitride (hBN) as a function of separation distance**. *vdW attraction between (A) graphite and (B) hBN are shown. The symbols show the calculations of Gould et al.*[19] *using ACFDT-RPA method for graphite (orange circles) and hBN (green diamonds). Their data are normalized to area of atoms in graphite and hBN using their lattice constant (see Figs. S40 and S41). Red and blues lines are prediction of Lifshitz theory without a shift and with distance shift of 2.24 Å, respectively. Hamaker constant was fixed to 1.495 eV for graphite and 1.032 eV for hBN. See Figs. S40 and S41 for details. The equilibrium position is the theoretical one obtained from ACFDT-RPA calculations*[20, 21].

Near the contact region (below 6 Å), as expected from earlier discussion, the vdW attraction is stronger than the prediction of eq. 1. It is however possible to approximate the vdW energy profile, for both graphite and hBN, using eq. 1 by introducing a distance shift of 2.24 Å. This distance shift, $d_0$, is correlated with the vdW thickness of layers in layered crystals. In the absence of other intermolecular forces, Pauli repulsion counterbalances the vdW attraction and controls the equilibrium distance and binding energy.[20] Another distance shift (difference of equilibrium distance and the [effective] onset of Pauli repulsion, $d_1$) should thus be added to eq. 1, and $E_{vdW} = -\frac{H}{12\pi(d-d_0+d_1)^2}$. These effective distance shifts are not universal values, but the difference of the equilibrium and shift distances is. In our view, and in analogy to

interactions between atoms or atom with solid interfaces, the Hamaker constant presents the effective leading dispersion term whereas the distance shift $d_0$ compensates for higher order vdW interactions terms. This distance shift is correlated with vdW thickness. This could also explain why the universal $d_{cut\text{-}off}$ (which is the difference of the layer thickness and the distance shifts) is very similar to the so-called vdW gap (See section 2).

**Section 2- On similarity of vdW gap and the universal cut-off distance:**

First, we calculated the vdW gap for 86 elements using the covalent ($r_C$) and vdW ($r_{vdW}$) radii as follows:

$$d_{vdW} = 2(r_C - r_{vdW}). \qquad \text{(S4)}$$

We used covalent radii reported by Cordero *et al.*[22]. vdW radii reported by Alvarez[23] and Batsanov[24] are slightly different (Figure S3A). The average vdW gap of 86 elements based on Alvarez's data is 1.85 ± 0.26 Å, whereas it is 1.38 ± 0.33 Å using Batsanov's recommended data (for 65 elements) (Fig. S3). Average of vdW gap based on these data sets is 1.66 ± 0.25 Å (Figure S3B). Pauling has also suggested a similar range for vdW gap, *i.e.* 1.5-1.66 Å (See page 263 of ref.[25]). It should be noted that the vdW gap of halogens and chalcogens are distinctively smaller than most metals. Therefore, a simple interpretation is that the actual vdW gap of many crystals covers a narrower range. Indeed, the cut-off distance computed from experimental surface energies and Hamaker constants of 52 liquids and solids, matches with the average vdW gap of the elements in periodic table, but with much smaller variation (1.66 ± 0.09 Å), see Figure S3B and S3C.

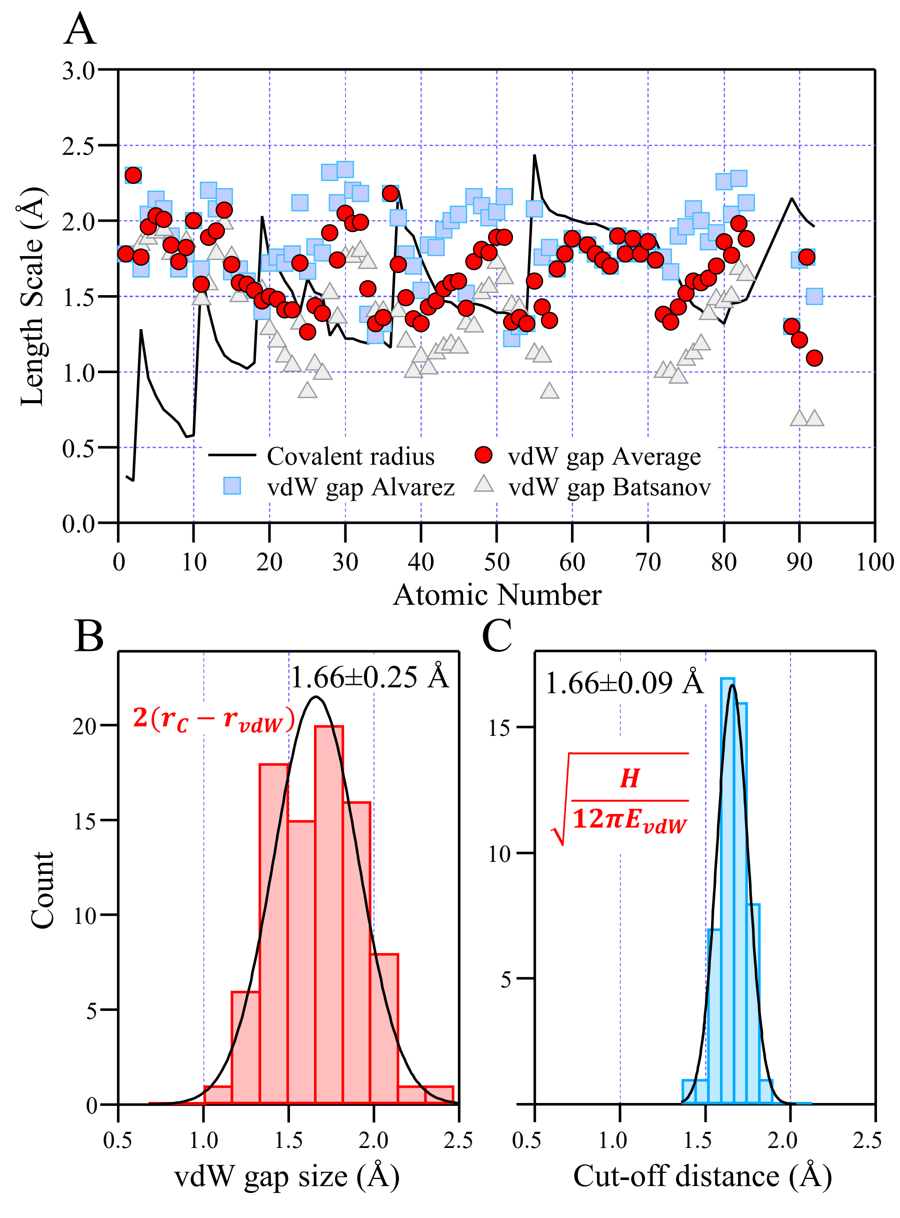

**Fig. S3. Analysis of the vdW gap.** *Panel (A) shows the covalent radii (black line), and the vdW gaps (symbols) for 86 different elements versus their atomic number. Red circles are the average value of the vdW gap (derived from eq. S4) based on the reported vdW radii by Alvarez and Batsanov. Panel (B) shows the distribution of vdW gap (shown by red circles in top panel). Panel (C) shows the cut-off distance calculated from the experimental dispersive surface energy and Hamaker constants of 52 different materials (details are given in Table S3). The errors refer to the one standard deviation of the distribution.*

**Section 3- Computation protocol of Hamaker constant:**

Given that we assumed a universal cut-off distance of 1.66 Å for all the crystals, then the vdW binding energy only depends on the Hamaker constant. We have used the non-retarded form of Lifshitz theory for anisotropic crystals, *i.e.* eq. 1, to compute the Hamaker constant. More details can be found in Parsegian's book[7]. The temperature was assumed to be 298 K unless otherwise stated. The summation was done up to photon energy of 300 eV. The only input to the model is the dielectric function of the crystal at imaginary frequencies. In principle, the dielectric functions in all optical axes and in very wide photon energies (namely from IR to soft X-ray) are required.[16] Such data are barely available for crystals. Therefore, we rely on approximating the dielectric functions where the experimental data were unknown.[16] The accuracy of this approach is discussed in section 5. Figure S4 shows the general overview of the protocol we followed to acquire the dielectric functions.

We have prioritized using the experimental dielectric function whenever such data were available. We followed the protocol explained in our recent work to evaluate the self-consistency of the dielectric functions.[16] In such cases, the sources of data and details of any post-processing has been given. For details see section 12 or ref.[16] If such data were not available, then we approximated the dielectric function using the modified harmonic oscillator model (See ref.[16] for details). In cases of semiconductors and insulators, this empirical model requires four inputs which are chemical composition, density, optical band gap ($E_g$) and electronic dielectric constant ($\varepsilon_\infty$). The electronic dielectric function at imaginary frequencies then follows:

$$\varepsilon(i\xi) = 1 + \frac{\varepsilon_\infty - 1}{1+\left(\frac{\xi}{\omega_{UV}}\right)^{\alpha}}, \qquad \text{(S5)}$$

where $\omega_{UV} = 3.05 \cdot {E_g}^{0.736}$. This is an empirical approximation and the unit is in electron volts[16]. The power exponent $\alpha$ is given by the following empirical relation[16, 26]:

$$\alpha = -0.36 \cdot \log\left(\frac{r_e \lambda^2}{\pi(\varepsilon_\infty - 1)E_g^{1.2}} \sum_i N_i {f_1}_i\right) - 0.545, \qquad \text{(S6)}$$

where $r_e$ is the classical electron radius, $\lambda$ is the wavelength and is chosen to be 1.24 Å, $N_i$ is the atom density of type $i$ in the crystal and $f_1$ is the real of part of the complex atomic scattering factor. The unit of band gap is electron volt. Please note that, in transcendental functions, it is meaningless to include dimensioned quantities, as the functions themselves cannot operate on quantities with physical dimensions. As such, before applying the power, we divide both band gap (or the Hamaker constant, see Eq. 6) to their unit value and obtain a dimensionless number (ratio). [27] The complex atomic scattering factors are given in CXRO database[26, 28].

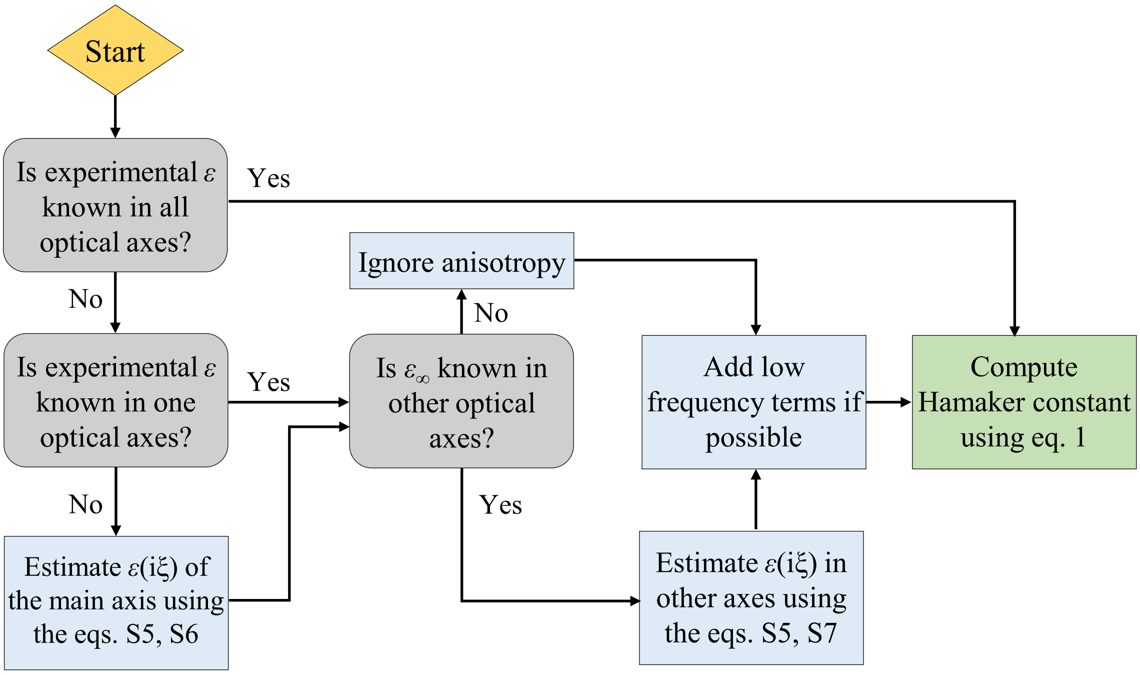


**Figure S4. Flowchart describing the protocol that has been followed throughout this work to compute Hamaker constants.**

Optical anisotropy is often large in layered crystals and therefore we have considered the optical anisotropy whenever it was possible. We have discussed the impact of ignoring the anisotropy in the next section. Again, the major problem is that the limited optical constants of layered materials are measured in out-of-plane polarization. We, therefore, mostly relied on another approximation to compute the dielectric function in other polarization.[16] The main assumption behind this approximation is that the anisotropy fades at very high photon energies (namely soft X-ray), except close to absorption edges of the inner electrons. Details are given in supplementary information of our previous work.[16] We assumed that power exponent $\alpha$ is the same in all axes for every crystal. Therefore, to use the modified harmonic oscillator model, *i.e.* eq. S5, it is just required to know the characteristic frequency, $\omega_{UV}$. If the electronic dielectric constant in the other optical axes are known, then the $\omega_{UV}$ for that axis can be derived from the $\omega_{UV}$ of the main axis as follow[16]:

$$\frac{\omega_{UV\perp}}{\omega_{UV\parallel}} = \left(\frac{\varepsilon_{\infty\parallel}-1}{\varepsilon_{\infty\perp}-1}\right)^{\frac{1}{\alpha}}. \qquad \text{(S7)}$$

Finally, we have included the polarization bands in infra-red region and/or those from free-electron (Drude band), if such data were available experimentally. For all 107 crystals that we have analysed, details of the sources where the data are taken from are given separately in section 16.

**Section 4- Impact of anisotropy on the magnitude of vdW interactions:**

For some of the layered crystals we analysed here, dielectric constants in out-of-plane polarization were not available. We had to therefore assume that these crystals are optically isotropic. Here we address the impact of this assumption on the outcome of the analysis. For 22 representative crystals we ignored the anisotropy and calculated the Hamaker constant just using the in-plane dielectric function. These Hamaker constants were then compared to those obtained from the anisotropic dielectric functions, Fig. S5. For graphite and hexagonal boron nitride (hBN), the difference was notable and the anisotropic Hamaker constants were about 25% lower than isotropic Hamaker constants. This indicates a significant anisotropy in these two crystals which are mono-atomically thin. For the rest of the materials, the deviation was mostly below 10%. It appeared that for crystals with thicker monolayers, such as $Bi_2Te_3$ or $Sb_2Te_3$, and $CdI_2$ type metal halides, the impact of ignoring anisotropy in the calculations is not significant. Overall, ignoring the anisotropy in dielectric function results in maximum 10% increase in the computed Hamaker constants. However, we have included the anisotropy in the dielectric function in our calculations wherever it was possible.

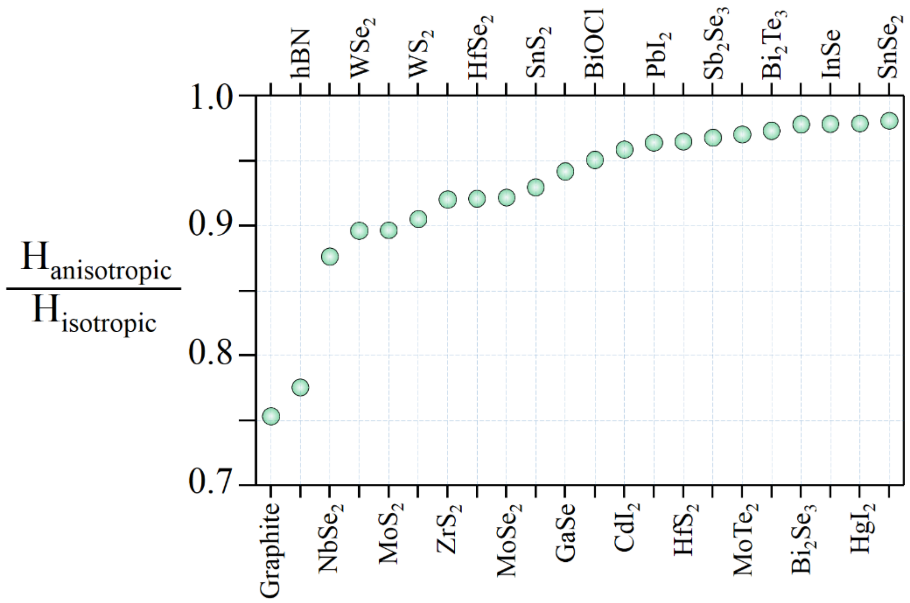


**Fig. S5. Impact of optical anisotropy on the Hamaker constant of the layered crystals.** The ratio of anisotropic Hamaker constant (derived from the anisotropic dielectric function) to the isotropic Hamaker constant (derived from in-plane dielectric function) for various layered crystals are given.

**Section 5- Accuracy of modified harmonic oscillator model to estimate the vdW interactions:**

We calculated the non-retarded Hamaker constant of 107 layered crystals. It is pertinent to mention that only for 27 crystals the full spectrum of dielectric function at least in one optical axis is known. For all other crystals, the calculations rely on estimating the dielectric function using the modified harmonic oscillator model.[16] However, there exists some level of uncertainty in the estimation of the dielectric function using the modified harmonic oscillator model. The input to the model is the chemical formula, density, optical band gap and electronic dielectric constant. Assuming the electronic dielectric constant to be an exact value, then the major error occurs in estimation of the characteristic frequency of the oscillator and the power exponent of the modified harmonic oscillator.[16] Propagation of these errors in calculation of the Hamaker constant is not trivial to compute analytically. Therefore, to provide a picture on the magnitude of the errors on the estimation of the Hamaker constant, we performed the following analysis.

For 16 layered crystals (insulators and semiconductors), we used their density, dielectric constants (static and electronic) and optical band gap to construct the dielectric function. For all of these crystal, the full spectrum of the dielectric function at least in one axis is known (See ref.[16] and Section 12). Then, Hamaker constant for each material was calculated using the full spectrum (exact value) or employing the modified harmonic oscillator model (approximated value). Fig. S6 compares the approximated Hamaker constant and the exact values for these crystals (we have also included rutile $TiO_2$ as an example of a non-layered anisotropic crystal). The standard error in the estimation of the Hamaker constant is found to be about 8%. Note that the assumption here is that the input to the model (density, band gap and dielectric constants) are accurate. The uncertainty in each of these parameters varies for different materials, and so does the error in the calculated Hamaker constant for each material.

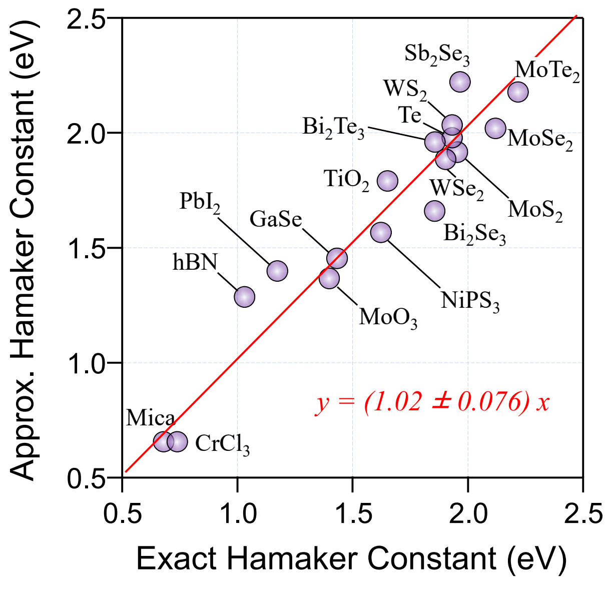


**Fig. S6. Comparison of the Hamaker constants calculated from the experimental dielectric constant (exact values) and those obtained from the modified harmonic oscillator model (approximated values).** *The parameters of the modified harmonic oscillator model are obtained directly from the density, band gap and dielectric constants.*[16]

**Section 6- Benchmarking of binding energy:**

Our approach to estimate the binding energy relies on two empirical indices, one being the cut-off distance and the other is an index for non-vdW interactions (ionicity). The cut-off distance is assumed be a universal value and obtained from analysis of experimental data on dispersive binding energy and optical constants of various materials (See section 1). On the other hand, our analysis of contribution of non-vdW interactions relies on benchmarking against theoretical binding energy. We used binding energies computed by ACFDT-RPA method (by Björkman *et al.*[21]) as the reference values. This method is often considered as *de facto* standard for computing dispersion forces in layered crystals.[29] However, such data were only available for 28 different crystals. For the rest of the crystals, we relied on semi-empirical values obtained from the rescaled rVV10 DFT calculations by Mounet *et al.*[30, 31] We corrected the binding energy reported by Mounet *et al.* by a factor of 0.66. This is based on the observation of Björkman *et al.*[21] However, we noted that Björkman *et al.* have used VV10 vdW correction as opposed to rVV10 correction in the work of Mounet *et al.* Our analysis, nevertheless, shows that the outcomes of both functional follow RPA's results with the same magnitude of error (Fig. S7).

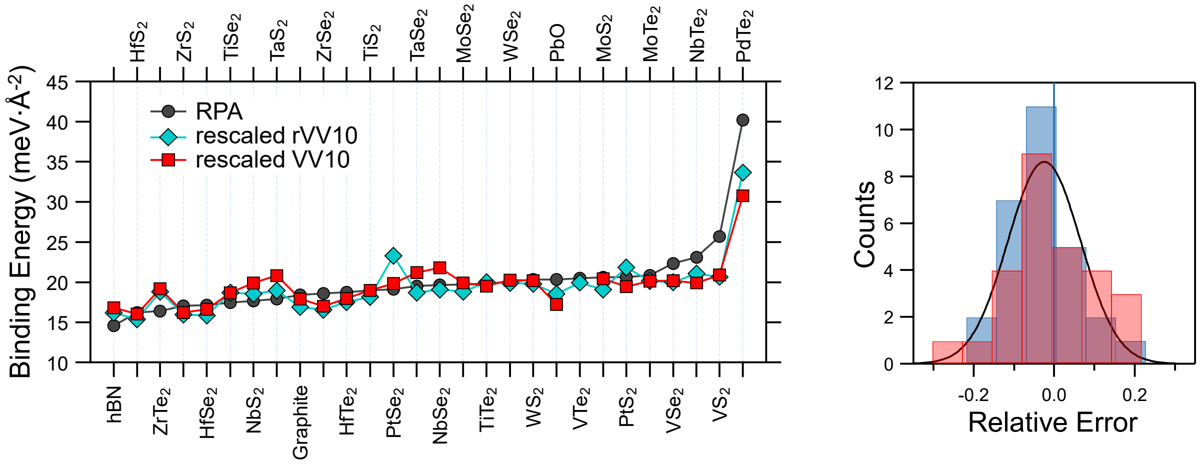


**Fig. S7.** *Left panel shows binding energy of 28 layered crystals computed with demanding ACFDT-RPA (labelled as RPA and shown by dark circles) reported in ref.*[21]*. Results of rescaled DFT calculations (VV10 from ref.*[21] *and rVV10 from ref.*[30]*, rescaled by a factor of 0.66) are compared with RPA outcomes. Right panel shows the error distribution of DFT methods relative to RPA approach. Error is defined as* $\frac{E_{DFT}-E_{RPA}}{E_{RPA}}$. *The standard deviation is about 9% in both cases.*

**Section 7- Ionicity scales and contribution of non-vdW interactions:**

The contribution of non-vdW interactions to the binding energies of layered crystals has been rarely explicitly addressed. For organic liquids and polymers, non-vdW interactions (also known as polar interactions) correlate with polarity of the molecules. Polar molecules such as water possess large surface energy mostly due to non-vdW interactions (about 70% at room temperature).[1] Quantification of polarity of organic molecules and likewise ionicity in crystalline solids has been heavily discussed and many approaches have been developed to quantify the ionicity.[1, 32, 33] A renowned approach is to use the Pauling's scale of electronegativity to compute the ionic nature of an $A-B$ bond:[32]

$$f_{Pauling} = 1 - exp^{-(\frac{X_A - X_B}{2})^2} \quad , \qquad \text{(S8)}$$

where $X_i$ is the electronegativity. Yet, this approach fails to consider the impact of other parameters in crystals such as symmetry (Pauling has suggested different relations for different crystal symmetries[34]). Other approaches, for instance those proposed by Phillips[33] or by Szigeti[35], rely on prior knowledge of dielectric function of the crystals, which limits their application to only well-characterized crystals. Furthermore, the correlation between different ionicity scales is not always linear and re-scaling one to another is not trivial. In principle, one can define a new ionicity scale as the ratio of non-vdW surface energy to the total surface energy. Our attempt in this work was to find a plausible ionicity scale to relate that to this newly defined scale. As shown in Figure 2A, the Pauling's ionicity appears to correlate with the ratio of non-vdW surface energy to the total surface energy linearly. Therefore, for the sake of simplicity but at the cost of losing some accuracy, we chose to use the Pauling's ionicity to calculate the total binding energy. For crystal with more than two atoms, we often use an average value of ionicity of bonds.

**Section 8- Modified Tabor-Winterton (TW) model of Hamaker constant (eq. 6):**

The Lifshitz theory requires knowledge of dielectric function of the interacting bodies. TW assumed[36] dielectric function is given by a narrow peak with a characteristic frequency of $\omega_{UV}$, [37]and by plugging this function to Lifshitz theory, which states[38]:

$$H = \frac{3}{4\pi}\int_0^{\infty}\left(\frac{\varepsilon(i\xi)-1}{\varepsilon(i\xi)+1}\right)^2 d\xi, \qquad \text{(S9)}$$

they arrived to the following relation for Hamaker constant[36]:

$$H = \frac{3\sqrt{2}}{32}\frac{(\varepsilon_\infty-1)^2}{(\varepsilon_\infty+1)^{1.5}}\omega_{UV}. \qquad \text{(S10)}$$

However, the response function of materials is far from the ideal case of a single narrow peak[16]. We have overcome this inefficiency by using the empirical modified harmonic oscillator model (eq. S5). The main difference of this model with harmonic oscillator with no damping is the introduction of the power exponent $\alpha$. For $\alpha = 2$ original TW model (eq. S10) is revived. However, $\alpha$ is experimentally found to be between 1.85 and 1.4.[16] Our numerical analysis of eqs. S5 and S9 shows that for any value of $\alpha$ (within the physical range), TW model can be modified to:

$$H \sim \frac{(\varepsilon_\infty-1)^2}{(\varepsilon_\infty+1)^{1.5-\delta}}\omega_{UV}, \qquad \text{(S11)}$$

where $\delta$ is small value and depends on $\alpha$. For instance, if $\alpha = 2$ or 1.5, then $\delta$ is 0 or $\frac{1}{6}$, respectively. We now recall the empirical relation of $\omega_{UV} = 3.05 \cdot {E_g}^{0.736}$.[16] Thus, eq. S11 becomes:

$$H \sim \frac{(\varepsilon_\infty-1)^2}{(\varepsilon_\infty+1)^{1.5-\delta}}{E_g}^{0.736}. \qquad \text{(S12)}$$

Also, $\varepsilon_\infty$ inversely scales $E_g$.[16] Therefore, above relation can be approximated as follow:

$$H \sim \frac{(\varepsilon_\infty-1)^2}{(\varepsilon_\infty+1)^{1.5}}{E_g}^{0.736-\delta'}. \qquad \text{(S13)}$$

The empirical factor $\delta'$ is a small quantity, within the same range of $\delta$. We then analysed $\varepsilon_\infty$, $E_g$ and Hamaker constants of 169 materials (96 layered crystals, 35 inorganic and 38 organic compounds), and found:

$$H \sim \frac{(\varepsilon_\infty-1)^2}{(\varepsilon_\infty+1)^{1.5}}{E_g}^{0.6\pm0.02}. \qquad \text{(S14)}$$

Note that above relation is empirical where unit inhomogeneity is allowed.[27]

**Section 9- Effective valence electron density:**

The number of valence electrons, the electrons in outermost shell of atoms, are nominally taken from the electron configuration.[39] For transition metals, it is not as trivial to define the number of valence electrons but it can be estimated from the oxidation state.[39] These electrons are dominating the absorption bands above the band gap and thus the electronic dielectric constant ($\varepsilon_\infty$).[40] Here, we are searching for the effective number of electrons associated with the transitions which control the magnitude of $\varepsilon_\infty$. The effective number of electrons up to a photon energy of $\omega_0$ can be obtained from *f*-sum rule and using the imaginary part of the dielectric function, $\varepsilon_2$, as follow[16, 40]:

$$n_{eff}(\omega_0) = \frac{2\varepsilon_0 m_e}{\pi N \hbar^2 e^2} \int_0^{\omega_0} \omega \varepsilon_2(\omega) d\omega \quad , \qquad \text{(S15)}$$

where $N$, $e$, $m_e$, $\varepsilon_0$, $\hbar$ are the atom density, elementary charge, mass of electron, vacuum permittivity and Planck's constant, respectively. We now need to define a cut-off energy ($E_{cut-off}$) where most of the transitions contributing to $\varepsilon_\infty$ are located. We recall the Kramers-Kronig relation and introduce a $E_{cut-off}$ which reads[16]:

$$\varepsilon_\infty - 1 = \frac{2}{\pi} \int_{E_g}^{\infty} \frac{\varepsilon_2(\omega)}{\omega} d\omega = \frac{2}{0.99\pi} \int_{E_g}^{E_{cut-off}} \frac{\varepsilon_2(\omega)}{\omega} d\omega. \qquad \text{(S16)}$$

Thus, $E_{cut-off}$ is a photon energy below which transitions bands contributing to 99% of $\varepsilon_\infty - 1$ are located. We then define the effective number of valence electrons by setting $\omega_0 = E_{cut-off}$ in eq. (S15) and we arrive at:

$$N_{eff} = \frac{2\varepsilon_0 m_e}{\pi N \hbar^2 e^2} \int_0^{E_{cut-off}} \omega \varepsilon_2(\omega) d\omega. \qquad \text{(S17)}$$

Note that the choice of 99% in eq. S16 is entirely arbitrary and we used this presentation to consistently compare $N_{eff}$ with nominal number of valence electrons for different crystals.

We used experimental $\varepsilon_2$ of three representative layered crystal to obtain $N_{eff}$ and to compare them with nominal values (Fig. S8). hBN and $CdBr_2$ are wide band gap crystals, but the latter contains heavier elements. On the other hand, band gap of $MoSe_2$ is in infra-red region but the atomic number of Mo and Se are close to Cd and Br, respectively.

Following the above protocol, we found $N_{eff}$ of hBN is 22% smaller than its nominal number of valence electrons. For $CdBr_2$, on the other hand, $N_{eff}$ is 61% larger than nominal value. This deviation nearly disappears in case of $MoSe_2$.

Overall, $N_{eff}$ is smaller than the nominal valence electron when crystal is made of light elements, but it is larger than nominal value for insulators made of heavier elements. Therefore, $N_{eff}$ shows a positive correlation with atomic number and band gap. We derived an empirical approximation (M. Moazzami Gudarzi, S. H. Aboutalebi; *Unpublished*) to estimate $N_{eff}$ which follows:

$$N_{eff} = 0.13 \cdot \beta \cdot E_g^{0.6} \cdot N_{VE}, \qquad \text{(S18)}$$

where $N_{VE}$ is the nominal number of valence electrons and unit of band gap is in electron volts. $\beta$ is the number average of electron shells of constituting elements of the crystal, and $\beta = \frac{\sum_i (L_i - 1) X_i}{\sum_i X_i}$ where $L_i$ is the number of electron shells of each element (*e.g.* 2 for oxygen and 6 for Bi.), and $X_i$ is the number of the element in the chemical formula of the crystal.

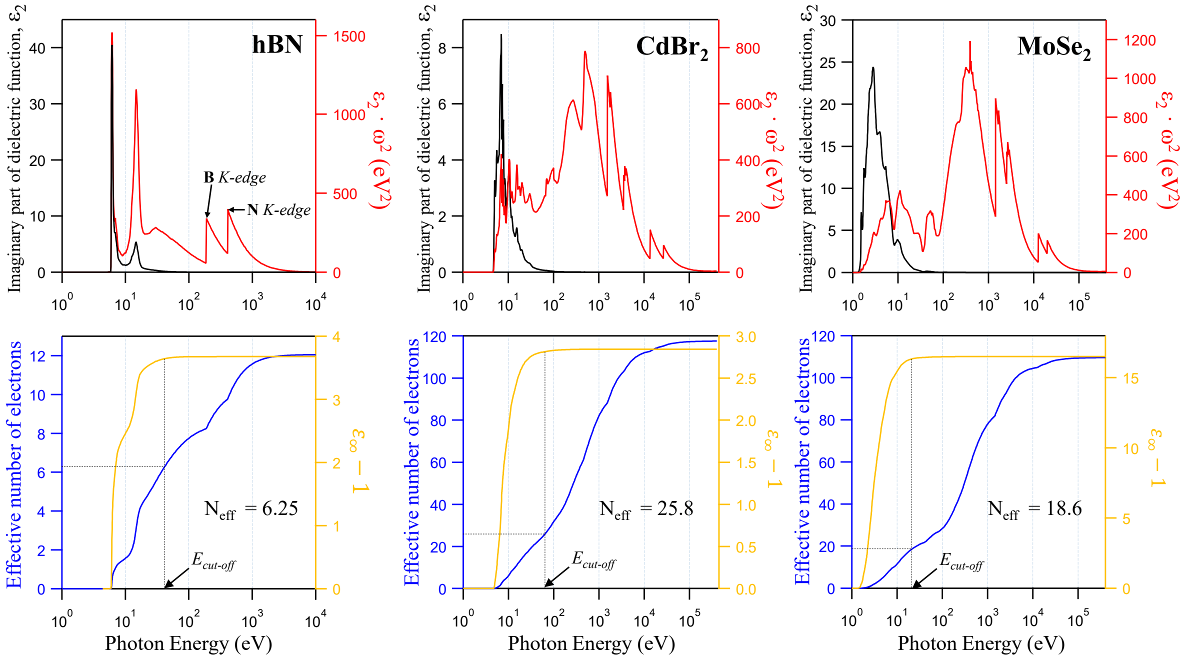


**Fig. S8. Computation of $N_{eff}$ for three representative crystals**, *namely hBN, $CdBr_2$ and $MoSe_2$. Top panels show the imaginary part of the dielectric function, $\varepsilon_2$, (black lines) along with $\varepsilon_2 \cdot \omega^2$ (red lines). The latter quantity shows the transition from core electrons more clearly. The bottom panels show the effective number of electron (blue line) and $\varepsilon_\infty - 1$(orange lines) as a function of photon energy. The cut-off energy ($E_{cut-off}$) and $N_{eff}$ are obtained following the protocol explained in the text.*

**Section 10 Derivation of equation 8:**

We recall the TW model for Hamaker constant which states[36]:

$$H = \frac{3\sqrt{2}}{32}\frac{(\varepsilon_\infty - 1)^2}{(\varepsilon_\infty + 1)^{1.5}}\omega_{UV}. \qquad \text{(S10)}$$

The characteristic frequency $\omega_{UV}$ is very close to Penn's band gap ($E_P$). In principle, dielectric function (below band gap) can be approximated as[41]:

$$\varepsilon(\omega) = 1 + \frac{{\omega_p}^2}{{E_P}^2 - \omega^2}. \qquad \text{(S19)}$$

Thus, at imaginary frequencies:

$$\varepsilon(i\xi) = 1 + \frac{{\omega_p}^2}{{E_P}^2 + \xi^2}. \qquad \text{(S20)}$$

In TW model, the dielectric function is approximated as follow[36]:

$$\varepsilon_1(i\xi) = 1 + \frac{(\varepsilon_\infty - 1){\omega_{UV}}^2}{{\omega_{UV}}^2 + \xi^2}. \qquad \text{(S21)}$$

Therefore, we can assume that $\omega_{UV} = E_P$. When $\varepsilon_\infty$ is small (< 10 ), we can write:

$$\frac{(\varepsilon_\infty - 1)^2}{(\varepsilon_\infty + 1)^{1.5}} \cong \frac{1}{4}(\varepsilon_\infty - 1). \qquad \text{(S22)}$$

Recalling the modified Penn's model from the main text (eq. 8):

$$\varepsilon_\infty - 1 = N_{eff}(\frac{\omega_p}{E_P})^2. \qquad \text{(S23)}$$

Therefore, by combining eqs. S10, S22 and S23, TW model modifies to:

$$H = \frac{3}{64\sqrt{2}} N_{eff} \frac{{\omega_p}^2}{E_P}. \qquad \text{(S24)}$$

**Section 11- Correlation of the band gap and ionicity:**

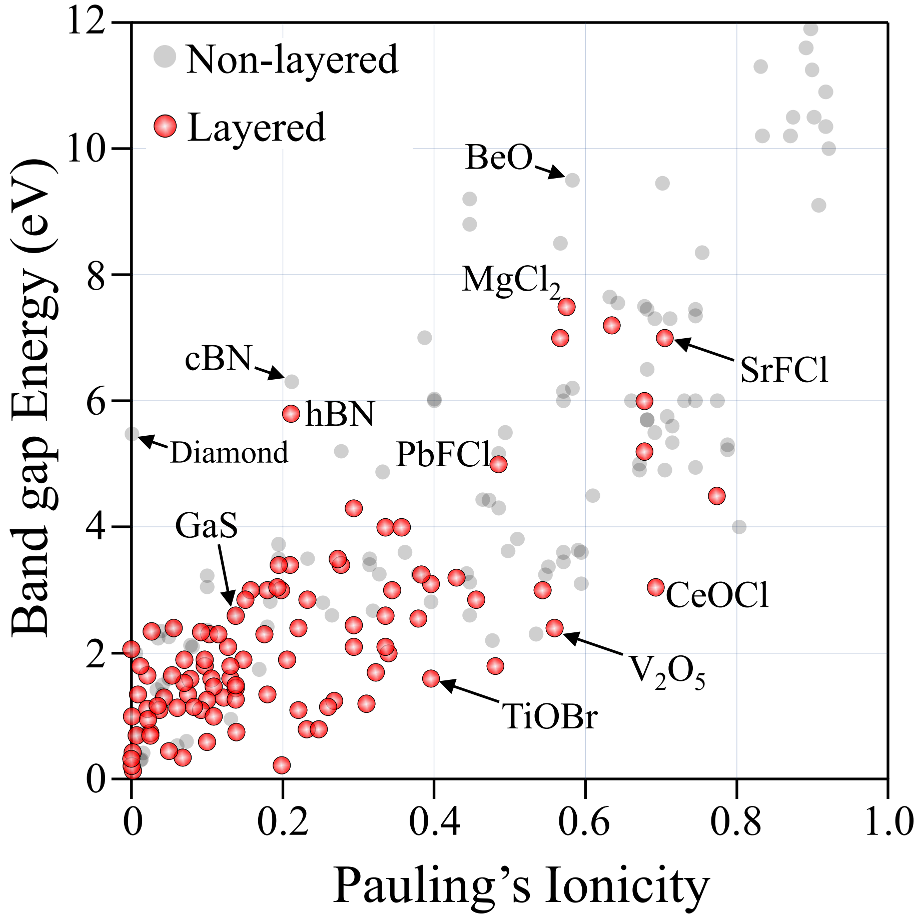


**Fig. S9. Correlation of the ionicity and optical band gap of solids.** Optical band gap of 211 solids (99 layered and 112 non-layered crystals) are shown versus their Pauling's ionicity. We should stress that the large band gap does not necessarily imply large ionicity, as in diamond. The ionic character of band gap of solids has been discussed in details by Phillips.[33]

**Section 12- Self-consistent dielectric functions of 14 layered crystals:**

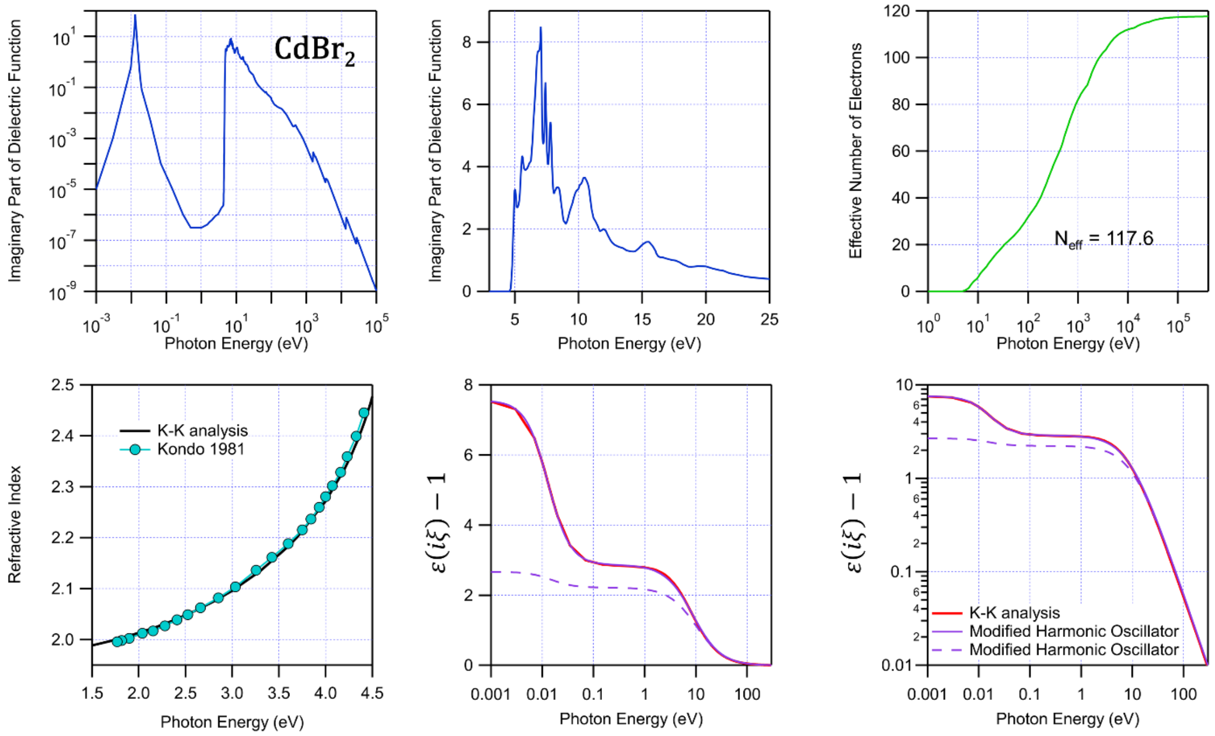


**Fig. S10. The dielectric Function of $CdBr_2$ (perpendicular to C-axis).** Infrared absorption band is reported in ref.[42] We analysed reflectivity spectrum of $CdBr_2$ to calculate the full dielectric function. Reflectivity data between 4 to 9 eV are from the ref.[43] (those which were measured at room temperature), between 9 to 20 eV from ref.[44] (those which were measured at 195K) and between 20 to 30 eV reflectivity data are approximated from the values reported in ref.[45]. For photon energies above 30 eV, reflectivity is computed from the theoretical X-ray refractive indices.[46, 47] The imaginary part of the dielectric function was then computed by K-K analysis of the reflectivity data. Near the fundamental absorption edge, we used data obtained from the analysis of the absorption spectrum reported in ref.[48] at room temperature assuming the refractive index is 2.45[49]. Intensity of the calculated absorption spectrum between 4-100 eV increased by 4% to have an accurate estimation for refractive indices in visible region and to account correctly for effective number of electrons. The calculated effective number of electrons matches the expected value (118) for $CdBr_2$. Also, the refractive indices computed from K-K analysis match the measurements of Kondo[49]. The dielectric function parallel to C-axis (dashed lines) is approximated using the electronic dielectric constant computed from refractive indices reported by Kondo[49]. The strength of infrared band (0.45) is a theoretical estimation reported in Materials Project webpage [50] and the position of the band (15 meV) was an arbitrary choice.

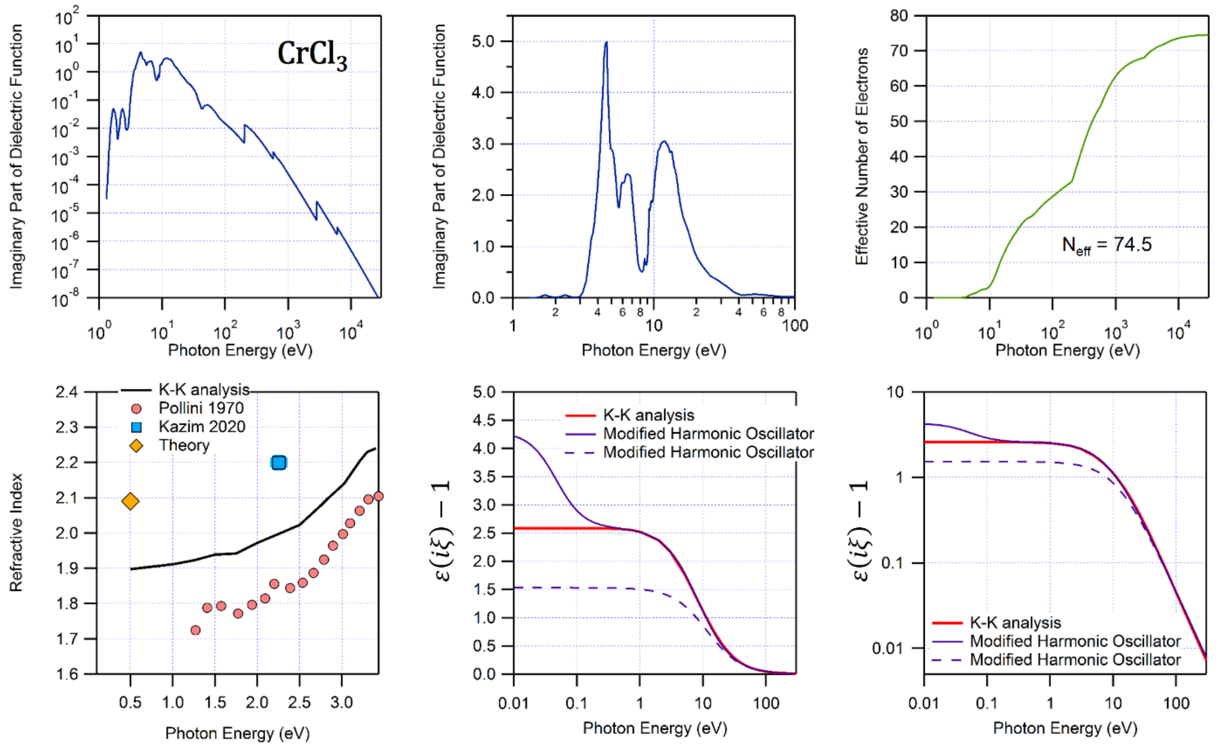


**Fig. S11. The dielectric Function of $CrCl_3$ (perpendicular to C-axis).** Data up to 2.9 eV are estimated using the absorption spectrum reported by Pollini and Spinolo[51]. Between photon energies of 2.9 to 30 eV, the data of Carricabruru *et al*[52] are used. For the rest of the photon energies, the data are estimated based on the atomic scattering factors and the nominal density using the toolbox of the Centre for X-ray Optics' (CXRO) website.[47] The refractive indices of $CrCl_3$ have been rarely reported and the indices calculated using K-K analysis are between the reported values by Kazim[53] and Pollini[51]. The theoretical electronic refractive index is also shown for the sake of comparison.[54] The position of the infrared band (47meV) is approximated from the work of Borghesi *et al*.[55], but the oscillators strength is a theoretical value.[54] The dielectric function parallel to C-axis (dashed line) is approximated using the theoretical electronic dielectric constant (2.53).[54]

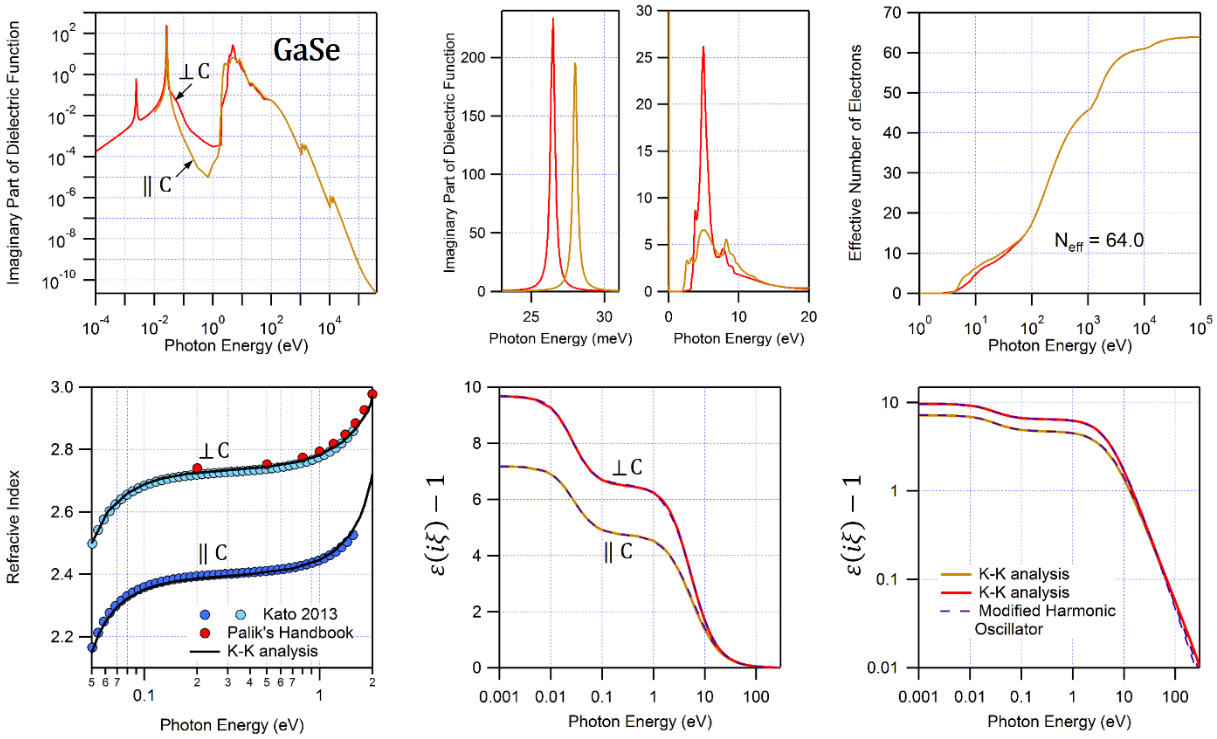


**Fig. S12. The dielectric function of GaSe.** In perpendicular to C-axis, the data are extracted from our recent work[16]. Details and references are given there. In parallel to C-axis, the IR band is calculated based on the data reported by Chen *et al*.[56] Data between 2 and 40 eV are reported by Soukiassian et al.[57] Between 40 to 50 eV, the data are interpolated. Above 50 eV, it is assumed that there is no anisotropy. The computed refractive indices are in excellent agreement with the report by Kato *et al*.[58] and those tabulated in Palik's handbook[59].

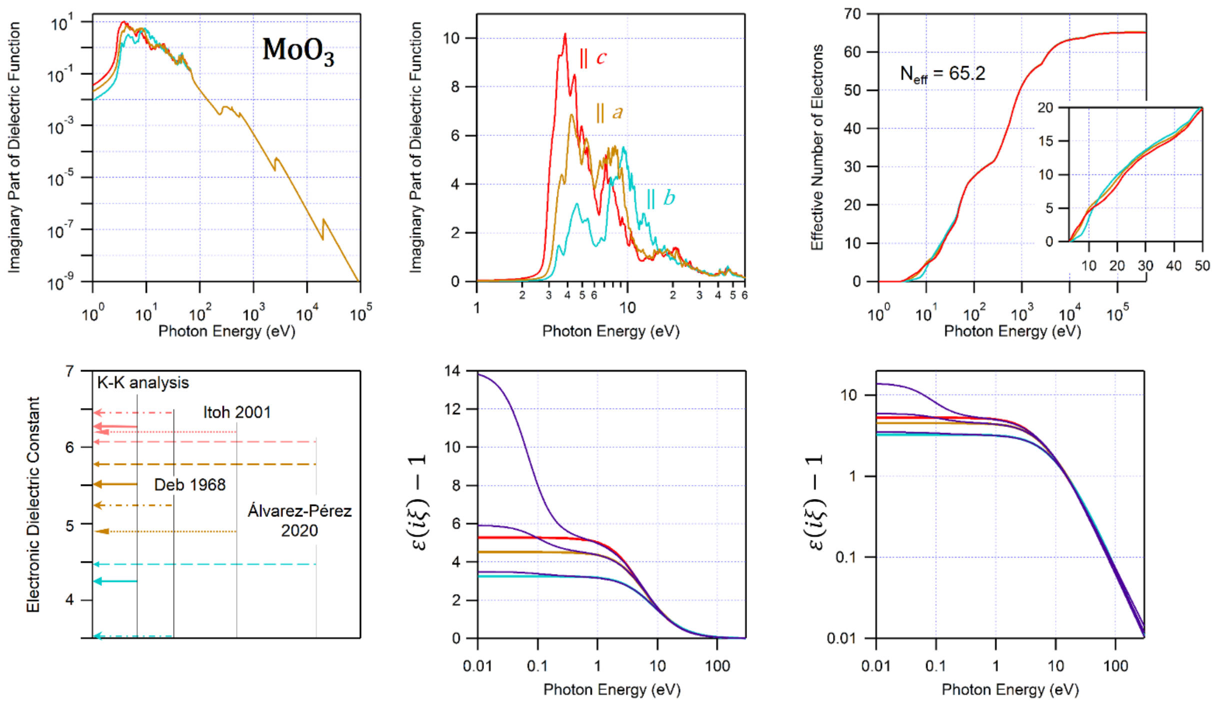


**Fig. S13. The dielectric function of α-MoO3.** The data up to 65 eV is reported by Lajaunie *et al.*[60] Above 65 eV and up to 30 keV, data are estimated from the atomic scattering factors and the nominal density of α-MoO3 using the CXRO toolbox.[47] Above 30 keV, data are from the National Institute of Standards and Technology's (NIST) database [46]. The calculated effective number of electrons in all optical axes are the same and are 65.2. The reported values for electronic dielectric constants of α-MoO3 in different optical axes are not consistent. We have compared the values obtained from the K-K analysis to the proposed values by Itoh[61], Deb[62], Alvarez-Pèrez[63]. The IR bands positions and strengths reported by Alvarez-Pèrez[63] are added to the dielectric functions calculated by K-K analysis.

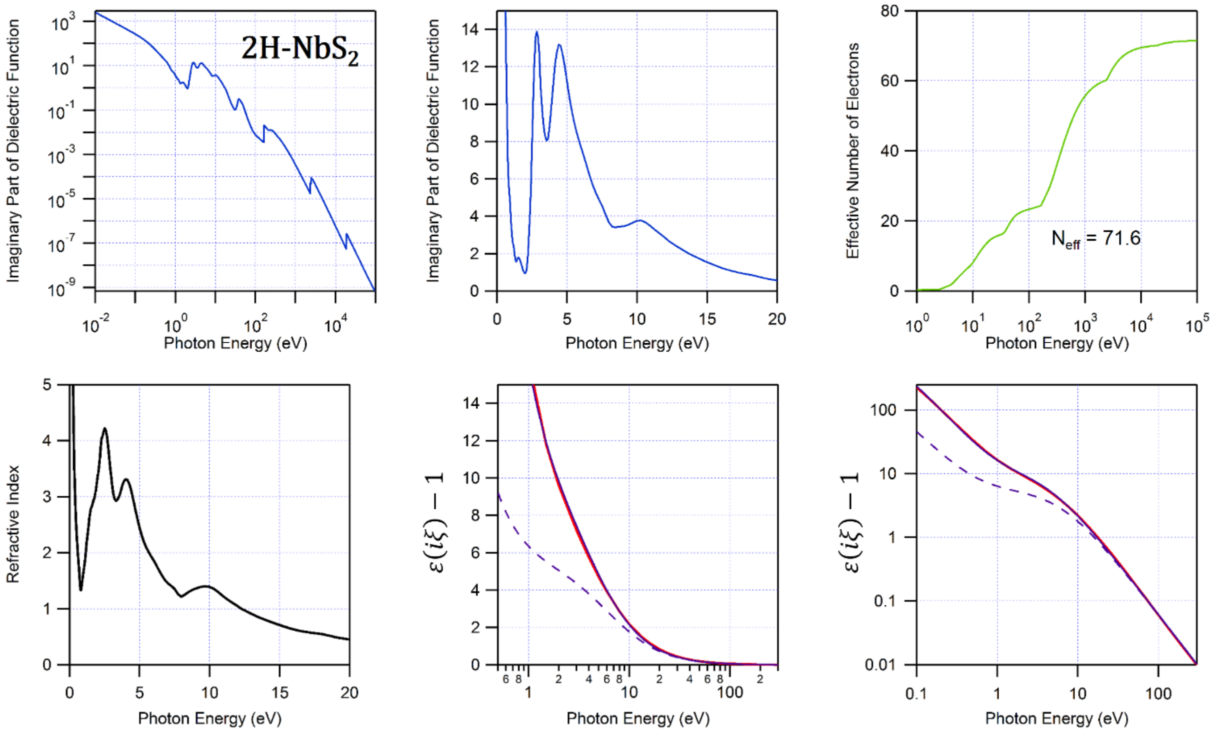


**Fig. S14. The dielectric Function of 2H-NbS$_2$.** In perpendicular to C-axis: Data at low photon energies are estimated using a Drude band with a plasma frequency, $\omega_p$, of 2.75 eV and a scattering rate, $\gamma$, of 0.31 eV. These parameters are obtained from fitting the experimental data of Parkin and Beal.[64] We noted that these parameters lead to a DC conductivity lower than the experimental value, namely 11000 S·cm$^{-1}$ reported by Naito *et al.*[65] This deviation, however, should have a minor effect on the calculated Hamaker constant as the interband transitions are dominant for this metal.[16] Between photon energies of 0.3 to 5 eV, reflectivity data of Naito *et al.*[65] were used. The absolute value of reflectivity is not reported by authors. Therefore, we normalized the reflectivity such that the reflectivity values match with the work of Manzke *et al.* [66] The reflectivity up to 41 eV was computed from the data provided by Manzke *et al.* whereas for photon energies above 60 eV, reflectivity is computed from the theoretical X-ray refractive indices.[46, 47] Between 41 to 60 eV, the reflectivity values are interpolated. The reflectivity spectrum was then analysed by K-K relations to obtain the complex dielectric function. The computed effective number of electrons is 71.6 which slightly lower than the nominal value, *i.e.* 73. We could not find an independent measurement on refractive index of 2H-NbS$_2$ to compare it with the calculated refractive indices. In parallel to C-axis, we used the same Drude parameters as we proposed for NbSe$_2$[16]. The electronic dielectric constant is assumed to 6.45, a crude estimation based on the anisotropy magnitude in NbSe$_2$.

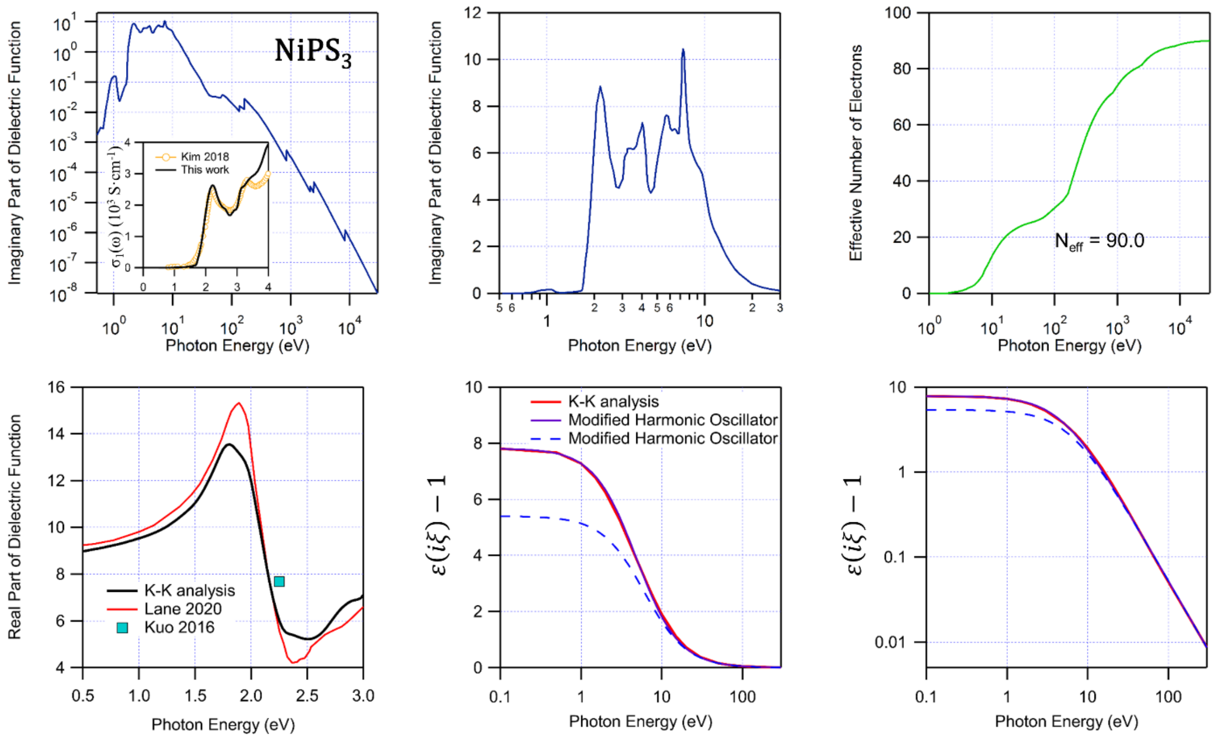


**Fig. S15. The dielectric function of NiPS3.** In perpendicular to C-axis: Data up to 1.6 eV are estimated from the absorption spectrum reported by Foot and Nevett[67], assuming an average refractive index of 3.1 in this range. Between 1.6 to 30 eV, data are from the work by Piacentini *et al.* [68] This data are consistent with the measurements of Kim *et al.*[69] up to 3eV (See inset of the topmost-right panel). Between 3 to 6 eV, the data of these two works do not match. Data above 30 eV are estimation using the atomic scattering factors of the elements and the nominal density.[47] The computed effective number of electrons is 1.1% lower than the nominal value, *i.e.* 91. There is no report on the refractive index of $NiPS_3$ in the visible region. Kuo *et al.* [70] have estimated complex refractive index of $NiPS_3$ based on the optical contrast of its thin layer atop of oxidized silicon. We also compared our K-K analysis with the *ab-initio* calculations by Lane and Zhu[71] for bulk $NiPS_3$. We have ignored the contribution of the IR bands in our calculations of the dielectric function. Recently, Belvin *et al.*[72] reported dielectric constant of 8 for $NiPS_3$ in terahertz region, which consistent with low polarity of this crystal and minor contribution of IR bands to total dielectric constant. Also, we were not able to find any experimental data on optical constants of $NiPS_3$ in parallel to C-axis. Therefore, we used the theoretical value, *i.e.* 6.4, calculated by Lane and Zhu.[71]

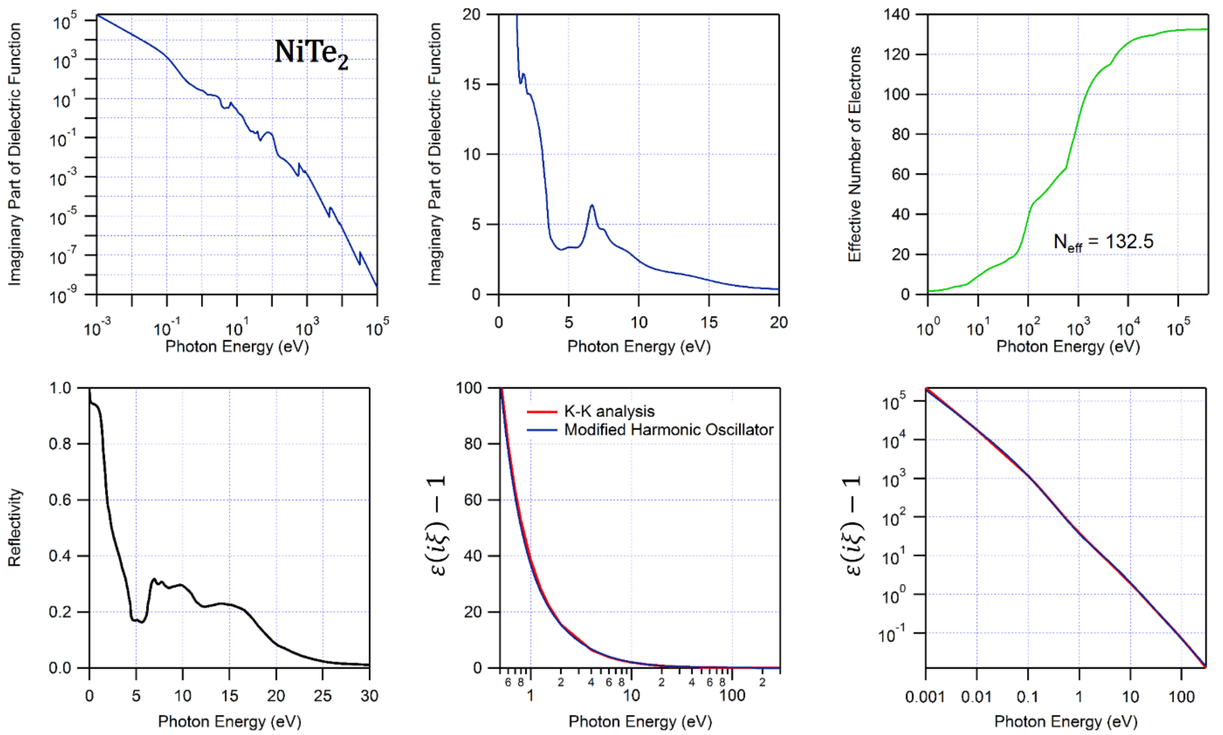


**Fig. S16. The dielectric Function of $NiTe_2$.** In perpendicular to C-axis: At low photon energies, a Drude band with $\omega_p$ = 5.35 eV and $\gamma$ = 0.154 eV. These parameters are estimated from the work of Xu *et al.* [73] It is assumed that the room temperature conductivity is 25000 $S\cdot cm^{-1}$ and the average effective mass of carriers is 0.25 $m_e$ ($m_e$ is mass of electron). The carriers' mobility is assumed to be 30 $cm^2\cdot V^{-1}\cdot s^{-1}$. Mamy *et al.*[74] have reported the reflectivity of the $NiTe_2$ between 3 to 40 eV. However, the reflectivity of $NiTe_2$ is not reported at lower energies. Therefore, we estimated the reflectivity between 0.1 to 3 eV using the data reported for $IrTe_2$.[75] Data at photon energies above 40 eV are estimated using the atomic scattering factors of Ni and Te and density of $NiTe_2$. The reflectivity spectrum was then analysed using K-K relations to obtain the complex dielectric function. The computed effective number of electrons is in good agreement with the nominal value of 132.

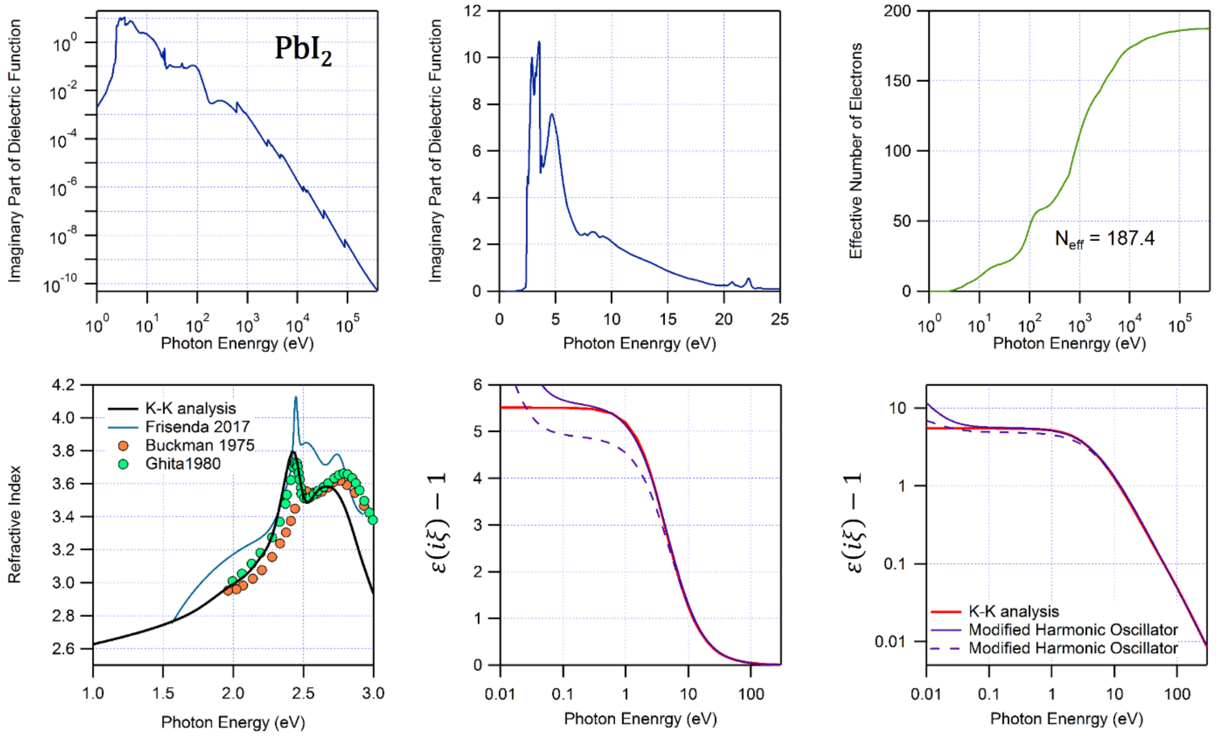


**Fig. S17. The dielectric function of $PbI_2$**. In perpendicular to C-axis: Data up to 5 eV are from the ellipsometry measurements by Ghità *et al.* [76] The magnitude of the $\varepsilon_2$ is increased compared to the original report to result in accurate refractive indices in visible range. Between 5 to 8 eV, data are interpolated. From 8 to 18 eV, data by Abreu[77] are used. Between 18 to 26 eV, absorption coefficients reported by Hayashi *et al.* [78] are used. In order to compute the $\varepsilon_2$, the refractive indices suggested by Abreu[77] are employed. Between 26 to 30 eV, data are interpolated. Above 30eV, refractive indices are computed from the atomic scattering factors and the nominal density of $PbI_2$.[46, 47] The calculated effective number of electrons agrees with nominal value of 188. The computed refractive indices in visible range also match three independent measurements by Frisenda[79], Buckman[80] and Ghità[76]. The IR band position and strength are given in the work of Haas[81]. He also has listed the electronic and static dielectric constant of $PbI_2$ parallel to C-axis, which we used to construct the dielectric function (dashed line) following the protocol explained in our previous work.[16]

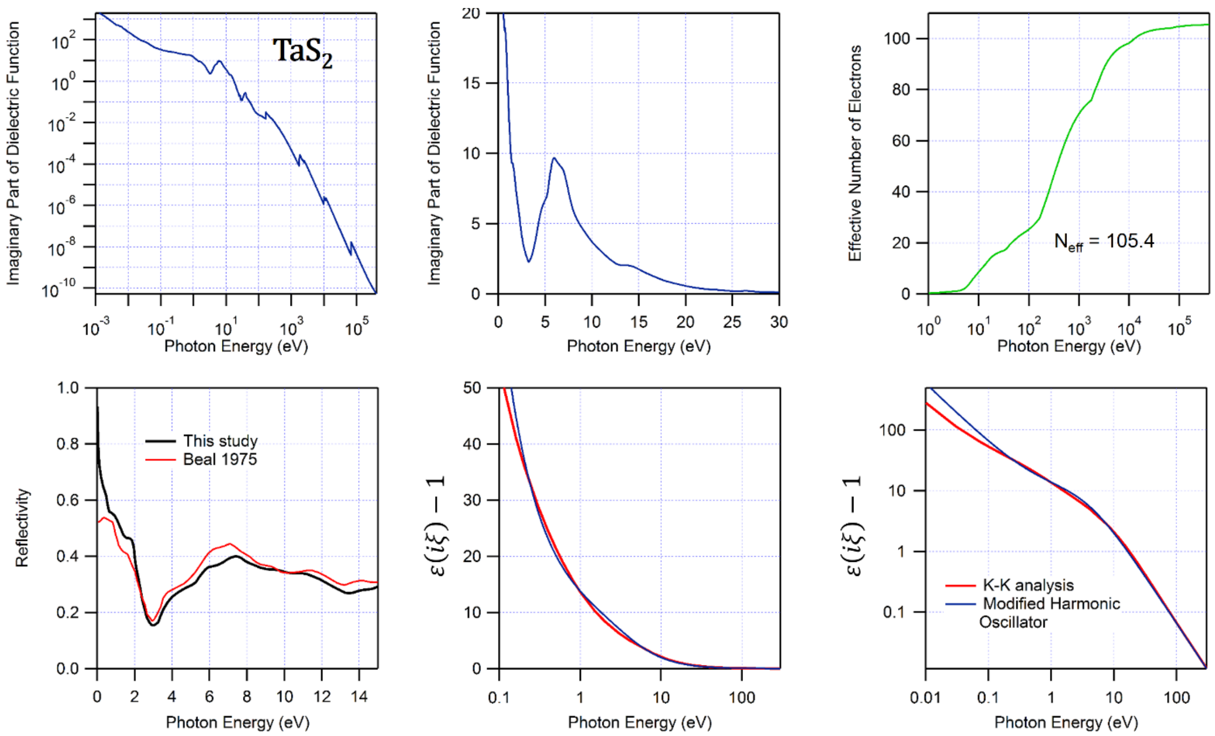


**Fig. S18. The dielectric function of 1T-TaS₂.** In perpendicular to C-axis: Below 2 eV, reflectivity data by Dean *et al*.[82] and Lucovsky *et al*.[83] are used. Mamy *et al*.[74] has reported reflectivity data from 2 to 41 eV. Between 41 to 50 eV, reflectivity is interpolated. Above 50 eV, reflectivity is estimated from the X-ray refractive indices computed from the atomic scattering factors and density.[46, 47] Finally, the reflectivity spectrum was analysed using K-K relations to compute the complex dielectric function. The computed effective number of electrons agrees with the nominal value of 105. Also, the reflectivity measurements by Beal *et al*.[84] shows a good agreement with the compiled data in this study, at least between 2 and 15 eV. We were not able to find any measurements on optical constant of 1T-$TaS_2$ in parallel to C-axis.

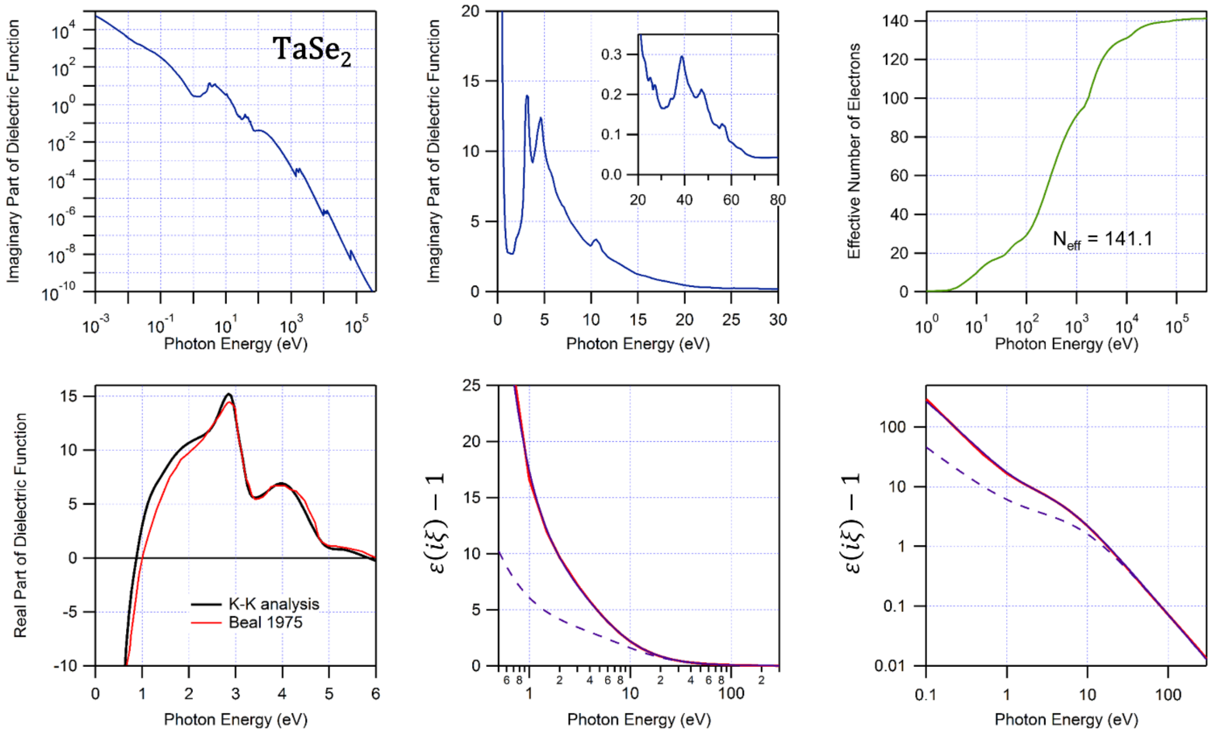


**Fig. S19. The dielectric function of 2H-TaSe$_2$.** In perpendicular to C-axis: Data below 1 eV are obtained from the work of Ruzicka *et al.*[85] Between 1 to 12 eV, data reported by Beal *et al*.[84] are used. Between 12 to 70 eV, the EELS spectrum reported by König *et al.* [86] was K-K analysed. The analysis is done by extrapolating the EELS spectrum to higher energies using the refractive indices obtained from atomic scattering factors. Data between 70 to 80 eV are interpolation. Between 80 to 20000 eV, data are obtained from the CXRO dataset.[47] Above 20 keV, data are from NIST database.[46] The computed effective number of electrons agrees with nominal value of 141. Real part of dielectric function obtained from the K-K analysis of the compiled data agrees with the data of Beal *et al.* [84] The dielectric function in parallel to C-axis (dashed line) is approximated using the data reported by Ruzicka *et al*.[85] following the protocol explained in our previous work.[16]

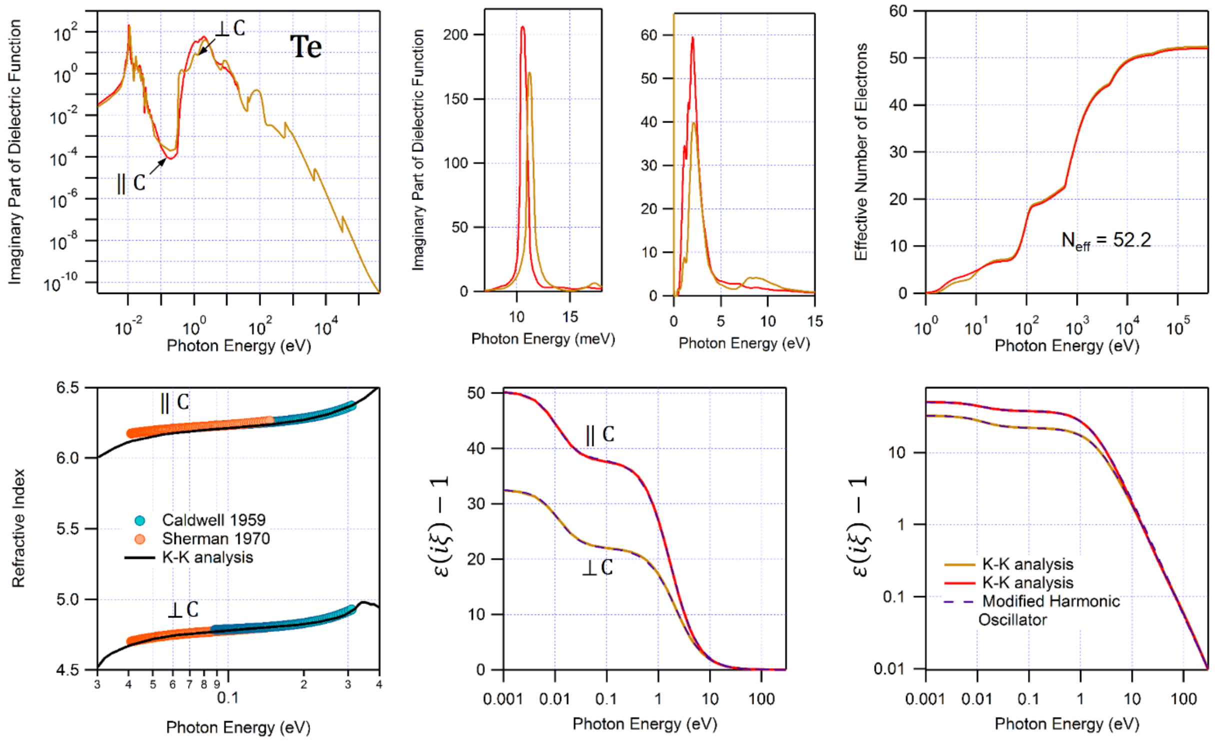


**Fig. S20. The dielectric function of Tellurium.** For both polarization the impact of free-carriers has been ignored due to negligible carrier concentration compare to the total electron concentration.[16] However, Selders *et al.*[87] have measured and discussed this part of the dielectric function of tellurium. *Parallel to C-axis:* up to 0.05 eV, the data are reported by Grosse *et al.*[88] Between photon energies of 0.05 to 0.35 eV, data from Palik's handbook are used.[89] From 0.35 to 5 eV, data measured by Tutihasi *et al.*[90] are used. Between 5 to 20 eV, data from the work of Bammes *et al.*[91] are used. Bammes *et al.* have measured the dielectric function up to 30 eV. However, their data at higher energy tail is not consistent with the measurement of Sonntag *et al.*[92] We therefore interpolated data in energy range of 20 to 30 eV, to be consistent with the data by Sonntag *et al.* (which are consistent with data in CXRO data base). Above 30 eV, data from scattering form factor of tellurium are used.[46, 47] We note that between 200 to 600 eV, data tabulated in CXRO and NIST database are not consistent. We used values in between to obtain more accurate estimation for effective number of electrons. *Perpendicular to C-axis:* up to 0.35 eV, we obtained data from the same references listed above. Between 0.35 to 12 eV, data measured by Tutihasi *et al.*[90] are used. To achieve a more accurate estimation of the refractive indices, the intensity is slightly increased compared to the original work. From 12 to 23 eV, data from the work of Bammes *et al.*[91] are used. Between 23 to 30 eV, data are interpolated. Above 30 eV, data are the same as the other polarization. The computed effective number of electrons for both polarizations are the same, namely 52.2, close to the atomic number of tellurium. The calculated refractive indices in IR region (below the band gap of tellurium) are in very good agreement with the measurements of Caldwell and Fan[93] and Sherman[94, 95]. Two modified harmonic oscillators are enough to fit the dielectric function in imaginary frequencies.

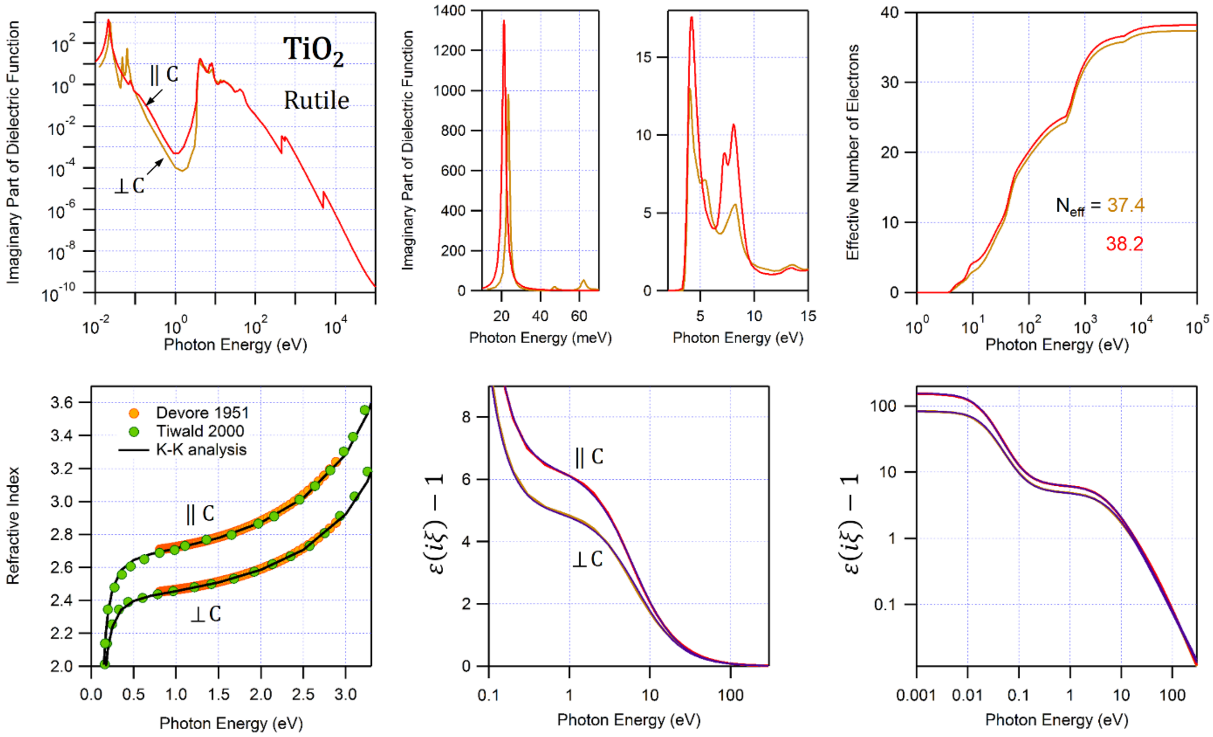


**Fig. S21. The dielectric function of rutile $TiO_2$.** We have included data of rutile $TiO_2$ as a well-known example of non-layered optically anisotropic crystal. The purpose is to provide another example that the optical anisotropy fades at high photon energy (at least in imaginary frequencies). IR bands in both polarizations are measured by Schöche *et al.*[96] Data between 0.1 to 3 eV are interpolation, assuming the rutile is transparent is this range. Between 3 to 4 eV, absorption spectrum reported by Sbai *et al.*[97] are used. Between 4 to 8.2 eV, data reported by Tiwald and Schubert[98] are used. In photon energy range of 8.2 to 25 eV, data by French *et al.*[99] are used. Data from 25 to 30 eV are interpolation. Above 30 eV, atomic scattering factors are used to calculate the complex refractive indices.[46, 47] The calculated effective numbers of electrons in both polarization deviate less than 1.6% from the nominal value of 38. The computed refractive indices from the K-K analysis is in perfect agreement with two independent measurements by Tiwald and Schubert[98] and DeVore[100].

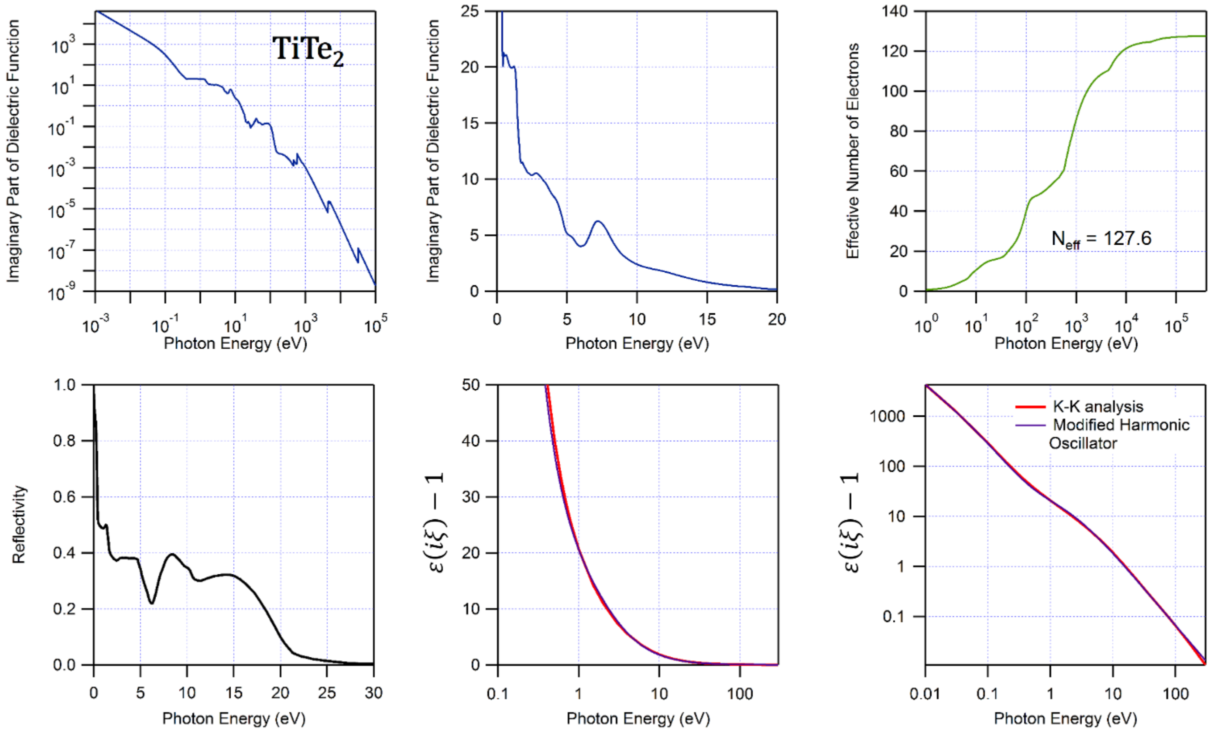


**Fig. S22. The dielectric function of 1T-TiTe₂.** Data at low frequencies are modelled using a Drude band with $\omega_p$ = 2.95 eV and $\gamma$ = 0.198 eV. The $\omega_p$ is given in the work of Allen and Chetty.[101] The scattering rate, $\gamma$, is estimated using room temperature DC conductivity of 5900 S·cm$^{-1}$.[102] Between 0.4 to 1.9 eV, reflectivity data of Greenaway and Nitsche[103] are used. From 1.9 to 41 eV, reflectivity is reported by Mamy *et al.*[74] At higher photon energies, reflectivity is computed using the atomic scattering factors and nominal density.[46, 47] The reflectivity spectrum was then analysed using the K-K relations to calculate the complex dielectric function. The calculated effective number of electrons is 1.3% higher than the nominal value of 126.

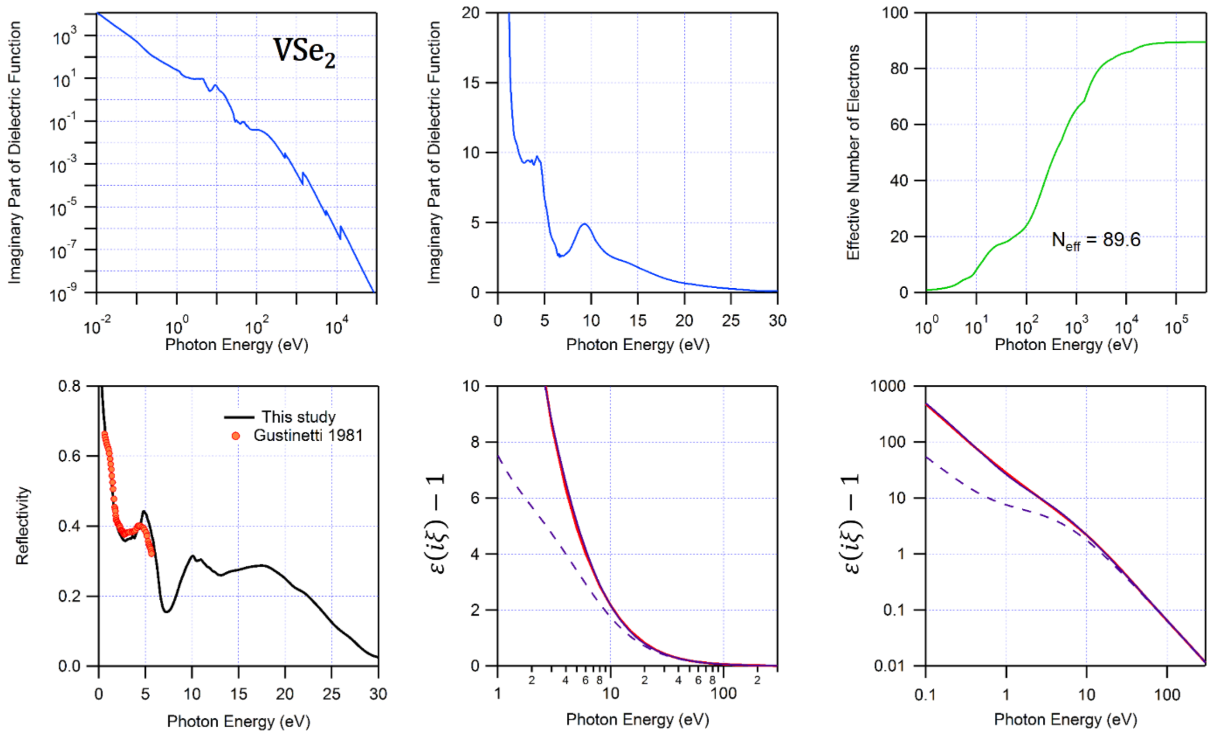


**Fig. S23. The dielectric function of 1T-VSe2**. In perpendicular to C-axis: reflectivity data up to 2.6 eV are reported by Feng *et al*.[104] Reflectivity between 2.6 to 3 eV is interpolation. From 3 to 14 eV, reflectivity data reported by Bayliss and Liang[105] are used. Above 14 eV, we were not able to find any data on optical constant of 1T-VSe2. Therefore, we analysed the reflectivity spectrum by adding an arbitrary band around 17 eV, similar to many transition metal tellurides.[74] It was adjusted such that the effective number of electrons agree with the nominal value. At photon energies above 30 eV, reflectivity is computed using the atomic scattering factors and the density of 1T-VSe2. The reflectivity spectrum was then analysed using K-K relations. The effective number of electrons is 1.5% lower than the nominal value of 91. The reflectivity measured by Gustinetti and Campagnoli[106] shows good agreement with the current work. The dielectric function in parallel to C-axis (dashed line) is a crude approximation using a protocol explained for NbSe2 in our previous work.[16]

**Section 13- List of non-layered materials used in Figure 3 and their corresponding electronic dielectric constant, band gap and Hamaker constant:**

Below we have listed all non-layered materials we analysed and presented in Figure 3. Some of the materials in this list have been analysed in our previous work[16] and we used their full spectrum of the dielectric function to calculate their Hamaker constant. For other materials, we used harmonic oscillator model to estimate the dielectric function and consequently the Hamaker constant.

**Table S1. Optical band gap, electronic dielectric constant and computed Hamaker constant of 73 materials presented in Figure 3.**

| | Materials | Band gap (eV) | Electronic dielectric constant | Hamaker constant (eV) |
|---|---|---|---|---|
| *1* | Acetone | 6.3 | 1.81 | 0.29 |
| *2* | $Al_2O_3$ | 8.5 | 3.08 | 1.09 |
| *3* | AlN | 6 | 4.41 | 1.38 |
| *4* | $BaTiO_3$ | 3.2 | 5.16 | 1.54 |
| *5* | Benzene | 5.5 | 2.18 | 0.39 |
| *6* | BeO | 9.5 | 2.92 | 1.084 |
| *7* | Bromo naphthalene | 4 | 2.60 | 0.541 |
| *8* | Bovine serum albumin | 5.3 | 2.37 | 0.577 |
| *9* | $C_6F_{14}$ | 10 | 1.535 | 0.2 |
| *10* | $C_6H_5Br$ | 4.6 | 2.38 | 0.56 |
| *11* | $C_7H_8O$ | 5.7 | 2.30 | 0.47 |
| *12* | $CaCO_3$ | 7.4 | 2.69 | 0.713 |
| *13* | $CaF_2$ | 11.6 | 2.04 | 0.49 |
| *14* | $CCl_4$ | 6.5 | 2.10 | 0.40 |
| *15* | CdS | 2.42 | 5.18 | 1.107 |
| *16* | $CH_2I_2$ | 3.5 | 2.89 | 0.627 |
| *17* | Chlorobenzene | 5.4 | 2.27 | 0.46 |
| *18* | CsI | 6.2 | 3.04 | 0.802 |
| *19* | Diamond | 5.5 | 5.65 | 0.219 |
| *20* | 1,2-dichloroethane | 6.9 | 2.045 | 0.43 |
| *21* | DMF | 4.8 | 2.00 | 0.33 |
| *22* | DMSO | 5.5 | 2.13 | 0.43 |
| *23* | DNA | 4.2 | 2.43 | 0.61 |
| *24* | Ethanol | 7.5 | 1.83 | 0.33 |
| *25* | Ethyl acetate | 6.9 | 1.87 | 0.33 |
| *26* | ethylene glycol | 7 | 2.02 | 0.43 |
| *27* | $Fe_2O_3$ | 2.2 | 7.08 | 1.391 |
| *28* | Formamide | 6.05 | 2.03 | 0.39 |
| *29* | GaP | 2.24 | 9.23 | 1.753 |
| *30* | Ge | 0.78 | 16.10 | 1.897 |
| *31* | Glycerol | 8.2 | 2.14 | 0.52 |
| *32* | hexadecane | 6.5 | 2.02 | 0.39 |
| *33* | hexafluoro benzene | 6 | 1.86 | 0.34 |
| *34* | Iodobenzene | 5.7 | 2.52 | 0.53 |

| | | | | |
|---|---|---|---|---|
| ***35*** | Isopropyle alcohol | 7.5 | 1.86 | 0.33 |
| ***36*** | KCl | 7.35 | 2.18 | 0.45 |
| ***37*** | $LaF_3$ | 10.5 | 2.55 | 0.899 |
| ***38*** | LiF | 11.9 | 1.93 | 0.50 |
| ***39*** | Liquid nitrogen | 11.9 | 1.44 | 0.15 |
| ***40*** | Liquid oxygen | 12 | 1.488 | 0.19 |
| ***41*** | methanol | 7.5 | 1.75 | 0.29 |
| ***42*** | methyl naphthalene | 4 | 2.50 | 0.49 |
| ***43*** | $MgF_2$ | 11.3 | 1.96 | 0.52 |
| ***44*** | MgO | 7.5 | 2.95 | 0.966 |
| ***45*** | montmorillonite | 7.85 | 2.03 | 0.43 |
| ***46*** | n-octane | 7.35 | 1.93 | 0.34 |
| ***47*** | NaCl | 7.3 | 2.36 | 0.51 |
| ***48*** | nitro methane | 5.4 | 1.86 | 0.28 |
| ***49*** | nitrobenzene | 4.2 | 2.32 | 0.48 |
| ***50*** | NMP | 5.15 | 2.10 | 0.39 |
| ***51*** | PbS | 0.42 | 17.00 | 1.751 |
| ***52*** | Polycarbonate | 4.4 | 2.39 | 0.53 |
| ***53*** | PDMS | 5.75 | 1.96 | 0.33 |
| ***54*** | Polyethylene | 6.5 | 2.21 | 0.47 |
| ***55*** | polyisoprene | 5.65 | 2.22 | 0.46 |
| ***56*** | Polystyrene | 5 | 2.45 | 0.53 |
| ***57*** | PVC | 4.6 | 2.33 | 0.46 |
| ***58*** | Pyridine | 4.4 | 2.21 | 0.44 |
| ***59*** | Silicon | 1.14 | 11.70 | 1.695 |
| ***60*** | $Si_3N_4$ | 5.2 | 3.94 | 1.282 |
| ***61*** | SiC (6H) | 3.05 | 6.55 | 1.647 |
| ***62*** | $SiO_2$-quartz | 9.2 | 2.36 | 0.647 |
| ***63*** | $SiO_2$-silica | 8.8 | 2.11 | 0.49 |
| ***64*** | $SrTiO_3$ | 3.2 | 5.15 | 1.352 |
| ***65*** | $Ta_2O_5$ | 4.5 | 4.40 | 1.404 |
| ***66*** | $TiO_2$-anatase | 3.6 | 5.84 | 1.448 |
| ***67*** | $TiO_2$-rutile | 3.1 | 7.24 | 1.722 |
| ***68*** | Toluene | 5.7 | 2.18 | 0.41 |
| ***69*** | water | 7.2 | 1.76 | 0.31 |
| ***70*** | $Y_2O_3$ | 5.75 | 3.60 | 1.207 |
| ***71*** | ZnO | 3.37 | 3.80 | 0.893 |
| ***72*** | ZnS-Cubic | 3.54 | 5.08 | 1.352 |
| ***73*** | $ZrO_2$ | 5 | 4.45 | 1.613 |

**Section 14- Experimental data on binding energy of graphite and mica:**
**Table S2. Experimental binding energy of graphite and mica**

| | Material | Binding energy (meV·Å$^{-2}$) | Measurement method and reference | Note |
|---|---|---|---|---|
| *1* | ***Graphite*** *(multiwalled carbon nanotubes)* | 12 | AFM (from sliding forces)[107] | |
| *2* | ***Graphite*** ***(****double walled carbon nanotubes)* | 13.61 | Force measurement using AFM[108] | |
| *3* | ***Graphite*** *(carbon nanotubes)* | 13.1 | Direct observation of layers' mechanics under TEM[109] | |
| *4* | ***Graphite*** | 16.2 | Heat of wetting[110] | |
| *5* | ***Graphite*** | 13.36±1.37 | Contact angle measurements[3] | Average of different experimental values in page 110 is reported here. |
| *6* | ***Graphite*** | 15.6 | Contact angle measurements[111] | |
| *7* | ***Graphite*** | 15.0±1.9 | Mechanical cleavage[112] | Data are analysis by Abrahamson using two different elastic constants for graphite. The surface energies derived from the higher shear stiffness modulus are taken, as this value is closer to both theoretical and experimental values.[113] |
| *8* | ***Graphite*** | 23.28±1.93 | Desorption energy of aromatic molecules[114] | |
| *9* | ***Graphite*** | 18.1 | Thermal desorption of $C_{60}$ molecules[115] | The binding energy is approximated based on Figure 2 in this report. |
| *10* | ***Graphite*** | 11.9±0.6 | AFM friction [116] | |
| *11* | ***Graphite*** | 13.23±3.75 | AFM friction[117] | |
| *12* | ***Graphite*** | 14.17±3.12 | AFM friction [118] | |
| *13* | ***Graphite*** | 23.1±0.6 | From shear force-displacement measurements[119] | |
| *14* | ***Graphite*** | 21.2±4.4<br>13.1±2.5 | Mechanical peeling of large graphite[120] | |

| | | | | |
|---|---|---|---|---|
| *15* | ***Graphite (graphene atop of graphite)*** | 13.79±0.69 | Analysis of atomic scale blisters [121] | |
| *16* | ***Few layered graphene*** | 14.85±3.75 | From adhesion forces measured by surface force apparatus[122] | |
| *17* | ***graphene*** | 19.16±2.50 | Adhesion forces from AFM[123] | |
| *18* | ***Graphite and multiwalled carbon nanotubes*** | 12.5±5.6<br>22.5±10 | AFM measurement in a SEM chamber[124] | Two values are reported due to the uncertainty about CNTs configuration |
| *19* | ***Muliwalled carbon nanotubes*** | 14.11 | Inverse gas chromatography[125] | |
| *20* | ***Graphite*** | 13.48 | Inverse gas chromatography[126] | |
| *21* | ***Muliwalled carbon nanotubes*** | 15.35 | Inverse gas chromatography[127] | |
| *22* | ***Graphite*** | 15.29 | Inverse gas chromatography[128] | |
| *23* | ***Graphite*** | 16.79 | Temperature programmed desorption spectroscopy[129] | |
| *24* | ***Mica*** | 18.1±3.7 | From adhesion force measurements using a surface force apparatus[130] | Average value of different measurements reported in table 1 of this reference is reported |
| *25* | ***Mica*** | 15.0 | Mechanical cleavage[131] | Reported in table 1 of entry 24 |
| *26* | ***Mica*** | 15.0 | Contact angles[132] | Reported in table 1 of entry 24 |
| *27* | ***Mica*** | 9.8±1.0 | Direct force measurements[133] | Reported in table 1 of entry 24 |
| *28* | ***Mica*** | 13.1±4.4 | Mechanical cleavage[134] | Reported in table 1 of entry 24 |
| *29* | ***Mica*** | 21.8±2.2 | Mechanical cleavage[135] | Reported in table 1 of entry 24 |

**Section 15- List of materials used for analysis of the vdW cut-off distance:**
Below, we have listed all 52 materials that have been analysed to compute the empirical vdW cut-off distances presented in Figure S3 and 1C. For each material, the dielectric function at imaginary frequencies or the reference to experimental dielectric function are given. From these functions we calculated the non-retarded Hamaker constant. The experimental value for both total and dispersion surface energy (half of binding energy) are given whenever it was possible. The vdW cut-off distances were then computed from the Eq. 3.

**Table S3. List of the materials along with their dielectric function, Hamaker constant and surface energy used in the analysis.**

| | *Material* | *Dielectric function* | *Hamaker constant (eV)* | *Surface energy (meV·Å⁻²)* Total | Dispersive | *Notes and references* |
|---|---|---|---|---|---|---|
| **1** | $CH_2I_2$ | $1+\frac{1.89}{1+(\frac{\xi}{8.83})^{1.6}}+\frac{0.22}{1+(\frac{\xi}{0.1})^{1.5}}$ | 0.627 | 3.17 | 3.03 | *$E_g$ = 3.45 eV [136], $C_{UV}$ = 1.89 [137]. IR band is a rough estimation based on the IR refractive indices.[137]* |
| **2** | *Bromo naphthalene* | $1+\frac{1.6}{1+(\frac{\xi}{9.85})^{1.71}}+\frac{0.15}{1+(\frac{\xi}{0.1})^{1.5}}$ | 0.54 | 2.78 [138] | 2.73 | *$E_g$ = 4 eV [136] (approaximated based on data for chloronaphthalene). $C_{UV}$ = 1.6 [139]. IR band is a crude estimation.[139]* |
| **3** | *Methyl naphthalene* | $1+\frac{1.5}{1+(\frac{\xi}{9.85})^{1.75}}+\frac{0.17}{1+(\frac{\xi}{0.1})^{1.5}}$ | 0.49 | 2.48[139] | 2.48 | *$E_g$ = 4 eV [136] (approaximated based on data for chloronaphthalene). $C_{UV}$ = 1.5 [139]. IR band is a crude estimation.[139]* |
| **4** | *tert butyl naphthalene* | $1+\frac{1.388}{1+(\frac{\xi}{10.03})^{1.75}}+\frac{0.14}{1+(\frac{\xi}{0.1})^{1.5}}$ | 0.4 | 2.10 | 2.10 | *$E_g$ = 4.1 eV [140] (an estimation based on data for naphthalene). $C_{UV}$ = 1.388 [139]. IR band is a crude estimation.[139]* |
| **5** | *Parafin* | $1+\frac{1.141}{1+(\frac{\xi}{14.08})^{1.79}}+\frac{0.03}{1+(\frac{\xi}{0.18})^{1.35}}$ | 0.46 | 2.02 | 2.02 | *$C_{UV}$ = 1.141 [139]. The rest of parameters are the same as polyethylene.* |
| **6** | *Benzyl alcohol* | $1+\frac{1.3}{1+(\frac{\xi}{11.6})^{1.8}}$ | 0.5 | 2.4 | 1.89 | *$C_{UV}$=1.3 estimated from tabulated refractive indices.[141] $\omega_{UV}$ is the average of the experimental one for benzene and chlorobenzene. IR bands are omitted.* |
| **7** | *Polypropylene* | $1+\frac{1.145}{1+(\frac{\xi}{13.1})^{1.79}}+\frac{0.08}{1+(\frac{\xi}{0.18})^{1.35}}$ | 0.4 | 1.88 | 1.88 | *$E_g$ = 5.9 eV [142] $C_{UV}$=1.145 estimated from tabulated refractive indices. IR* |

| | | | | | | |
|---|---|---|---|---|---|---|
| | | | | | | *band is approximated from far-IR refractive index [143] and data for polyethylene.* |
| ***8*** | *polyisoprene* | $1+\frac{1.22}{1+(\frac{\xi}{12.7})^{1.85}}$ | 0.46 | 2.0 | 2.0 | *$E_g$ = 5.65 eV [144], $C_{UV}$ = 1.22 assuming that $n_{IR}$ = 1.49. IR band is omitted* |
| ***9*** | *Styrene-butadiene rubber* | $1+\frac{1.3}{1+(\frac{\xi}{12.45})^{1.8}}$ | 0.50 | 2.1 | 2.1 | *$C_{UV}$ = 1.3 assuming that $n_{IR}$ = 1.515. $E_g$ = 5.5 eV, a crude estimation based on the band gap of PS and polyolefins.* |
| ***10*** | *polyethylene terephthalate* | $1+\frac{1.39}{1+(\frac{\xi}{10.21})^{1.7}}+\frac{0.184}{1+(\frac{\xi}{0.14})^{1.86}}$ | 0.46 | 2.6 | 2.33 | *$E_g$ = 4.2 eV [145], $n_{IR}$ and IR band is estimated from the work of Zhang et al.[146]* |
| ***11*** | *Nylon 6,6* | $1+\frac{1.28}{1+(\frac{\xi}{12.45})^{1.76}}$ | 0.50 | 2.9 | 2.21 | *$E_g$ = 5.5 eV [147], $C_{UV}$ = 1.28 assuming that $n_{IR}$ = 1.51. IR band is omitted* |
| ***12*** | *PMMA* | $1+\frac{1.196}{1+(\frac{\xi}{13.75})^{1.76}}+\frac{0.1}{1+(\frac{\xi}{0.141})^{2}}$ | 0.49 | 2.7 | 2.4 | *Fit to experimental data.[148]* |
| ***13*** | *PVC* | $1+\frac{1.33}{1+(\frac{\xi}{10.91})^{1.71}}+\frac{0.056}{1+(\frac{\xi}{0.112})^{1.69}}$ | 0.46 | 2.57 | 2.27 | *$E_g$ = 4.6 eV [149], $n_{IR}$ and IR band is estimated from the work of Zhang et al.[146]* |
| ***14*** | *bromobenzene* | $1+\frac{1.38}{1+(\frac{\xi}{12.5})^{1.75}}+\frac{0.17}{1+(\frac{\xi}{0.022})^{1.4}}$ | 0.55 | 2.28 | 2.28 | *$C_{UV}$ is slightly different that previous estimation, assuming $n_{IR}$=1.54.[150]* |
| ***15*** | *Xylene* | $1+\frac{1.22}{1+(\frac{\xi}{12.4})^{1.75}}$ | 0.5 | 1.88 | 1.88 | *The parameters for dielectric function are given in our previous work.[16]* |
| ***16*** | *Rutile $TiO_2$* | $1+\frac{6.36}{1+(\frac{\xi}{6.4})^{1.59}}+\frac{148.1}{1+(\frac{\xi}{0.0205})^{1.95}}$ $1+\frac{4.95}{1+(\frac{\xi}{7.1})^{1.59}}+\frac{78.3}{1+(\frac{\xi}{0.0239})^{1.93}}$ | 1.651 | | 8.93 | *Fit to experimental data.[148]* |
| ***17*** | *Iodobenzene* | $1+\frac{1.52}{1+(\frac{\xi}{10.21})^{1.685}}+\frac{0.07}{1+(\frac{\xi}{0.12})^{1.6}}$ | 0.53 | 2.48 | 2.48 | *$E_g$ = 5.7 eV [136], $n_{IR}$ and IR band is estimated from the work of Keefe et al.[151]* |
| ***18*** | *Nitrobenzene* | $1+\frac{1.315}{1+(\frac{\xi}{11.4})^{1.71}}+\frac{0.75}{1+(\frac{\xi}{0.0044})}$ | 0.48 | 2.74 | 2.32 | *Fit to experimental data.[148] Debye relation term is ignored.* |
| ***19*** | *Carbon tetrabromide* | $1+\frac{1.51}{1+(\frac{\xi}{13.6})^{1.67}}$ | 0.691 | 3.10 | 3.10 | *$E_g$ = 6.1 eV [136], $C_{UV}$ = 1.51 assuming that $n_{IR}$ = 1.585.* |

| | | | | | | |
|---|---|---|---|---|---|---|
| **20** | *$Fe_2O_3$* | $1+\frac{6.07}{1+(\frac{\xi}{5.08})^{1.55}}+\frac{16.7}{1+(\frac{\xi}{0.042})^{1.87}}$ $1+\frac{4.9}{1+(\frac{\xi}{5.83})^{1.55}}+\frac{12.5}{1+(\frac{\xi}{0.04})^{1.78}}$ | 1.39 | | 6.68 *[152]* | *Based on the data reported by Querry.[153]* |
| **21** | *Liquid mercury* | $1+\frac{0.79}{1+(\frac{\xi}{25.5})^{1.57}}+\frac{13^2}{\xi^2+2.06\xi}$ | 2.300 | 30.3 | 12.5 *[152]* | *Fit to experimental data.[148]* |
| **22** | *Liquid gallium* | $1+\frac{0.16}{1+(\frac{\xi}{47})^{1.44}}+\frac{15.4^2}{\xi^2+1.01\xi}$ | 2.32 | 44.8 | 15.0 *[154]* | *Fit to experimental data.[148]* |
| **23** | *Polyvinyl alcohol* | $1+\frac{1.149}{1+(\frac{\xi}{13.6})^{1.76}}$ | 0.47 | 3.18 | 2.41 | *$E_g$ = 6.2 eV [155], $C_{UV}$ = 1.149 from dispersion formula.[95, 156]* |
| **24** | *Polyacetylene* | $1+\frac{3}{1+(\frac{\xi}{4.8})^{1.64}}$ | 0.61 | 3.2 | 3.08 *[157]* | *$E_g$ = 1.5 eV and $C_{UV}$ = 3 based on data for trans PAc.[158] IR and Drude bands are ignored as they have minor impact at least for undoped samples.[159]* |
| **25** | *Polyxylyene (parylene)* | $1+\frac{1.56}{1+(\frac{\xi}{10.6})^{1.73}}+\frac{0.44}{1+(\frac{\xi}{0.15})^{1.5}}$ | 0.56 | 2.9 | 2.85 *[160]* | *$E_g$ = 4.4 eV [161]. $C_{UV}$ = 1.56 assuming that $n_{IR}$ = 1.6. IR band is approximated based the data of Li et al.[162] and assuming a dielectric constant of 3.* |
| **26** | *Polyvinyl pyrrolidone* | $1+\frac{1.3}{1+(\frac{\xi}{12.3})^{1.75}}$ | 0.50 | 3.03 | 2.71 | *$E_g$ = 5.4 eV[163]. $C_{UV}$ = 1.3.[164] IR bands are ignored.* |
| **27** | *Poly(p-phenylene oxide)* | $1+\frac{1.45}{1+(\frac{\xi}{10.1})^{1.74}}$ | 0.48 | 2.9 | 2.2 | *$E_g$ = 4.15 eV[165]. $C_{UV}$ = 1.45 from the reported refractive indices. IR bands are ignored.* |
| **28** | *$CaCO3$ , calcite* | $1+\frac{1.69}{1+(\frac{\xi}{13.31})^{1.69}}$ $1+\frac{1.18}{1+(\frac{\xi}{16.46})^{1.69}}$ | 0.690 | | ≈ 3.1±0.3 *[166]* | *The parameters for dielectric functions are given in previous work. IR bands are ignored.* |
| **29** | *Zinc oxide* | $1+\frac{2.8}{1+(\frac{\xi}{7.46})^{1.53}}+\frac{4.35}{1+(\frac{\xi}{0.051})^{2}}$ | 0.908 | | 5.2 *[167]* | *$E_g$ = 3.37 eV[168]. Optical anisotropy of ZnO is minor, thus average $n_{IR}$ = 1.95 was used.[169] IR band is reported by Collins et al.[170]* |

| | | | | | | |
|---|---|---|---|---|---|---|
| **30** | *Magnesium oxide* | $1+\frac{1.95}{1+(\frac{\xi}{13.83})^{1.68}}+\frac{6.7}{1+(\frac{\xi}{0.053})^{2}}$ | 1.00 | | 5.8[167] | *$E_g$ = 7.8 eV [171]. IR band and $C_{UV}$ are estimated from the work of Hanna.[172]* |
| **31** | *Clay minerals* | $1+\frac{1.034}{1+(\frac{\xi}{13.9})^{1.66}}+\frac{2.6}{1+(\frac{\xi}{0.1})^{1.7}}$ | 0.43 | | 2.29 [173] | *$C_{UV}$ and IR band is estimated from the optical constants of Montmorillonite reported by Querry.[174] The rest of parameters for UV band is same as the one for mica.* |
| **32** | *Quartz, $SiO_2$* | $1+\frac{1.37}{1+(\frac{\xi}{14.9})^{1.69}}+\frac{2.3}{1+(\frac{\xi}{0.1})^{1.7}}$ | 0.67 | | 3.1 [173] | *Average refractive index of two axes of quartz was used to calculate $C_{UV}$.[175] $E_g$ = 8.6 eV.[175] IR band is an approximation.[176]* |
| **33** | *Nickel oxide (NiO)* | $1+\frac{4.55}{1+(\frac{\xi}{8.47})^{1.53}}+\frac{6.2}{1+(\frac{\xi}{0.05})^{2}}$ | 1.79 | | 10 [177] | *Fit to experimental data.[148]* |
| **34** | *Calcium fluoride, $CaF_2$* | $1+\frac{1.039}{1+(\frac{\xi}{18.5})^{1.68}}+\frac{4.72}{1+(\frac{\xi}{0.032})^{2}}$ | 0.49 | | 2.49[178] | *Electronic dielectric constant is given in ref.[179]. $E_g$ = 11.6 eV.[180]. IR band is an approximated.[181]* |
| **35** | *Solid Argon (T=0)* | $1+\frac{0.673}{1+(\frac{\xi}{17.4})^{1.82}}$ | 0.245 | 1.42 | 1.42 | *Dielectric constant is calculated from Clausius–Mossotti relation and using the dielectric constant and density at triple point[182] and at T=0[183]. Characteristic frequency and power exponent are estimated by analysing the absorption cross section.[184] Hamaker constant is calculated using eq. 4.15 in ref.[14]* |
| **36** | *Solid Krypton (T=0)* | $1+\frac{0.904}{1+(\frac{\xi}{15.7})^{1.73}}$ | 0.357 | 1.812 | 1.812 | *Same protocol as solid Argon.* |
| **37** | *Solid Xenon (T=0)* | $1+\frac{1.198}{1+(\frac{\xi}{11.9})^{1.63}}$ | 0.428 | 2.18 | 2.18 | *Same protocol as solid Argon.* |
| **38** | *Polystyrene* | *Experimental values[16]* | 0.54 | 2.6 | 2.6 | |
| **39** | *Anatase $TiO_2$* | *Experimental values[16]* | 1.47 | | 7.240 [152] | |
| **40** | *Polyethylene* | *Experimental values[16]* | 0.47 | 2.2 | 2.2 | |
| **41** | *Polycarbonate* | *Experimental values[16]* | 0.53 | 2.9 | 2.67 | |

| | | | | | | |
|---|---|---|---|---|---|---|
| ***42*** | *Polyimide, Kapton* | *Experimental values*[16] | 0.70 | 3.3 | 3.1 | |
| ***43*** | *Silica* | *Experimental values*[16] | 0.5 | | 2.1 | |
| ***44*** | *Chlorobenzene* | *Experimental values*[16] | 0.46 | 2.1 | 2.1 | |
| ***45*** | *Glycerol* | *Experimental values*[16] | 0.5 | 4.0 | 2.3 | |
| ***46*** | *Alumina* | *Experimental values*[16] | 1.105 | | 5.45 ±0.81 *[185]* | |
| ***47*** | *Mica* | *Experimental values*[16] | 0.68 | 7.74± 2.1 | 3.12 ±0.22 *[173]* | *List of the experimental total surface energy of mica is given in Table S2.* |
| ***48*** | *Pyridine* | *Experimental values*[16] | 0.44 | 2.37 | 2.32 *[186]* | |
| ***49*** | *Graphite* | *Experimental values*[16] | 1.495 | | 7.74± 0.50 | *List of the experimental surface energy of graphitic materials is given in Table S2.* |
| ***50*** | *NaCl* | *Experimental values*[16] | 0.51 | | 2.50[178] | |
| ***51*** | DNA | *Experimental values*[16] | 0.61 | 3.43±0.89 | 2.59± 0.31 | The reported surface tension is the average values for microbial cells reported by Sharma *et al.*[187] |
| ***52*** | Bovine serum albumin | *Experimental values*[16] | 0.58 | | | |

**Section 16- List of 107 layered crystals analysed in this study:**

We have compiled the full list of the crystals studied here in an alphabetic order. For each crystal detailed information along with the source of data are presented in the format shown below.

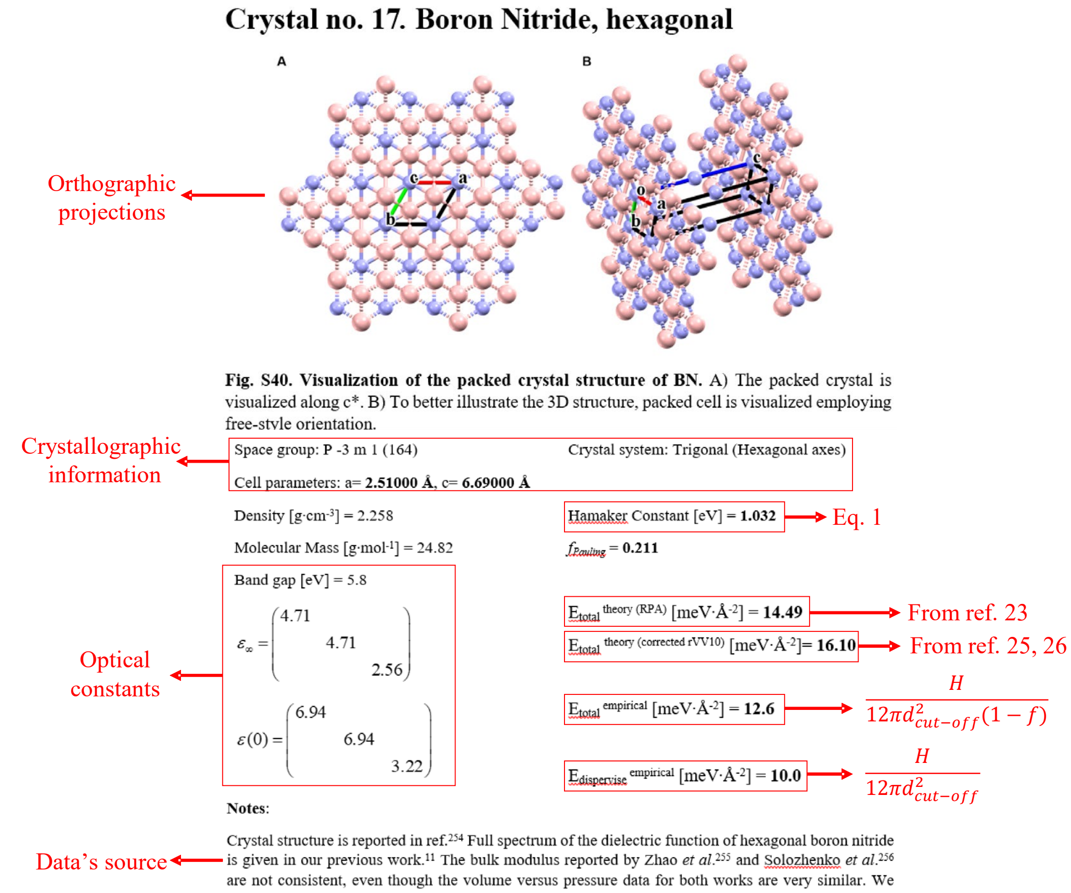


**Crystal no. 17. Boron Nitride, hexagonal**

**Fig. S40. Visualization of the packed crystal structure of BN.** A) The packed crystal is visualized along c*. B) To better illustrate the 3D structure, packed cell is visualized employing free-stvle orientation.

Space group: P -3 m 1 (164) Crystal system: Trigonal (Hexagonal axes)

Cell parameters: a= **2.51000 Å**, c= **6.69000 Å**

Density [g·cm$^{-3}$] = 2.258

Molecular Mass [g·mol$^{-1}$] = 24.82

Hamaker Constant [eV] = **1.032**

$f_{Pauling}$ = **0.211**

Band gap [eV] = 5.8

$$\varepsilon_\infty = \begin{pmatrix} 4.71 & & \\ & 4.71 & \\ & & 2.56 \end{pmatrix}$$

$$\varepsilon(0) = \begin{pmatrix} 6.94 & & \\ & 6.94 & \\ & & 3.22 \end{pmatrix}$$

$E_{total}$ $^{theory\ (RPA)}$ [meV·Å$^{-2}$] = **14.49**

$E_{total}$ $^{theory\ (corrected\ rVV10)}$ [meV·Å$^{-2}$]= **16.10**

$E_{total}$ $^{empirical}$ [meV·Å$^{-2}$] = **12.6** $\rightarrow \dfrac{H}{12\pi d^2_{cut-off}(1-f)}$

$E_{dispersive}$ $^{empirical}$ [meV·Å$^{-2}$] = **10.0** $\rightarrow \dfrac{H}{12\pi d^2_{cut-off}}$

**Notes:**

Crystal structure is reported in ref.[254] Full spectrum of the dielectric function of hexagonal boron nitride is given in our previous work.[11] The bulk modulus reported by Zhao *et al.*[255] and Solozhenko *et al.*[256] are not consistent, even though the volume versus pressure data for both works are very similar. We

# Crystal no. 1. $As_2Te_3$

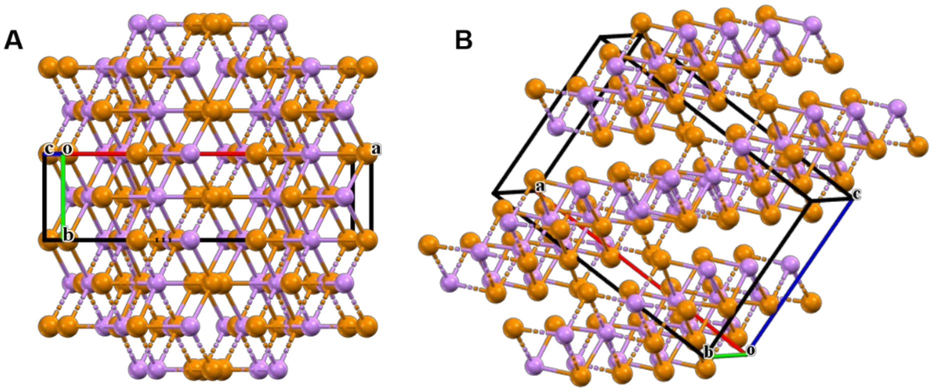


**Fig. S24. Visualization of the packed crystal structure of $As_2Te_3$.** A) The packed crystal is visualized along c*. B) To better illustrate the 3D structure, packed cell is visualized employing free-style orientation.

Space group: C 1 2/m 1 (12) Crystal system: Monoclinic

Cell parameters: **a= 14.33900 Å, b= 4.00600 Å, c= 9.87300 Å, β= 95.000 º**

Calculated Density [$g\cdot cm^{-3}$] = 6.26200

Hamaker Constant [eV] = **1.829**

Molecular Mass [$g\cdot mol^{-1}$] = 532.6

$f_{Pauling}$ = **0.0016**

Band gap [eV] = 0.43

$$\varepsilon_\infty = \begin{pmatrix} 25.4 & & \\ & N/A & \\ & & N/A \end{pmatrix}$$

N/A

$E_{total}^{\text{theory (RPA)}}$ [$meV\cdot Å^{-2}$] = **N/A**

$E_{total}^{\text{theory (corrected rVV10)}}$ [$meV\cdot Å^{-2}$]=**N/A**

$E_{total}^{\text{empirical}}$ [$meV\cdot Å^{-2}$] = **17.6**

$E_{dispervise}^{\text{empirical}}$ [$meV\cdot Å^{-2}$] = **17.6**

**Notes**:
Crystal Structure is given in ref.[188]. Band gap of $As_2Te_3$ is reported by Vaney et al.[189] The electronic dielectric constant is an estimation based on the Eqs. (7) and (S18).

# Crystal no. 2. $AsI_3$

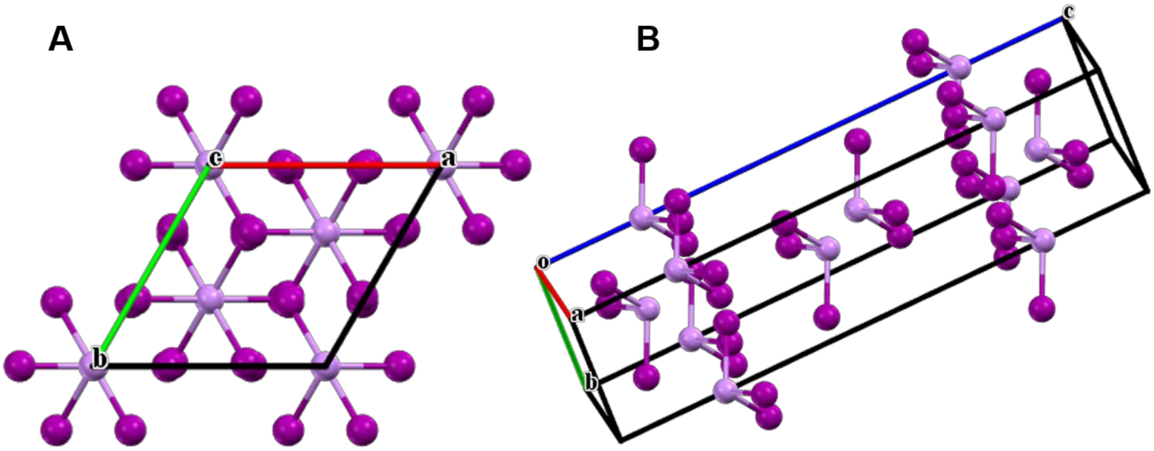


**Fig. S25. Visualization of the packed crystal structure of $AsI_3$.** A) The packed crystal is visualized along c*. B) To better illustrate the 3D structure, the packed cell is visualized employing free-style orientation.

Space group: R -3 (148)

Crystal system: Trigonal (hexagonal axes)

Cell parameters: **a= 7.20800 Å, c= 21.43600 Å**

Density [g·cm$^{-3}$] = 4.70700

Hamaker Constant [eV] = **0.93**

Molecular Mass [g·mol$^{-1}$] = 455.6

$f_{Pauling}$ = **0.056**

Band gap [eV] = 2.4

$$\varepsilon_\infty = \begin{pmatrix} 4.8 & & \\ & 4.8 & \\ & & 3.9 \end{pmatrix}$$

$E_{total}$ $^{theory\ (RPA)}$ [meV·Å$^{-2}$] = **N/A**
$E_{total}$ $^{theory\ (corrected\ rVV10)}$ [meV·Å$^{-2}$]=**N/A**

$$\varepsilon(0) = \begin{pmatrix} 7.9 & & \\ & 7.9 & \\ & & 4.7 \end{pmatrix}$$

$E_{total}$ $^{empirical}$ [meV·Å$^{-2}$] = **9.5**

$E_{dispervise}$ $^{empirical}$ [meV·Å$^{-2}$] = **9.0**

**Notes**:
Crystal Structure is given in ref.[190] Band gap is reported in ref. [191] The band gap in original report was 2.47 eV which is measured at 90 K. Generally, the band gap of semiconductors decreases slightly by increasing temperature, thus the band gap at room temperature is approximated to be 2.4 eV. Dielectric constants (both electronic and static) are listed in ref.[192] The infrared bands are located at 20 and 27 meV, for in-plane and out-of-plane polarizations, respectively.

# Crystal no. 3. $Bi_2O_2Se$

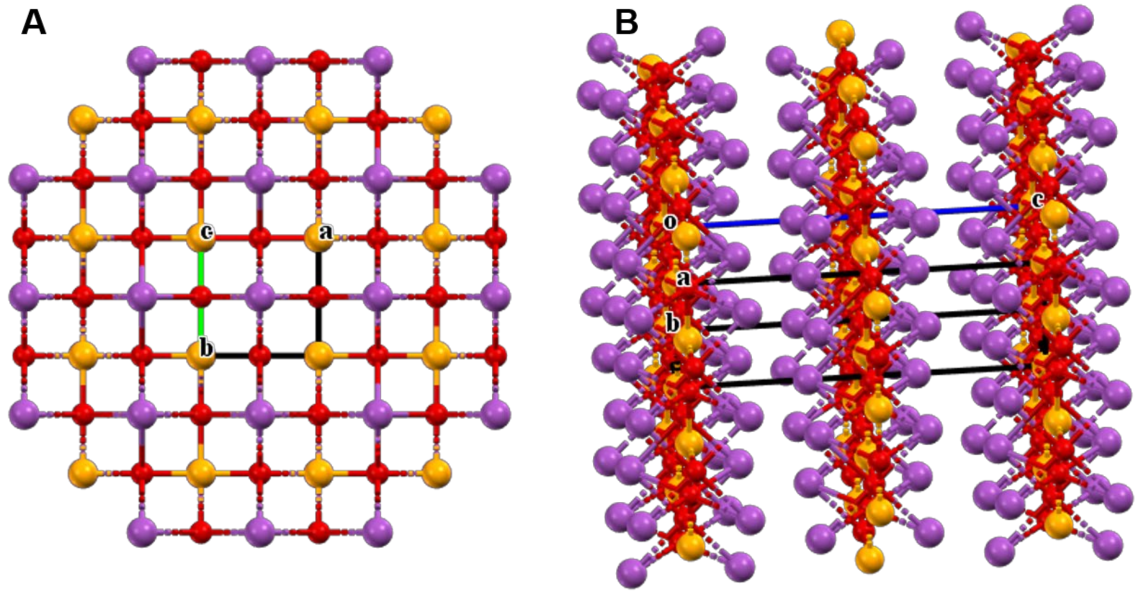


**Fig. S26. Visualization of the packed crystal structure of $Bi_2O_2Se$.** A) The packed crystal is visualized along c*. B) To better illustrate the 3D structure, packed cell is visualized employing free-style orientation.

Space group: I 4/m m m (139)

Crystal system: Tetragonal

Cell parameters: **a= 3.89100 Å, c= 12.21300 Å**

Density [g·cm$^{-3}$] = 9.5000

Hamaker Constant [eV] = **2.05**

Molecular Mass [g·mol$^{-1}$] = 528.9

$f_{Pauling}$ = **0.232**

Band gap [eV] = 0.8

$$\varepsilon_\infty = \begin{pmatrix} 19.1 & & \\ & N/A & \\ & & N/A \end{pmatrix}$$

$E_{total}^{\text{theory (RPA)}}$ [meV·Å$^{-2}$] = **N/A**
$E_{total}^{\text{theory (corrected rVV10)}}$ [meV·Å$^{-2}$]=**N/A**

N/A

$E_{total}^{\text{empirical}}$ [meV·Å$^{-2}$] = **25.7**

$E_{dispervise}^{\text{empirical}}$ [meV·Å$^{-2}$] = **19.7**

**Notes**:
Crystal Structure is given in ref.[193] [193] The band gap is given by Chen *et al.*[194] The electronic dielectric constant is estimated using the Eqs. (7) and (S18).

# Crystal no. 4. $Bi_2S_3$

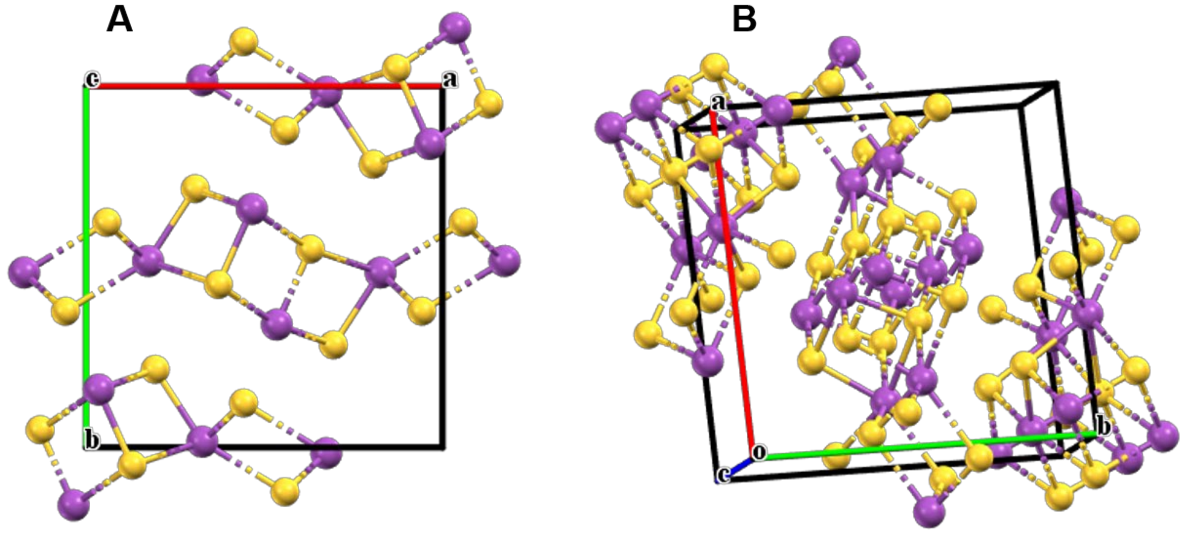


**Fig. S27. Visualization of the packed crystal structure of $Bi_2S_3$.** A) The packed crystal is visualized along c*. B) To better illustrate the 3D structure, packed cell is visualized employing free-style orientation.

Space group: P b n m (62)

Crystal system: Orthorhombic

Cell parameters: a= **11.17000 Å**, b= **11.31900 Å,** c= **3.99200 Å**

Density [g·cm$^{-3}$] = 6.76600

Hamaker Constant [eV] = **1.685**

Molecular Mass [g·mol$^{-1}$] = 514.2

$f_{Pauling}$ = **0.075**

Band gap [eV] = 1.35

$$\varepsilon_\infty = \begin{pmatrix} 10.9 & & \\ & 10.9 & \\ & & 9.6 \end{pmatrix}$$

$E_{total}$ $^{theory (RPA)}$ [meV·Å$^{-2}$] = **N/A**

$E_{total}$ $^{theory (corrected rVV10)}$ [meV·Å$^{-2}$]= **N/A**

$$\varepsilon(0) = \begin{pmatrix} 22.9 & & \\ & 22.9 & \\ & & 21.6 \end{pmatrix}$$

$E_{total}$ $^{empirical}$ [meV·Å$^{-2}$] = **17.5**

$E_{dispervise}$ $^{empirical}$ [meV·Å$^{-2}$] = **16.2**

**Notes**:

Crystal Structure is given in ref.[195] [195] Band gap is reported in ref.[196] Electronic dielectric constants are estimated based on the refractive indices reported by Cantarero *et al.*[196] They have reported slight in-plane anisotropy which has been ignored here. The static dielectric constants are crude estimation based on the measurements by Cantarero *et al.* where they measured infrared reflectivity (unpolarized). The position of the infrared band is assumed to be 27 meV. [196] The same group has measured transport properties of $Bi_2S_3$,[197] based on which we have estimated the Drude band for calculation of the Hamaker constant. The plasma frequencies are found to be 10 and 6.33 meV for in-plane and out-of-plane, respectively. The Drude scattering factor are 67.5 and 54 meV, respectively.

# Crystal no. 5. $Bi_2Se_3$

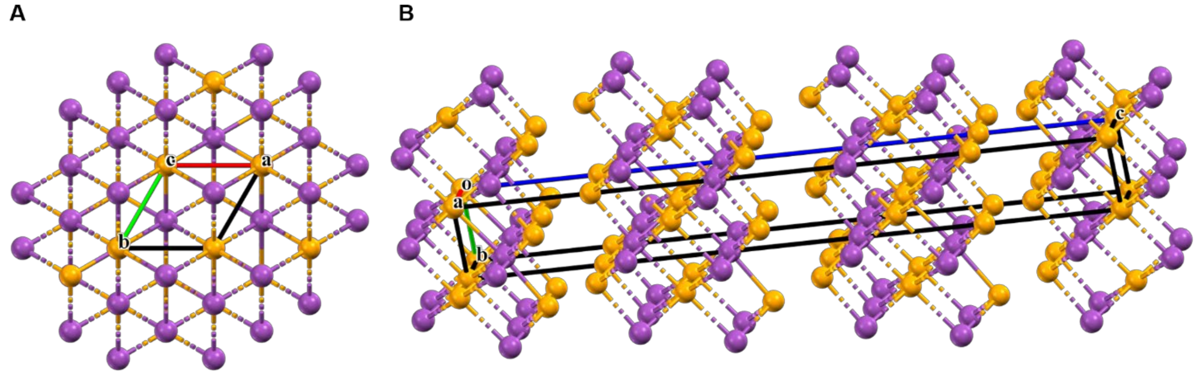


**Fig. S28. Visualization of the packed crystal structure of $Bi_2Se_3$.** A) The packed crystal is visualized along c*. B) To better illustrate the 3D structure, packed cell is visualized employing free-style orientation.

Density [g·cm$^{-3}$] = 7.7

Molecular Mass [g·mol$^{-1}$] = 654.8

Band gap [eV] = 0.35

$$\varepsilon_\infty = \begin{pmatrix} 30.1 & & \\ & 30.1 & \\ & & 21.1 \end{pmatrix}$$

N/A

Hamaker Constant [eV] = **1.855**

$f_{Pauling}$ = **0.068**

$E_{total}^{\text{theory (RPA)}}$ [meV·Å$^{-2}$] = **N/A**
$E_{total}^{\text{theory (corrected rVV10)}}$ [meV·Å$^{-2}$]= **17.56**

$E_{total}^{\text{empirical}}$ [meV·Å$^{-2}$] = **19.2**

$E_{dispervise}^{\text{empirical}}$ [meV·Å$^{-2}$] = **17.9**

**Notes**:
Density is calculated from XRD data in ref.[198] Full spectrum of dielectric function is given in our previous work.[16]

# Crystal no. 6. $Bi_2Sr_2CaCu_2O_8$

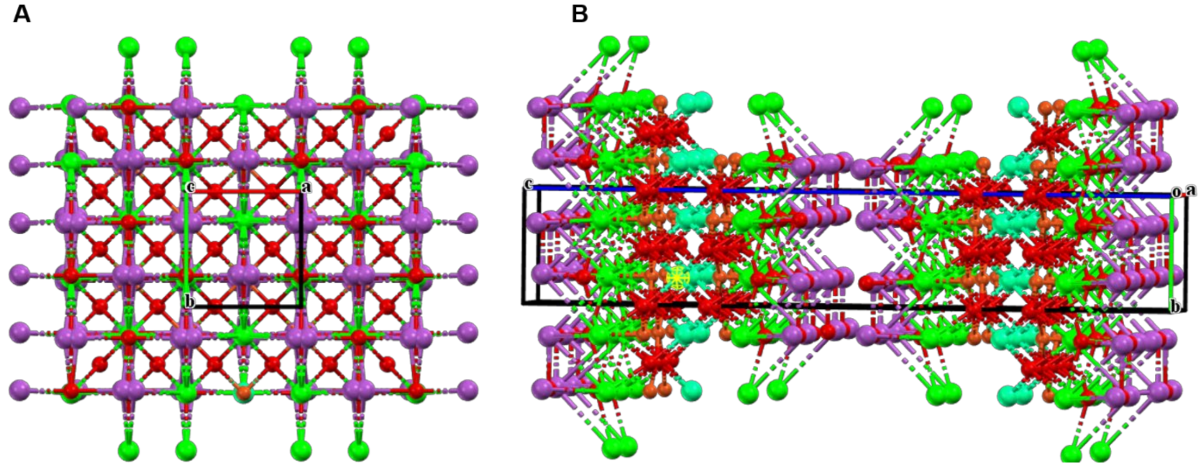

**Fig. S29. Visualization of the packed crystal structure of $Bi_2Sr_2CaCuO_8$.** A) The packed crystal is visualized along c*. B) To better illustrate the 3D structure, packed cell is visualized employing free-style orientation.

Space group: A m a a (66)

Crystal system: Orthorombic

Cell parameters: a= **5.40540 Å**, b= **5.40160 Å**, c= **30.71520 Å**

Density [g·cm$^{-3}$] = 6.57900

Hamaker Constant [eV] = **1.250**

Molecular Mass [g·mol$^{-1}$] = 888.4

$f_{Pauling}$ = **0.396**

Band gap [eV] = 3.1

$$\varepsilon_{\infty} = \begin{pmatrix} 4.3 & & \\ & 4.3 & \\ & & N/A \end{pmatrix}$$

$E_{total}^{theory\ (RPA)}$ [meV·Å$^{-2}$] = **N/A**

$E_{total}^{theory\ (corrected\ rVV10)}$ [meV·Å$^{-2}$]= **30.16**

N/A

$E_{total}^{empirical}$ [meV·Å$^{-2}$] = **20.0**

$E_{dispervise}^{empirical}$ [meV·Å$^{-2}$] = **12.1**

**Notes**:

Crystal Structure is given in ref.[199] [199] Optical properties (band gap, electronic dielectric constant and Drude parameters) are based on the study by Hwang *et al.*[200] The room temperature plasma and scattering frequencies in Drude model are 2.1 and 0.211 eV, respectively (We chose parameters for sample OPT96). Ionicity is estimated form the ionicity of Bi-O bond.

# Crystal no. 7. $Bi_2Te_3$

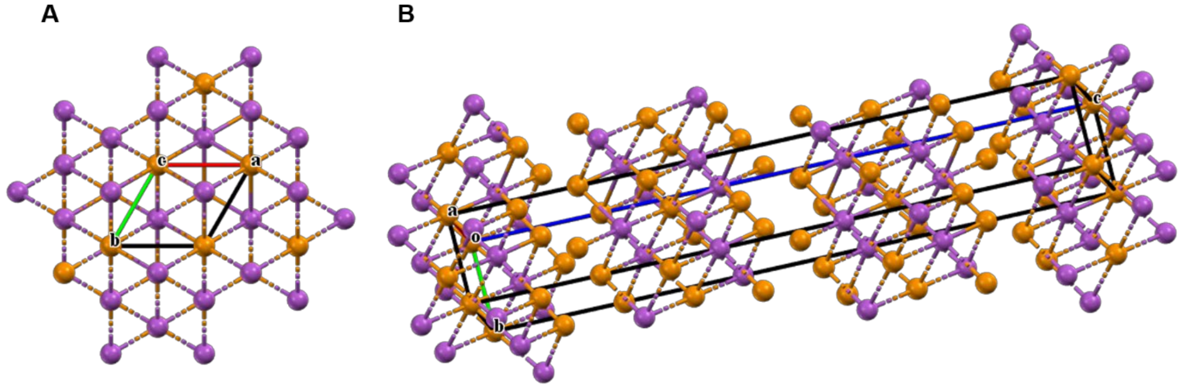


**Fig. S30. Visualization of the packed crystal structure of $Bi_2Te_3$.** A) The packed crystal is visualized along c*. B) To better illustrate the 3D structure, packed cell is visualized employing free-style orientation.

Space group: R -3 m (166)

Crystal system: Trigonal (Hexagonal axes)

Cell parameters: a= **4.38600 Å**, c= **30.49700 Å**

Density [$g{\cdot}cm^{-3}$] = 7.85100

Hamaker Constant [eV] = **1.856**

Molecular Mass [$g{\cdot}mol^{-1}$] = 800.8

$f_{Pauling}$ = **0.002**

Band gap [eV] = 0.14

$$\varepsilon_{\infty} = \begin{pmatrix} 84 & & \\ & 84 & \\ & & 50 \end{pmatrix}$$

$E_{total}^{\text{theory (RPA)}}$ [$meV{\cdot}Å^{-2}$] = **N/A**

$E_{total}^{\text{theory (corrected rVV10)}}$ [$meV{\cdot}Å^{-2}$]= **18.94**

N/A

$E_{total}^{\text{empirical}}$ [$meV{\cdot}Å^{-2}$] = **17.9**

$E_{dispervise}^{\text{empirical}}$ [$meV{\cdot}Å^{-2}$] = **17.9**

**Notes**:

Crystal structure is given in ref.[201] [201] Full spectrum of dielectric function is given in our previous work.[16]

# Crystal no. 8. BiCuSeO

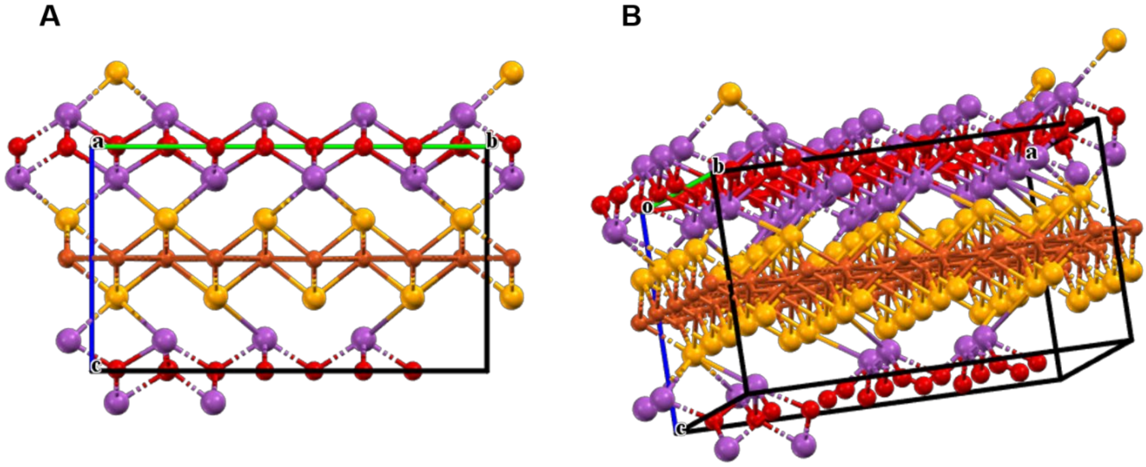


**Fig. S31.Visualization of the packed crystal structure of BiCuSeO**. A) The packed crystal is visualized along a*. B) To better illustrate the 3D structure, packed cell is visualized employing free-style orientation.

Space group: P 1 (1)

Crystal system: Trigonal (Anorthic)

Cell parameters: a= **15.67720 Å, ** b= **15.67720 Å,** c= **8.91500 Å,** α= **90.00º,** β= **90.00 º,** γ= **90.00 º**

Density [g·cm$^{-3}$] = 8.75389

Hamaker Constant [eV] = **2.035**

Molecular Mass [g·mol$^{-1}$] = 367.5

$f_{Pauling}$ = **0.248**

Band gap [eV] = 0.8

$$\varepsilon_{\infty} = \begin{pmatrix} 19.15 & & \\ & 19.15 & \\ & & N/A \end{pmatrix}$$

$E_{total}^{theory\ (RPA)}$ [meV·Å$^{-2}$] = **N/A**

$E_{total}^{theory\ (corrected\ rVV10)}$ [meV·Å$^{-2}$]=**N/A**

N/A

$E_{total}^{empirical}$ [meV·Å$^{-2}$] = **26.1**

$E_{dispervise}^{empirical}$ [meV·Å$^{-2}$] = **19.6**

**Notes**:

Crystal structure is given in ref. [202] [202] Band gap is reported in ref.[203] The electronic dielectric constant is an estimation based on the Eqs. (7) and (S18). The anisotropy is ignored. The reported ionicity is the average value of Bi-O and Cu-Se bonds.

# Crystal no. 9. BiCuSO

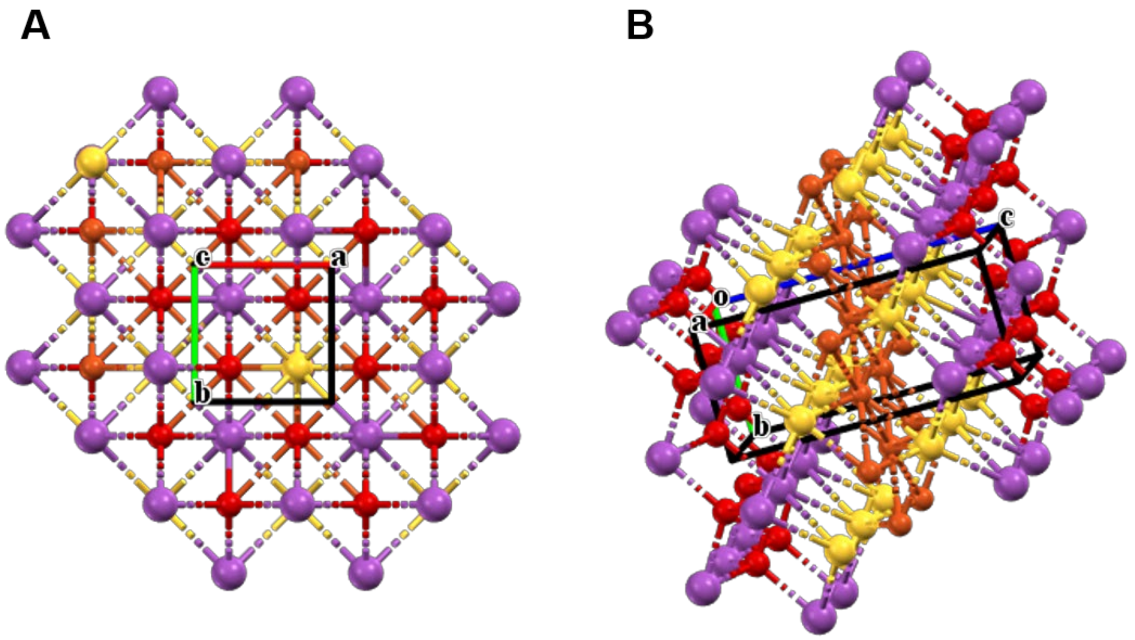


**Fig. S32. Visualization of the packed crystal structure of BiCuSO.** A) The packed crystal is visualized along c*. B) To better illustrate the 3D structure, packed cell is visualized employing free-style orientation.

Space group: P 4/n m m (129)

Crystal system: Tetragonal

Cell parameters: a= **3.86910 Å, c= 8.56020 Å**

Density [g·cm$^{-3}$] = 8.25932

Hamaker Constant [eV] = **1.94**

Molecular Mass [g·mol$^{-1}$] = 320.6

$f_{Pauling}$ = **0.253**

Band gap [eV] = 1.1

$$\varepsilon_{\infty} = \begin{pmatrix} 14.1 & & \\ & 14.1 & \\ & & N/A \end{pmatrix}$$

$E_{total}$ $^{theory\ (RPA)}$ [meV·Å$^{-2}$] = **N/A**

$E_{total}$ $^{theory\ (corrected\ rVV10)}$ [meV·Å$^{-2}$]=**N/A**

N/A

$E_{total}$ $^{empirical}$ [meV·Å$^{-2}$] = **25.0**

$E_{dispervise}$ $^{empirical}$ [meV·Å$^{-2}$] = **18.7**

**Notes**:
Crystal structure is given in ref.[204] Band gap is reported in ref.[205] The electronic dielectric constant is an approximation using the Eqs. (7) and (S18). Anisotropy is ignored. The reported ionicity is the average value of Bi-O and Cu-S bonds.

# Crystal no. 10. $BiI_3$

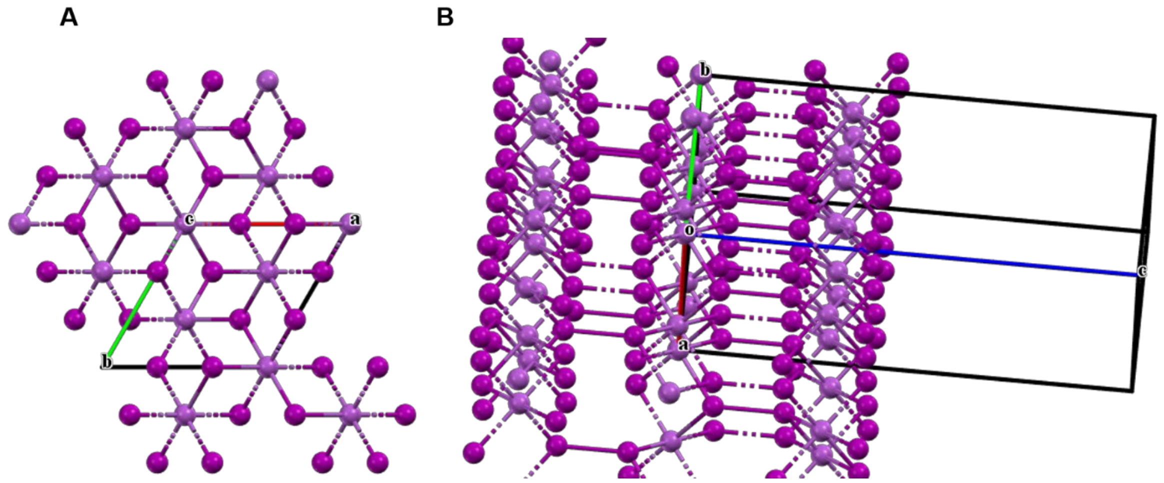


**Fig. S33. Visualization of the packed crystal structure of $BiI_3$.** A) The crystal is visualized along c*. B) To better illustrate the 3D structure, packed cell is visualized employing free-style orientation.

Space group: P 3 (143)

Crystal system: Trigonal (Hexagonal axes)

Cell parameters: a= **7.49800 Å**, c= **20.67600 Å**

Density [g·cm$^{-3}$] = 5.83600

Hamaker Constant [eV] = **1.31**

Molecular Mass [g·mol$^{-1}$] = 589.7

$f_{Pauling}$ = **0.097**

Band gap [eV] = 1.8

$$\varepsilon_\infty = \begin{pmatrix} 7.1 & & \\ & 7.1 & \\ & & 6.4 \end{pmatrix}$$

$E_{total}$ $^{theory\ (RPA)}$ [meV·Å$^{-2}$] = **N/A**
$E_{total}$ $^{theory\ (corrected\ rVV10)}$ [meV·Å$^{-2}$]= **8.712**

$$\varepsilon(0) = \begin{pmatrix} 54 & & \\ & 54 & \\ & & 8.6 \end{pmatrix}$$

$E_{total}$ $^{empirical}$ [meV·Å$^{-2}$] = **13.9**

$E_{dispervise}$ $^{empirical}$ [meV·Å$^{-2}$] = **12.6**

**Notes**:
Crystal structure is given in ref.[206] Band gap is reported in ref.[207] The electronic dielectric constants are cited in ref.[208] The static dielectric constants along with the infrared reflectance spectrum are listed in ref.[209] The infrared bands are approximately located at 8.7 and 18 meV, for in-plane and out-of-plane polarizations, respectively.

# Crystal no. 11. BiOBr

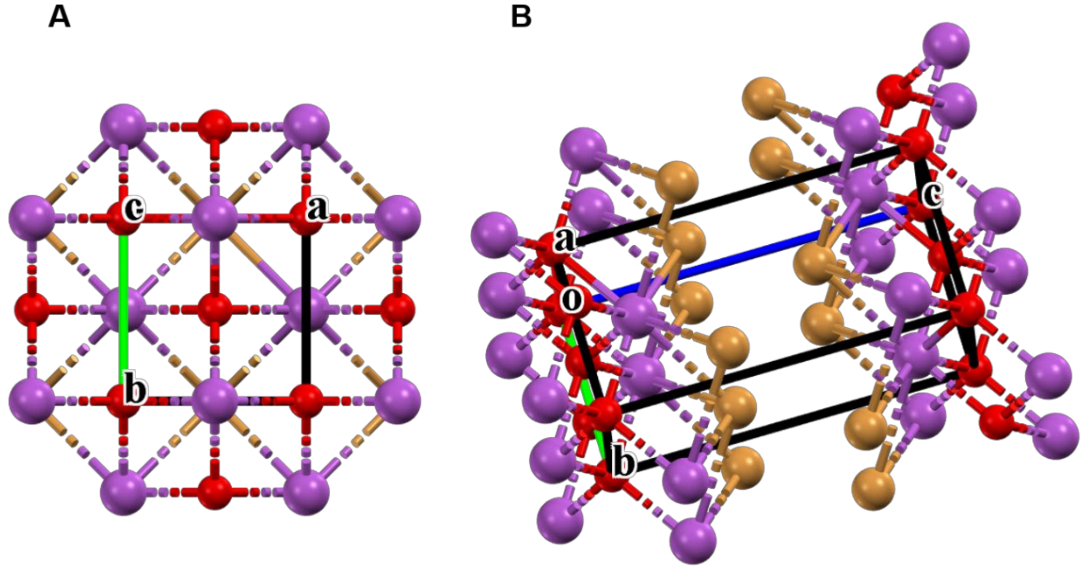


**Fig. S34. Visualization of the packed crystal structure of BiOBr.** A) The crystal is visualized along c*. B) To better illustrate the 3D structure, packed cell is visualized employing free-style orientation.

Space group: P 4/n m m (129) Crystal system: Tetragonal

Cell parameters: a= **3.92000 Å**, c= **8.11000 Å**

Density [g·cm$^{-3}$] = 8.12500

Hamaker Constant [eV] = **1.54**

Molecular Mass [g·mol$^{-1}$] = 304.9

$f_{Pauling}$ = **0.198**

Band gap [eV] = 3

$$\varepsilon_\infty = \begin{pmatrix} 6.5 & & \\ & 6.5 & \\ & & 4.3 \end{pmatrix}$$

N/A

$E_{total}$ $^{theory\ (RPA)}$ [meV·Å$^{-2}$] = **N/A**

$E_{total}$ $^{theory\ (corrected\ rVV10)}$ [meV·Å$^{-2}$]= **17.23**

$E_{total}$ $^{empirical}$ [meV·Å$^{-2}$] = **18.5**

$E_{dispervise}$ $^{empirical}$ [meV·Å$^{-2}$] = **14.8**

**Notes**:
Crystal structure is given in ref.[210] Band gap is listed in ref.[211] The electronic dielectric constant perpendicular to C-axis is approximated using the Eqs. (7) and (S18). Parallel to C-axis, we used the theoretical value for electronic dielectric constant.[50] The ionicity is for Bi-Br bond.

# Crystal no. 12. BiOCl

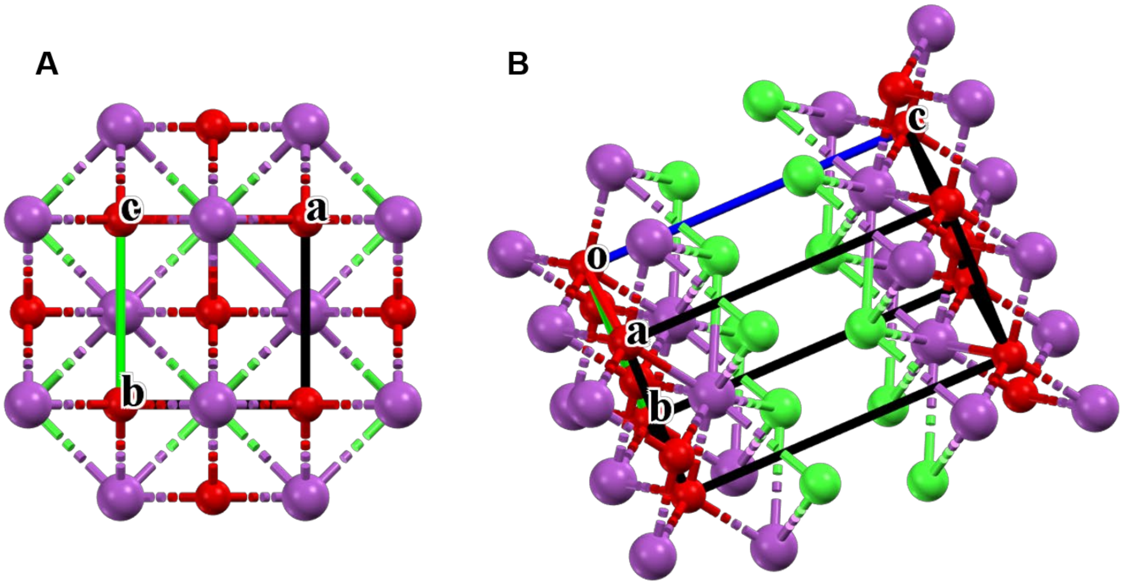


**Fig. S35. Visualization of the packed crystal structure of BiOCl.** A) The crystal is visualized along c*. B) To better illustrate the 3D structure, packed cell is visualized employing free-style orientation.

Space group: P 4/n m m (129)

Crystal system: Tetragonal

Cell parameters: a= **3.89000 Å**, c= **7.37000 Å**

Density [g·cm$^{-3}$] = 7.75500

Hamaker Constant [eV] = **1.68**

Molecular Mass [g·mol$^{-1}$] = 260.4

$f_{Pauling}$ = **0.277**

Band gap [eV] = 3.4

$$\varepsilon_\infty = \begin{pmatrix} 6.25 & & \\ & 6.25 & \\ & & 4.1 \end{pmatrix}$$

$E_{total}$ $^{theory\ (RPA)}$ [meV·Å$^{-2}$] = **N/A**

$E_{total}$ $^{theory\ (corrected\ rVV10)}$ [meV·Å$^{-2}$]= **20.59**

N/A

$E_{total}$ $^{empirical}$ [meV·Å$^{-2}$] = **22.3**

$E_{dispervise}$ $^{empirical}$ [meV·Å$^{-2}$] = **16.2**

**Notes**:

Crystal structure is reported in ref.[210] The band gap is reported in ref.[212] The electronic dielectric constant in perpendicular to C-axis is approximated using the refractive indices reported by Unuma *et al.*[213] It is somewhat larger than previous reports.[214] The electronic dielectric constant in other polarization is estimated assuming a birefringency of 0.24 in visible region.[215] The ionicity is for Bi-Cl bond.

# Crystal no. 13. BiOI

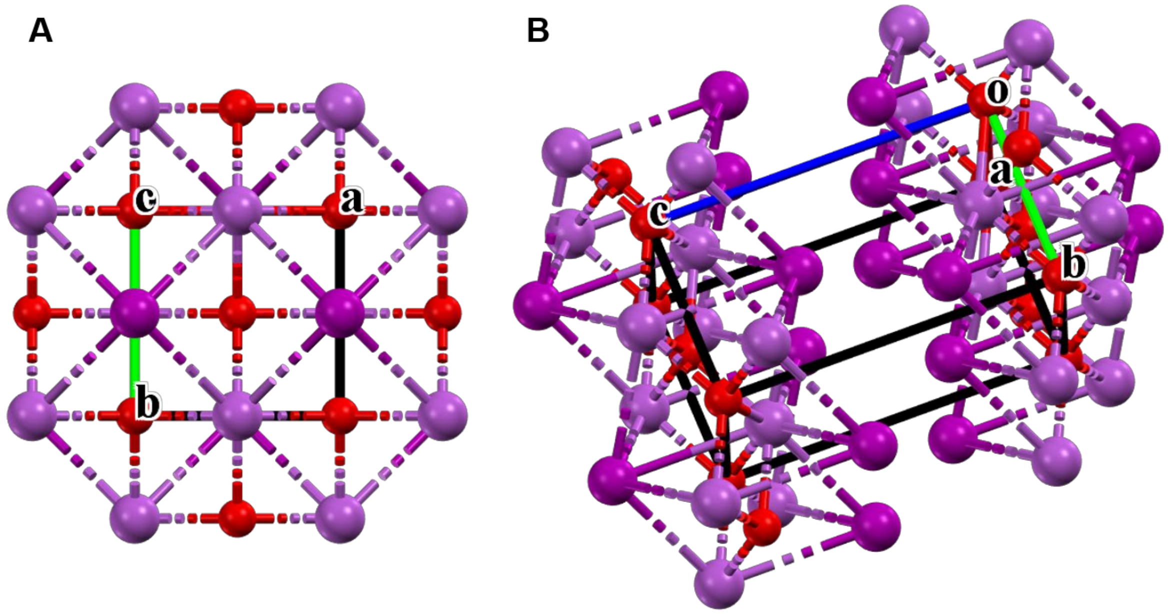


**Fig. S36. Visualization of the packed crystal structure of BiOI.** A) The crystal is visualized along c*. B) To better illustrate the 3D structure, packed cell is visualized employing free-style orientation.

Space group: P 4/n m m (129)

Crystal system: Tetragonal

Cell parameters: a= **4.01000 Å**, c= **9.14000 Å**

Density [g·cm$^{-3}$] = 7.900

Hamaker Constant [eV] = **1.49**

Molecular Mass [g·mol$^{-1}$] = 351.9

$f_{Pauling}$ = **0.097**

Band gap [eV] = 1.9

$$\varepsilon_\infty = \begin{pmatrix} 8.9 & & \\ & 8.9 & \\ & & 4.42 \end{pmatrix}$$

$E_{total}^{theory\ (RPA)}$ [meV·Å$^{-2}$] = **N/A**
$E_{total}^{theory\ (corrected\ rVV10)}$ [meV·Å$^{-2}$]= **13.86**

N/A

$E_{total}^{empirical}$ [meV·Å$^{-2}$] = **15.9**

$E_{dispervise}^{empirical}$ [meV·Å$^{-2}$] = **14.4**

**Notes**:
Crystal structure is reported in ref.[210] Band gap is listed in ref.[216] The electronic dielectric constant perpendicular to C-axis is approximated using the Eqs. (7) and (S18). Parallel to C-axis, we used the theoretical value for electronic dielectric constant.[50] The ionicity is for Bi-I bond.

# Crystal no. 14. BiTeBr

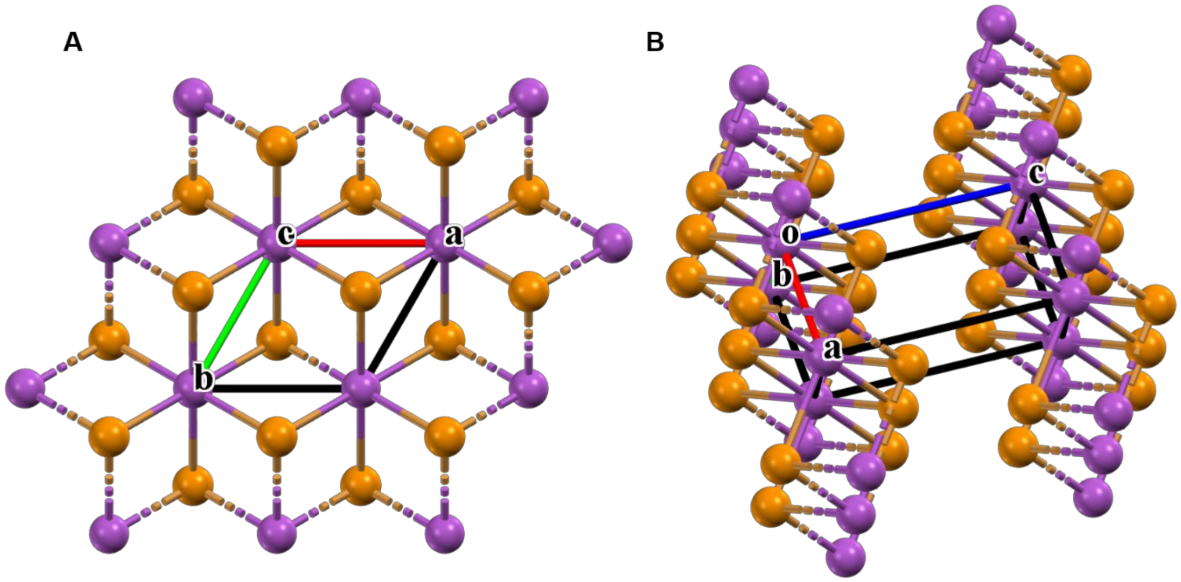


**Fig. S37. Visualization of the packed crystal structure of BiTeBr.** A) The crystal is visualized along c*. B) To better illustrate the 3D structure, packed cell is visualized employing free-style orientation.

Space group: P -3 m 1 (164)

Crystal system: Trigonal (Hexagonal axes)

Cell parameters: a= **4.23000 Å**, c= **6.47000 Å**

Density [g·cm$^{-3}$] = 6.89800

Hamaker Constant [×10$^{-21}$ J] = **1.40**

Molecular Mass [g·mol$^{-1}$] = 416.5

$f_{Pauling}$ = **0.1**

Band gap [eV] = 0.6

$$\varepsilon_\infty = \begin{pmatrix} 14.4 & & \\ & 14.4 & \\ & & N/A \end{pmatrix}$$

$E_{total}^{theory\ (RPA)}$ [meV·Å$^{-2}$] = **N/A**

$E_{total}^{theory\ (corrected\ rVV10)}$ [meV·Å$^{-2}$]= **15.05**

N/A

$E_{total}^{empirical}$ [meV·Å$^{-2}$] = **15.0**

$E_{dispervise}^{empirical}$ [meV·Å$^{-2}$] = **13.5**

**Notes**:

Crystal Structure is reported in ref.[217] The band gap and dielectric constant are estimated from the work of Martin *et al.*[218] We have included a Drude band in dielectric function for calculating the Hamaker constant. The plasma and scattering frequencies are 208 and 24.8 meV.[218] The ionicity is the average value of Bi-Te and Bi-Br bond. Theoretical binding energy is the average value for BiTeCl and BiTeI.

# Crystal no. 15. BiTeCl

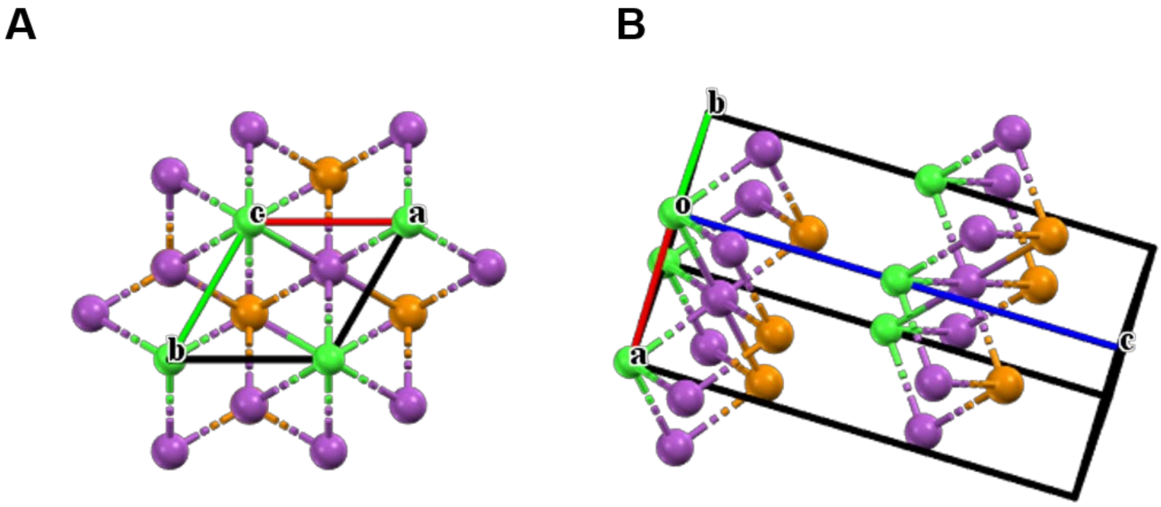


**Fig. S38. Visualization of the packed crystal structure of BiTeCl.** A) The crystal is visualized along c*. B) To better illustrate the 3D structure, packed cell is visualized employing free-style orientation.

Density [g·cm$^{-3}$] = 6.4

Molecular Mass [g·mol$^{-1}$] = 372.0

Band gap [eV] = 0.7

$$\varepsilon_\infty = \begin{pmatrix} 15.2 & & \\ & 15.2 & \\ & & N/A \end{pmatrix}$$

N/A

Hamaker Constant [eV] = **1.51**

$f_{Pauling}$ = **0.139**

$E_{total}$ $^{theory\ (RPA)}$ [meV·Å$^{-2}$] = **N/A**
$E_{total}$ $^{theory\ (corrected\ rVV10)}$ [meV·Å$^{-2}$]= **15.64**

$E_{total}$ $^{empirical}$ [meV·Å$^{-2}$] = **16.9**

$E_{dispervise}$ $^{empirical}$ [meV·Å$^{-2}$] = **14.5**

**Notes**:
Density is calculated from the XRD data in ref.[219] Band gap is reported in refs.[220, 221] Electronic dielectric constant is estimated from the reflectivity spectrum reported in ref.[221] We also included a Drude band in dielectric function with plasma and scattering frequencies of 316 and 22.4 meV, respectively.[221] This is equivalent to DC conductivity of 60 S·cm$^{-1}$, which is consistent with ref.[222] Anisotropy is ignored. The ionicity is the average value of Bi-Te and Bi-Cl bond.

# Crystal no. 16. BiTeI

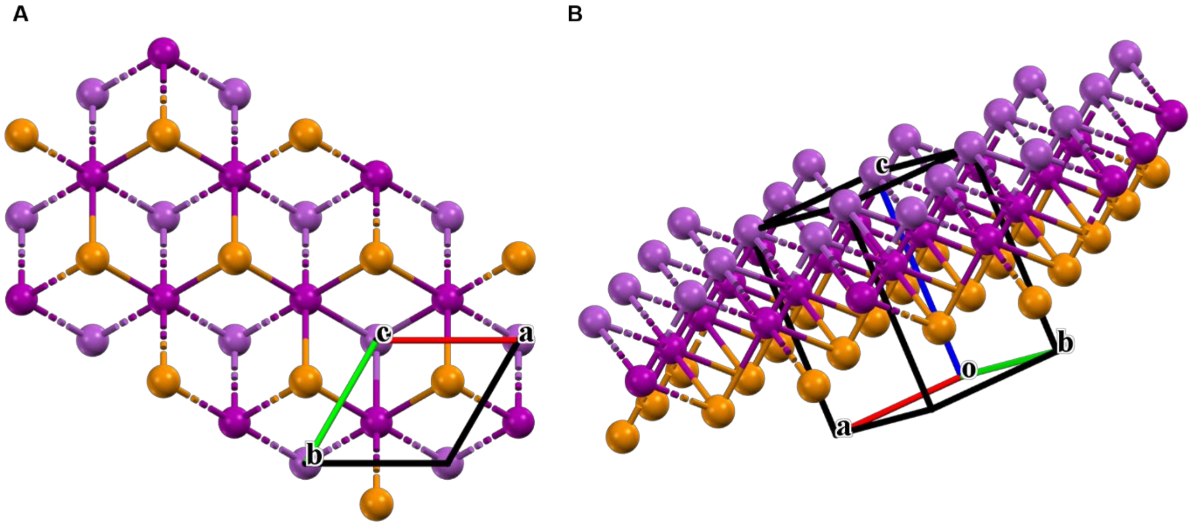


**Fig. S39. Visualization of the packed crystal structure of BiTeI.** A) The crystal is visualized along c*. B) To better illustrate the 3D structure, packed cell is visualized employing free-style orientation.

Space group: P 3 (143)

Crystal system: Trigonal (Hexagonal axes)

Cell parameters: a= **4.34600 Å**, c= **6.83500 Å**

Density [g·cm$^{-3}$] = 6.88400

Hamaker Constant [eV] = **1.50**

Molecular Mass [g·mol$^{-1}$] = 463.5

$f_{Pauling}$ = **0.05**

Band gap [eV] = 0.38

$$\varepsilon_\infty = \begin{pmatrix} 19 & & \\ & 19 & \\ & & N/A \end{pmatrix}$$

$E_{total}$ $^{theory\ (RPA)}$ [meV·Å$^{-2}$] = **N/A**

$E_{total}$ $^{theory\ (corrected\ rVV10)}$ [meV·Å$^{-2}$]= **14.45**

N/A

$E_{total}$ $^{empirical}$ [meV·Å$^{-2}$] = **15.2**

$E_{dispervise}$ $^{empirical}$ [meV·Å$^{-2}$] = **14.5**

**Notes**:

Crystal structure is reported in ref.[223] The band gap is reported by Ishizaka *et al.*[224] which is consistent with the measurements by Tran *et al.*[225] and Lostak *et al.*[226] The electronic dielectric constant is reported by Lostak *et al.*[226] They have also reported the Drude band with plasma and scattering frequencies of 3.57 and 0.345 eV, respectively. This is equivalent to DC conductivity of 497 S·cm$^{-1}$ which is consistent with independent experimental measurements.[227] Anisotropy is ignored. The ionicity is the average value of Bi-Te and Bi-I bond.

# Crystal no. 17. Boron Nitride, hexagonal

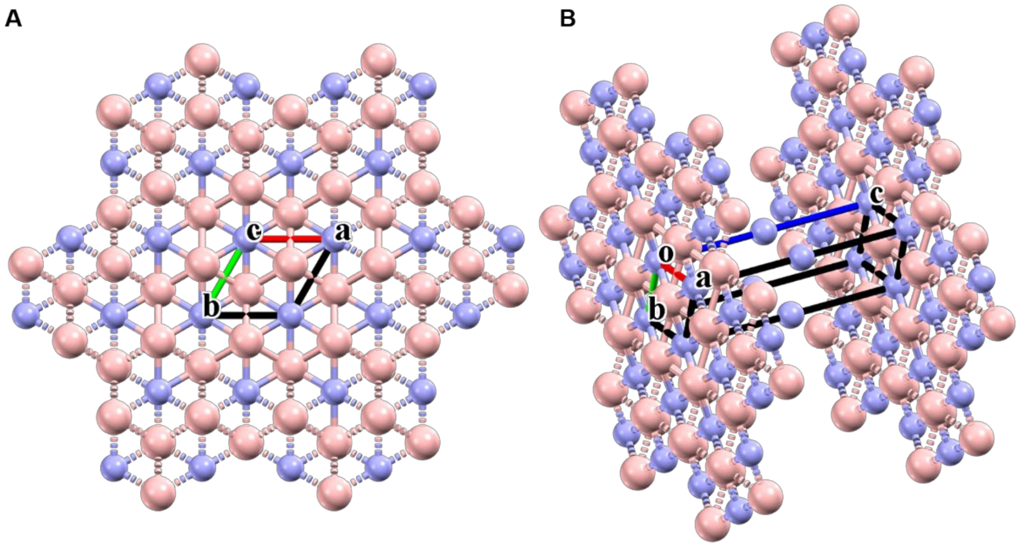


**Fig. S40. Visualization of the packed crystal structure of BN.** A) The packed crystal is visualized along c*. B) To better illustrate the 3D structure, packed cell is visualized employing free-style orientation.

Space group: P -3 m 1 (164)

Crystal system: Trigonal (Hexagonal axes)

Cell parameters: a= **2.51000 Å**, c= **6.69000 Å**

Density [g·cm$^{-3}$] = 2.258

Hamaker Constant [eV] = **1.032**

Molecular Mass [g·mol$^{-1}$] = 24.82

$f_{Pauling}$ = **0.211**

Band gap [eV] = 5.8

$$\varepsilon_\infty = \begin{pmatrix} 4.71 & & \\ & 4.71 & \\ & & 2.56 \end{pmatrix}$$

$E_{total}$ $^{theory\ (RPA)}$ [meV·Å$^{-2}$] = **14.49**

$E_{total}$ $^{theory\ (corrected\ rVV10)}$ [meV·Å$^{-2}$]= **16.10**

$$\varepsilon(0) = \begin{pmatrix} 6.94 & & \\ & 6.94 & \\ & & 3.22 \end{pmatrix}$$

$E_{total}$ $^{empirical}$ [meV·Å$^{-2}$] = **12.6**

$E_{dispervise}$ $^{empirical}$ [meV·Å$^{-2}$] = **10.0**

**Notes**:
Crystal structure is reported in ref.[228] Full spectrum of the dielectric function of hexagonal boron nitride is given in our previous work.[16]

# Crystal no. 18. C (graphite)

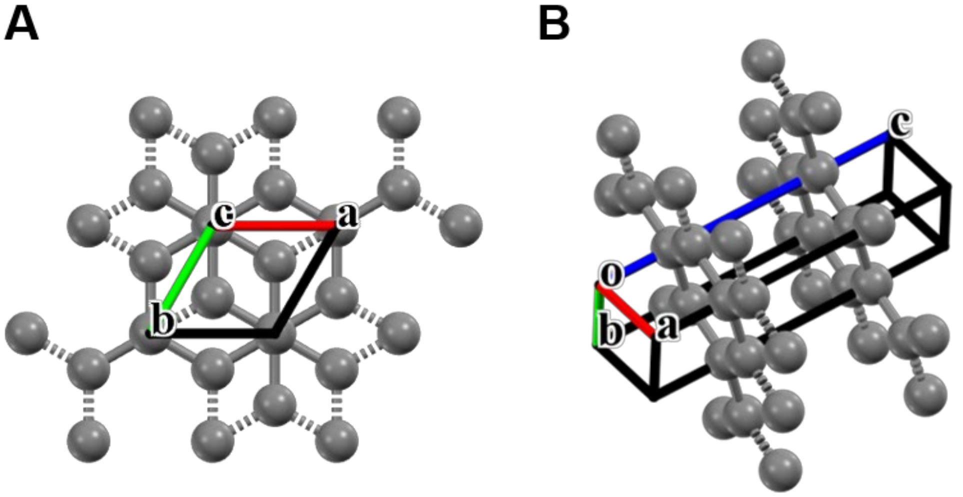


**Fig. S41. Visualization of the packed crystal structure of graphite.** A) The packed crystal is visualized along c*. B) To better illustrate the 3D structure, packed cell is visualized employing free-style orientation.

Space group: P 63 m c (186)

Crystal system: Hexagonal

Cell parameters: a= **2.46400 Å**, c= **6.71100 Å**

Density [g·cm$^{-3}$] = 2.26

Hamaker Constant [eV] = **1.495**

Molecular Mass [g·mol$^{-1}$] = 12

$f_{Pauling}$ = **0**

Band gap [eV] = N/A

N/A

$E_{total}$ $^{theory\ (RPA)}$ [meV·Å$^{-2}$] = **18.32**
$E_{total}$ $^{theory\ (corrected\ rVV10)}$ [meV·Å$^{-2}$]= **16.83**
$E_{total}$ $^{experimental}$ [meV·Å$^{-2}$] = **15.5 ± 3.2 (N=24)**

N/A

$E_{total}$ $^{empirical}$ [meV·Å$^{-2}$] = **14.4**

$E_{dispervise}$ $^{empirical}$ [meV·Å$^{-2}$] = **14.4**

**Notes**:
Crystal structure is given in ref.[229] Full spectrum of the dielectric function of graphite is given in our previous work.[16] The experimental binding energy is the average value of the reported values in literature which are listed in Table S2.

# Crystal no. 19. $(C_4H_9NH_3)_2(CH_3NH_3)_3Pb_4I_{13}$

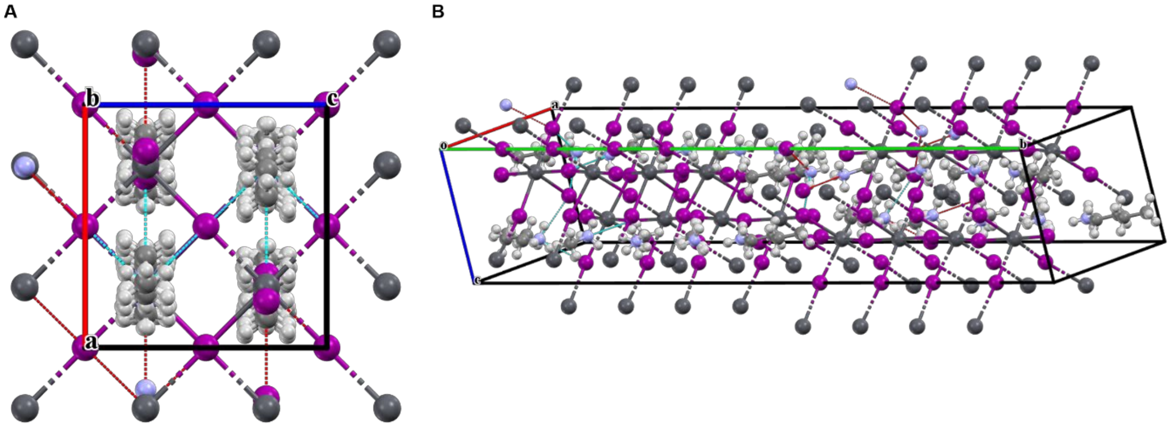

**Fig S42. Visualization of the packed crystal structure of $C_{11}H_{42}N_5I_{13}Pb_4$.** A) The packed crystal is visualized along b*. B) To better illustrate the 3D structure, packed cell is visualized employing free-style orientation. Hydrogen bondings are also illustrated using dashed blue and red lines.

Space group: C c 2 m (40)

Crystal system: Orthorombic

Cell parameters: a= **8.92740 Å**, b= **64.38300 Å,** c= **8.88160 Å**

Density [g·cm$^{-3}$] = 3.53400

Hamaker Constant [eV] = **0.75**

Molecular Mass [g·mol$^{-1}$] = 2723

$f_{Pauling} \approx$ **0**

Band gap [eV] = 2.07

$$\varepsilon_\infty = \begin{pmatrix} 4.4 & & \\ & 4.4 & \\ & & N/A \end{pmatrix}$$

$E_{total}$ $^{theory\ (RPA)}$ [meV·Å$^{-2}$] = **N/A**

$E_{total}$ $^{theory\ (corrected\ rVV10)}$ [meV·Å$^{-2}$]= **N/A**

N/A

$E_{total}$ $^{empirical}$ [meV·Å$^{-2}$] = **7.2**

$E_{dispervise}$ $^{empirical}$ [meV·Å$^{-2}$] = **7.2**

**Notes**:

Crystal structure is reported in ref.[230] Band gap and electronic dielectric constant is given in ref.[231] The ionicity is assumed to be zero due to the low polarity of both hydrocarbon chains and Pb-I bond.

# Crystal no. 20. $Ca(OH)_2$

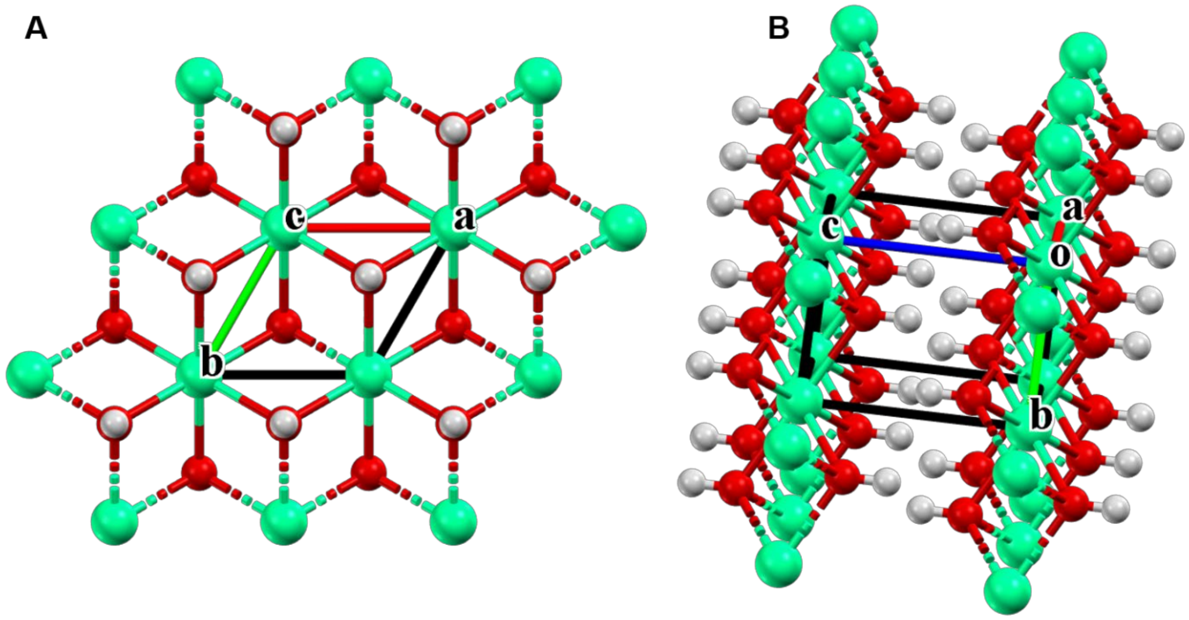


**Fig S43. Visualization of the packed crystal structure of $Ca(OH)_2$.** A) The packed crystal is visualized along c*. B) To better illustrate the 3D structure, packed cell is visualized employing free-style orientation.

Space group: P -3 m 1 (164)

Crystal system: Trigonal (Hexagonal axes)

Cell parameters: a= **3.58620 Å**, c= **4.88010 Å**

Density [g·cm$^{-3}$] = 2.26300

Hamaker Constant [eV] = **0.447**

Molecular Mass [g·mol$^{-1}$] = 74.1

$f_{Pauling}$ = **0.774**

Band gap [eV] = 4.5

$$\varepsilon_\infty = \begin{pmatrix} 2.4 & & \\ & 2.4 & \\ & & N/A \end{pmatrix}$$

$E_{total}$ $^{theory\ (RPA)}$ [meV·Å$^{-2}$] = **N/A**

$E_{total}$ $^{theory\ (corrected\ rVV10)}$ [meV·Å$^{-2}$]= **18.08**

N/A

$E_{total}$ $^{empirical}$ [meV·Å$^{-2}$] = **19.0**

$E_{dispervise}$ $^{empirical}$ [meV·Å$^{-2}$] = **4.3**

**Notes**:
Crystal structure is reported in ref.[232] Band gap is cited in ref.[233] Electronic dielectric constant is approximated from the reported refractive indices.[234] The ionicity of Ca-O is cited.

# Crystal no. 21. $CdBr_2$

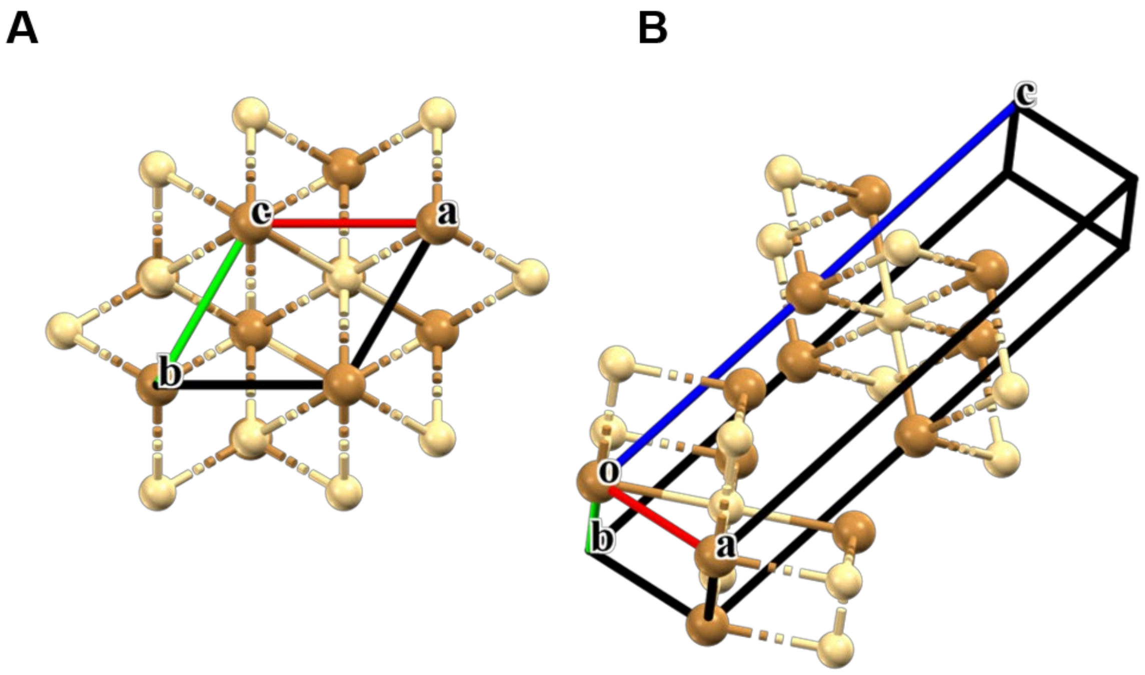


**Fig. S44. Visualization of the packed crystal structure of $CdBr_2$.** A) The packed crystal is visualized along c*. B) To better illustrate the 3D structure, packed cell is visualized employing free-style orientation.

Space group: P -3 m 1 (164)

Crystal system: Trigonal (Hexagonal axes)

Cell parameters: **a= 4.24400 Å, c= 6.85900 Å**

Density [g·cm$^{-3}$] = 5.23400

Hamaker Constant [×10$^{-21}$ J meV] = **0.971**

Molecular Mass [g·mol$^{-1}$] = 272.2

$f_{Pauling}$ = **0.33**

Band gap [eV] = 4.6

$$\varepsilon_\infty = \begin{pmatrix} 3.87 & & \\ & 3.87 & \\ & & 3.2 \end{pmatrix}$$

$E_{total}^{theory\ (RPA)}$ [meV·Å$^{-2}$] = **N/A**

$E_{total}^{theory\ (corrected\ rVV10)}$ [meV·Å$^{-2}$]= **11.55**

$$\varepsilon(0) = \begin{pmatrix} 8.6 & & \\ & 8.6 & \\ & & 3.65 \end{pmatrix}$$

$E_{total}^{empirical}$ [meV·Å$^{-2}$] = **13.9**

$E_{dispervise}^{empirical}$ [meV·Å$^{-2}$] = **9.34**

**Notes**:
Density is calculated form the XRD data in ref.[235] Crystal Full spectrum of dielectric function (in-plane) is used. Details are given in section 12.

# Crystal no. 22. $CdI_2$

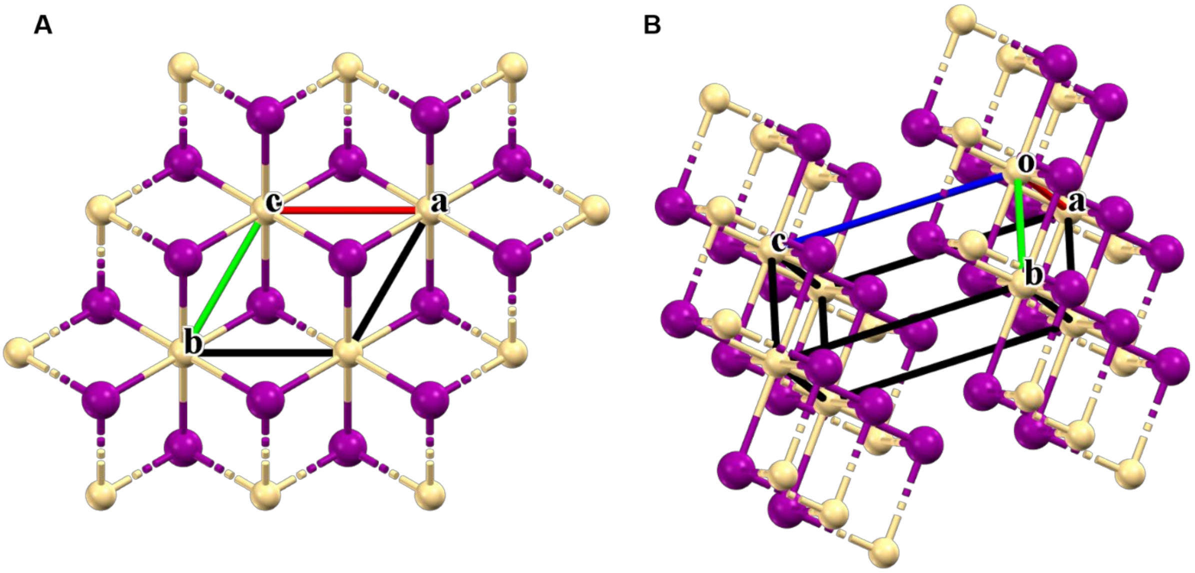


**Fig. S45. Visualization of the packed crystal structure of $CdI_2$.** A) The packed crystal is visualized along c*. B) To better illustrate the 3D structure, packed cell is visualized employing free-style orientation.

Space group: P -3 m 1 (164)

Crystal system: Trigonal (Hexagonal axes)

Cell parameters: **a= 4.24400 Å, c= 6.85900 Å**

Density [g·cm$^{-3}$] = 5.68400

Hamaker Constant [eV] = **1.131**

Molecular Mass [g·mol$^{-1}$] = 366.2

$f_{Pauling}$ = **0.21**

Band gap [eV] = 3.4

$$\varepsilon_\infty = \begin{pmatrix} 4.6 & & \\ & 4.6 & \\ & & 4.3 \end{pmatrix}$$

$E_{total}$ $^{theory\ (RPA)}$ [meV·Å$^{-2}$] = **N/A**
$E_{total}$ $^{theory\ (corrected\ rVV10)}$ [meV·Å$^{-2}$]= **11.15**

$$\varepsilon(0) = \begin{pmatrix} 12.9 & & \\ & 12.9 & \\ & & 5.5 \end{pmatrix}$$

$E_{total}$ $^{empirical}$ [meV·Å$^{-2}$] = **13.8**

$E_{dispervise}$ $^{empirical}$ [meV·Å$^{-2}$] = **10.9**

**Notes**:
Crystal structure is given in ref.[236] Band gap is reported in ref.[237] Dielectric constants (both electronic and static) are reported by Haas.[81] infrared bands are located at 9.8 and 16.9 meV, for in-plane and out-of-plane polarization, respectively.

# Crystal no. 23. $CdPS_3$

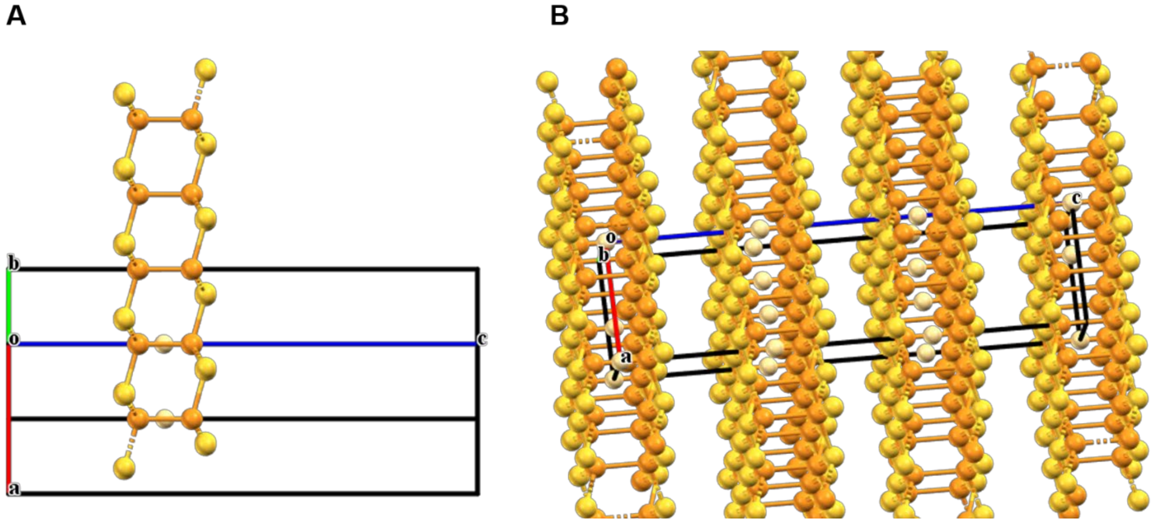

**Fig. S46. Visualization of the packed crystal structure of $CdPS_3$.** A) The packed crystal is visualized along b*. B) To better illustrate the 3D structure, packed cell is visualized employing free-style orientation.

Space group: C R -3 (148)

Crystal system: Trigonal (Hexagonal axes)

Cell parameters: a= **6.22400 Å**, c= **19.49000 Å**

Density [g·cm$^{-3}$] = 3.64900

Hamaker Constant [eV] = **1.15**

Molecular Mass [g·mol$^{-1}$] = 239.6

$f_{Pauling}$ = **0.18**

Band gap [eV] = 3

$$\varepsilon_\infty = \begin{pmatrix} 4.9 & & \\ & 4.9 & \\ & & N/A \end{pmatrix}$$

$E_{total}$ $^{theory\ (RPA)}$ [meV·Å$^{-2}$] = **N/A**

$E_{total}$ $^{theory\ (corrected\ rVV10)}$ [meV·Å$^{-2}$]= **13.2**

N/A

$E_{total}$ $^{empirical}$ [meV·Å$^{-2}$] = **13.6**

$E_{dispervise}$ $^{empirical}$ [meV·Å$^{-2}$] = **11.2**

**Notes**:

Crystal structure is given in ref.[238] Band gap is reported in ref.[239] The electronic dielectric constant is approximated using the Eqs. (7) and (S18). Anisotropy is ignore. The ionicity is for Cd-S bond.

# Crystal no. 24. $CeTe_3$

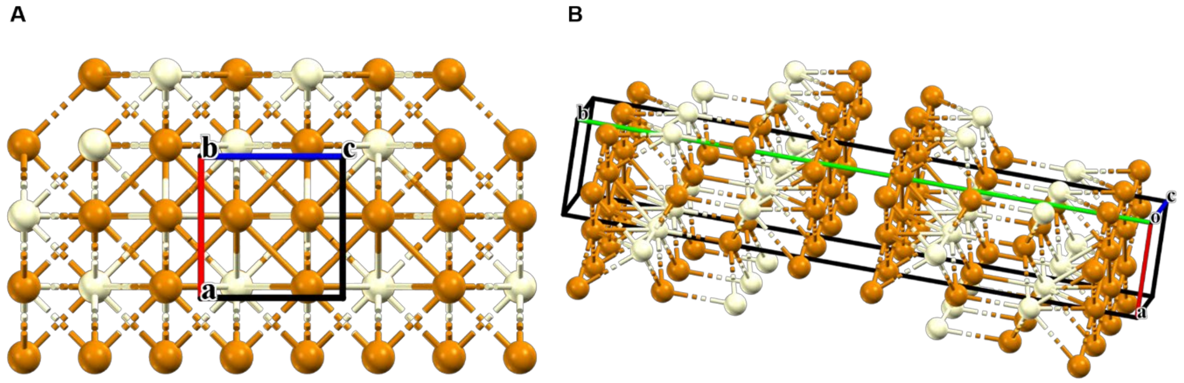


**Fig. S47. Visualization of the packed crystal structure of $CeTe_3$.** A) The packed crystal is visualized along b*. B) To better illustrate the 3D structure, packed cell is visualized employing free-style orientation.

Space group: C 2 c m (40)

Crystal system: Orthorombic

Cell parameters: **a= 4.37320 Å, b= 25.97300 Å, c= 4.38490 Å**

Density [g·cm$^{-3}$] = 6.97300

Hamaker Constant [eV] = **1.85**

Molecular Mass [g·mol$^{-1}$] = 522.9

$f_{Pauling}$ = **0.213**

Band gap [eV] = N/A

N/A

$E_{total}^{theory\ (RPA)}$ [meV·Å$^{-2}$] = **N/A**

$E_{total}^{theory\ (corrected\ rVV10)}$ [meV·Å$^{-2}$] = **17.69**

N/A

$E_{total}^{empirical}$ [meV·Å$^{-2}$] = **22.7**

$E_{dispervise}^{empirical}$ [meV·Å$^{-2}$] = **17.8**

**Notes**:
Crystal Structure is given in ref.[240] The dielectric function was estimated using the reflectivity data measured by Sacchetti *et al*.[241] Anisotropy is ignored.

# Crystal no. 25. $Co(OH)_2$

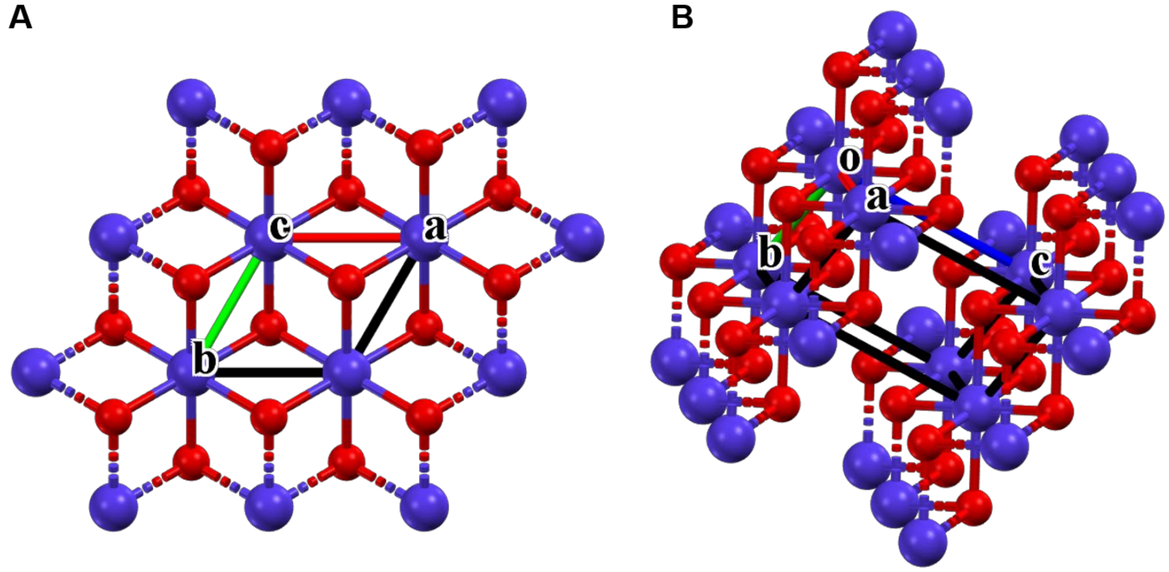


**Fig. S48. Visualization of the packed crystal structure of $Co(OH)_2$.** A) The packed crystal is visualized along c*. B) To better illustrate the 3D structure, packed cell is visualized employing free-style orientation.

Space group: P -3 m 1 (164)

Crystal system: Trigonal (Hexagonal axes)

Cell parameters: a= **3.17300 Å**, c= **4.64000 Å**

Density [g·cm$^{-3}$] = 3.73200

Hamaker Constant [eV] = **1.39**

Molecular Mass [g·mol$^{-1}$] = 92.95

$f_{Pauling}$ = **0.456**

Band gap [eV] = 2.85

$$\varepsilon_\infty = \begin{pmatrix} 5.8 & & \\ & 5.8 & \\ & & N/A \end{pmatrix}$$

$E_{total}$ $^{theory\ (RPA)}$ [meV·Å$^{-2}$] = **N/A**

$E_{total}$ $^{theory\ (corrected\ rVV10)}$ [meV·Å$^{-2}$] = **25.01**

N/A

$E_{total}$ $^{empirical}$ [meV·Å$^{-2}$] = **24.6**

$E_{dispervise}$ $^{empirical}$ [meV·Å$^{-2}$] = **13.4**

**Notes**:

Crystal Structure is given in ref.[236] Band gap is reported in ref.[242] The electronic dielectric constant is approximated using the Eqs. (7) and (S18). Anisotropy is ignored. Ionicity is for Co-O bond.

# Crystal no. 26. $CoCl_2$

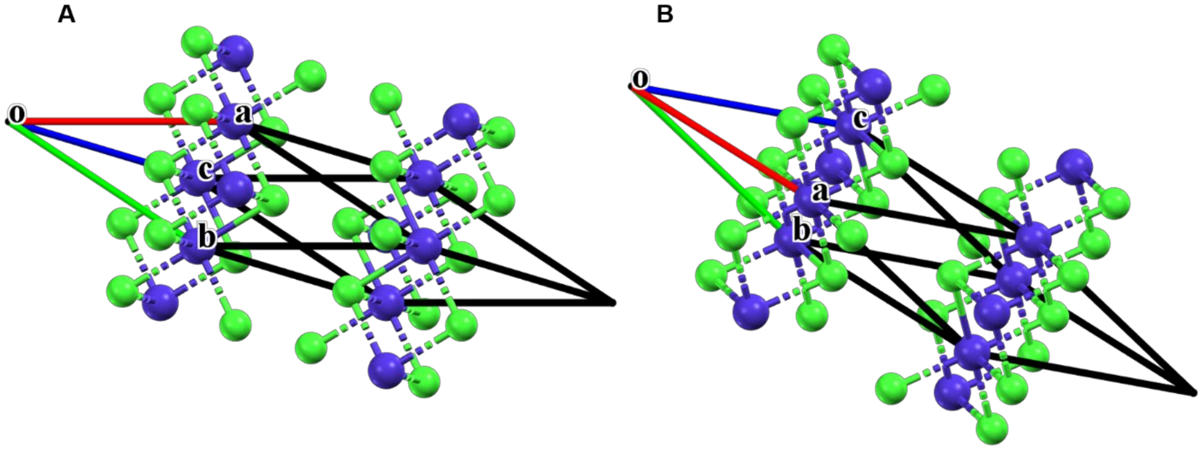


**Fig. S49. Visualization of the packed crystal structure of $CoCl_2$.** A) The packed crystal is visualized along c*. B) To better illustrate the 3D structure, packed cell is visualized employing free-style orientation.

Space group: R -3 m (166)

Crystal system: Trigonal (Rhombohedral axes)

Cell parameters: a= **6.16000 Å**, α= **33.430º**

Density [g·cm$^{-3}$] = 3.41200

Hamaker Constant [eV] = **0.77**

Molecular Mass [g·mol$^{-1}$] = 129.8

$f_{Pauling}$ = **0.336**

Band gap [eV] = 4

$$\varepsilon_\infty = \begin{pmatrix} 3.3 & & \\ & 3.3 & \\ & & N/A \end{pmatrix}$$

$E_{total}$ $^{theory\ (RPA)}$ [meV·Å$^{-2}$] = **N/A**

$E_{total}$ $^{theory\ (corrected\ rVV10)}$ [meV·Å$^{-2}$] = **10.49**

N/A

$E_{total}$ $^{empirical}$ [meV·Å$^{-2}$] = **11.2**

$E_{dispervise}$ $^{empirical}$ [meV·Å$^{-2}$] = **7.45**

**Notes**:

Crystal structure is given in ref.[243] Band gap is reported in ref.[244] The electronic dielectric constant is approximated from the reflectance spectrum reported by Pollini *et al*.[245] Anisotropy is ignored.

# Crystal no. 27. $Cr_2Ge_2Te_6$

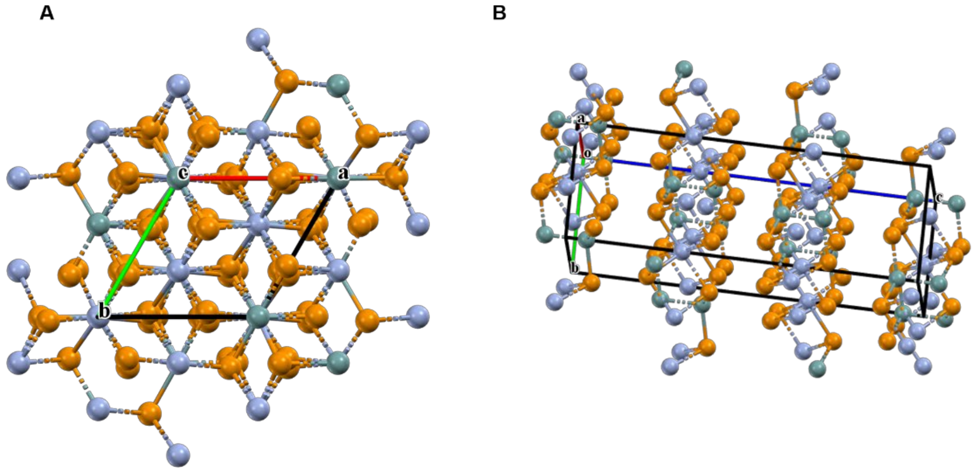


**Fig. S50. Visualization of the packed crystal structure of $Cr_2Ge_2Te_6$.** A) The packed crystal is visualized along c*. B) To better illustrate the 3D structure, packed cell is visualized employing free-style orientation.

Space group: R -3 (148)

Crystal system: Trigonal (Hexagonal axes)

Cell parameters: a= **6.80900 Å**, α= **20.44390º**

Density [g·cm$^{-3}$] = 6.15800

Hamaker Constant [eV] = **1.69**

Molecular Mass [g·mol$^{-1}$] = 1014.9

$f_{Pauling}$ = **0.025**

Band gap [eV] = 0.74

$$\varepsilon_\infty = \begin{pmatrix} 16.7 & & \\ & 16.7 & \\ & & N/A \end{pmatrix}$$

$E_{total}^{\text{theory (RPA)}}$ [meV·Å$^{-2}$] = **N/A**

$E_{total}^{\text{theory (corrected rVV10)}}$ [meV·Å$^{-2}$] = **19.14**

N/A

$E_{total}^{\text{empirical}}$ [meV·Å$^{-2}$] = **16.6**

$E_{dispervise}^{\text{empirical}}$ [meV·Å$^{-2}$] = **16.2**

**Notes**:

Crystal structure is given in ref.[246] Band gap is reported in ref.[247] The electronic dielectric constant is estimated using the empirical relation, Eq. (S1). Anisotropy is ignored. Ionicity is the average value of the ionicity of Cr-Te and Ge-Te bonds.

# Crystal no. 28. $CrBr_3$

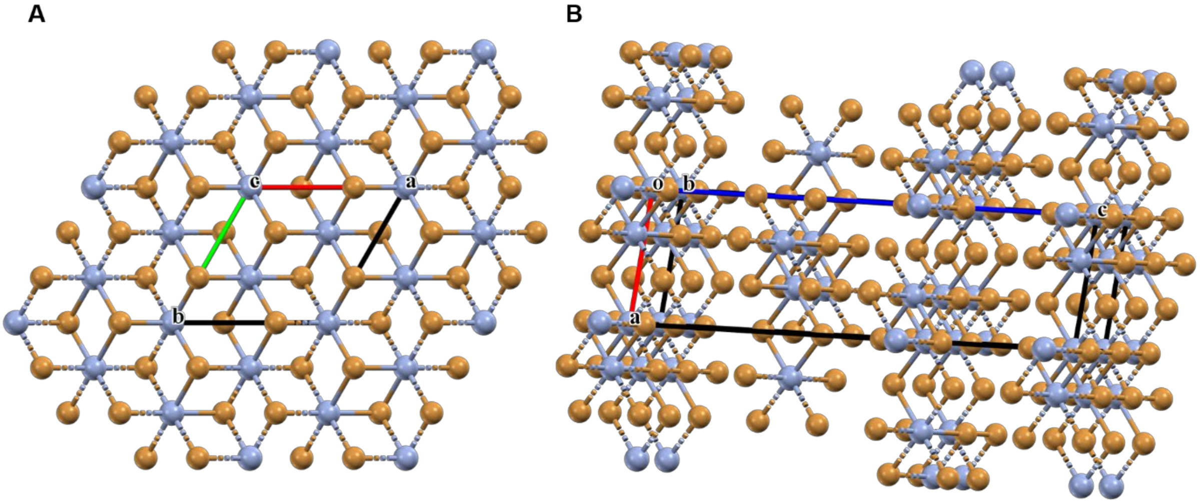


**Fig. S51. Visualization of the packed crystal structure of $CrBr_3$.** A) The packed crystal is visualized along c*. B) To better illustrate the 3D structure, packed cell is visualized employing free-style orientation.

Space group: P 3 (143)

Crystal system: Trigonal (Hexagonal axes)

Cell parameters: a= **6.26000 Å**, c= **18.20000**

Density [g·cm$^{-3}$] = 4.70500

Hamaker Constant [eV] = **0.95**

Molecular Mass [g·mol$^{-1}$] = 291.7

$f_{Pauling}$ = **0.345**

Band gap [eV] = 2.9

$$\varepsilon_\infty = \begin{pmatrix} 4.7 & & \\ & 4.7 & \\ & & 3.2 \end{pmatrix}$$

$E_{total}$ $^{theory\ (RPA)}$ [meV·Å$^{-2}$] = **N/A**
$E_{total}$ $^{theory\ (corrected\ rVV10)}$ [meV·Å$^{-2}$] = **14.06**

$$\varepsilon(0) = \begin{pmatrix} 6.3 & & \\ & 6.3 & \\ & & 3.3 \end{pmatrix}$$

$E_{total}$ $^{empirical}$ [meV·Å$^{-2}$] = **13.9**

$E_{dispervise}$ $^{empirical}$ [meV·Å$^{-2}$] = **9.1**

**Notes**:
Crystal Structure is given in ref.[248] Band gap and in-plane electronic dielectric constant are estimated from the reflectivity spectra at room temperature reported in ref.[249] The in-plane static dielectric constant is estimated from the infrared oscillator's strength of the absorption band at 32.5 meV, reported by Borghesi *et al.*[55] The out-of-plane dielectric constants are theoretical values reported in Materials Project webpage.[54]

# Crystal no. 29. $CrCl_3$

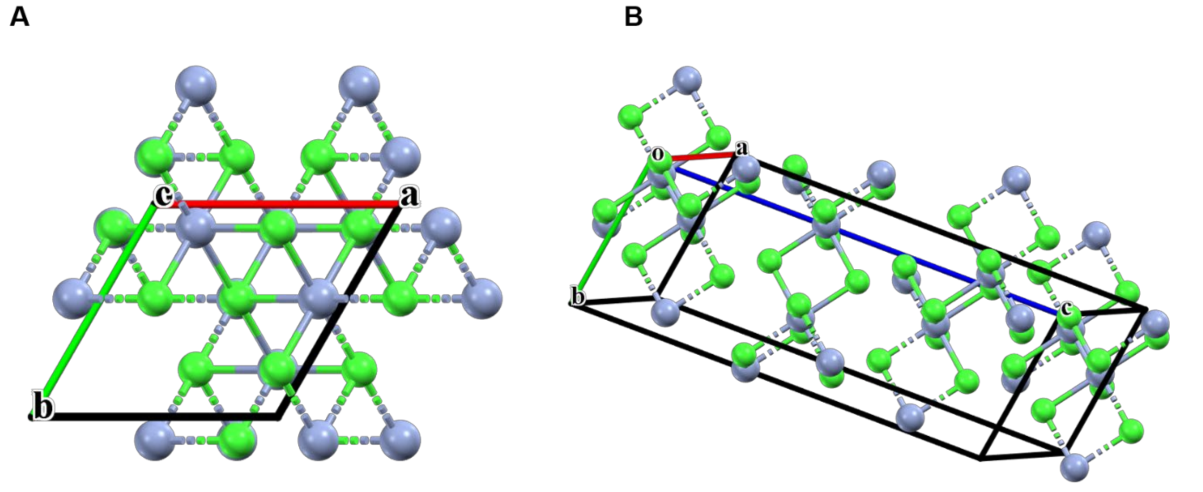


**Fig. S52. Visualization of the packed crystal structure of $CrCl_3$.** A) The packed crystal is visualized along c*. B) To better illustrate the 3D structure, packed cell is visualized employing free-style orientation.

Space group: P 32 1 2 (153)

Crystal system: Trigonal (Hexagonal axes)

Cell parameters: a= **6.01700 Å**, c= **17.30000 Å**

Density [g·cm$^{-3}$] = 2.90800

Hamaker Constant [eV] = **0.74**

Molecular Mass [g·mol$^{-1}$] = 158.4

$f_{Pauling}$ = **0.43**

Band gap [eV] = 3.2

$$\varepsilon_\infty = \begin{pmatrix} 3.6 & & \\ & 3.6 & \\ & & 2.53 \end{pmatrix}$$

$E_{total}^{\text{theory (RPA)}}$ [meV·Å$^{-2}$] = **N/A**

$E_{total}^{\text{theory (corrected rVV10)}}$ [meV·Å$^{-2}$] = **9.83**

$$\varepsilon(0) = \begin{pmatrix} 5.3 & & \\ & 5.3 & \\ & & N/A \end{pmatrix}$$

$E_{total}^{\text{empirical}}$ [meV·Å$^{-2}$] = **12.5**

$E_{dispervise}^{\text{empirical}}$ [meV·Å$^{-2}$] = **7.1**

**Notes**:

Crystal structure is given in ref.[250] Full spectrum of dielectric function (in-plane) is used. Details are given in section 12.

# Crystal no. 30. CrOCl

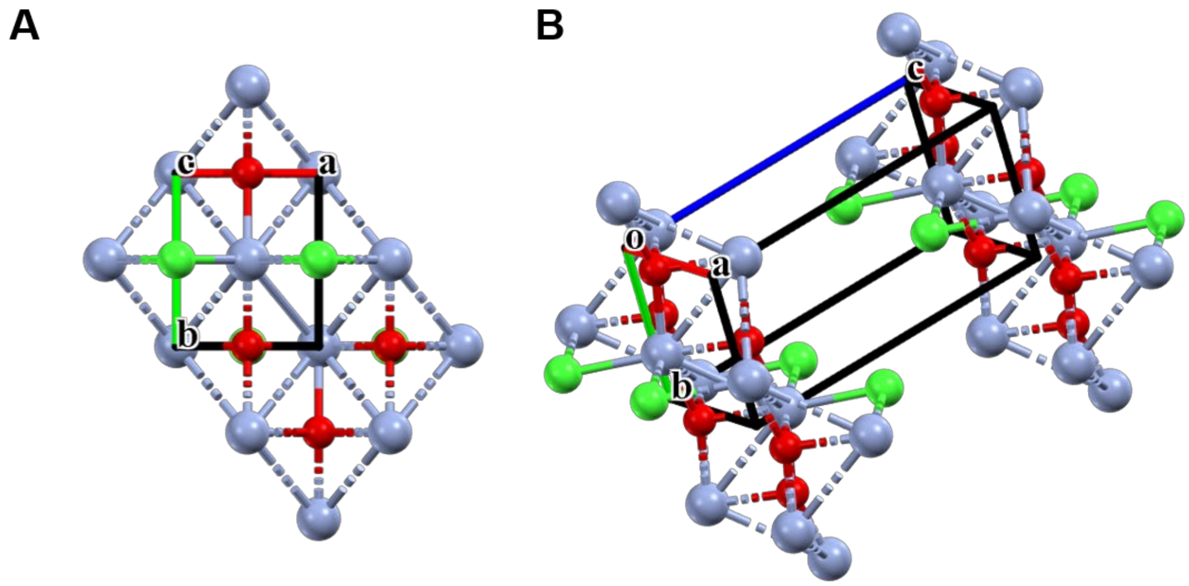


**Fig. S53. Visualization of the packed crystal structure of CrOCl.** A) The crystal is visualized along c*. B) To better illustrate the 3D structure, packed cell is visualized employing free-style orientation.

Density [g·cm$^{-3}$] = 4.11

Molecular Mass [g·mol$^{-1}$] = 103.45

Band gap [eV] ≈ 3

$$\varepsilon_\infty = \begin{pmatrix} 4 & & \\ & 4 & \\ & & N/A \end{pmatrix}$$

N/A

Hamaker Constant [eV] = **0.89**

$f_{Pauling}$ = **0.489**

$E_{total}$ $^{theory\ (RPA)}$ [meV·Å$^{-2}$] = **N/A**
$E_{total}$ $^{theory\ (corrected\ rVV10)}$ [meV·Å$^{-2}$] = **14.65**

$E_{total}$ $^{empirical}$ [meV·Å$^{-2}$] = **16.7**

$E_{dispervise}$ $^{empirical}$ [meV·Å$^{-2}$] = **8.5**

**Notes**:
Density is calculated form the XRD data in ref.[251] Optical band gap is an approximation based on the transmission spectra reported in refs.[252, 253] In both reports, two bands at around 2 and 2.8 eV are reported which could be due to the d-d transitions. In our opinion, the fundamental absorption edge is about 3 eV. The electronic dielectric constant is approximated from the reported refractive indices in ref.[253] Anisotropy is ignored. Ionicity is an average value of Cr-O and Cr-Cl bond.

# Crystal no. 31. $CrPS_4$

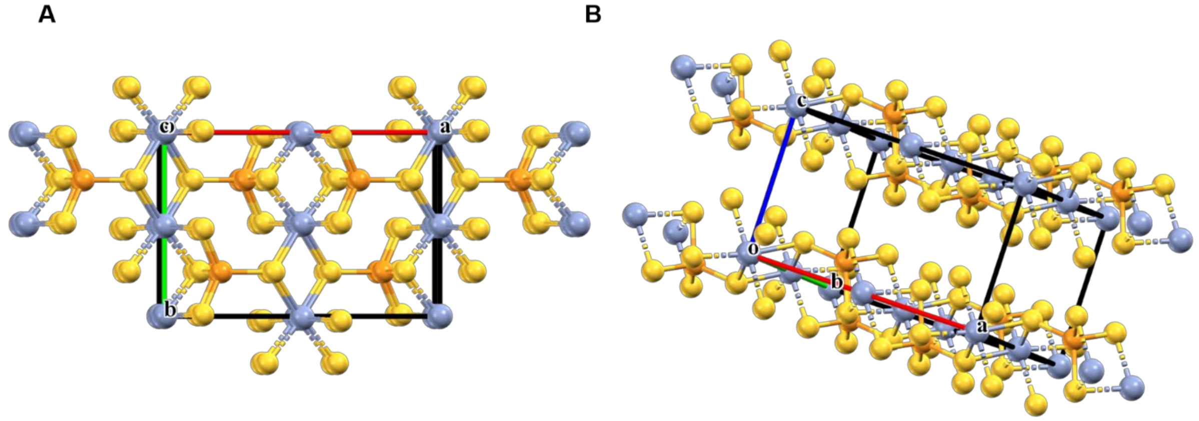


**Fig. S54. Visualization of the packed crystal structure of $CrPS_4$.** A) The packed crystal is visualized along c*. B) To better illustrate the 3D structure, packed cell is visualized employing free-style orientation.

Space group: C 1 2 1 (5)

Crystal system: Monoclinic

Cell parameters: a= **10.87100 Å**, b= **7.25400 Å, c= 6.14000 Å, β= 91.880 º**

Density [g·cm$^{-3}$] = 2.89900

Hamaker Constant [eV] = **1.40**

Molecular Mass [g·mol$^{-1}$] = 211.2

$f_{Pauling}$ = **0.18**

Band gap [eV] = 1.35

$$\varepsilon_{\infty} = \begin{pmatrix} 10.3 & & \\ & 10.3 & \\ & & N/A \end{pmatrix}$$

E$_{total}$ $^{theory\ (RPA)}$ [meV·Å$^{-2}$] = **N/A**

E$_{total}$ $^{theory\ (corrected\ rVV10)}$ [meV·Å$^{-2}$] = **15.31**

N/A

E$_{total}$ $^{empirical}$ [meV·Å$^{-2}$] = **16.5**

E$_{dispervise}$ $^{empirical}$ [meV·Å$^{-2}$] = **13.5**

**Notes**:
Crystal structure is given ref.[254] Band gap is reported in ref.[255] Electronic dielectric constant is approximated using the Eqs. (7) and (S18). Anisotropy is ignored. Ionicity is for Cr-S bond.

# Crystal no. 32. CrSBr

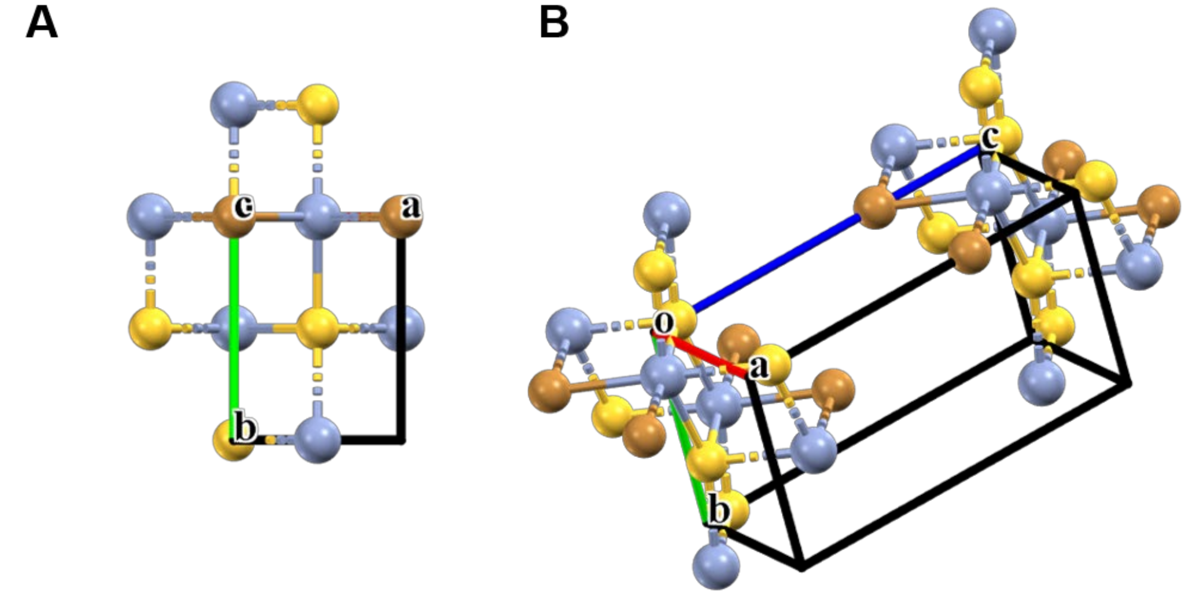


**Fig. S55. Visualization of the packed crystal structure of CrSBr.** A) The crystal is visualized along c*. B) To better illustrate the 3D structure, packed cell is visualized employing free-style orientation.

Density [g·cm$^{-3}$] = 4.1

Molecular Mass [g·mol$^{-1}$] = 164.0

Band gap [eV] = 1.25

$$\varepsilon_{\infty} = \begin{pmatrix} 11.1 & & \\ & 11.1 & \\ & & N/A \end{pmatrix}$$

N/A

Hamaker Constant [eV] = **1.58**

$f_{Pauling}$ = **0.268**

$E_{total}$ $^{theory\ (RPA)}$ [meV·Å$^{-2}$] = **N/A**
$E_{total}$ $^{theory\ (corrected\ rVV10)}$ [meV·Å$^{-2}$] = **17.95**

$E_{total}$ $^{empirical}$ [meV·Å$^{-2}$] = **20.8**

$E_{dispervise}$ $^{empirical}$ [meV·Å$^{-2}$] = **15.2**

**Notes**:
Density is estimated from the XRD data in ref.[256] Band gap is reported in ref.[257] Electronic dielectric constant is approximated using the Eqs. (7) and (S18). Anisotropy is ignored. Ionicity is the average value for Cr-S and Cr-Br bonds.

# Crystal no. 33. $Cs_3Bi_2I_9$

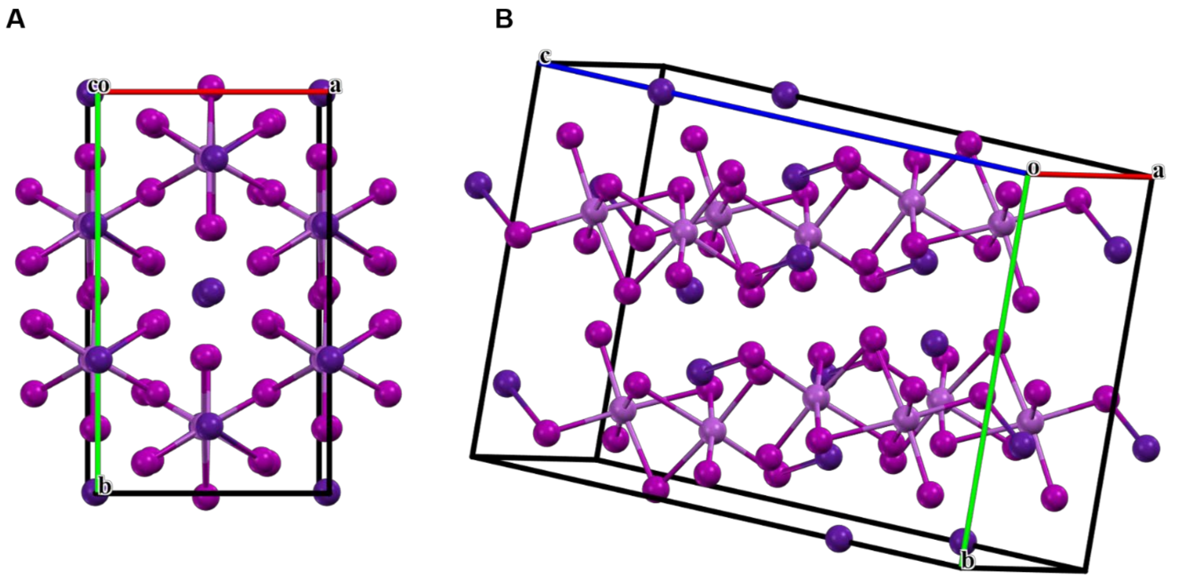


**Fig. S56. Visualization of the packed crystal structure of $Cs_3Bi_2I_9$.** A) The packed crystal is visualized along c*. B) To better illustrate the 3D structure, packed cell is visualized employing free-style orientation.

Space group: C 1 2/c 1 (15) — Crystal system: Monoclinic

Cell parameters: a= **8.34600 Å**, b= **14.47200 Å**, c= **21.10000 Å**, β= **91.000 º**

Density [g·cm$^{-3}$] = 5.10600 — Hamaker Constant [eV] = **0.95**

Molecular Mass [g·mol$^{-1}$] = 1958.8 — $f_{Pauling}$ = **0.34**

Band gap [eV] = 2

$$\varepsilon_\infty = \begin{pmatrix} 5.05 & & \\ & 5.05 & \\ & & N/A \end{pmatrix}$$

$E_{total}^{\text{theory (RPA)}}$ [meV·Å$^{-2}$] = **N/A**
$E_{total}^{\text{theory (corrected rVV10)}}$ [meV·Å$^{-2}$] = **N/A**

N/A — $E_{total}^{\text{empirical}}$ [meV·Å$^{-2}$] = **13.8**

$E_{dispervise}^{\text{empirical}}$ [meV·Å$^{-2}$] = **9.1**

**Notes**:
Crystal Structure is given in ref.[258] Band gap is reported in ref.[259] Electronic dielectric constant is approximated using the Eqs. (7) and (S18). Anisotropy is ignored. Ionicity is the average value for Cs-I and Bi-I bonds.

# Crystal no. 34. $FeBr_2$

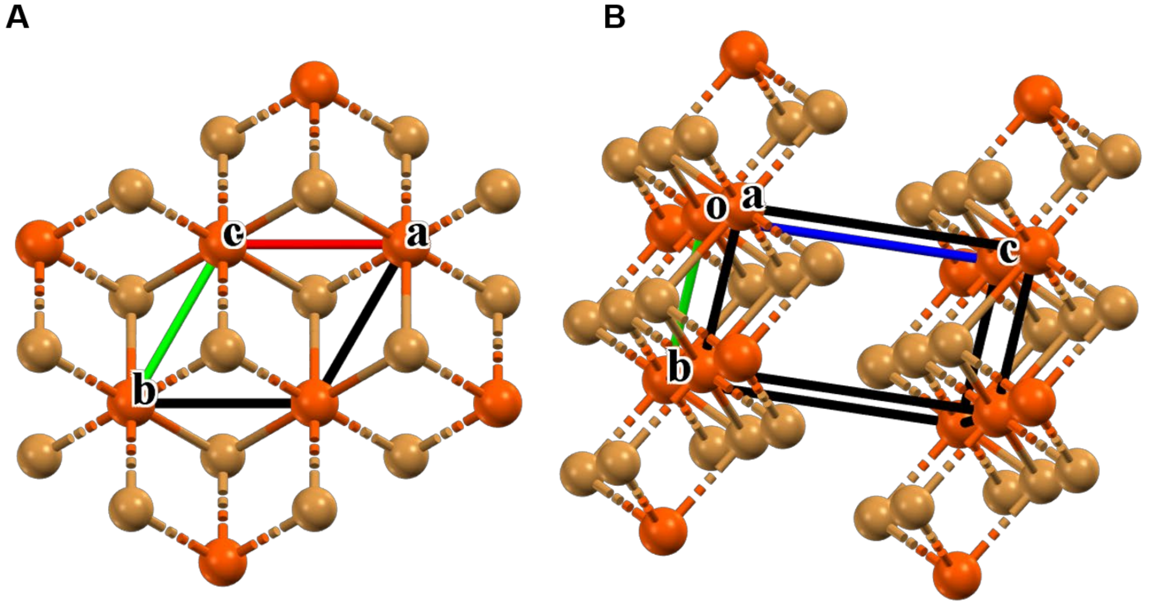


**Fig. S57. Visualization of the packed crystal structure of $FeBr_2$.** A) The packed crystal is visualized along c*. B) To better illustrate the 3D structure, packed cell is visualized employing free-style orientation.

Space group: P -3 m 1 (164)

Crystal system: Trigonal (Hexagonal axes)

Cell parameters: a= **3.77620 Å**, c= **6.22700 Å**

Density [g·cm$^{-3}$] = 4.64700

Hamaker Constant [eV] = **0.96**

Molecular Mass [g·mol$^{-1}$] = 215.7

$f_{Pauling}$ = **0.273**

Band gap [eV] = 3.5

$$\varepsilon_{\infty} = \begin{pmatrix} 3.94 & & \\ & 3.94 & \\ & & N/A \end{pmatrix}$$

$E_{total}^{\text{theory (RPA)}}$ [meV·Å$^{-2}$] = **N/A**

$E_{total}^{\text{theory (corrected rVV10)}}$ [meV·Å$^{-2}$] = **14.65**

N/A

$E_{total}^{\text{empirical}}$ [meV·Å$^{-2}$] = **12.7**

$E_{dispervise}^{\text{empirical}}$ [meV·Å$^{-2}$] = **9.25**

**Notes**:

Crystal structure is given in ref.[260] Band gap is reported in ref.[244] Electronic dielectric constant is given in ref.[261] Anisotropy is ignored.

# Crystal no. 35. $FeCl_2$

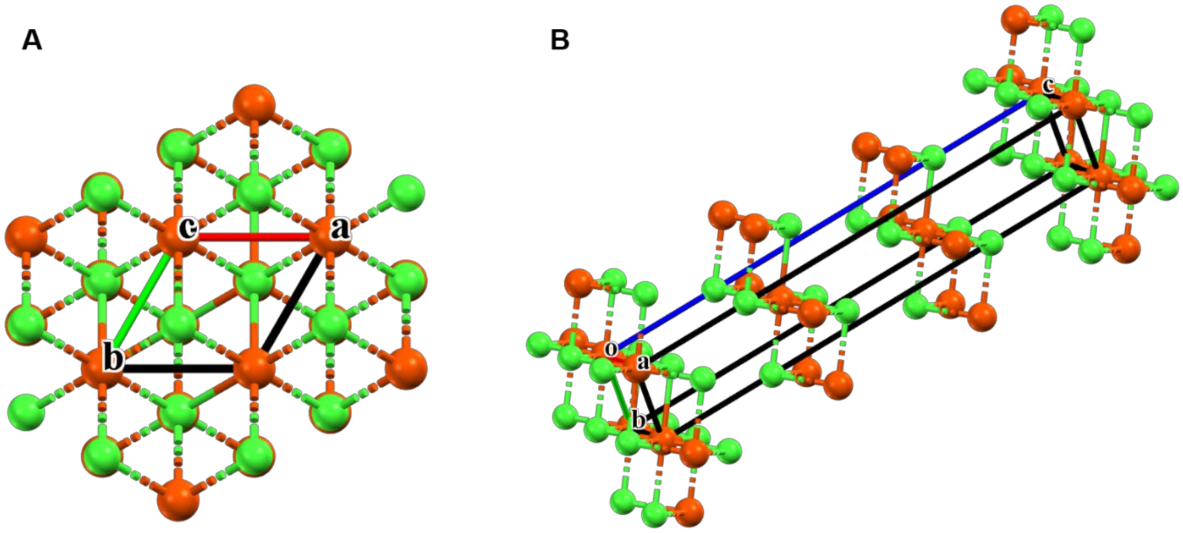


**Fig. S58. Visualization of the packed crystal structure of $FeCl_2$.** A) The packed crystal is visualized along c*. B) To better illustrate the 3D structure, packed cell is visualized employing free-style orientation.

Space group: R -3 m (166)

Crystal system: Trigonal (Hexagonal axes)

Cell parameters: a= **3.57900 Å**, c= **17.53600 Å**

Density [g·cm$^{-3}$] = 3.24600

Hamaker Constant [eV] = **0.81**

Molecular Mass [g·mol$^{-1}$] = 126.8

$f_{Pauling}$ = **0.357**

Band gap [eV] = 4

$$\varepsilon_{\infty} = \begin{pmatrix} 3.4 & & \\ & 3.4 & \\ & & N/A \end{pmatrix}$$

$E_{total}^{theory\ (RPA)}$ [meV·Å$^{-2}$] = **N/A**

$E_{total}^{theory\ (corrected\ rVV10)}$ [meV·Å$^{-2}$] = **10.03**

N/A

$E_{total}^{empirical}$ [meV·Å$^{-2}$] = **12.05**

$E_{dispervise}^{empirical}$ [meV·Å$^{-2}$] = **7.75**

**Notes**:

Crystal structure is given in ref.[262] Band gap is reported in ref.[244] Electronic dielectric constant is given in ref.[261] Anisotropy is ignored.

# Crystal no. 36. $FeI_2$

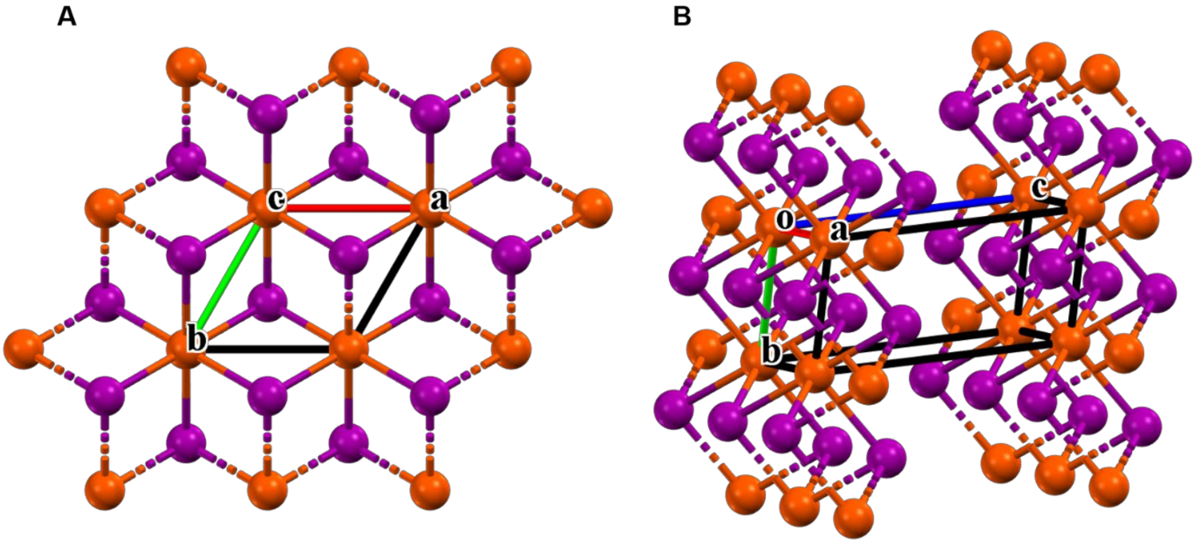


**Fig. S59. Visualization of the packed crystal structure of $FeI_2$.** A) The packed crystal is visualized along c*. B) To better illustrate the 3D structure, packed cell is visualized employing free-style orientation.

Space group: P -3 m 1 (164)

Crystal system: Trigonal (Hexagonal axes)

Cell parameters: a= **4.04000 Å, c= 6.75000 Å**

Density [g·cm$^{-3}$] = 5.28900

Hamaker Constant [eV] = **0.83**

Molecular Mass [g·mol$^{-1}$] = 309.7

$f_{Pauling}$ = **0.158**

Band gap [eV] = 3

$$\varepsilon_\infty = \begin{pmatrix} 3.81 & & \\ & 3.81 & \\ & & N/A \end{pmatrix}$$

N/A

$E_{total}$ $^{theory\ (RPA)}$ [meV·Å$^{-2}$] = **N/A**
$E_{total}$ $^{theory\ (corrected\ rVV10)}$ [meV·Å$^{-2}$] = **15.38**

$E_{total}$ $^{empirical}$ [meV·Å$^{-2}$] = **9.5**

$E_{dispervise}$ $^{empirical}$ [meV·Å$^{-2}$] = **8.0**

**Notes**:
Crystal structure is given in ref.[262] Band gap and electronic dielectric constant are taken from ref.[263] Anisotropy is ignored.

# Crystal no. 37. FeOCl

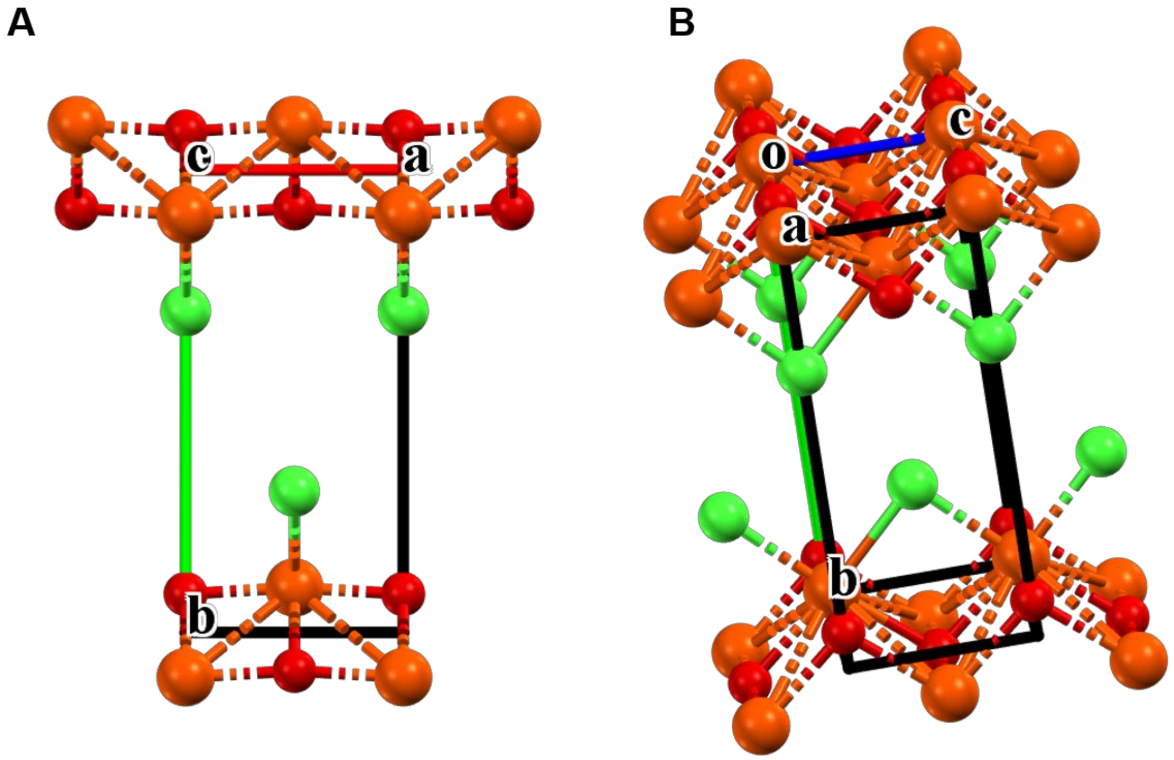


**Fig. S60. Visualization of the packed crystal structure of FeOCl.** A) The packed crystal is visualized along c*. B) To better illustrate the 3D structure, packed cell is visualized employing free-style orientation.

Space group: P m n m (59)

Crystal system: Orthorhombic

Cell parameters: a= **3.75000 Å**, b= **7.95000 Å,** c= **3.30000 Å**

Density [g·cm$^{-3}$] = 3.62200

Hamaker Constant [eV] = **0.79**

Molecular Mass [g·mol$^{-1}$] = 107.3

$f_{Pauling}$ = **0.417**

Band gap [eV] = 2.0

$$\varepsilon_\infty = \begin{pmatrix} 4.05 & & \\ & 3.21 & \\ & & 5.19 \end{pmatrix}$$

$E_{total}$ $^{theory\ (RPA)}$ [meV·Å$^{-2}$] = **N/A**

$E_{total}$ $^{theory\ (corrected\ rVV10)}$ [meV·Å$^{-2}$] = **15.05**

N/A

$E_{total}$ $^{empirical}$ [meV·Å$^{-2}$] = **13.04**

$E_{dispervise}$ $^{empirical}$ [meV·Å$^{-2}$] = **7.60**

**Notes**:
Crystal structure is given in ref.[264] Band gap is reported in ref.[265]. Dielectric constants are the theoretical values.[50] Infrared bands were ignored in calculation of the Hamaker constant.

# Crystal no. 38. $Fe_2P_2S_6$

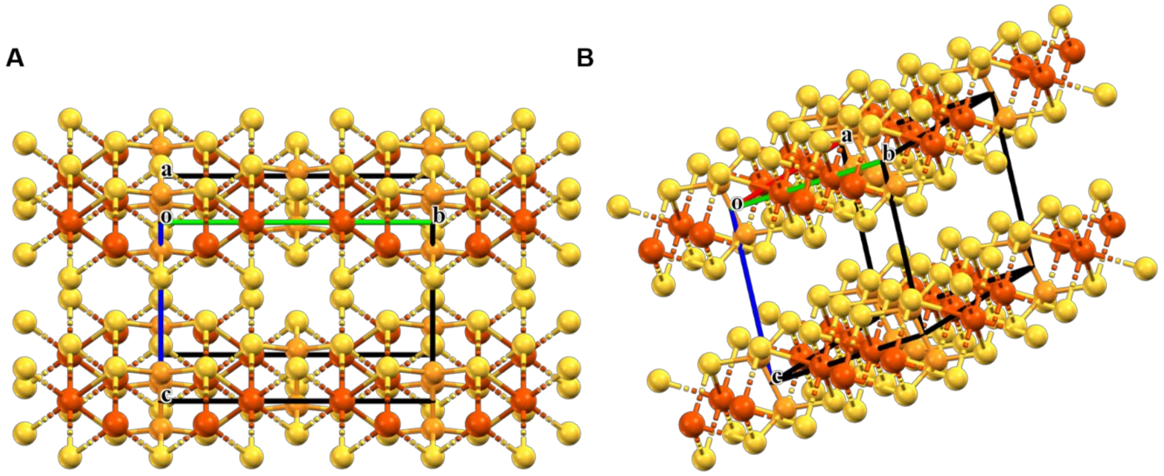


**Fig. S61. Visualization of the packed crystal structure of $Fe_2P_2S_6$.** A) The packed crystal is visualized along a*. B) To better illustrate the 3D structure, packed cell is visualized employing free-style orientation.

Space group: C 1 2/m 1 (12)

Crystal system: Monoclinic

Cell parameters: a= **5.93400 Å**, b= **10.28000 Å**, c= **6.77200 Å**, β= **107.200 º**

Density [g·cm$^{-3}$] = 3.08000

Hamaker Constant [eV] = **1.62**

Molecular Mass [g·mol$^{-1}$] = 183.0

$f_{Pauling}$ = **0.131**

Band gap [eV] = 1.6

$$\varepsilon_\infty = \begin{pmatrix} 9.8 & & \\ & 9.8 & \\ & & N/A \end{pmatrix}$$

$E_{total}$ $^{theory\ (RPA)}$ [meV·Å$^{-2}$] = **N/A**

$E_{total}$ $^{theory\ (corrected\ rVV10)}$ [meV·Å$^{-2}$] = **18.74**

N/A

$E_{total}$ $^{empirical}$ [meV·Å$^{-2}$] = **17.9**

$E_{dispervise}$ $^{empirical}$ [meV·Å$^{-2}$] = **15.6**

**Notes**:

Crystal structure is given in ref.[266] Band gap is reported in ref.[267] Electronic dielectric constant is approximated using the Eqs. (7) and (S18). Anisotropy is ignored. Ionicity is for Fe-S bond.

# Crystal no. 39. $Fe_2P_2Se_6$

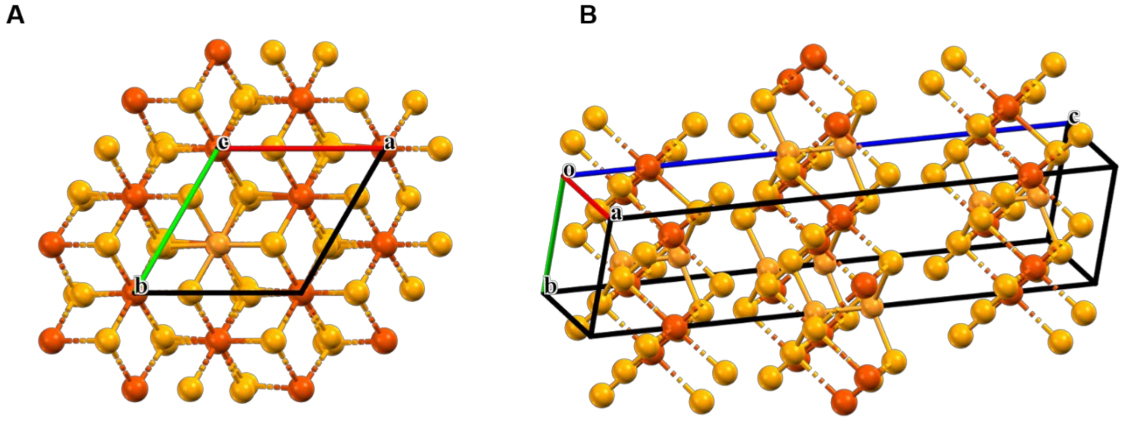


**Fig. S62. Visualization of the packed crystal structure of $Fe_2P_2Se_6$.** A) The packed crystal is visualized along c*. B) To better illustrate the 3D structure, packed cell is visualized employing free-style orientation.

Space group: R 3 (146)

Crystal system: Trigonal (Hexagonal axes)

Cell parameters: a= **6.26500 Å**, c= **19.80000 Å**

Density [g·cm$^{-3}$] = 4.79100

Hamaker Constant [eV] = **1.84**

Molecular Mass [g·mol$^{-1}$] = 647.4

$f_{Pauling}$ = **0.122**

Band gap [eV] = 1.3

$$\varepsilon_\infty = \begin{pmatrix} 12.8 & & \\ & 12.8 & \\ & & N/A \end{pmatrix}$$

$E_{total}$ $^{theory\ (RPA)}$ [meV·Å$^{-2}$] = **N/A**

$E_{total}$ $^{theory\ (corrected\ rVV10)}$ [meV·Å$^{-2}$] = **19.60**

N/A

$E_{total}$ $^{empirical}$ [meV·Å$^{-2}$] = **20.2**

$E_{dispervise}$ $^{empirical}$ [meV·Å$^{-2}$] = **17.7**

**Notes**:
Crystal structure is given in ref.[266] Band gap is reported in ref.[268] Electronic dielectric constant is approximated using the Eqs. (7) and (S18). Anisotropy is ignored. Ionicity is for Fe-Se bond.

# Crystal no. 40. GaGeTe

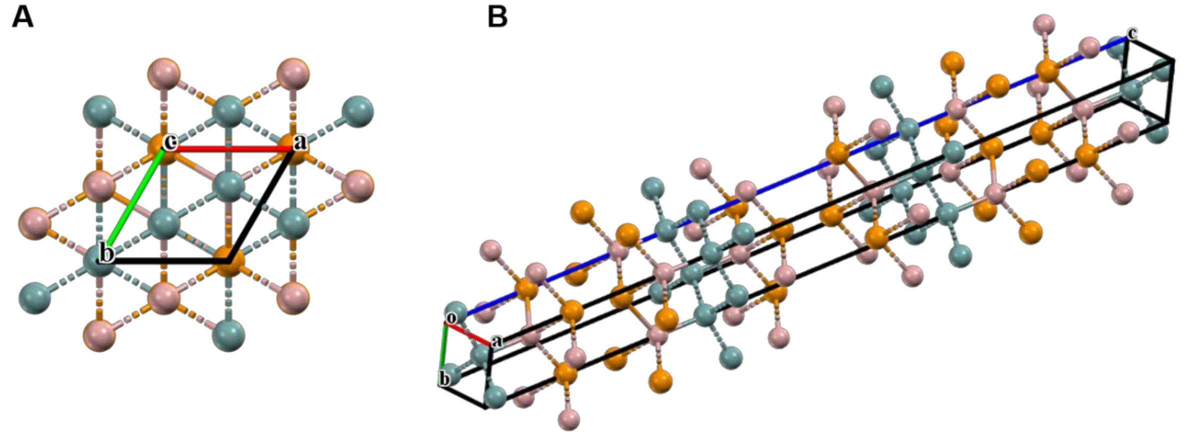


**Fig. S63. Visualization of the packed crystal structure of GaGeTe.** A) The crystal is visualized along c*. B) To better illustrate the 3D structure, packed cell is visualized employing free-style orientation.

Density [g·cm$^{-3}$] = 5.40

Molecular Mass [g·mol$^{-1}$] = 267

Band gap [eV] = 1.12

$$\varepsilon_{\infty} = \begin{pmatrix} 13.3 & & \\ & 13.3 & \\ & & N/A \end{pmatrix}$$

N/A

Hamaker Constant [eV] = **1.78**

$f_{Pauling}$ = **0.021**

$E_{total}$ $^{theory\ (RPA)}$ [meV·Å$^{-2}$] = **N/A**
$E_{total}$ $^{theory\ (corrected\ rVV10)}$ [meV·Å$^{-2}$] = **14.52**

$E_{total}$ $^{empirical}$ [meV·Å$^{-2}$] = **17.5**

$E_{dispervise}$ $^{empirical}$ [meV·Å$^{-2}$] = **17.1**

**Notes**:
Density is calculated form the XRD data is ref.[269] Band gap and electronic dielectric constant are reported in ref.[270] A Drude band with plasma and scattering frequencies of 620 and 306 meV, respectively. This is equivalent to conductivity of 17 S·cm$^{-1}$ which is consistent with the measurements in ref.[271] Ionicity is the ionicity of Ga-Te bond.

# Crystal no. 41. GaS

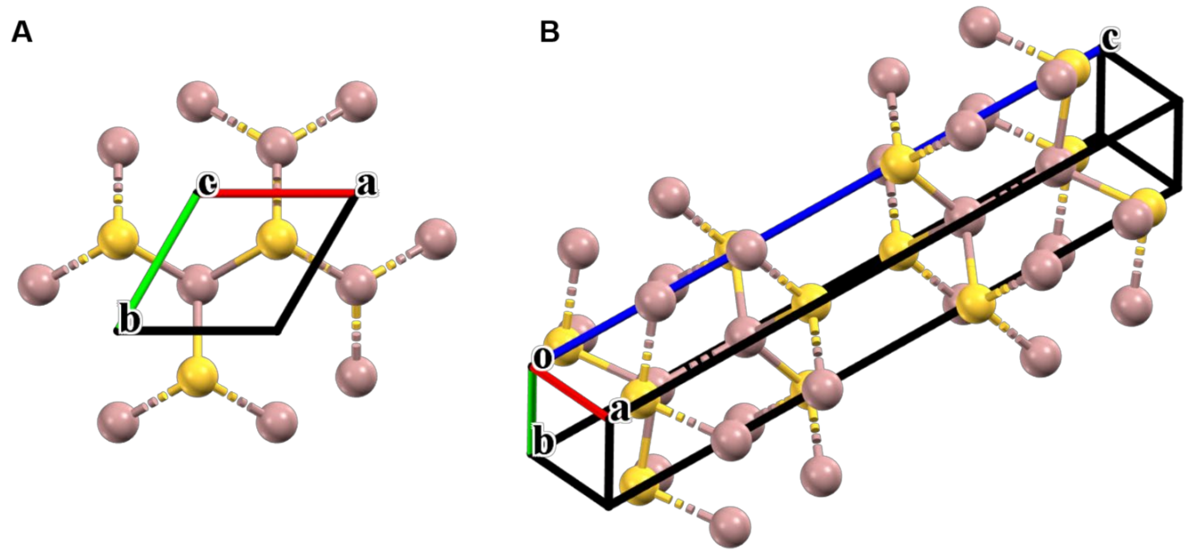


**Fig. S64. Visualization of the packed crystal structure of GaS.** A) The packed crystal is visualized along c*. B) To better illustrate the 3D structure, packed cell is visualized employing free-style orientation.

Space group: P 63/m m c (194)

Crystal system: Hexagonal

Cell parameters: a=**3.58500 Å**, c= **15.50000 Å**

Density [g·cm$^{-3}$] = 3.91900

Hamaker Constant [eV] = **1.39**

Molecular Mass [g·mol$^{-1}$] = 101.8

$f_{Pauling}$ = **0.138**

Band gap [eV] = 2.6

$$\varepsilon_{\infty} = \begin{pmatrix} 6.5 & & \\ & 6.5 & \\ & & 4.05 \end{pmatrix}$$

$E_{total}^{theory\ (RPA)}$ [meV·Å$^{-2}$] = **N/A**

$E_{total}^{theory\ (corrected\ rVV10)}$ [meV·Å$^{-2}$] = **13.60**

$$\varepsilon(0) = \begin{pmatrix} 9.1 & & \\ & 9.1 & \\ & & 4.4 \end{pmatrix}$$

$E_{total}^{empirical}$ [meV·Å$^{-2}$] = **15.5**

$E_{dispervise}^{empirical}$ [meV·Å$^{-2}$] = **13.3**

**Notes**:
Crystal structure is given in ref.[272] Band gap is given in ref.[237] Electronic dielectric constants are estimated from the refractive indices measured in ref.[273] Static dielectric constants are given in ref.[274]

# Crystal no. 42. GaSe

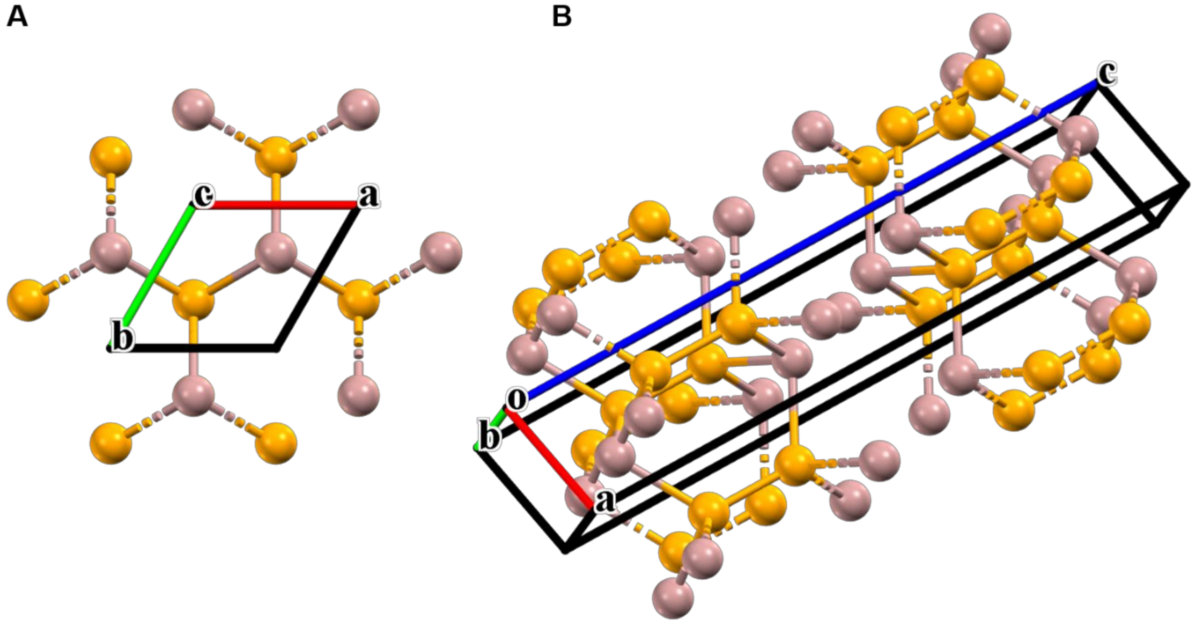


**Fig. S65. Visualization of the packed crystal structure of GaSe.** A) The packed crystal is visualized along c*. B) To better illustrate the 3D structure, packed cell is visualized employing free-style orientation.

Space group: P 63/m m c (194)

Crystal system: Hexagonal

Cell parameters: a=**3.75000 Å**, c= **15.92000 Å**

Density [g·cm$^{-3}$] = 5.09300

Hamaker Constant [eV] = **1.432**

Molecular Mass [g·mol$^{-1}$] = 148.7

$f_{Pauling}$ = **0.128**

Band gap [eV] = 2.1

$$\varepsilon_\infty = \begin{pmatrix} 7.54 & & \\ & 7.54 & \\ & & 5.8 \end{pmatrix}$$

$E_{total}$ $^{theory\ (RPA)}$ [meV·Å$^{-2}$] = **N/A**

$E_{total}$ $^{theory\ (corrected\ rVV10)}$ [meV·Å$^{-2}$] = **13.46**

$$\varepsilon(0) = \begin{pmatrix} 10.68 & & \\ & 10.68 & \\ & & 8.25 \end{pmatrix}$$

$E_{total}$ $^{empirical}$ [meV·Å$^{-2}$] = **15.8**

$E_{dispervise}$ $^{empirical}$ [meV·Å$^{-2}$] = **13.8**

**Notes**:

Crystal structure is given in ref.[275] Dielectric spectra of GaSe in both polarization are given in section 12.

# Crystal no. 43. GaTe

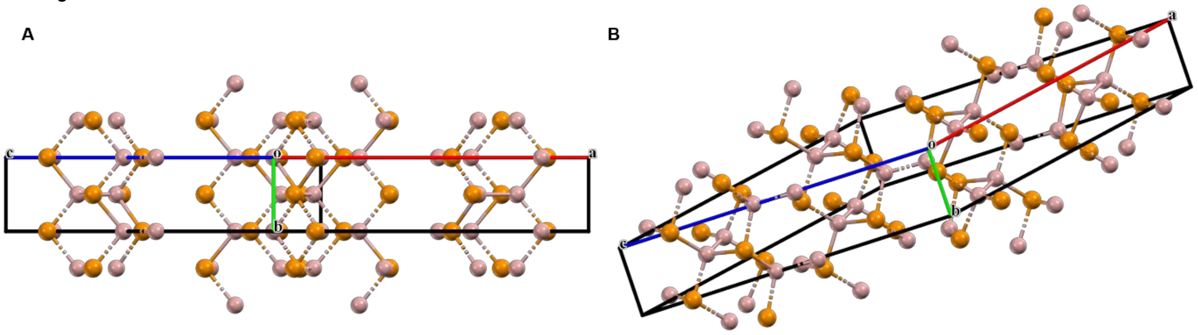


**Fig. S66. Visualization of the packed crystal structure of GaTe.** A) The packed crystal is visualized along c*. B) To better illustrate the 3D structure, packed cell is visualized employing free-style orientation.

Space group: C 1 2/m 1 (12)

Crystal system: Monoclinic

Cell parameters: a=**17.40400 Å**, b= **4.07700 Å** c= **17.93000 Å,** β= **145.610 º**

Density [g·cm$^{-3}$] = 5.47100

Hamaker Constant [eV] = **1.62**

Molecular Mass [g·mol$^{-1}$] = 197.3

$f_{Pauling}$ = **0.021**

Band gap [eV] = 1.6

$$\varepsilon_{\infty} = \begin{pmatrix} 9.3 & & \\ & 9.45 & \\ & & N/A \end{pmatrix}$$

$E_{total}$ $^{theory\ (RPA)}$ [meV·Å$^{-2}$] = **N/A**

$E_{total}$ $^{theory\ (corrected\ rVV10)}$ [meV·Å$^{-2}$] = **13.60**

$$\varepsilon(0) = \begin{pmatrix} 10.8 & & \\ & 15.95 & \\ & & N/A \end{pmatrix}$$

$E_{total}$ $^{empirical}$ [meV·Å$^{-2}$] = **16.0**

$E_{dispervise}$ $^{empirical}$ [meV·Å$^{-2}$] = **15.6**

**Notes**:

Crystal structure is given in ref.[276] Band gap along with the electronic dielectric constants are reported in ref.[277] The static dielectric constant perpendicular to 'b' axis is given in ref.[278] The static dielectric constant parallel to 'b' axis is a theoretical value from the Materials Project webpage.[50] The theoretical binding energy is for the hexagonal polymorph of GaTe.[30]

# Crystal no. 44. GaTeCl

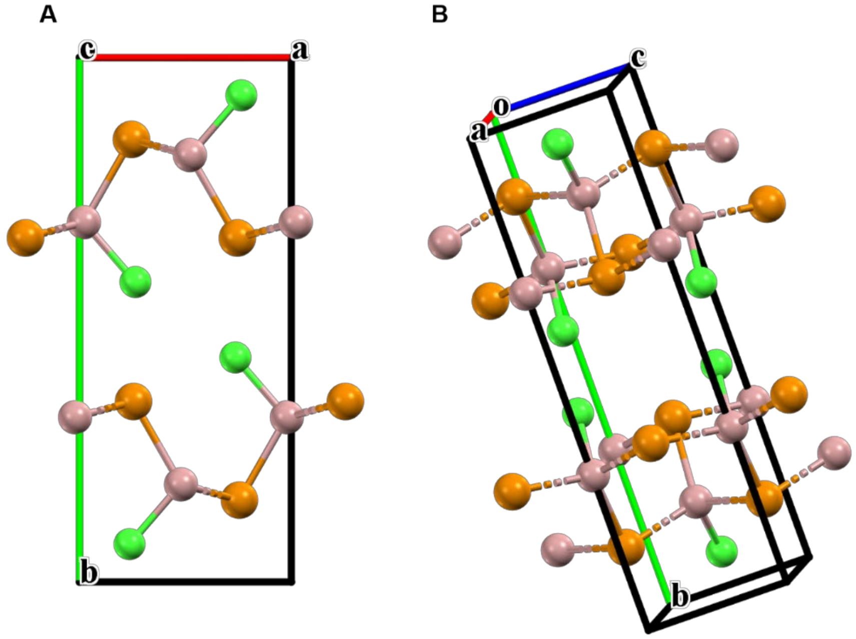


**Fig. S67. Visualization of the packed crystal structure of GaTeCl.** A) The packed crystal is visualized along c*. B) To better illustrate the 3D structure, packed cell is visualized employing

Space group: P n n m (58)

Crystal system: Orthorhombic

Cell parameters: a=**5.85200 Å**, b= **14.46600 Å** c= **4.08200 Å**

Density [g·cm$^{-3}$] = 4.47400

Hamaker Constant [eV] = **1.22**

Molecular Mass [g·mol$^{-1}$] = 232.8

$f_{Pauling}$ = **0.193**

Band gap [eV] = 3.05

$$\varepsilon_\infty = \begin{pmatrix} 5.02 & & \\ & 5.02 & \\ & & N/A \end{pmatrix}$$

$E_{total}$ $^{theory\ (RPA)}$ [meV·Å$^{-2}$] = **N/A**

$E_{total}$ $^{theory\ (corrected\ rVV10)}$ [meV·Å$^{-2}$] = **13.73**

N/A

$E_{total}$ $^{empirical}$ [meV·Å$^{-2}$] = **14.6**

$E_{dispervise}$ $^{empirical}$ [meV·Å$^{-2}$] = **11.8**

**Notes**:

Crystal structure is given in ref.[279] Band gap is reported in ref.[280] Electronic dielectric constant is approximated using the Eqs. (7) and (S18). Anisotropy is ignored. Ionicity is the average value of ionicity of Ga-Te and Ga-Cl bonds.

# Crystal no. 45. GeAs

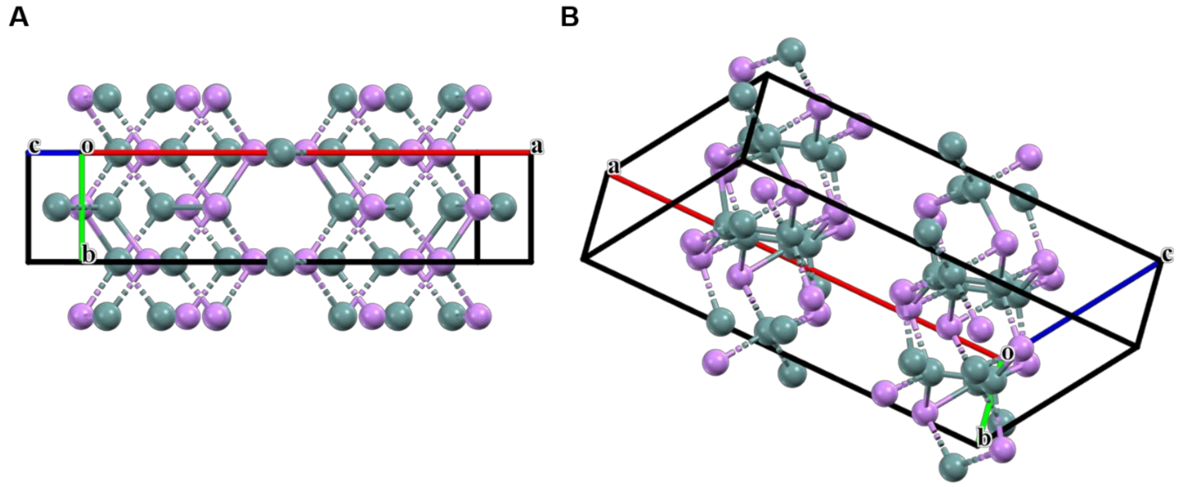


**Fig. S68. Visualization of the packed crystal structure of GeAs.** A) The packed crystal is visualized along c*. B) To better illustrate the 3D structure, packed cell is visualized employing free-style orientation.

Density [g·cm$^{-3}$] = 5.35

Molecular Mass [g·mol$^{-1}$] = 147.6

Band gap [eV] = 0.7

$$\varepsilon_\infty = \begin{pmatrix} 16.8 & & \\ & 15 & \\ & & N/A \end{pmatrix}$$

N/A

Hamaker Constant [eV] = **1.59**

$f_{Pauling}$ = **0.007**

$E_{total}$ $^{theory\ (RPA)}$ [meV·Å$^{-2}$] = **N/A**

$E_{total}$ $^{theory\ (corrected\ rVV10)}$ [meV·Å$^{-2}$] = **19.73**

$E_{total}$ $^{empirical}$ [meV·Å$^{-2}$] = **15.4**

$E_{dispervise}$ $^{empirical}$ [meV·Å$^{-2}$] = **15.3**

**Notes**:
Density is calculated from XRD data in ref.[281] Band gap is reported in ref.[282] Electronic dielectric constants are estimated from the refractive indices reported in ref.[283]

# Crystal no. 46. GeS

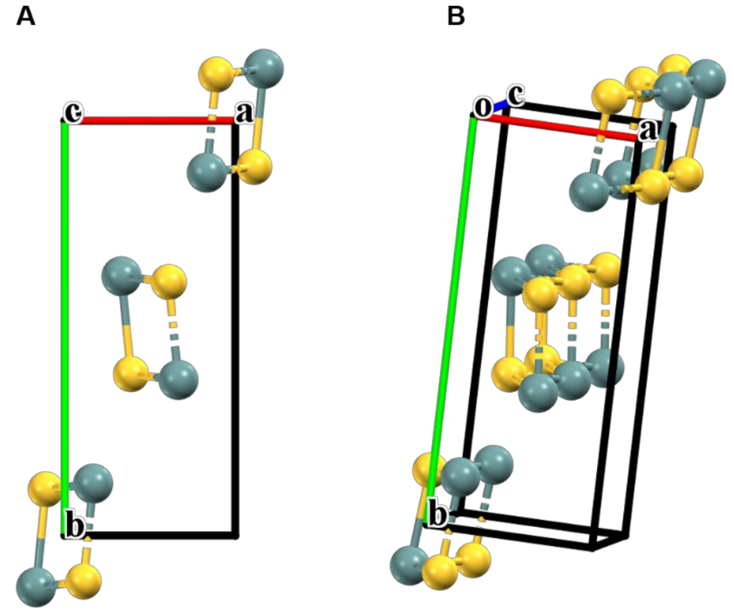


**Fig. S69. Visualization of the packed crystal structure of GeS.** A) The packed crystal is visualized along c*. B) To better illustrate the 3D structure, packed cell is visualized employing free-style orientation.

Space group: P b n m S (-1)

Crystal system: Orthorhombic

Cell parameters: a=**4.29000 Å**, b=**10.42000 Å** c= **3.64000 Å**

Density [g·cm$^{-3}$] = 4.27300

Hamaker Constant [eV] = **1.87**

Molecular Mass [g·mol$^{-1}$] = 104.7

$f_{Pauling}$ = **0.078**

Band gap [eV] = 1.6

$$\varepsilon_\infty = \begin{pmatrix} 11.3 & & \\ & 11.3 & \\ & & 10.1 \end{pmatrix}$$

$E_{total}^{theory\ (RPA)}$ [meV·Å$^{-2}$] = **N/A**

$E_{total}^{theory\ (corrected\ rVV10)}$ [meV·Å$^{-2}$] = **22.97**

N/A

$E_{total}^{empirical}$ [meV·Å$^{-2}$] = **19.6**

$E_{dispervise}^{empirical}$ [meV·Å$^{-2}$] = **18.0**

**Notes**:
Crystal structure is given in ref.[284] Band gap is reported in ref.[285] Electronic dielectric constants are approximated from the reported refractive indices.[286] In-plane anisotropy is ignored.

# Crystal no. 47. GeSe

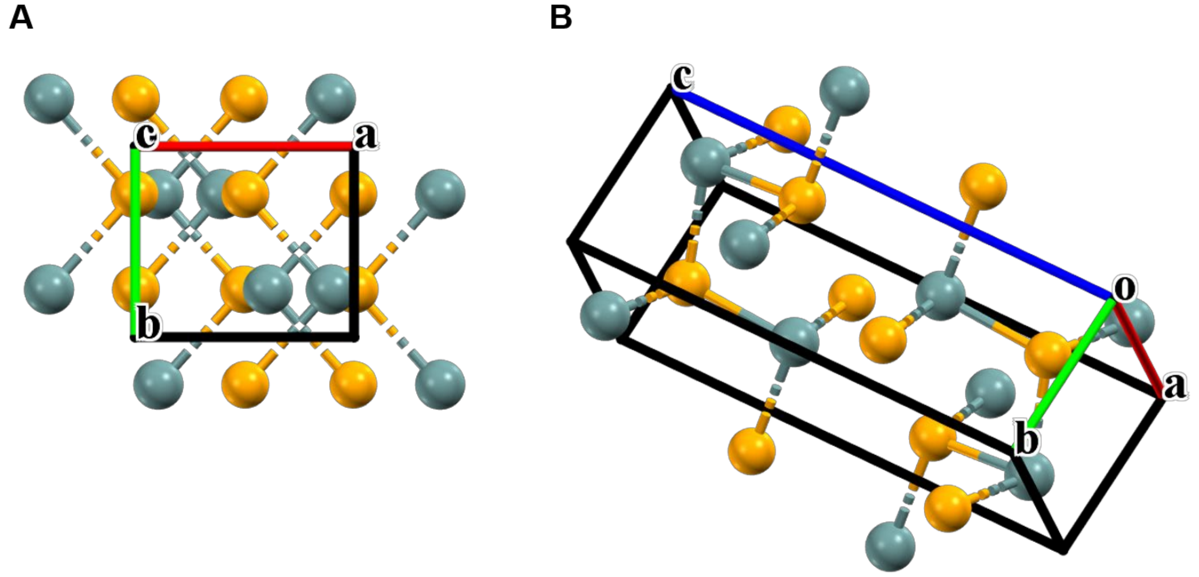


**Fig. S70. Visualization of the packed crystal structure of GeSe.** A) The packed crystal is visualized along c*. B) To better illustrate the 3D structure, packed cell is visualized employing free-style orientation.

Space group: P c m n (62)

Crystal system: Orthorhombic

Cell parameters: a=**4.38000 Å**, b= **3.82000 Å** c= **10.79000 Å**

Density [g·cm$^{-3}$] = 5.57600

Hamaker Constant [eV] = **1.421**

Molecular Mass [g·mol$^{-1}$] = 151.6

$f_{Pauling}$ = **0.07**

Band gap [eV] = 1.53

$$\varepsilon_{\infty} = \begin{pmatrix} 8.4 & & \\ & 8.4 & \\ & & 8.13 \end{pmatrix}$$

$E_{total}$ $^{theory\ (RPA)}$ [meV·Å$^{-2}$] = **N/A**

$E_{total}$ $^{theory\ (corrected\ rVV10)}$ [meV·Å$^{-2}$] = **N/A**

$$\varepsilon(0) = \begin{pmatrix} 19.7 & & \\ & 19.7 & \\ & & N/A \end{pmatrix}$$

$E_{total}$ $^{empirical}$ [meV·Å$^{-2}$] = **14.8**

$E_{dispervise}$ $^{empirical}$ [meV·Å$^{-2}$] = **13.7**

**Notes**:

Crystal structure is given in ref.[287] Band gap is reported in ref.[288] Electronic dielectric constants are reported in ref.[289] In-plane anisotropy is ignored. Static dielectric constants are estimated based on the reflectivity data in ref.[290]

# Crystal no. 48. $HfS_2$

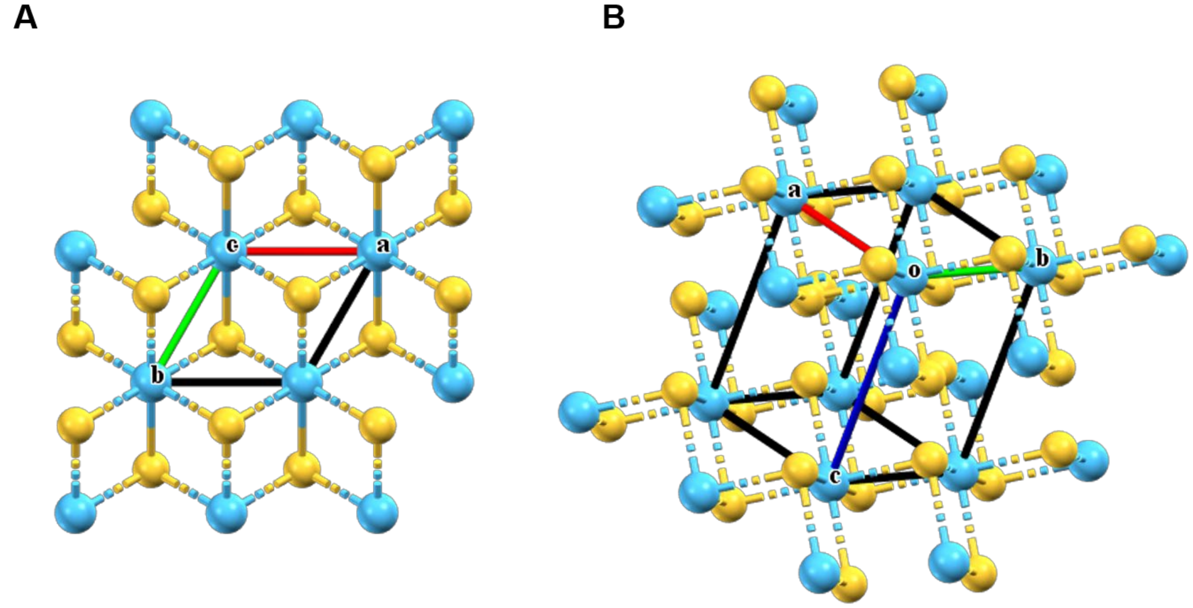


**Fig. S71. Visualization of the packed crystal structure of $HfS_2$.** A) The packed crystal is visualized along c*. B) To better illustrate the 3D structure, packed cell is visualized employing free-style orientation.

Density [g·cm$^{-3}$] = 6.06

Molecular Mass [g·mol$^{-1}$] = 242.6

Band gap [eV] = 2.1

$$\varepsilon_\infty = \begin{pmatrix} 6.2 & & \\ & 6.2 & \\ & & 5.3 \end{pmatrix}$$

$$\varepsilon(0) = \begin{pmatrix} 23.2 & & \\ & 23.2 & \\ & & 7.2 \end{pmatrix}$$

Hamaker Constant [eV] = **1.189**

$f_{Pauling}$ = **0.336**

$E_{total}$ $^{theory\ (RPA)}$ [meV·Å$^{-2}$] = **16.13**
$E_{total}$ $^{theory\ (corrected\ rVV10)}$ [meV·Å$^{-2}$] = **15.31**

$E_{total}$ $^{empirical}$ [meV·Å$^{-2}$] = **17.2**

$E_{dispervise}$ $^{empirical}$ [meV·Å$^{-2}$] = **11.4**

**Notes**:
Density is estimated from XRD data in ref.[291] Band gap is reported in ref.[103] Dielectric constants are reported in ref.[292]

# Crystal no. 49. $HfSe_2$

A B

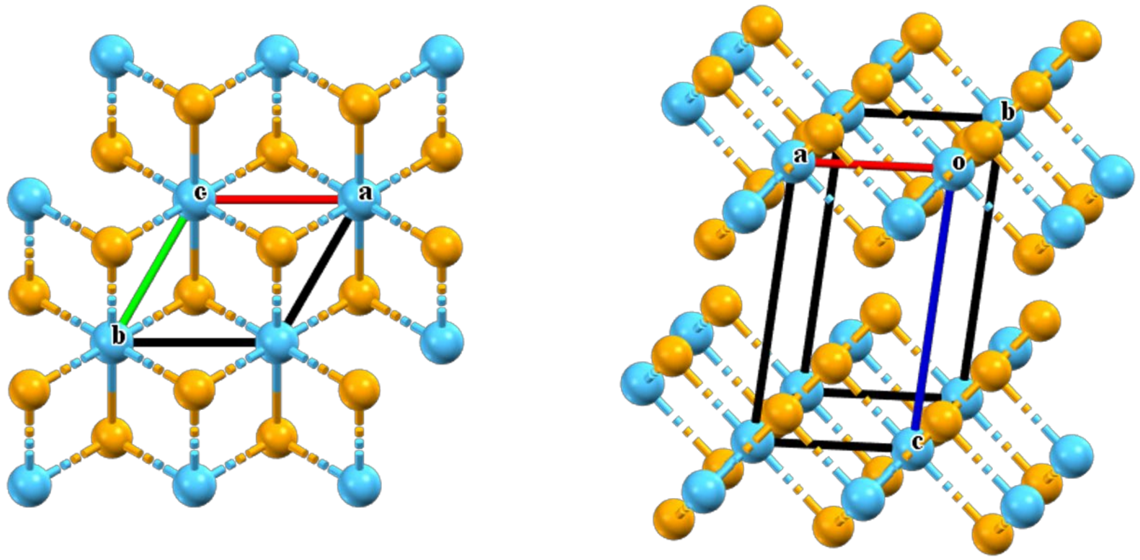


**Fig. S72. Visualization of the packed crystal structure of $HfSe_2$.** A) The packed crystal is visualized along c*. B) To better illustrate the 3D structure, packed cell is visualized employing free-style orientation.

Density [g·cm$^{-3}$] = 7.5

Molecular Mass [g·mol$^{-1}$] = 336.4

Band gap [eV] = 1.15

$$\varepsilon_\infty = \begin{pmatrix} 11 & & \\ & 11 & \\ & & 7.2 \end{pmatrix}$$

$$\varepsilon(0) = \begin{pmatrix} 40 & & \\ & 40 & \\ & & 7.8 \end{pmatrix}$$

Hamaker Constant [eV] = **1.42**

$f_{Pauling}$ = **0.26**

$E_{total}$ $^{theory\ (RPA)}$ [meV·Å$^{-2}$] = **17.09**
$E_{total}$ $^{theory\ (corrected\ rVV10)}$ [meV·Å$^{-2}$] = **15.77**

$E_{total}$ $^{empirical}$ [meV·Å$^{-2}$] = **18.4**

$E_{dispervise}$ $^{empirical}$ [meV·Å$^{-2}$] = **13.6**

**Notes**:
Density is estimated from XRD data in ref.[293] Band gap is reported in ref.[103] In-plane dielectric constants are reported in ref.[294] Out-of-plane dielectric constants are theoretical prediction in ref.[295]

# Crystal no. 50. $HfTe_2$

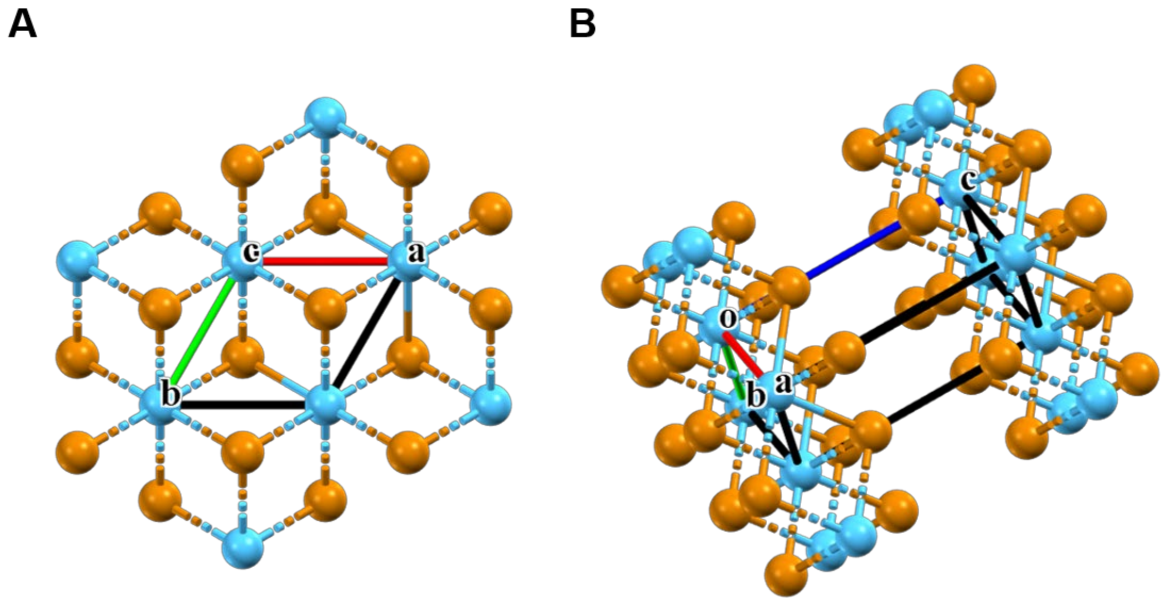


**Fig. S73. Visualization of the packed crystal structure of HfTe2.** A) The packed crystal is visualized along c*. B) To better illustrate the 3D structure, packed cell is visualized employing free-style orientation.

Density [g·cm$^{-3}$] = 8.0

Molecular Mass [g·mol$^{-1}$] = 433.7

Band gap [eV] = N/A

N/A

N/A

Hamaker Constant [eV] = **1.66**

$f_{Pauling}$ = **0.148**

$E_{total}$ $^{theory (RPA)}$ [meV·Å$^{-2}$] = **18.68**
$E_{total}$ $^{theory (corrected rVV10)}$ [meV·Å$^{-2}$] = **17.42**

$E_{total}$ $^{empirical}$ [meV·Å$^{-2}$] = **18.8**

$E_{dispervise}$ $^{empirical}$ [meV·Å$^{-2}$] = **16.0**

**Notes**:
Density is estimated from XRD data in ref.[293] Hamaker constant is calculated assuming the electronic dielectric constant is 20.3.[296] The onset of the interband transitions is estimated to be 0.5 eV from the ab-initio calculation is ref.[297] Using these data, one can estimate the electronic dielectric function required for calculation of the vdW interactions.[16] A Drude band is also included in calculation with a plasma and scattering frequencies of 1.28 and 0.0827 eV.[296] This is equivalent to DC conductivity of about 270 S·cm$^{-1}$ which is consistent with the Hall effect measurements.[298]

# Crystal no. 51. $HgI_2$

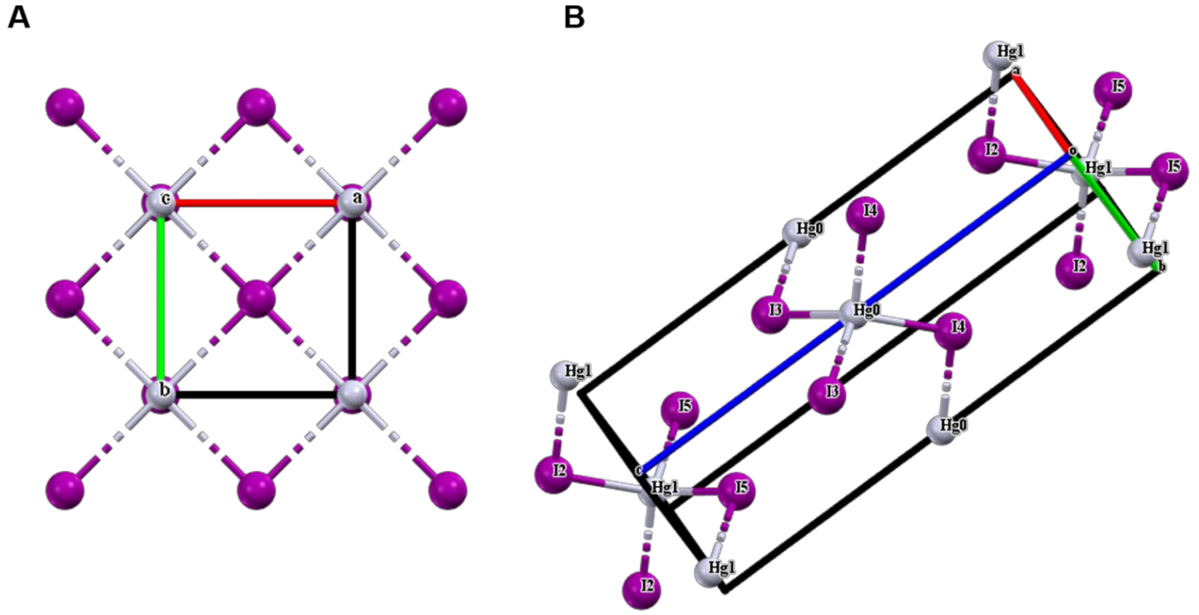


**Fig.S74. Visualization of the packed crystal structure of $HgI_2$. A**) The packed crystal is visualized along c*. B) To better illustrate the 3D structure, packed cell is visualized employing free-style orientation.

Space group: P 42/n m c (137)

Crystal system: Tetragonal

Cell parameters: a=**4.35700 Å**, c= **12.36000 Å**

Density [g·cm$^{-3}$] = 6.43100

Hamaker Constant [eV] = **1.008**

Molecular Mass [g·mol$^{-1}$] = 454.4

$f_{Pauling}$ = **0.103**

Band gap [eV] = 2.3

$$\varepsilon_{\infty} = \begin{pmatrix} 5.03 & & \\ & 5.03 & \\ & & 4.91 \end{pmatrix}$$

$E_{total}$ $^{theory (RPA)}$ [meV·Å$^{-2}$] = **N/A**

$E_{total}$ $^{theory (corrected rVV10)}$ [meV·Å$^{-2}$] = **12.41**

$$\varepsilon(0) = \begin{pmatrix} 8.16 & & \\ & 8.16 & \\ & & 6.17 \end{pmatrix}$$

$E_{total}$ $^{empirical}$ [meV·Å$^{-2}$] = **10.9**

$E_{dispervise}$ $^{empirical}$ [meV·Å$^{-2}$] = **9.7**

**Notes**:

Crystal structure is given in ref.[299] Band gap is reported in ref.[237] Dielectric constants are reported in ref.[300] The infrared bands are located at 12.3 and 15.9 meV.[300]

# Crystal no. 52. $In_2Se_3$

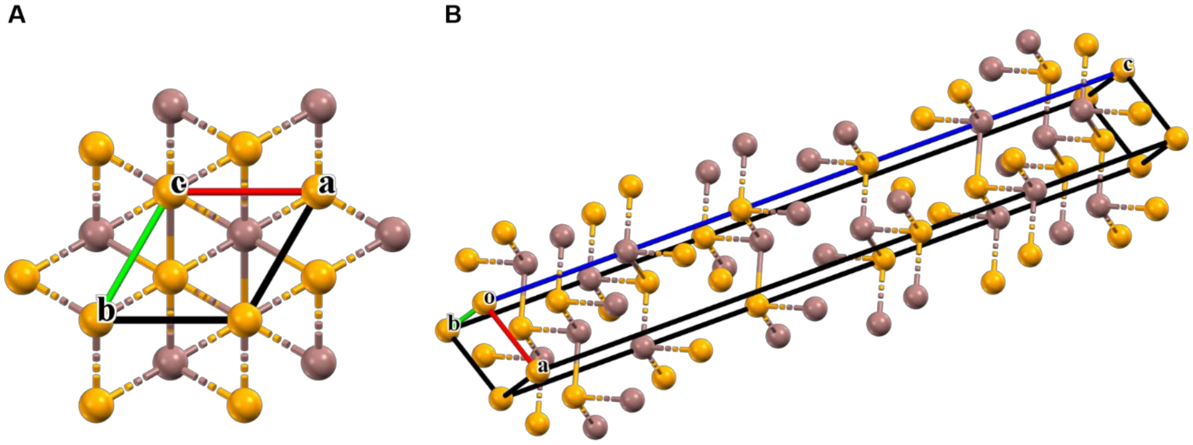


**Fig. S75. Visualization of the packed crystal structure of $In_2Se_3$.** A) The packed crystal is visualized along c*. B) To better illustrate the 3D structure, packed cell is visualized employing free-style orientation.

Space group: R -3 m (166)

Crystal system: Trigonal (Hexagonal axes)

Cell parameters: a=**4.05000 Å**, c= **29.41000 Å**

Density [$g \cdot cm^{-3}$] = 5.563

Hamaker Constant [eV] = **1.49**

Molecular Mass [$g \cdot mol^{-1}$] = 466.5

$f_{Pauling}$ = **0.138**

Band gap [eV] = 1.45

$$\varepsilon_\infty = \begin{pmatrix} 9.51 & & \\ & 9.51 & \\ & & 6.37 \end{pmatrix}$$

$E_{total}^{theory\ (RPA)}$ [$meV \cdot Å^{-2}$] = **N/A**

$E_{total}^{theory\ (corrected\ rVV10)}$ [$meV \cdot Å^{-2}$] = **15.51**

$$\varepsilon(0) = \begin{pmatrix} 23.28 & & \\ & 23.28 & \\ & & 7.05 \end{pmatrix}$$

$E_{total}^{empirical}$ [$meV \cdot Å^{-2}$] = **16.2**

$E_{dispervise}^{empirical}$ [$meV \cdot Å^{-2}$] = **13.9**

**Notes**:

Crystal structure is given ref.[301] Band gap is reported in ref.[302] Dielectric constants and position of infrared bands are reported in ref.[303]

# Crystal no. 53. InS

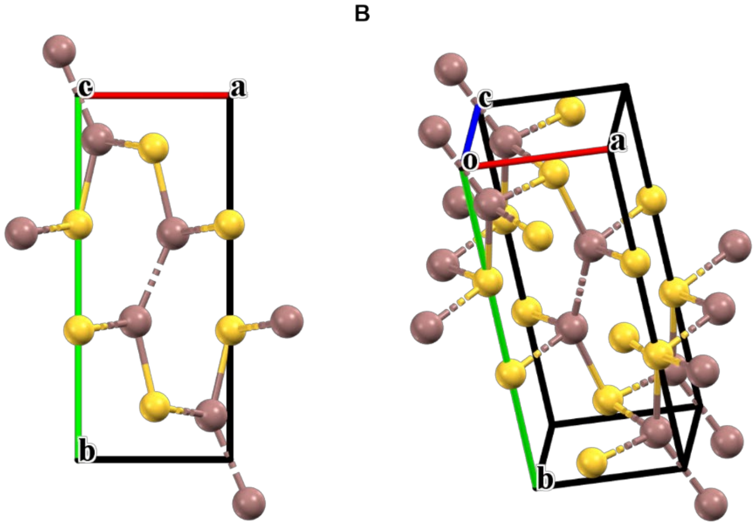


**Fig. S76. Visualization of the packed crystal structure of InS.** A) The packed crystal is visualized along c*. B) To better illustrate the 3D structure, packed cell is visualized employing free-style orientation.

Space group: P n n m (58)

Crystal system: Orthorhombic

Cell parameters: a=**4.44300 Å**, b= **10.64200 Å** c= **3.94000 Å**

Density [g·cm$^{-3}$] = 5.23700

Hamaker Constant [eV] = **1.59**

Molecular Mass [g·mol$^{-1}$] = 146.9

$f_{Pauling}$ = **0.148**

Band gap [eV] = 1.9

$$\varepsilon_\infty = \begin{pmatrix} 8.39 & & \\ & 8.39 & \\ & & 6.64 \end{pmatrix}$$

$E_{total}^{\text{theory (RPA)}}$ [meV·Å$^{-2}$] = **N/A**
$E_{total}^{\text{theory (corrected rVV10)}}$ [meV·Å$^{-2}$] = **N/A**

N/A

$E_{total}^{\text{empirical}}$ [meV·Å$^{-2}$] = **17.9**

$E_{dispervise}^{\text{empirical}}$ [meV·Å$^{-2}$] = **15.3**

**Notes**:
Crystal structure is given in ref.[304] Band gap and dielectric constants are given in ref.[305]

# Crystal no. 54. InSe

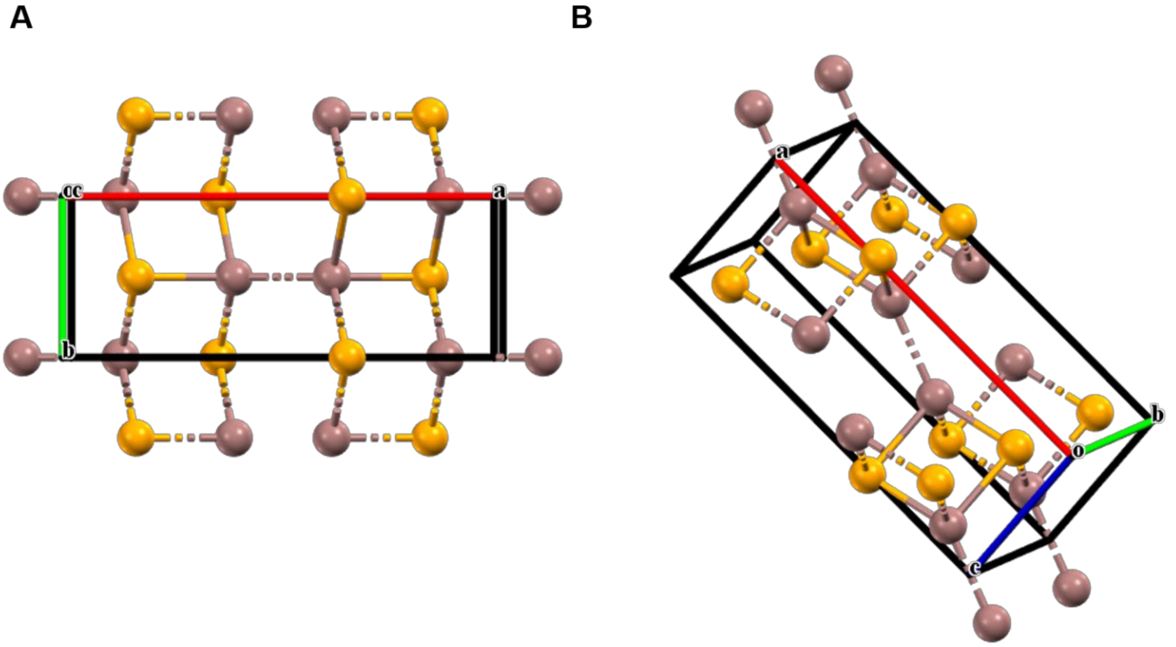


**Fig. S77. Visualization of the packed crystal structure of InSe.** A) The packed crystal is visualized along c*. B) To better illustrate the 3D structure, packed cell is visualized employing free-style orientation.

Space group: R 3 m (160)

Crystal system: Trigonal (Hexagonal axes)

Cell parameters: a=**4.00000 Å**, c= **24.95000 Å**

Density [g·cm$^{-3}$] = 5.58400

Hamaker Constant [eV] = **1.262**

Molecular Mass [g·mol$^{-1}$] = 193.8

$f_{Pauling}$ = **0.138**

Band gap [eV] = 1.27

$$\varepsilon_{\infty} = \begin{pmatrix} 8.8 & & \\ & 8.8 & \\ & & 7.8 \end{pmatrix}$$

$E_{total}$ $^{theory\ (RPA)}$ [meV·Å$^{-2}$] = **N/A**
$E_{total}$ $^{theory\ (corrected\ rVV10)}$ [meV·Å$^{-2}$] = **13.73**

$$\varepsilon(0) = \begin{pmatrix} 12 & & \\ & 12 & \\ & & 8.4 \end{pmatrix}$$

$E_{total}$ $^{empirical}$ [meV·Å$^{-2}$] = **14.1**

$E_{dispervise}$ $^{empirical}$ [meV·Å$^{-2}$] = **12.1**

**Notes**:
Crystal structure is given in ref.[306] Band gap is reported in ref.[307] Dielectric constants are reported in ref.[308] The theoretical binding energy cited here is for the hexagonal polymorph.[30]

# Crystal no. 55. InTeBr

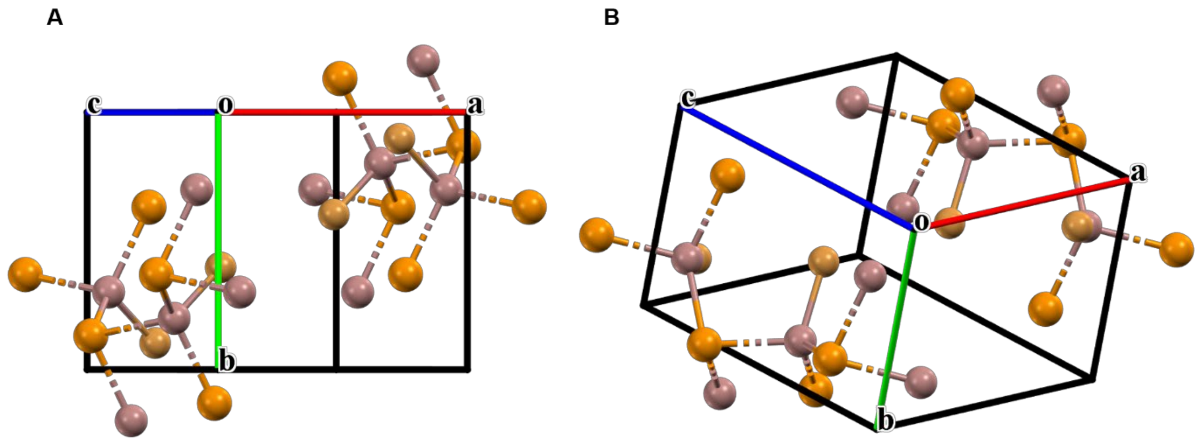


**Fig. S78. Visualization of the packed crystal structure of InTeBr.** A) The packed crystal is visualized along c*. B) To better illustrate the 3D structure, packed cell is visualized employing free-style orientation.

Space group: P 1 21/c 1 (14)

Crystal system: Monoclinic

Cell parameters: a=**7.35000 Å**, b= **7.57700 Å** c= **8.34300 Å**, β= **117.610 º**

Density [g·cm$^{-3}$] = 5.20000

Hamaker Constant [eV] = **1.34**

Molecular Mass [g·mol$^{-1}$] = 322.3

$f_{Pauling}$ = **0.025**

Band gap [eV] = 2.45

$$\varepsilon_\infty = \begin{pmatrix} 6.0 & & \\ & 6.0 & \\ & & N/A \end{pmatrix}$$

$E_{total}$ $^{theory\ (RPA)}$ [meV·Å$^{-2}$] = **N/A**

$E_{total}$ $^{theory\ (corrected\ rVV10)}$ [meV·Å$^{-2}$] = **13.00**

N/A

$E_{total}$ $^{empirical}$ [meV·Å$^{-2}$] = **13.2**

$E_{dispervise}$ $^{empirical}$ [meV·Å$^{-2}$] = **12.9**

**Notes**:

Crystal structure is given in ref.[309] Band gap is reported in ref.[310] Electronic dielectric constant is approximated using the Eqs. (7) and (S18). Anisotropy is ignored. Ionicity is the ionicity of In-Te bond.

# Crystal no. 56. InTeI

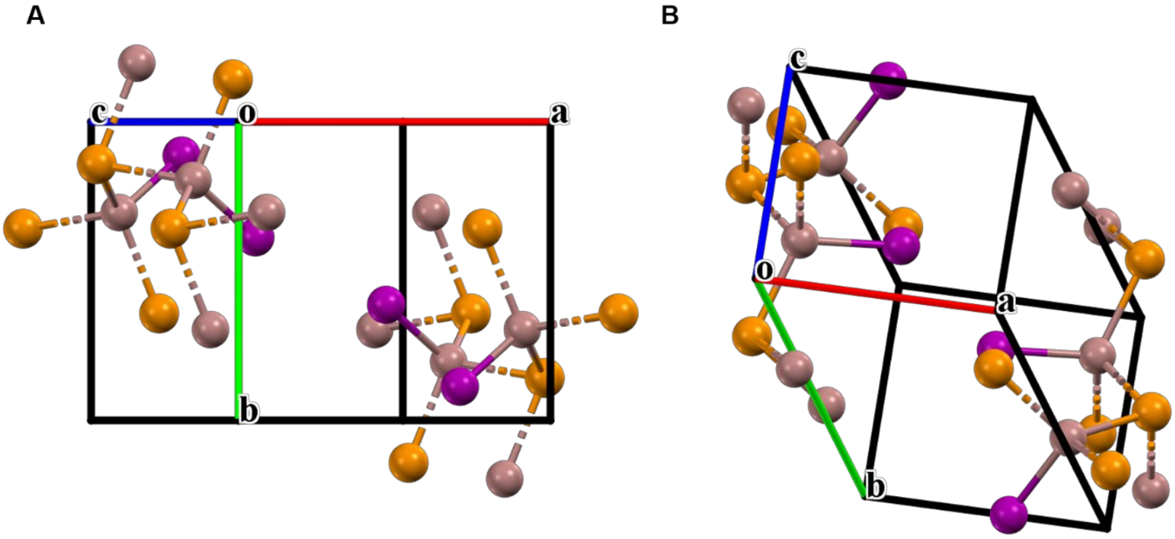


**Fig. S79. Visualization of the packed crystal structure of InTeI.** A) The packed crystal is visualized along c*. B) To better illustrate the 3D structure, packed cell is visualized employing free-style orientation.

Space group: P 1 21/c 1 (14)

Crystal system: Monoclinic

Cell parameters: a=**8.07600 Å**, b= **7.73100 Å** c= **8.40700 Å**, β= **117.030 º**

Density [g·cm$^{-3}$] = 5.24600

Hamaker Constant [eV] = **1.32**

Molecular Mass [g·mol$^{-1}$] = 369.3

$f_{Pauling}$ = **0.025**

Band gap [eV] = 2.3

$$\varepsilon_{\infty} = \begin{pmatrix} 6.1 & & \\ & 6.1 & \\ & & N/A \end{pmatrix}$$

E$_{total}$ $^{theory\ (RPA)}$ [meV·Å$^{-2}$] = **N/A**

E$_{total}$ $^{theory\ (corrected\ rVV10)}$ [meV·Å$^{-2}$] = **12.54**

N/A

E$_{total}$ $^{empirical}$ [meV·Å$^{-2}$] = **12.9**

E$_{dispervise}$ $^{empirical}$ [meV·Å$^{-2}$] = **12.6**

**Notes**:
Crystal structure is given in ref.[311] Band gap is reported in ref.[310] Electronic dielectric constant is approximated using the Eqs. (7) and (S18). Anisotropy is ignored. Ionicity is the ionicity of In-Te bond.

# Crystal no. 57. $KAl_3Si_3O_{10}(OH)_{1.8}F_{0.2}$ , Mica

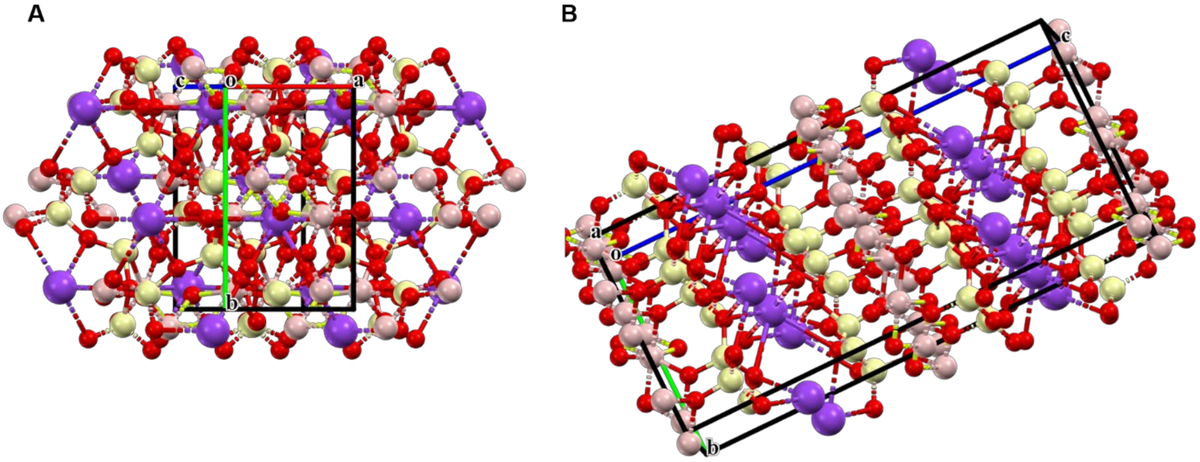


**Fig. S80. Visualization of the packed crystal structure of Mica.** A) The packed crystal is visualized along c*. B) To better illustrate the 3D structure, packed cell is visualized employing free-style orientation.

Space group: C 1 2/c 1 (15)

Crystal system: Monoclinic

Cell parameters: a= **5.18200 Å** b= **8.99300 Å** c= **20.23200 Å** β= **95.750 º**

Density [g·cm$^{-3}$] = 2.82300

Hamaker Constant [eV] = **680.3245**

Molecular Mass [g·mol$^{-1}$] = 398.7

$f_{Pauling}$ = **0.567**

Band gap [eV] = 7

$$\varepsilon_\infty = \begin{pmatrix} 2.485 & & \\ & 2.485 & \\ & & 2.485 \end{pmatrix}$$

$E_{total}$ $^{theory (RPA)}$ [meV·Å$^{-2}$] = **N/A**
$E_{total}$ $^{theory (corrected rVV10)}$ [meV·Å$^{-2}$] = **N/A**

$$\varepsilon(0) = \begin{pmatrix} 7.5 & & \\ & 7.5 & \\ & & 7.5 \end{pmatrix}$$

$E_{total}$ $^{experimental}$ [meV·Å$^{-2}$] = **15.8±4.4 (N=6)**
$E_{total}$ $^{empirical}$ [meV·Å$^{-2}$] = **15.1**

$E_{dispervise}$ $^{empirical}$ [meV·Å$^{-2}$] = **6.55**

**Notes**:
Crystal structure is given in ref.[312] Dielectric function of mica is given in our previous work.[16] Anisotropy is minor in mica and is ignored here. The experimental binding energy is the average value of the reported values in literature which are listed in Table S2. Due to the complex structure of mica, it is not trivial to define the Pauling ionicity. Here we assign the ionicity of Al-O bond to the ionicity of mica.

# Crystal no. 58. $Mg_3Si_4O_{10}(OH)_2$ , Talc

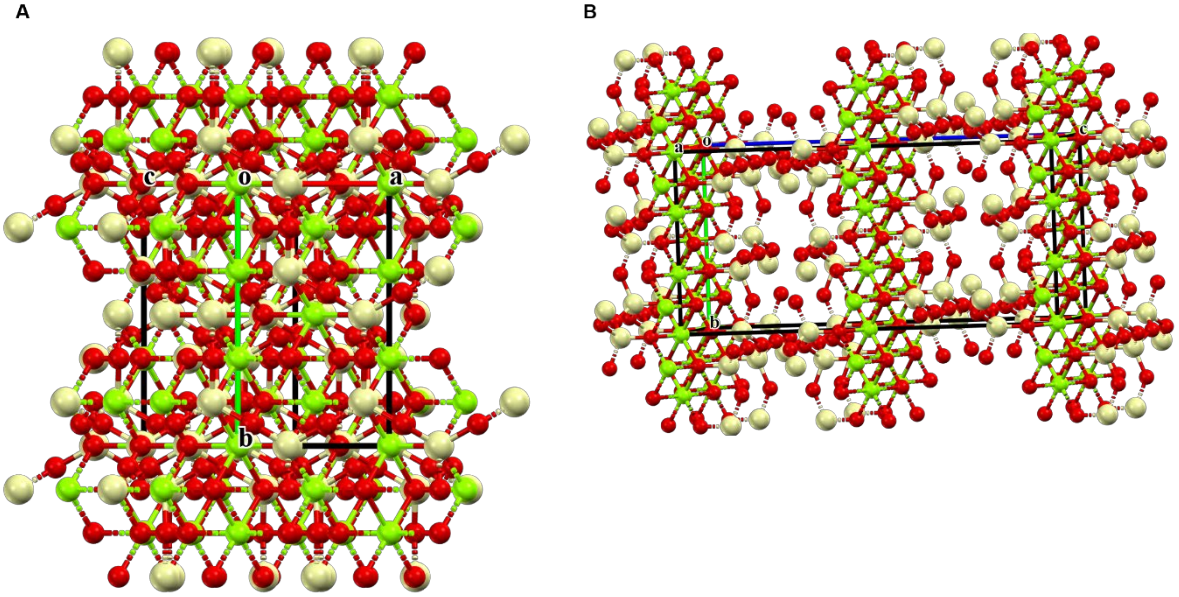


**Fig. S81. Visualization of the packed crystal structure of Talc.** A) The packed crystal is visualized along c*. B) To better illustrate the 3D structure, packed cell is visualized employing free-style orientation.

Space group: C 1 2/c 1 (15)

Crystal system: Monoclinic

Cell parameters: a=**5.26000 Å**, b= **9.10000 Å** c= **18.81000 Å**, β= **100.000 °**

Density [g·cm$^{-3}$] = 2.84100

Hamaker Constant [eV] = **0.40**

Molecular Mass [g·mol$^{-1}$] = 379.3

$f_{Pauling}$ = **0.678**

Band gap [eV] = 5.2

$$\varepsilon_\infty = \begin{pmatrix} 2.4 & & \\ & 2.4 & \\ & & 2.4 \end{pmatrix}$$

N/A

$E_{total}$ $^{theory\ (RPA)}$ [meV·Å$^{-2}$] = **N/A**

$E_{total}$ $^{theory\ (corrected\ rVV10)}$ [meV·Å$^{-2}$] = **N/A**

$E_{total}$ $^{empirical}$ [meV·Å$^{-2}$] = **14.75**

$E_{dispervise}$ $^{empirical}$ [meV·Å$^{-2}$] = **4.75**

**Notes**:

Crystal structure is given in ref.[313] Band gap is reported in ref.[314] Dielectric constant is approximated from the refractive indices.[315] Anisotropy is minor and is ignored. Due to the complex structure of talc, it is not trivial to define the Pauling ionicity. Here we assign the ionicity of Mg-O bond to the ionicity of talc.

# Crystal no. 59. $Mg(OH)_2$

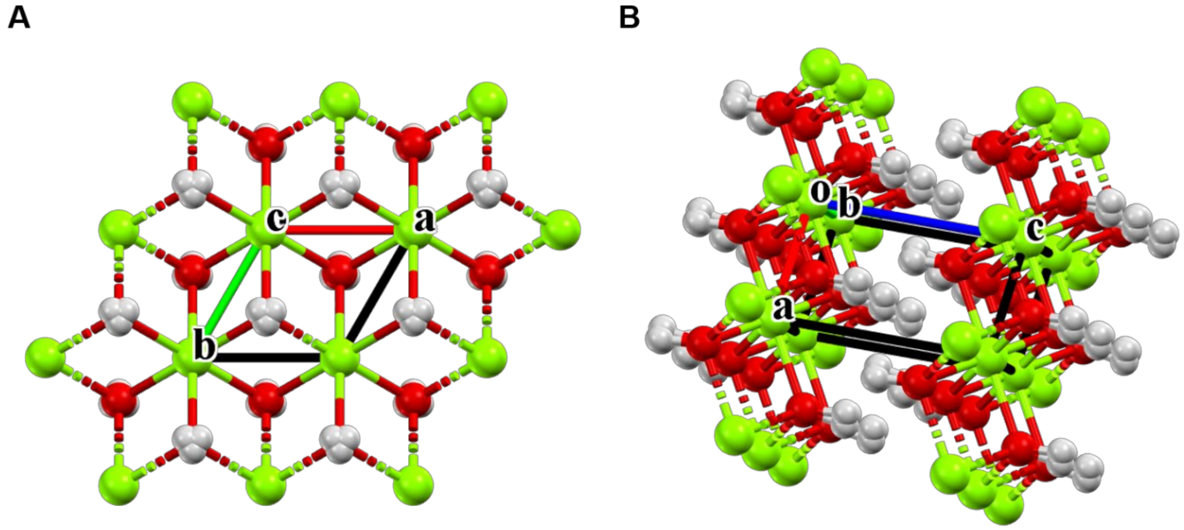


**Fig. S82. Visualization of the packed crystal structure of $Mg(OH)_2$.** A) The packed crystal is visualized along c*. B) To better illustrate the 3D structure, packed cell is visualized employing free-style orientation.

Space group: P -3 m 1 (164)

Crystal system: Trigonal (Hexagonal axes)

Cell parameters: a=**3.14800 Å**, c= **4.77900 Å**

Density [g·cm$^{-3}$] = 2.36000

Hamaker Constant [eV] = **0.59**

Molecular Mass [g·mol$^{-1}$] = 58.32

$f_{Pauling}$ = **0.678**

Band gap [eV] = 6

$$\varepsilon_\infty = \begin{pmatrix} 2.5 & & \\ & 2.5 & \\ & & N/A \end{pmatrix}$$

$E_{total}$ $^{theory\ (RPA)}$ [meV·Å$^{-2}$] = **N/A**

$E_{total}$ $^{theory\ (corrected\ rVV10)}$ [meV·Å$^{-2}$] = **24.16**

N/A

$E_{total}$ $^{empirical}$ [meV·Å$^{-2}$] = **17.5**

$E_{dispervise}$ $^{empirical}$ [meV·Å$^{-2}$] = **5.65**

**Notes**:

Crystal structure is given in ref.[316] Band gap is reported in ref.[317] Electronic dielectric constant is estimated from the refractive indices reported for brucite mineral.[318] Anisotropy is minor and is ignored.

# Crystal no. 60. $MgCl_2$

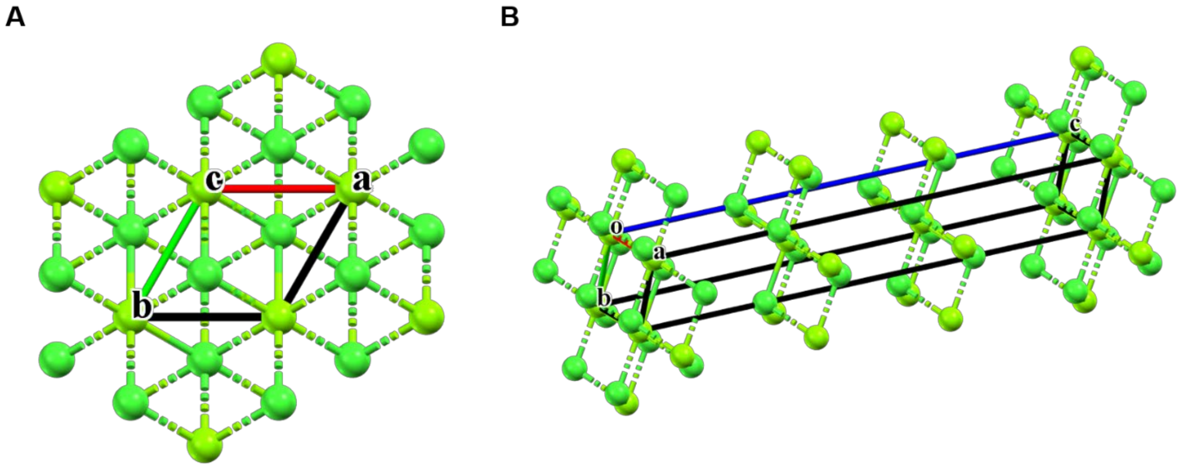


**Fig. S83. Visualization of the packed crystal structure of $MgCl_2$.** A) The packed crystal is visualized along c*. B) To better illustrate the 3D structure, packed cell is visualized employing free-style orientation.

Space group: R -3 m (166)

Crystal system: Trigonal (Hexagonal axes)

Cell parameters: a=**3.59500 Å**, c= **17.59000 Å**

Density [g·cm$^{-3}$] = 2.36000

Hamaker Constant [eV] = **0.78**

Molecular Mass [g·mol$^{-1}$] = 95.21

$f_{Pauling}$ = **0.575**

Band gap [eV] = 7.5

$$\varepsilon_\infty = \begin{pmatrix} 2.8 & & \\ & 2.8 & \\ & & 2.4 \end{pmatrix}$$

N/A

$E_{total}$ $^{theory\ (RPA)}$ [meV·Å$^{-2}$] = **N/A**

$E_{total}$ $^{theory\ (corrected\ rVV10)}$ [meV·Å$^{-2}$] = **11.68**

$E_{total}$ $^{empirical}$ [meV·Å$^{-2}$] = **17.7**

$E_{dispervise}$ $^{empirical}$ [meV·Å$^{-2}$] = **7.5**

**Notes**:
Crystal structure is given in ref.[319] Band gap is reported in ref.[320] Electronic dielectric constant (in-plane) is reported in ref.[321] Electronic dielectric constant in out-of-plane polarization is the theoretical prediction.[54]

# Crystal no. 61. $MnPS_3$

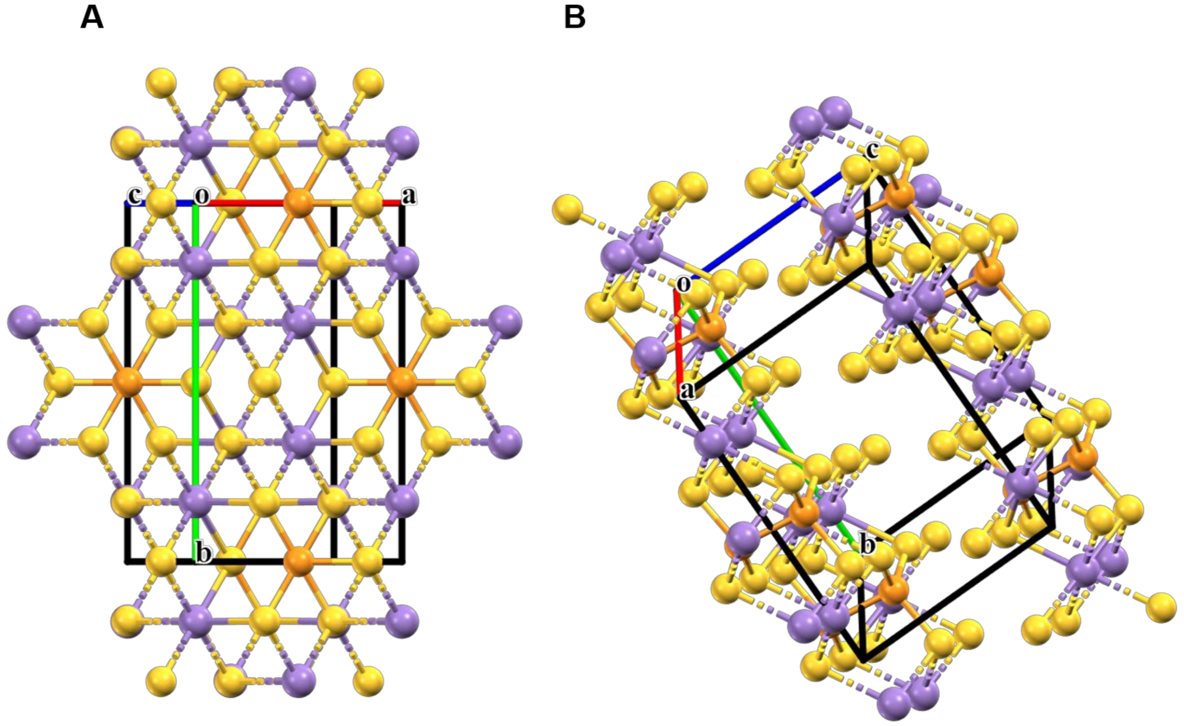


**Fig. S84. Visualization of the packed crystal structure of MnPS3.** A) The packed crystal is visualized along c*. B) To better illustrate the 3D structure, packed cell is visualized employing free-style orientation.

Density [g·cm$^{-3}$] = 2.91

Molecular Mass [g·mol$^{-1}$] = 182.1

Band gap [eV] = 2.85

$$\varepsilon_\infty = \begin{pmatrix} 5.5 & & \\ & 5.5 & \\ & & N/A \end{pmatrix}$$

N/A

Hamaker Constant [eV] = **1.29**

$f_{Pauling}$ = **0.233**

$E_{total}$ $^{theory\ (RPA)}$ [meV·Å$^{-2}$] = **N/A**

$E_{total}$ $^{theory\ (corrected\ rVV10)}$ [meV·Å$^{-2}$] = **18.22**

$E_{total}$ $^{empirical}$ [meV·Å$^{-2}$] = **16.2**

$E_{dispervise}$ $^{empirical}$ [meV·Å$^{-2}$] = **12.4**

**Notes**:
Density is calculated from XRD data in ref.[322] Band gap and electronic dielectric constant is reported in ref.[323] Anisotropy is ignored. Ionicity is for the Mn-S bond.

# Crystal no. 62. $MnPSe_3$

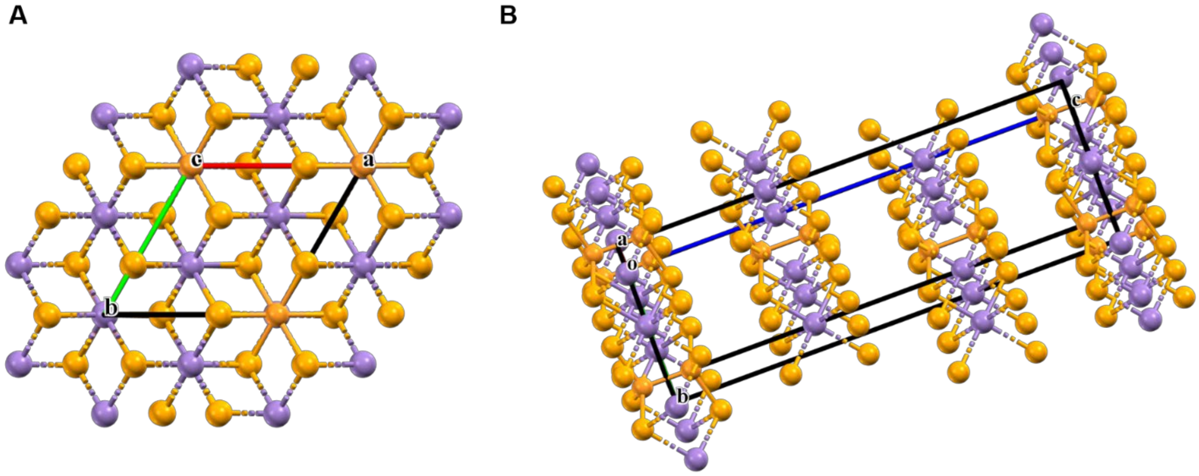


**Fig. S85. Visualization of the packed crystal structure of $MnPSe_3$.** A) The packed crystal is visualized along c*. B) To better illustrate the 3D structure, packed cell is visualized employing free-style orientation.

Density [g·cm$^{-3}$] = 4.55

Molecular Mass [g·mol$^{-1}$] = 322.8

Band gap [eV] = 2.3

$$\varepsilon_\infty = \begin{pmatrix} 7.1 & & \\ & 7.1 & \\ & & N/A \end{pmatrix}$$

N/A

Hamaker Constant [eV] = **1.52**

$f_{Pauling}$ = **0.221**

$E_{total}$ $^{theory\ (RPA)}$ [meV·Å$^{-2}$] = **N/A**

$E_{total}$ $^{theory\ (corrected\ rVV10)}$ [meV·Å$^{-2}$] = **20.13**

$E_{total}$ $^{empirical}$ [meV·Å$^{-2}$] = **18.7**

$E_{dispervise}$ $^{empirical}$ [meV·Å$^{-2}$] = **14.6**

**Notes**:
Density is calculated from neutron diffraction data in ref.[324] Band gap is reported in ref.[325] It is slightly lower compared to the one reported in ref.[268] (2.5 eV). Electronic dielectric constant is estimated from the reflectance spectrum in ref.[325] Anisotropy is ignored. Ionicity is for the Mn-Se bond.

# Crystal no. 63. $MoO_3$

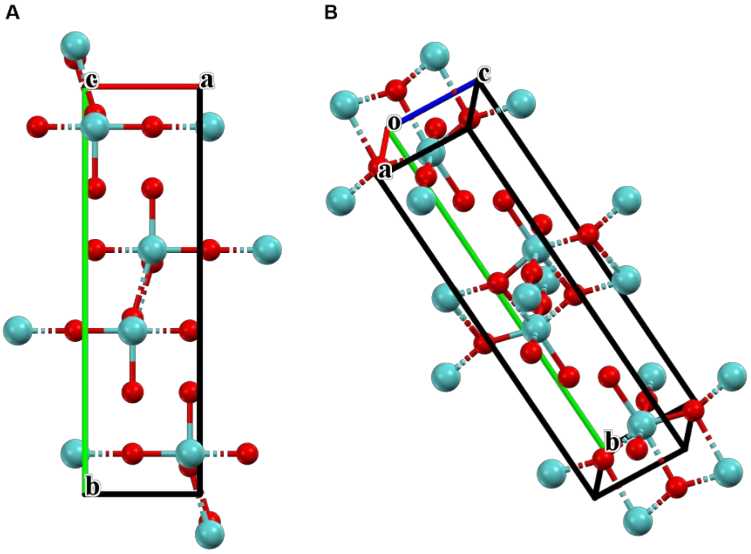


**Fig. S86. Visualization of the packed crystal structure of $MoO_3$.** A) The packed crystal is visualized along c*. B) To better illustrate the 3D structure, packed cell is visualized employing free-style orientation.

Space group: P b n m (62)

Crystal system: Orthorhombic

Cell parameters: a=**3.92000 Å**, b= **13.94000 Å** c= **3.66000 Å**

Density [g·cm$^{-3}$] = 4.78000

Hamaker Constant [eV] = **1.41**

Molecular Mass [g·mol$^{-1}$] = 143.94

$f_{Pauling}$ = **0.336**

Band gap [eV] = 2.6

$$\varepsilon_\infty = \begin{pmatrix} 6.30 & & \\ & 5.52 & \\ & & 4.25 \end{pmatrix}$$

$E_{total}$ $^{theory\ (RPA)}$ [meV·Å$^{-2}$] = **N/A**
$E_{total}$ $^{theory\ (corrected\ rVV10)}$ [meV·Å$^{-2}$] = **17.56**

$$\varepsilon(0) = \begin{pmatrix} 15 & & \\ & 6.89 & \\ & & 4.49 \end{pmatrix}$$

$E_{total}$ $^{empirical}$ [meV·Å$^{-2}$] = **20.45**

$E_{dispervise}$ $^{empirical}$ [meV·Å$^{-2}$] = **13.6**

**Notes**:
Crystal structure is given in ref.[326] Dielectric function of $MoO_3$ in all three axes are given in section 12.

# Crystal no. 64. $MoS_2$

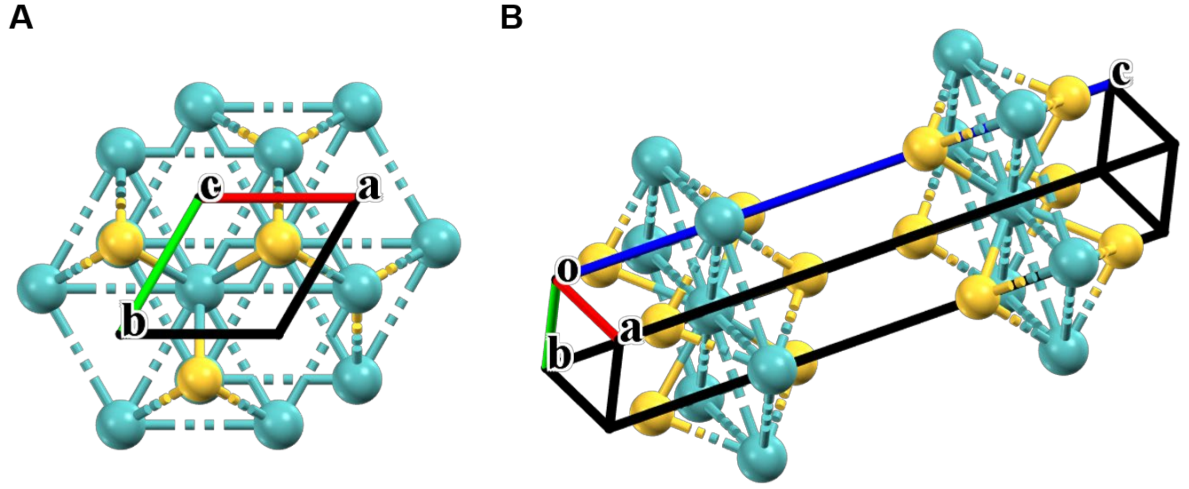


**Fig. S87. Visualization of the packed crystal structure of $MoS_2$.** A) The packed crystal is visualized along c*. B) To better illustrate the 3D structure, packed cell is visualized employing free-style orientation.

Space group: P 63/m m c (194)

Crystal system: Hexagonal

Cell parameters: a=**3.15000 Å**, c= **12.30000 Å**

Density [g·cm$^{-3}$] = 5.02900

Hamaker Constant [eV] = **1.95**

Molecular Mass [g·mol$^{-1}$] = 160.07

$f_{Pauling}$ = **0.043**

Band gap [eV] = 1.3

$$\varepsilon_{\infty} = \begin{pmatrix} 15.8 & & \\ & 15.8 & \\ & & 6.36 \end{pmatrix}$$

$E_{total}^{\text{theory (RPA)}}$ [meV·Å$^{-2}$] = **20.53**

$E_{total}^{\text{theory (corrected rVV10)}}$ [meV·Å$^{-2}$] = **22.11**

$$\varepsilon(0) = \begin{pmatrix} 15.95 & & \\ & 15.95 & \\ & & 6.39 \end{pmatrix}$$

$E_{total}^{\text{empirical}}$ [meV·Å$^{-2}$] = **19.7**

$E_{dispervise}^{\text{empirical}}$ [meV·Å$^{-2}$] = **18.8**

**Notes**:

Crystal structure is given in ref.[327] Dielectric function of $MoS_2$ is given in our previous work.[16]

# Crystal no. 65. $MoSe_2$

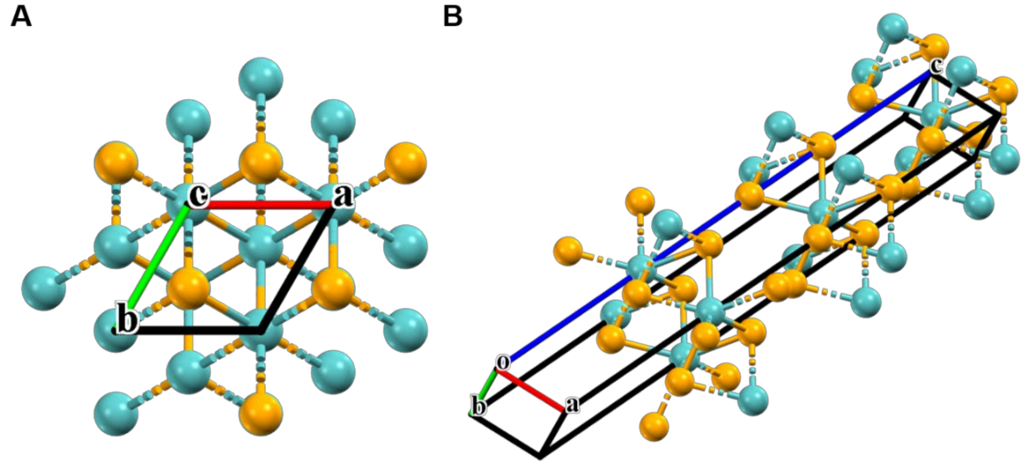


**Fig. S88. Visualization of the packed crystal structure of $MoSe_2$.** A) The packed crystal is visualized along c*. B) To better illustrate the 3D structure, packed cell is visualized employing free-style orientation.

Space group: R 3 m (160)

Crystal system: Trigonal (Hexagonal axes)

Cell parameters: a=**3.29200 Å**, = **19.39200 Å**

Density [$g \cdot cm^{-3}$] = 6.948000

Hamaker Constant [eV] = **2.12**

Molecular Mass [$g \cdot mol^{-1}$] = 253.9

$f_{Pauling}$ = **0.037**

Band gap [eV] = 1.1

$$\varepsilon_{\infty} = \begin{pmatrix} 17.52 & & \\ & 17.52 & \\ & & 8.3 \end{pmatrix}$$

$E_{total}^{theory\ (RPA)}$ [$meV \cdot Å^{-2}$] = **19.63**

$E_{total}^{theory\ (corrected\ rVV10)}$ [$meV \cdot Å^{-2}$] = **18.744**

$$\varepsilon(0) = \begin{pmatrix} 18.12 & & \\ & 18.12 & \\ & & N/A \end{pmatrix}$$

$E_{total}^{empirical}$ [$meV \cdot Å^{-2}$] = **21.2**

$E_{dispervise}^{empirical}$ [$meV \cdot Å^{-2}$] = **20.4**

**Notes**:
Crystal structure is given in ref.[328] Dielectric function of $MoSe_2$ is given in our previous work.[16]

# Crystal no. 66. $MoTe_2$

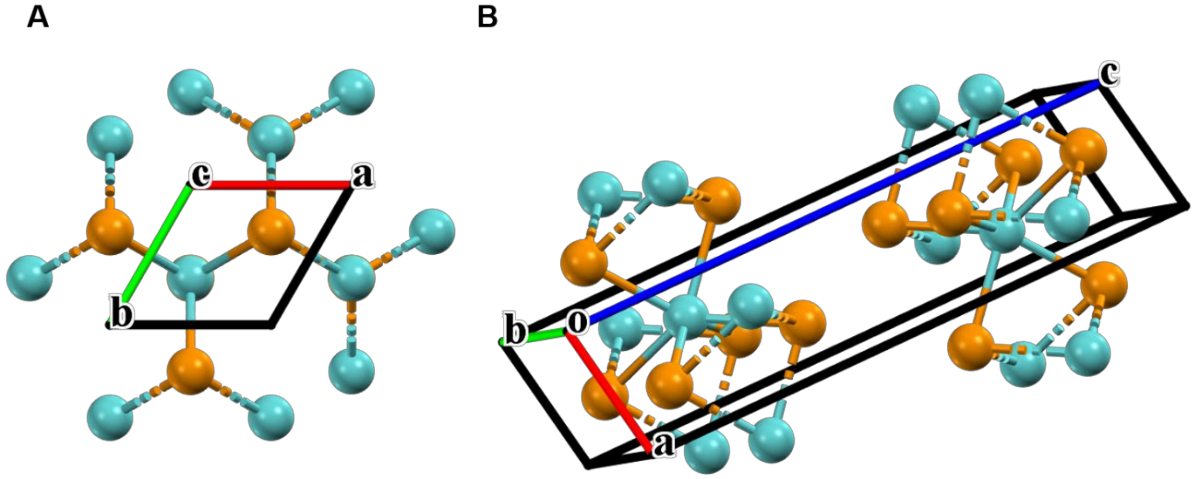


**Fig. S89. Visualization of the packed crystal structure of $MoTe_2$.** A) The packed crystal is visualized along c*. B) To better illustrate the 3D structure, packed cell is visualized employing free-style orientation.

Space group: P 63/m m c (194)

Crystal system: Hexagonal

Cell parameters: a=**3.51900 Å**, c= **13.96400 Å**

Density [g·cm$^{-3}$] = 7.78700

Hamaker Constant [eV] = **2.217**

Molecular Mass [g·mol$^{-1}$] = 351.1

$f_{Pauling}$ = **0.0009**

Band gap [eV] = 1

$$\varepsilon_\infty = \begin{pmatrix} 20.25 & & \\ & 20.25 & \\ & & 10 \end{pmatrix}$$

$E_{total}$ $^{theory\ (RPA)}$ [meV·Å$^{-2}$] = **20.80**
$E_{total}$ $^{theory\ (corrected\ rVV10)}$ [meV·Å$^{-2}$] = **19.87**

$$\varepsilon(0) = \begin{pmatrix} 22.25 & & \\ & 22.25 & \\ & & N/A \end{pmatrix}$$

$E_{total}$ $^{empirical}$ [meV·Å$^{-2}$] = **21.4**

$E_{dispervise}$ $^{empirical}$ [meV·Å$^{-2}$] = **21.3**

**Notes**:
Crystal structure is given in ref.[329] Dielectric function of $MoTe_2$ is given in our previous work.[16]

# Crystal no. 67. $NbS_2$

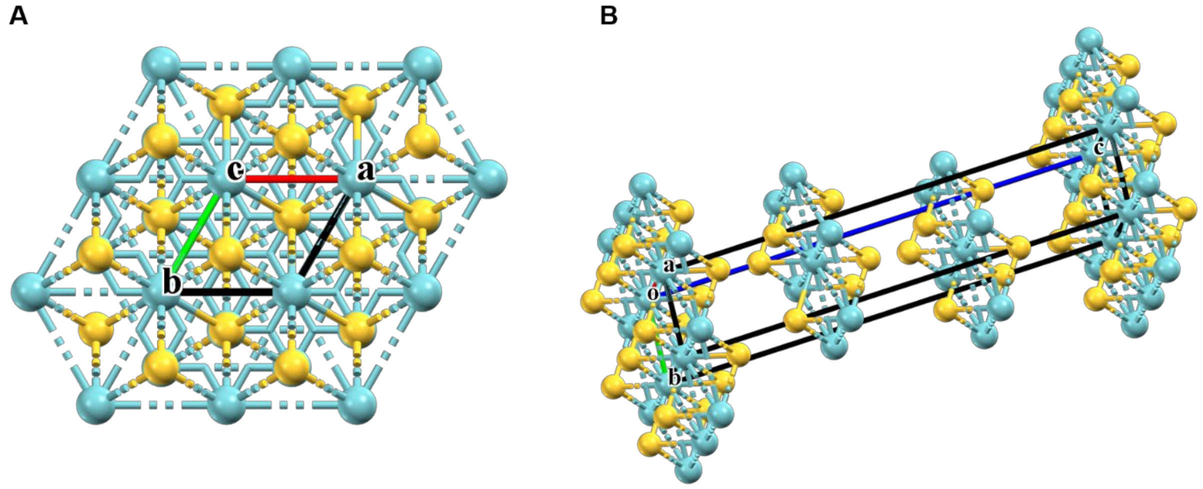


**Fig. S90. Visualization of the packed crystal structure of $NbS_2$.** A) The packed crystal is visualized along c*. B) To better illustrate the 3D structure, packed cell is visualized employing free-style orientation.

Space group: R -3 m (166)

Crystal system: Trigonal (Hexagonal axes)

Cell parameters: a=**3.33000 Å**, c= **17.80000 Å**

Density [g·cm$^{-3}$] = 4.57600

Hamaker Constant [eV] = **1.82**

Molecular Mass [g·mol$^{-1}$] = 157.04

$f_{Pauling}$ = **0.213**

Band gap [eV] = N/A

N/A

$E_{total}^{theory\ (RPA)}$ [meV·Å$^{-2}$] = **17.58**
$E_{total}^{theory\ (corrected\ rVV10)}$ [meV·Å$^{-2}$] = **18.48**

N/A

$E_{total}^{empirical}$ [meV·Å$^{-2}$] = **22.2**

$E_{dispervise}^{empirical}$ [meV·Å$^{-2}$] = **17.5**

**Notes**:
Crystal structure is given in ref.[330] Dielectric function of $NbS_2$ is given in section 12.

# Crystal no. 68. $NbSe_2$

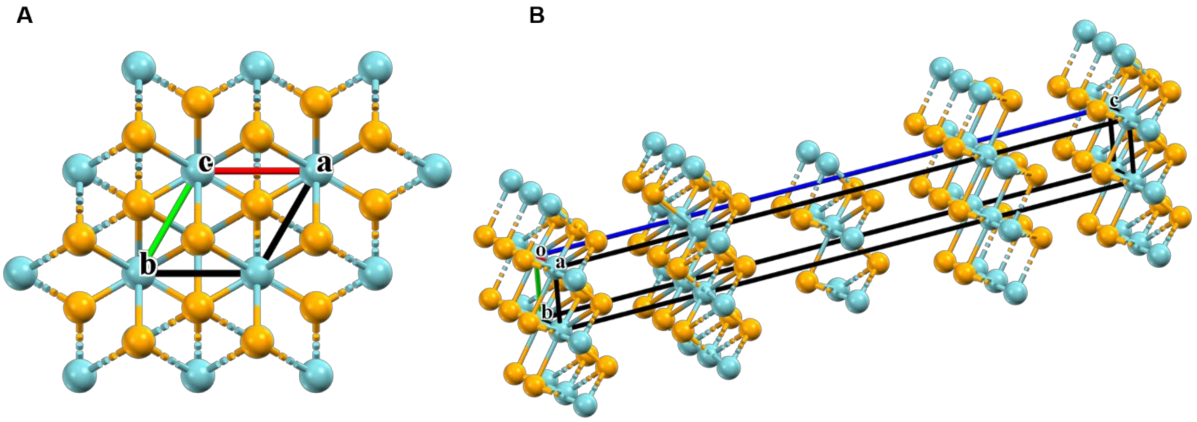


**Fig. S91. Visualization of the packed crystal structure of $NbSe_2$.** A) The packed crystal is visualized along c*. B) To better illustrate the 3D structure, packed cell is visualized employing free-style orientation.

Space group: P -6 m 2 (187)

Crystal system: Hexagonal

Cell parameters: a=**3.48000 Å**, c= **25.45000 Å**

Density [g·cm$^{-3}$] = 6.24100

Hamaker Constant [eV] = **1.826**

Molecular Mass [g·mol$^{-1}$] = 250.8

$f_{Pauling}$ = **0.202**

Band gap [eV] = N/A

N/A

$E_{total}^{theory (RPA)}$ [meV·Å$^{-2}$] = **19.57**
$E_{total}^{theory (corrected rVV10)}$ [meV·Å$^{-2}$] = **19.01**

N/A

$E_{total}^{empirical}$ [meV·Å$^{-2}$] = **22.0**

$E_{dispervise}^{empirical}$ [meV·Å$^{-2}$] = **17.6**

**Notes**:
Crystal structure is given in ref.[331] Dielectric function of $NbSe_2$ is given in our previous work.[16]

# Crystal no. 69. $Ni(OH)_2$

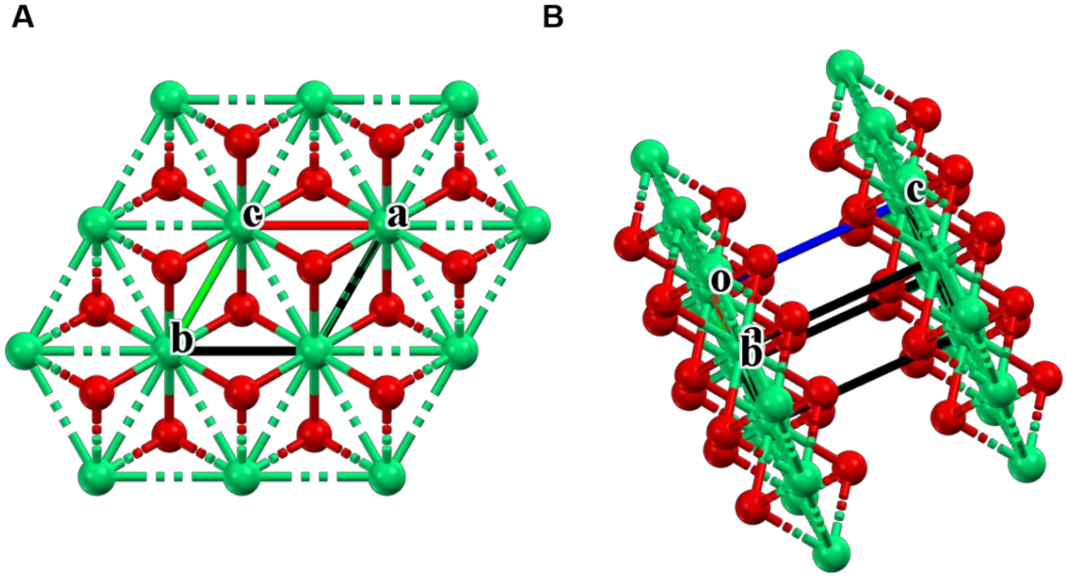


**Fig S92. Visualization of the packed crystal structure of $Ni(OH)_2$.** A) The packed crystal is visualized along c*. B) To better illustrate the 3D structure, packed cell is visualized employing free-style orientation.

Space group: P -3 m 1 (164)

Crystal system: Trigonal (Hexagonal axes)

Cell parameters: a=**3.11400 Å**, c= **4.61700 Å**

Density [g·cm$^{-3}$] = 3.92700

Hamaker Constant [eV] = **0.77**

Molecular Mass [g·mol$^{-1}$] = 92.71

$f_{Pauling}$ = **0.443**

Band gap [eV] = 5

$$\varepsilon_\infty = \begin{pmatrix} 3 & & \\ & 3 & \\ & & N/A \end{pmatrix}$$

$E_{total}$ $^{theory\ (RPA)}$ [meV·Å$^{-2}$] = **N/A**

$E_{total}$ $^{theory\ (corrected\ rVV10)}$ [meV·Å$^{-2}$] = **18.2**

N/A

$E_{total}$ $^{empirical}$ [meV·Å$^{-2}$] = **13.3**

$E_{dispervise}$ $^{empirical}$ [meV·Å$^{-2}$] = **7.4**

**Notes**:
Crystal structure is given in ref.[332] Band gap is calculated from the absorption spectrum given in ref.[333] Electronic dielectric constant is approximated from the refractive indices reported in ref.[334] Theoretical binding energy refers to the one for $NiO_2$.

# Crystal no. 70. $NiI_2$

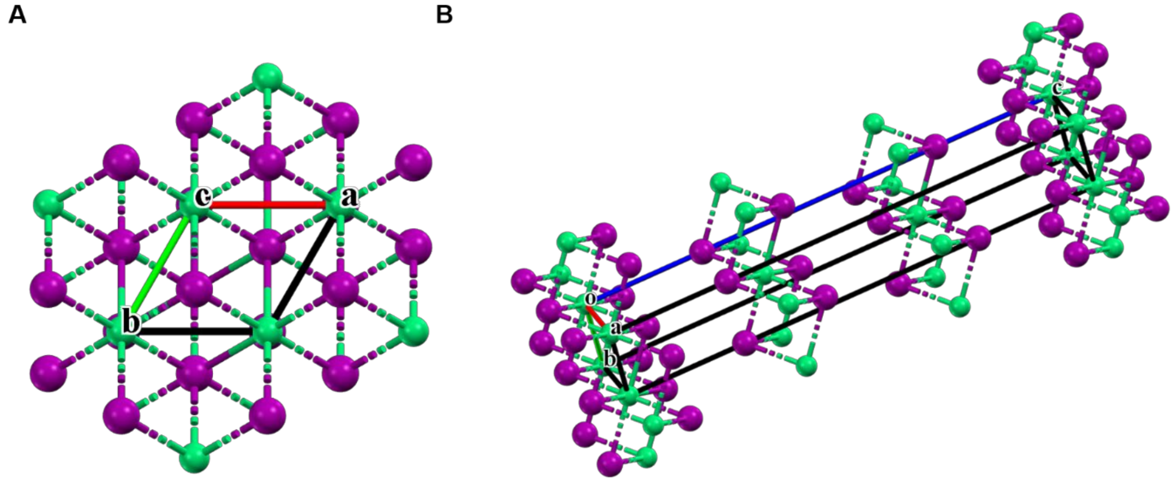


**Fig. S93. Visualization of the packed crystal structure of $NiI_2$.** A) The packed crystal is visualized along c*. B) To better illustrate the 3D structure, packed cell is visualized employing free-style orientation.

Space group: R -3 m (166)

Crystal system: Trigonal (Hexagonal axes)

Cell parameters: a=**3.89500 Å**, c= **19.63000 Å**

Density [g·cm$^{-3}$] = 5.83000

Hamaker Constant [eV] = **1.17**

Molecular Mass [g·mol$^{-1}$] = 312.5

$f_{Pauling}$ = **0.131**

Band gap [eV] = 1.8

$$\varepsilon_\infty = \begin{pmatrix} 6.4 & & \\ & 6.4 & \\ & & 5.8 \end{pmatrix}$$

$E_{total}$ $^{theory\ (RPA)}$ [meV·Å$^{-2}$] = **N/A**

$E_{total}$ $^{theory\ (corrected\ rVV10)}$ [meV·Å$^{-2}$] = **17.75**

$$\varepsilon(0) = \begin{pmatrix} 8 & & \\ & 8 & \\ & & 6.2 \end{pmatrix}$$

$E_{total}$ $^{empirical}$ [meV·Å$^{-2}$] = **13.0**

$E_{dispervise}$ $^{empirical}$ [meV·Å$^{-2}$] = **11.3**

**Notes**:

Crystal structure is given in ref.[335] Band gap is reported in ref.[336] Dielectric constants and position of infrared bands are reported in ref.[337]

# Crystal no. 71. $NiPS_3$

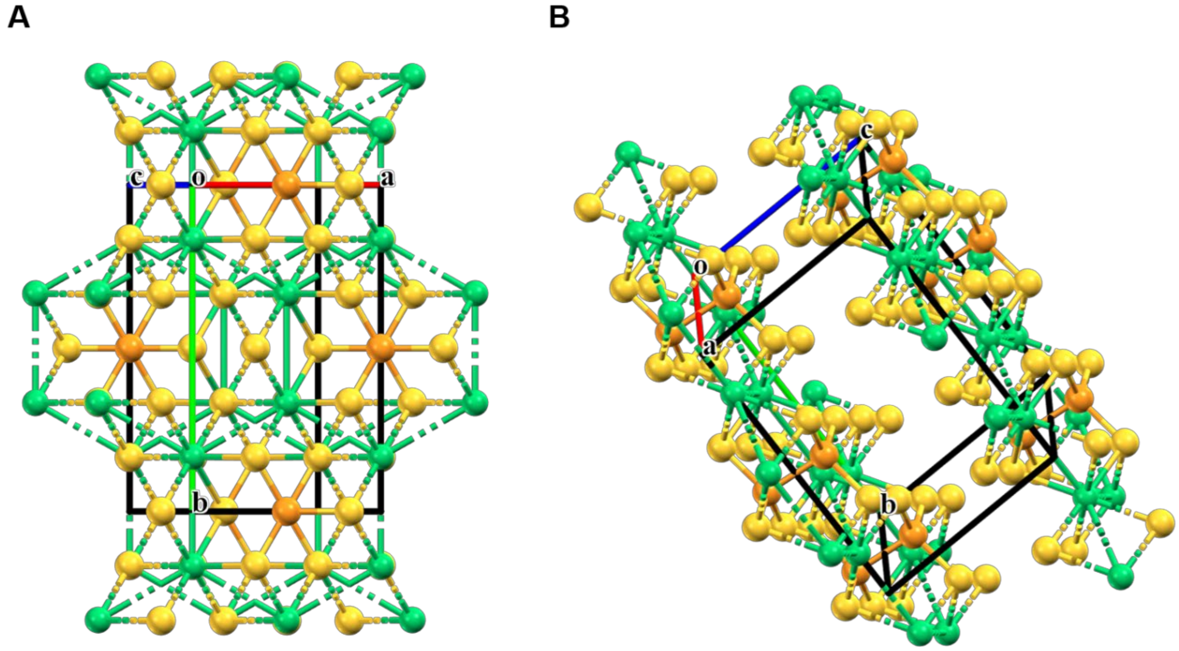


**Fig. S94. Visualization of the packed crystal structure of NiPS3.** A) The packed crystal is visualized along c*. B) To better illustrate the 3D structure, packed cell is visualized employing free-style orientation.

Density [g·cm$^{-3}$] = 3.33

Molecular Mass [g·mol$^{-1}$] = 185.9

Band gap [eV] = 1.6

$$\varepsilon_\infty = \begin{pmatrix} 8.8 & & \\ & 8.8 & \\ & & 6.4 \end{pmatrix}$$

N/A

Hamaker Constant [eV] = **1.525**

$f_{Pauling}$ = **0.106**

$E_{total}^{\text{theory (RPA)}}$ [meV·Å$^{-2}$] = **N/A**
$E_{total}^{\text{theory (corrected rVV10)}}$ [meV·Å$^{-2}$] = **16.30**

$E_{total}^{\text{empirical}}$ [meV·Å$^{-2}$] = **16.4**

$E_{dispervise}^{\text{empirical}}$ [meV·Å$^{-2}$] = **14.7**

**Notes**:
Density is calculated from XRD data in ref.[338] Dielectric function of $NiPS_3$ is given in section 12. Anisotropy is ignored. Ionicity refers to the ionicity of Ni-S bond.

# Crystal no. 72. $NiTe_2$

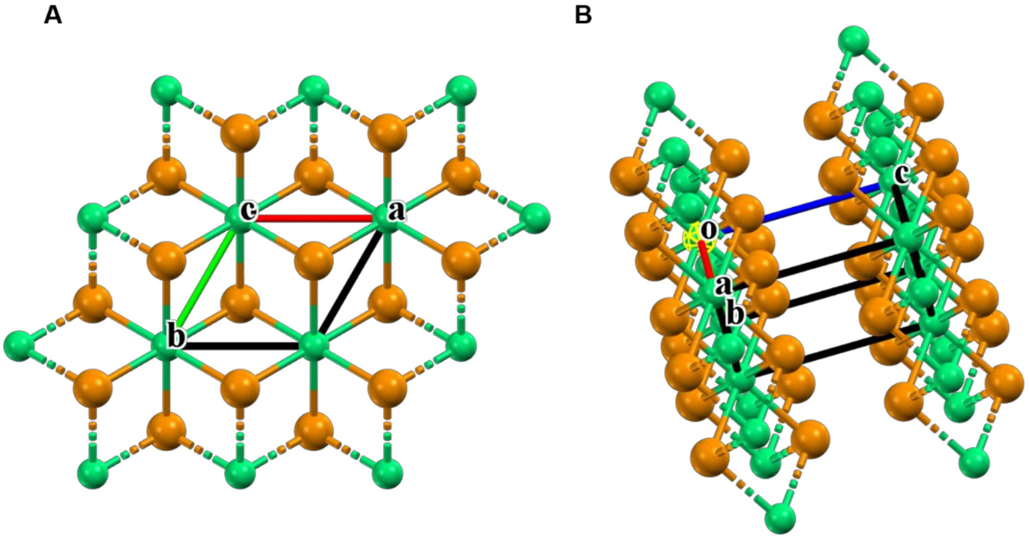


**Fig. S95. Visualization of the packed crystal structure of $NiTe_2$.** A) The packed crystal is visualized along c*. B) To better illustrate the 3D structure, packed cell is visualized employing free-style orientation.

Space group: P -3 m 1 (164)

Crystal system: Trigonal (Hexagonal axes)

Cell parameters: a=**3.86900 Å**, c= **5.30800 Å**

Density [g·cm$^{-3}$] = 7.57400

Hamaker Constant [eV] = **2.11**

Molecular Mass [g·mol$^{-1}$] = 313.9

$f_{Pauling}$ = **0.009**

Band gap [eV] = N/A

N/A

$E_{total}$ $^{theory\ (RPA)}$ [meV·Å$^{-2}$] = **N/A**

$E_{total}$ $^{theory\ (corrected\ rVV10)}$ [meV·Å$^{-2}$] = **31.68**

N/A

$E_{total}$ $^{empirical}$ [meV·Å$^{-2}$] = **20.5**

$E_{dispervise}$ $^{empirical}$ [meV·Å$^{-2}$] = **20.3**

**Notes**:
Crystal structure is given in ref.[339] Dielectric function of $NiTe_2$ is given in section 12.

# Crystal no. 73. PbFBr

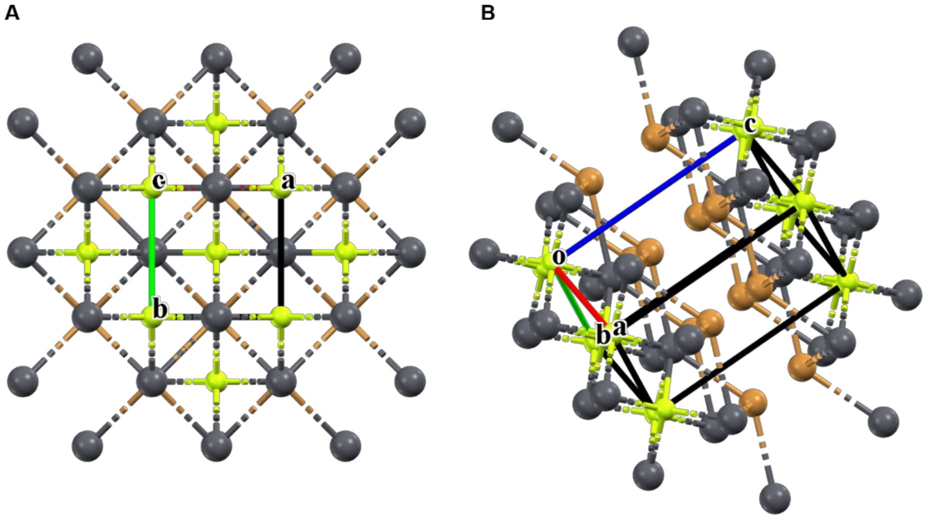


**Fig. S96. Visualization of the packed crystal structure of PbFBr.** A) The packed crystal is visualized along c*. B) To better illustrate the 3D structure, packed cell is visualized employing free-style orientation.

Space group: P 4/n m m (129)

Crystal system: Tetragonal

Cell parameters: a=**4.18000 Å**, c= **7.59000 Å**

Density [g·cm$^{-3}$] = 7.66500

Hamaker Constant [eV] = **1.55**

Molecular Mass [g·mol$^{-1}$] = 306.1

$f_{Pauling}$ = **0.294**

Band gap [eV] = 4.2

$$\varepsilon_{\infty} = \begin{pmatrix} 5.3 & & \\ & 5.3 & \\ & & 4.6 \end{pmatrix}$$

$E_{total}$ $^{theory (RPA)}$ [meV·Å$^{-2}$] = **N/A**

$E_{total}$ $^{theory (corrected rVV10)}$ [meV·Å$^{-2}$] = **20.13**

$$\varepsilon(0) = \begin{pmatrix} 25.2 & & \\ & 25.2 & \\ & & N/A \end{pmatrix}$$

$E_{total}$ $^{empirical}$ [meV·Å$^{-2}$] = **21.1**

$E_{dispervise}$ $^{empirical}$ [meV·Å$^{-2}$] = **14.9**

**Notes**:
Crystal structure is given in ref.[340] Band gap and electronic dielectric constant (in-plane) are estimated from the reflectance spectrum in ref.[341] Electronic dielectric constant (out-of-plane) is an approximation, assuming a birefringency similar to PbFCl. Static dielectric constant is given in ref.[342] Ionicity is the average ionicity of Pb-F and Pb-Br bonds.

# Crystal no. 74. PbFCl

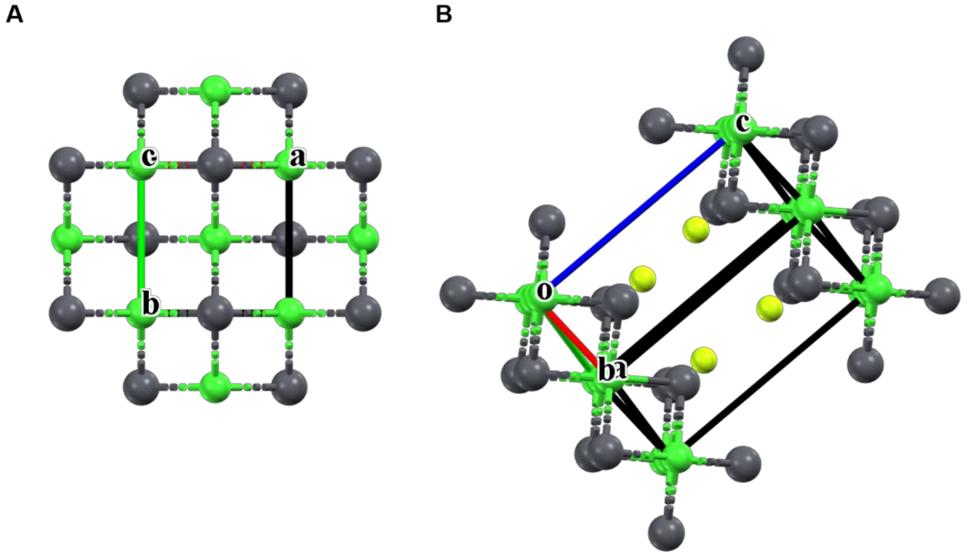


**Fig. S97. Visualization of the packed crystal structure of PbFCl.** A) The packed crystal is visualized along c*. B) To better illustrate the 3D structure, packed cell is visualized employing free-style orientation.

Space group: P 4/n m m (129)

Crystal system: Tetragonal

Cell parameters: a=**4.09000 Å**, c= **7.21000 Å**

Density [g·cm$^{-3}$] = 7.20400

Hamaker Constant [eV] = **1.30**

Molecular Mass [g·mol$^{-1}$] = 261.65

$f_{Pauling}$ = **0.485**

Band gap [eV] = 5

$$\varepsilon_\infty = \begin{pmatrix} 4.3 & & \\ & 4.3 & \\ & & 3.6 \end{pmatrix}$$

$E_{total}$ $^{theory (RPA)}$ [meV·Å$^{-2}$] = **N/A**
$E_{total}$ $^{theory (corrected rVV10)}$ [meV·Å$^{-2}$] = **24.62**

$$\varepsilon(0) = \begin{pmatrix} 12 & & \\ & 12 & \\ & & N/A \end{pmatrix}$$

$E_{total}$ $^{empirical}$ [meV·Å$^{-2}$] = **24.4**

$E_{dispervise}$ $^{empirical}$ [meV·Å$^{-2}$] = **12.6**

**Notes**:
Crystal structure is given in ref.[343] Band gap and electronic dielectric constant (in-plane) are estimated from the reflectance spectrum in ref.[341] Electronic dielectric constant (out-of-plane) and static dielectric constant (in-plane) are reported in ref.[342] Ionicity is for Pb-bond.

# Crystal no. 75. $PbI_2$

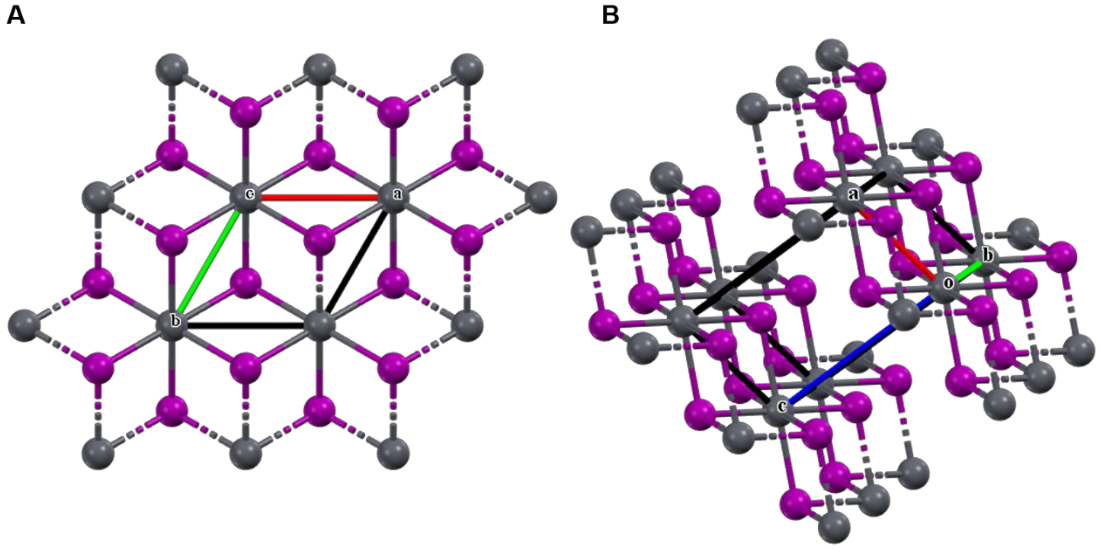


**Fig. S98. Visualization of the packed crystal structure of $PbI_2$.** A) The packed crystal is visualized along c*. B) To better illustrate the 3D structure, packed cell is visualized employing free-style orientation.

Space group: P -3 m 1 (164)

Crystal system: Trigonal (Hexagonal axes)

Cell parameters: a=**4.59000 Å**, c= **6.78000 Å**

Density [g·cm$^{-3}$] = 6.18800

Hamaker Constant [eV] = **1.17**

Molecular Mass [g·mol$^{-1}$] = 461.0

$f_{Pauling}$ = **0.027**

Band gap [eV] = 2.35

$$\varepsilon_\infty = \begin{pmatrix} 6.5 & & \\ & 6.5 & \\ & & 5.9 \end{pmatrix}$$

$E_{total}$ $^{theory\ (RPA)}$ [meV·Å$^{-2}$] = **N/A**
$E_{total}$ $^{theory\ (corrected\ rVV10)}$ [meV·Å$^{-2}$] = **10.43**

$$\varepsilon(0) = \begin{pmatrix} 26.8 & & \\ & 26.8 & \\ & & 9.3 \end{pmatrix}$$

$E_{total}$ $^{empirical}$ [meV·Å$^{-2}$] = **11.6**

$E_{dispervise}$ $^{empirical}$ [meV·Å$^{-2}$] = **11.3**

**Notes**:
Crystal structure is reported in ref.[344] Dielectric function of $PbI_2$ is given in section 12.

# Crystal no. 76. $PtS_2$

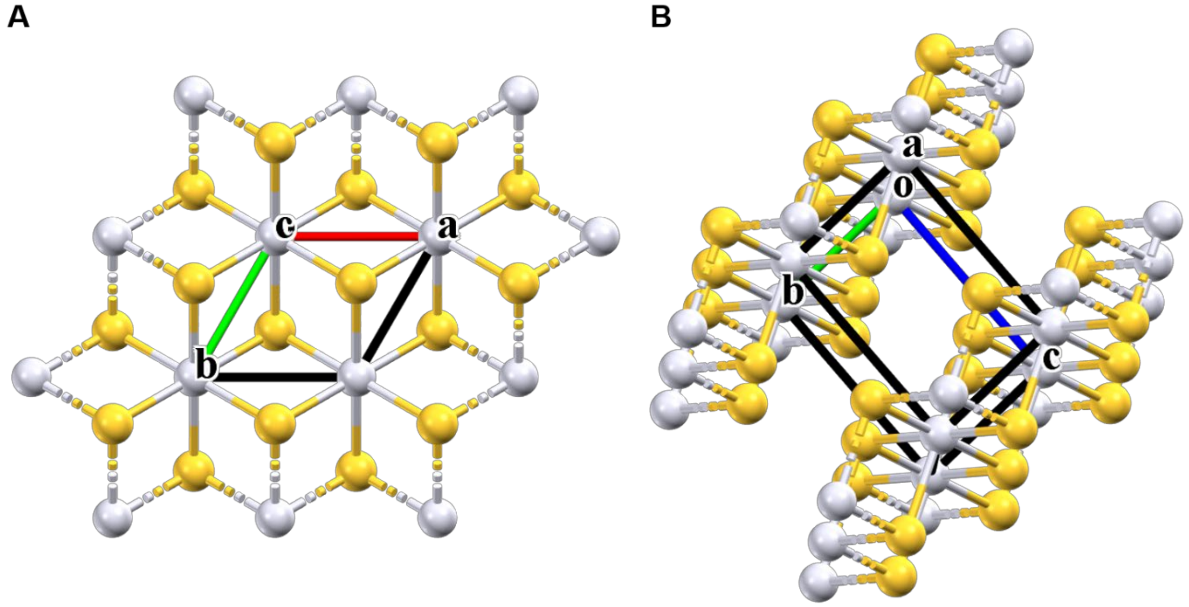


**Fig. S99. Visualization of the packed crystal structure of $PtS_2$.** A) The packed crystal is visualized along c*. B) To better illustrate the 3D structure, packed cell is visualized employing free-style orientation.

Space group: P -3 m 1 (164)

Crystal system: Trigonal (Hexagonal axes)

Cell parameters: a=**3.54320 Å**, c= **5.03880 Å**

Density [g·cm$^{-3}$] = 7.85700

Hamaker Constant [eV] = **1.93**

Molecular Mass [g·mol$^{-1}$] = 259.2

$f_{Pauling}$ = **0.022**

Band gap [eV] = 0.95

$$\varepsilon_{\infty} = \begin{pmatrix} 17.4 & & \\ & 17.4 & \\ & & 9.9 \end{pmatrix}$$

N/A

$E_{total}$ $^{theory\ (RPA)}$ [meV·Å$^{-2}$] = **20.55**

$E_{total}$ $^{theory\ (corrected\ rVV10)}$ [meV·Å$^{-2}$] = **21.78**

$E_{total}$ $^{empirical}$ [meV·Å$^{-2}$] = **19.0**

$E_{dispervise}$ $^{empirical}$ [meV·Å$^{-2}$] = **18.6**

**Notes**:
Crystal structure is given in ref.[345] Band gap and electronic dielectric constants are reported in ref.[346]

# Crystal no. 77. $PtSe_2$

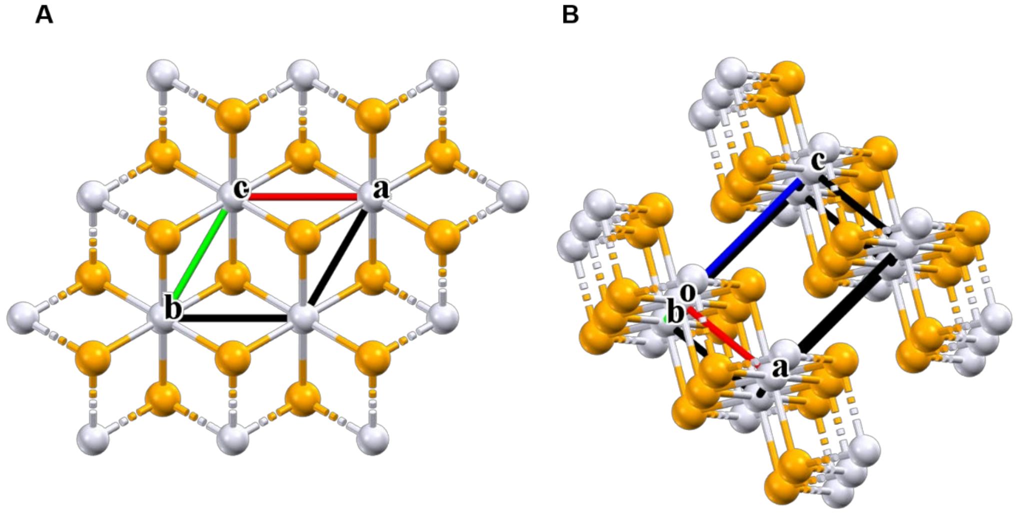


**Fig. S100. Visualization of the packed crystal structure of $PtSe_2$.** A) The packed crystal is visualized along c*. B) To better illustrate the 3D structure, packed cell is visualized employing free-style orientation.

Space group: P -3 m 1 (164)

Crystal system: Trigonal (Hexagonal axes)

Cell parameters: a=**3.72780 Å**, c= **5.03130 Å**

Density [$g \cdot cm^{-3}$] = 9.68000

Hamaker Constant [eV] = **2.15**

Molecular Mass [$g \cdot mol^{-1}$] = 353

$f_{Pauling}$ = **0.018**

Band gap [eV] = N/A

N/A

$E_{total}$ $^{theory\ (RPA)}$ [$meV \cdot Å^{-2}$] = **19.05**
$E_{total}$ $^{theory\ (corrected\ rVV10)}$ [$meV \cdot Å^{-2}$] = **23.23**

N/A

$E_{total}$ $^{empirical}$ [$meV \cdot Å^{-2}$] = **21.07**

$E_{dispervise}$ $^{empirical}$ [$meV \cdot Å^{-2}$] = **20.70**

**Notes**:
Crystal structure is given in ref.[347] Hamaker constant is estimated from the dielectric functions reported in ref.[348] (between 1.2 and 6 eV), ref.[349] (between 8 and 30 eV). Above 30 eV, atomic scattering factors are used to calculate the complex refractive indices.[46, 47] The rest is interpolation. Bands due to the free electrons are ignored as due to low career concentration,[350] and therefore minor role of them in vdW forces.[16]

# Crystal no. 78. $PtTe_2$

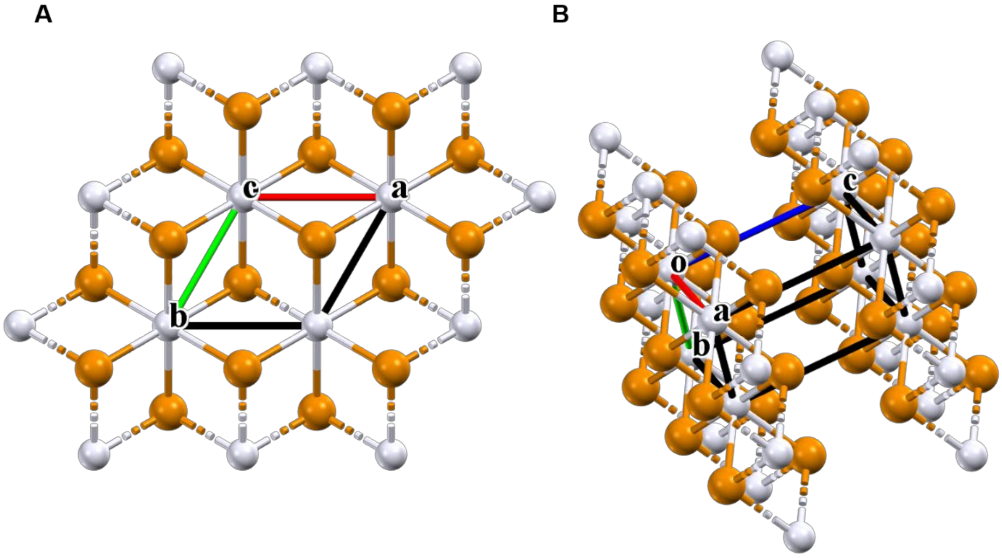


**Fig. S101. Visualization of the packed crystal structure of $PtTe_2$.** A) The packed crystal is visualized along c*. B) To better illustrate the 3D structure, packed cell is visualized employing free-style orientation.

Space group: P -3 m 1 (164)

Crystal system: Trigonal (Hexagonal axes)

Cell parameters: a=**4.02590 Å**, c= **5.22090 Å**

Density [g·cm$^{-3}$] = 10.20300

Hamaker Constant [eV] = **2.64**

Molecular Mass [g·mol$^{-1}$] = 450.3

$f_{Pauling}$ = **0.008**

Band gap [eV] = N/A

N/A

$E_{total}$ $^{theory\ (RPA)}$ [meV·Å$^{-2}$] = **N/A**
$E_{total}$ $^{theory\ (corrected\ rVV10)}$ [meV·Å$^{-2}$] = **26.73**

N/A

$E_{total}$ $^{empirical}$ [meV·Å$^{-2}$] = **25.62**

$E_{dispervise}$ $^{empirical}$ [meV·Å$^{-2}$] = **25.41**

**Notes**:
Crystal structure is given in ref.[347] Hamaker constant is approximated using the dielectric function derived from theoretical loss function in ref.[351] The intensity of the loss function was normalized such that at high photon energy it matches the optical constants derived from atomic scattering factors of Pt and Te and corresponding mass density, and that the loss function matches the metallic nature of $PtTe_2$. We estimated the plasma frequency and damping factor of 6.15 and 0.168 eV. This correspond to electrical resistivity of 33 μΩ·cm which is in agreement with experimental data.[352]

# Crystal no. 79. $ReS_2$

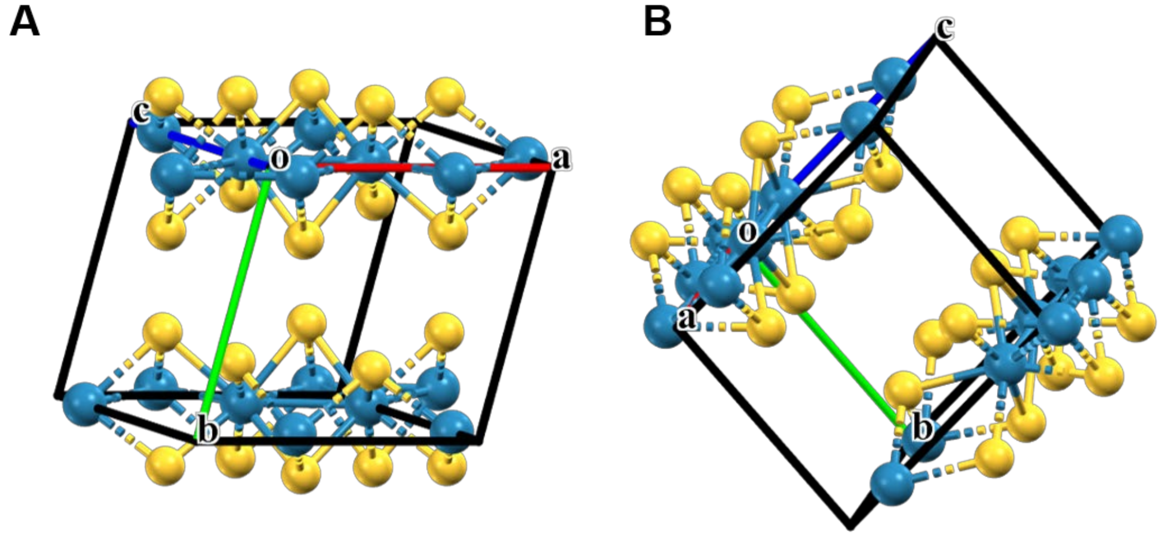


**Fig. S102. Visualization of the packed crystal structure of $ReS_2$.** A) The packed crystal is visualized along c*. B) To better illustrate the 3D structure, packed cell is visualized employing free-style orientation.

Density [g·cm$^{-3}$] = 7.58

Molecular Mass [g·mol$^{-1}$] = 250.3

Band gap [eV] = 1.47

$$\varepsilon_\infty = \begin{pmatrix} 13 & & \\ & 13 & \\ & & 5.8 \end{pmatrix}$$

N/A

Hamaker Constant [eV] = **1.83**

$f_{Pauling}$ = **0.109**

$E_{total}$ $^{theory\ (RPA)}$ [meV·Å$^{-2}$] = **N/A**
$E_{total}$ $^{theory\ (corrected\ rVV10)}$ [meV·Å$^{-2}$] = **18.15**

$E_{total}$ $^{empirical}$ [meV·Å$^{-2}$] = **19.8**

$E_{dispervise}$ $^{empirical}$ [meV·Å$^{-2}$] = **17.6**

**Notes**:
Density is calculated from XRD data in ref.[338] Band gap is reported in ref.[353] Electronic dielectric constant (in-plane) is approximated using the Eqs. (7) and (S18). Electronic dielectric (out-of-plane) is a theoretical value.[354]

# Crystal no. 80. $ReSe_2$

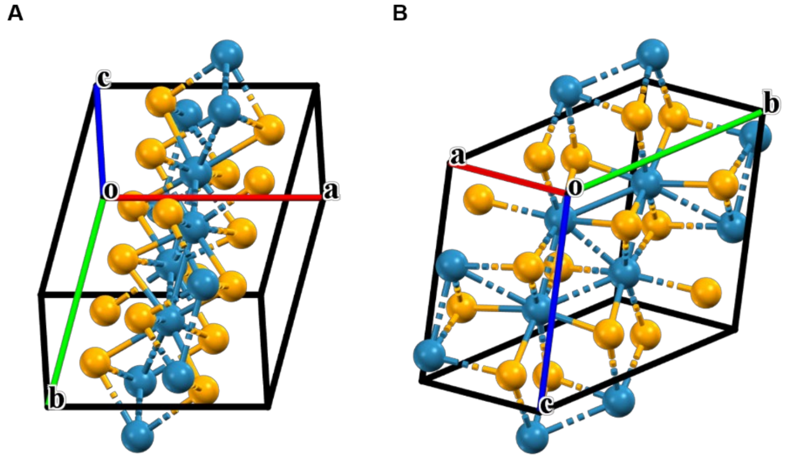


**Fig. S103. Visualization of the packed crystal structure of $ReSe_2$.** A) The packed crystal is visualized along c*. B) To better illustrate the 3D structure, packed cell is visualized employing free-style orientation.

Space group: P -1 (2)

Crystal system: Triclinic (Anorthic)

Cell parameters: a=**6.72720 Å**, b= **6.60650 Å** c= **6.71960 Å** α= **118.930°** β= **91.820 °** γ= **104.930 °**

Density [g·cm$^{-3}$] = 9.20900

Hamaker Constant [eV] = **1.77**

Molecular Mass [g·mol$^{-1}$] = 344.1

$f_{Pauling}$ = **0.1**

Band gap [eV] = 1.19

$$\varepsilon_\infty = \begin{pmatrix} 13.7 & & \\ & 13.7 & \\ & & 6.3 \end{pmatrix}$$

N/A

$E_{total}^{theory\ (RPA)}$ [meV·Å$^{-2}$] = **N/A**

$E_{total}^{theory\ (corrected\ rVV10)}$ [meV·Å$^{-2}$] = **20.00**

$E_{total}^{empirical}$ [meV·Å$^{-2}$] = **18.9**

$E_{dispervise}^{empirical}$ [meV·Å$^{-2}$] = **17.0**

**Notes**:

Crystal structure is given in ref.[355] Band gap is reported in ref.[356] Electronic dielectric constant (in-plane) is estimated using the refractive indices in terahertz region, assuming a small polarization in infrared region.[357] In-plane anisotropy is ignored. Electronic dielectric constant (out-of-plane) is a theoretical value reported in Materials Project webpage.[50]

# Crystal no. 81. $RuCl_3$

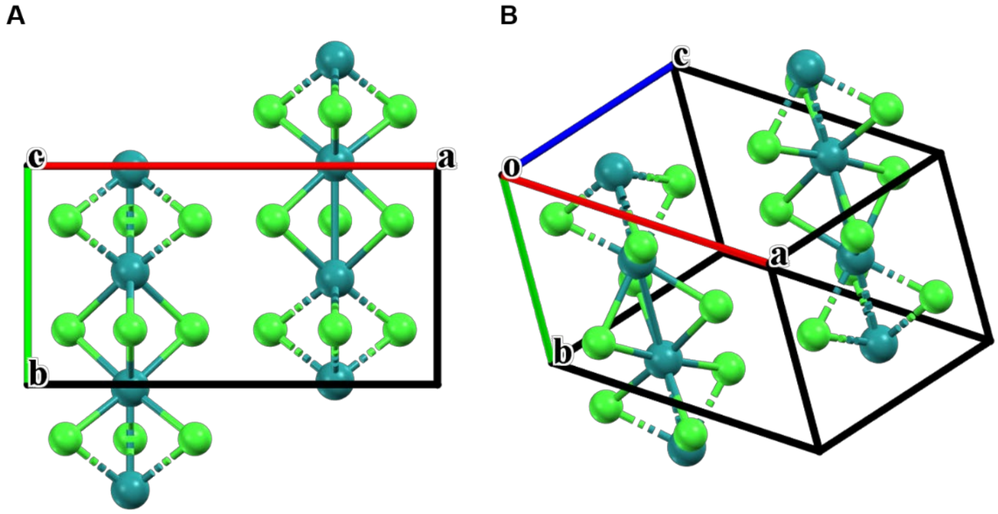


**Fig. S104. Visualization of the packed crystal structure of $RuCl_3$.** A) The packed crystal is visualized along c*. B) To better illustrate the 3D structure, packed cell is visualized employing free-style orientation.

Space group: P m m n (59)

Crystal system: Orthorhombic

Cell parameters: a=**10.57570 Å**, b= **5.63380 Å** c= **6.10590 Å**

Density [g·cm$^{-3}$] = 3.78700

Hamaker Constant [eV] = **1.55**

Molecular Mass [g·mol$^{-1}$] = 207.4

$f_{Pauling}$ = **0.206**

Band gap [eV] = 1.9

$$\varepsilon_\infty = \begin{pmatrix} 8.2 & & \\ & 8.2 & \\ & & N/A \end{pmatrix}$$

N/A

$E_{total}$ $^{theory\ (RPA)}$ [meV·Å$^{-2}$] = **N/A**

$E_{total}$ $^{theory\ (corrected\ rVV10)}$ [meV·Å$^{-2}$] = **13.53**

$E_{total}$ $^{empirical}$ [meV·Å$^{-2}$] = **18.8**

$E_{dispervise}$ $^{empirical}$ [meV·Å$^{-2}$] = **14.9**

**Notes**:

Crystal structure is given in ref.[358] Band gap is reported in ref.[359] Electronic dielectric constant is approximated using the Eqs. (7) and (S18). Anisotropy is ignored.

# Crystal no. 82. $Sb_2O_3$

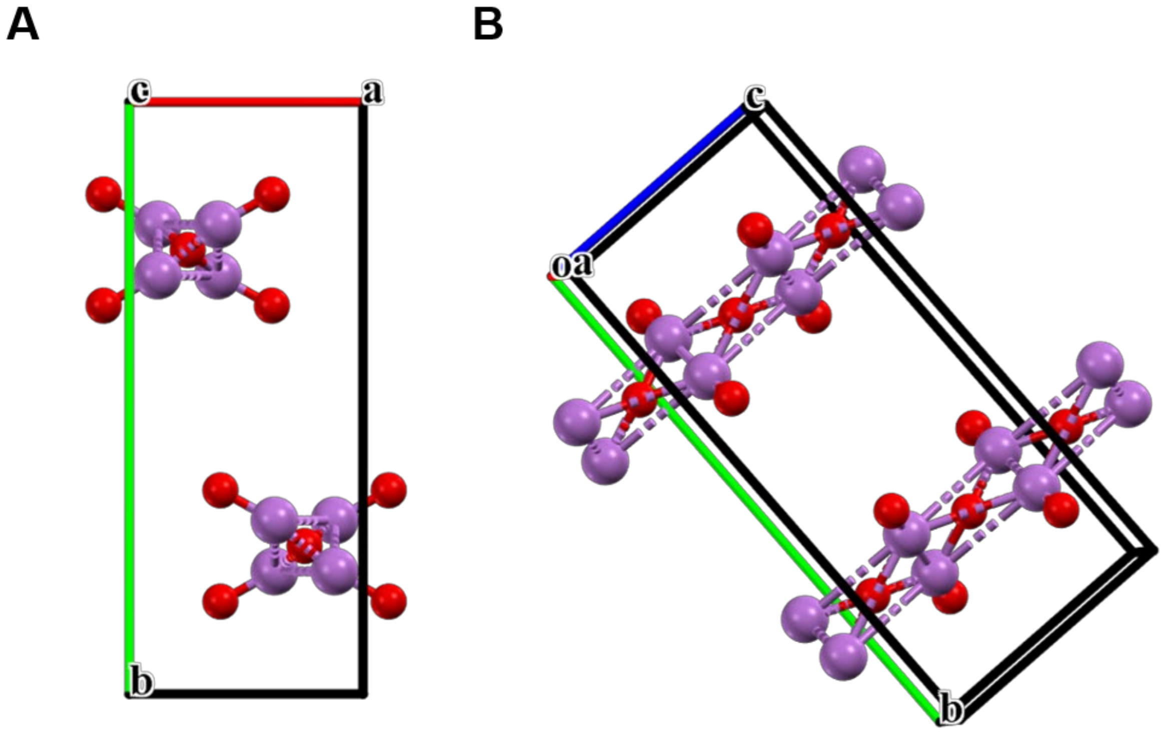


**Fig. S105. Visualization of the packed crystal structure of $Sb_2O_3$.** A) The packed crystal is visualized along c*. B) To better illustrate the 3D structure, packed cell is visualized employing free-style orientation.

Density [g·cm$^{-3}$] = 5.827

Molecular Mass [g·mol$^{-1}$] = 291.5

Band gap [eV] = 3.25

$$\varepsilon_\infty = \begin{pmatrix} 5 & & \\ & 5 & \\ & & 4.5 \end{pmatrix}$$

N/A

Hamaker Constant [eV] = **1.25**

$f_{Pauling}$ = **0.383**

$E_{total}$ $^{theory\ (RPA)}$ [meV·Å$^{-2}$] = **N/A**
$E_{total}$ $^{theory\ (corrected\ rVV10)}$ [meV·Å$^{-2}$] = **N/A**

$E_{total}$ $^{empirical}$ [meV·Å$^{-2}$] = **19.6**

$E_{dispervise}$ $^{empirical}$ [meV·Å$^{-2}$] = **12.1**

**Notes**:
Density is calculated from XRD data in ref.[360] Bang gap is reported in ref.[361] Electronic dielectric constants are approximated based on the refractive indices reported in ref.[234]

# Crystal no. 83. $Sb_2Se_3$

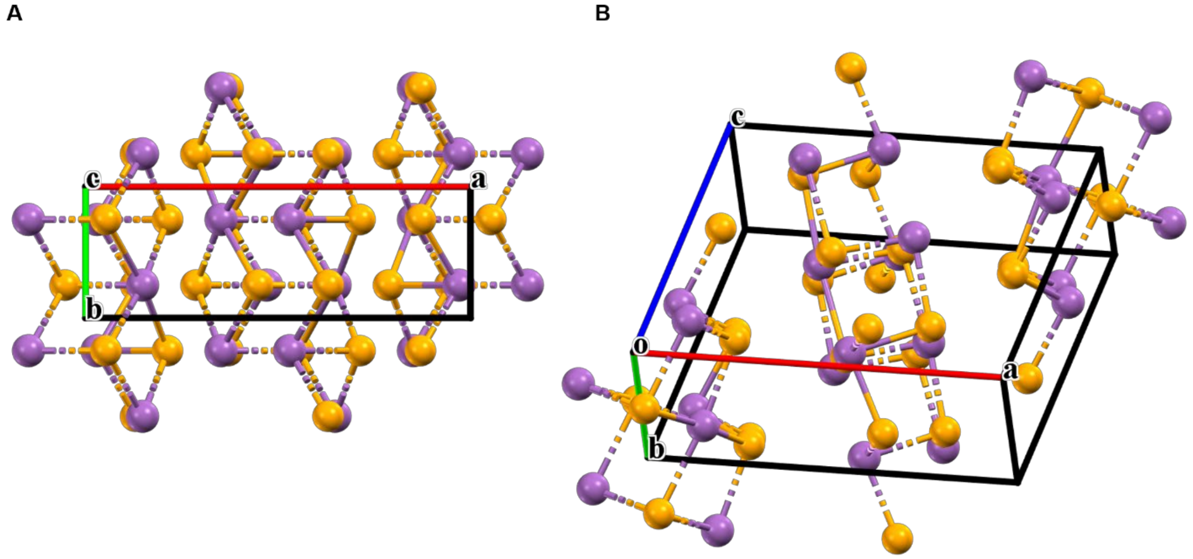


**Fig. S106. Visualization of the packed crystal structure of $Sb_2Se_3$.** A) The packed crystal is visualized along c*. B) To better illustrate the 3D structure, packed cell is visualized employing free-style orientation.

Space group: P n m a (62)

Crystal system: Orthorhombic

Cell parameters: a=**11.68000 Å**, b= **3.98000 Å** c= **11.58000 Å**

Density [g·cm$^{-3}$] = 5.92700

Hamaker Constant [eV] = **1.97**

Molecular Mass [g·mol$^{-1}$] = 480.8

$f_{Pauling}$ = **0.061**

Band gap [eV] = 1.14

$$\varepsilon_\infty = \begin{pmatrix} 19.6 & & \\ & 19.6 & \\ & & 13.5 \end{pmatrix}$$

$E_{total}$ $^{theory\ (RPA)}$ [meV·Å$^{-2}$] = **N/A**

$E_{total}$ $^{theory\ (corrected\ rVV10)}$ [meV·Å$^{-2}$] = **19.01**

$$\varepsilon(0) = \begin{pmatrix} 124.1 & & \\ & 124.1 & \\ & & 16.7 \end{pmatrix}$$

$E_{total}$ $^{empirical}$ [meV·Å$^{-2}$] = **20.1**

$E_{dispervise}$ $^{empirical}$ [meV·Å$^{-2}$] = **18.9**

**Notes**:

Crystal structure is given in ref.[362] Dielectric function of $Sb_2Se_3$ is given in our previous work.[16] Theoretical binding energy refers to the value for $Sb_2TeSe_2$.

# Crystal no. 84. $Sb_2Te_3$

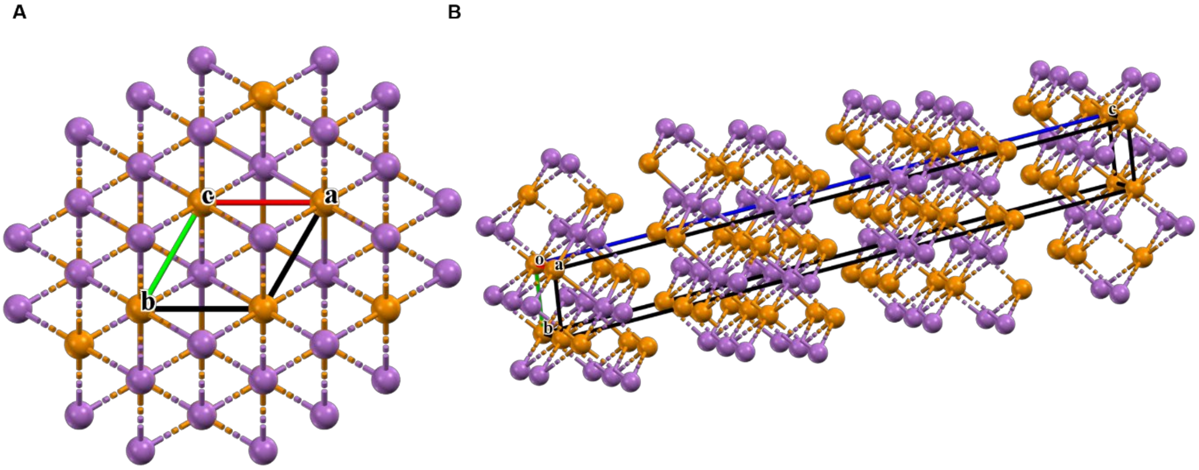


**Fig. S107. Visualization of the packed crystal structure of $Sb_2Te_3$.** A) The packed crystal is visualized along c*. B) To better illustrate the 3D structure, packed cell is visualized employing free-style orientation.

Space group: R -3 m (166)

Crystal system: Trigonal (Hexagonal axes)

Cell parameters: a== **4.25000 Å**, c= **29.96000 Å**

Density [g·cm$^{-3}$] = 6.65700

Hamaker Constant [eV] = **1.746**

Molecular Mass [g·mol$^{-1}$] = 626.3

$f_{Pauling}$ = **0.0006**

Band gap [eV] = 0.21

$$\varepsilon_\infty = \begin{pmatrix} 51 & & \\ & 51 & \\ & & 32.5 \end{pmatrix}$$

$E_{total}^{\text{theory (RPA)}}$ [meV·Å$^{-2}$] = **N/A**

$E_{total}^{\text{theory (corrected rVV10)}}$ [meV·Å$^{-2}$] = **19.8**

$$\varepsilon(0) = \begin{pmatrix} 168 & & \\ & 168 & \\ & & 36.5 \end{pmatrix}$$

$E_{total}^{\text{empirical}}$ [meV·Å$^{-2}$] = **16.8**

$E_{dispervise}^{\text{empirical}}$ [meV·Å$^{-2}$] = **16.8**

**Notes**:
Crystal structure is given in ref.[363] Band gap is reported in ref.[364] Dielectric constants and position of infrared bands are given in ref.[365] Also, Drude bands are included in dielectric function to calculate the Hamaker constant. For polarization parallel to plane, reflectivity data of Dordovic *et al.*[366] was analysed which results in plasma and scattering frequencies of 0.9 and 0.0237 eV, respectively. For polarization perpendicular to plane, it is assumed that the anisotropy is only caused by enhanced effective mass (about 2.25 times)[365], which leads to plasma and scattering frequencies of 0.6 and 0.0237 eV, respectively.

# Crystal no. 85. $SbI_3$

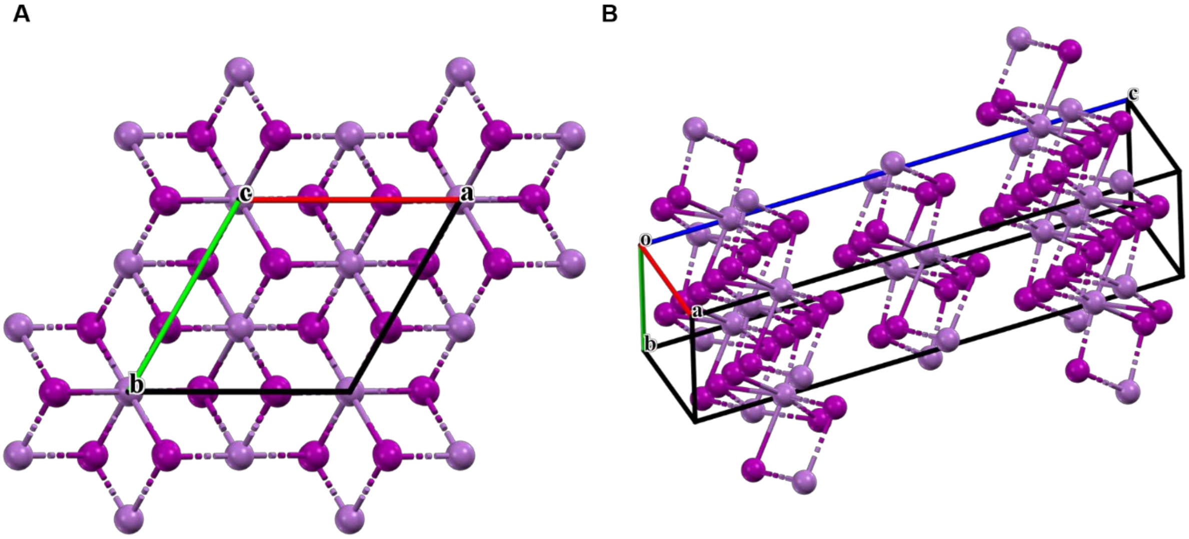


**Fig. S108. Visualization of the packed crystal structure of $SbI_3$.** A) The packed crystal is visualized along c*. B) To better illustrate the 3D structure, packed cell is visualized employing free-style orientation.

Space group: R -3 (148)

Crystal system: Trigonal (Hexagonal axes)

Cell parameters: a=**7.48000 Å**, c= **20.90000 Å**

Density [g·cm$^{-3}$] = 4.94300

Hamaker Constant [eV] = **1.047**

Molecular Mass [g·mol$^{-1}$] = 502.5

$f_{Pauling}$ = **0.115**

Band gap [eV] = 2.3

$$\varepsilon_\infty = \begin{pmatrix} 5.3 & & \\ & 5.3 & \\ & & 4.4 \end{pmatrix}$$

$$\varepsilon(0) = \begin{pmatrix} 16 & & \\ & 16 & \\ & & 8.9 \end{pmatrix}$$

$E_{total}^{\text{theory (RPA)}}$ [meV·Å$^{-2}$] = **N/A**

$E_{total}^{\text{theory (corrected rVV10)}}$ [meV·Å$^{-2}$] = **11.81**

$E_{total}^{\text{empirical}}$ [meV·Å$^{-2}$] = **11.4**

$E_{dispervise}^{\text{empirical}}$ [meV·Å$^{-2}$] = **10.1**

**Notes**:
Crystal structure is given in ref.[367] Band gap is reported in ref.[368] Dielectric constants are listed in ref.[369]

# Crystal no. 86. SnS

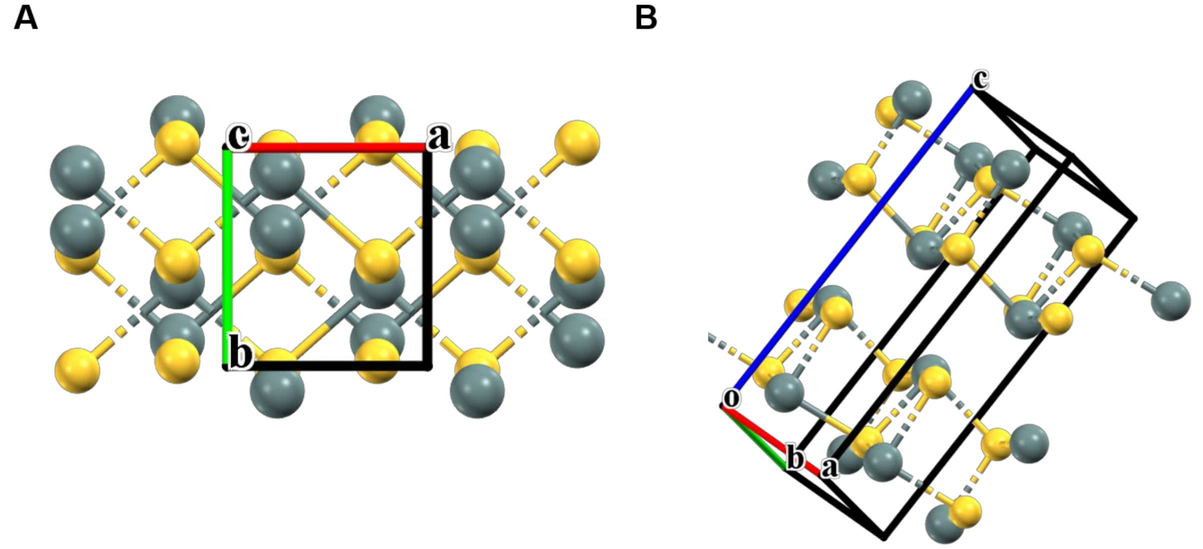


**Fig. S109. Visualization of the packed crystal structure of SnS.** A) The packed crystal is visualized along c*. B) To better illustrate the 3D structure, packed cell is visualized employing free-style orientation.

Space group: P m c n (62)

Crystal system: Orthorhombic

Cell parameters: a=**3.98000 Å**, b= **4.33000 Å** c= **11.18000 Å**

Density [g·cm$^{-3}$] = 5.19700

Hamaker Constant [eV] = **1.85**

Molecular Mass [g·mol$^{-1}$] = 150.8

$f_{Pauling}$ = **0.092**

Band gap [eV] = 1.1

$$\varepsilon_\infty = \begin{pmatrix} 15 & & \\ & 12 & \\ & & 12 \end{pmatrix}$$

E$_{total}$ $^{theory\ (RPA)}$ [meV·Å$^{-2}$] = **N/A**
E$_{total}$ $^{theory\ (corrected\ rVV10)}$ [meV·Å$^{-2}$] = **24.09**

$$\varepsilon(0) = \begin{pmatrix} 38 & & \\ & 29 & \\ & & 33 \end{pmatrix}$$

E$_{total}$ $^{empirical}$ [meV·Å$^{-2}$] = **19.7**

E$_{dispervise}$ $^{empirical}$ [meV·Å$^{-2}$] = **17.8**

**Notes**:
Crystal structure is given in ref.[370] Band gap is reported in ref.[371] Dielectric constants are reported in ref.[372]

# Crystal no. 87. $SnS_2$

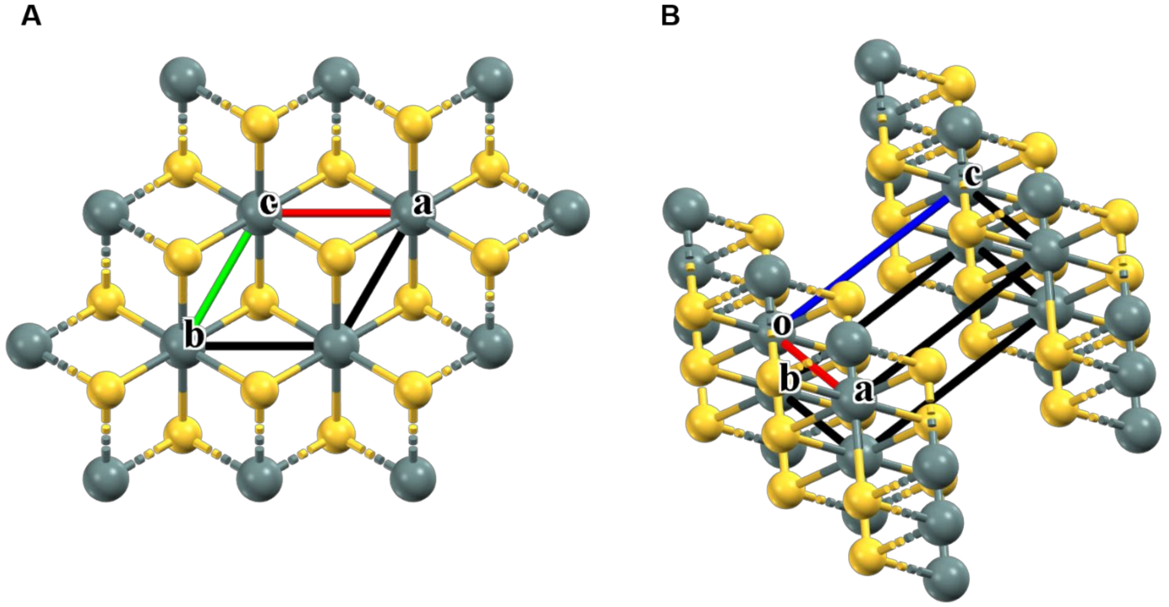


**Fig. S110. Visualization of the packed crystal structure of $SnS_2$.** A) The packed crystal is visualized along c*. B) To better illustrate the 3D structure, packed cell is visualized employing free-style orientation.

Space group: P -3 m 1 (164)

Crystal system: Trigonal (Hexagonal axes)

Cell parameters: a=**3.62000 Å**, c= **5.85000 Å**

Density [g·cm$^{-3}$] = 4.57300

Hamaker Constant [eV] = **1.520**

Molecular Mass [g·mol$^{-1}$] = 182.8

$f_{Pauling}$ = **0.092**

Band gap [eV] = 2.34

$$\varepsilon_\infty = \begin{pmatrix} 7.6 & & \\ & 7.6 & \\ & & 5.7 \end{pmatrix}$$

$E_{total}$ $^{theory\ (RPA)}$ [meV·Å$^{-2}$] = **N/A**
$E_{total}$ $^{theory\ (corrected\ rVV10)}$ [meV·Å$^{-2}$] = **15.64**

$$\varepsilon(0) = \begin{pmatrix} 17.8 & & \\ & 17.8 & \\ & & 6.2 \end{pmatrix}$$

$E_{total}$ $^{empirical}$ [meV·Å$^{-2}$] = **16.1**

$E_{dispervise}$ $^{empirical}$ [meV·Å$^{-2}$] = **14.6**

**Notes**:
Crystal structure is given in ref.[373] Band gap is reported in ref.[374] Dielectric constants and position of infrared bands are given in ref.[337]

# Crystal no. 88. $SnSe_2$

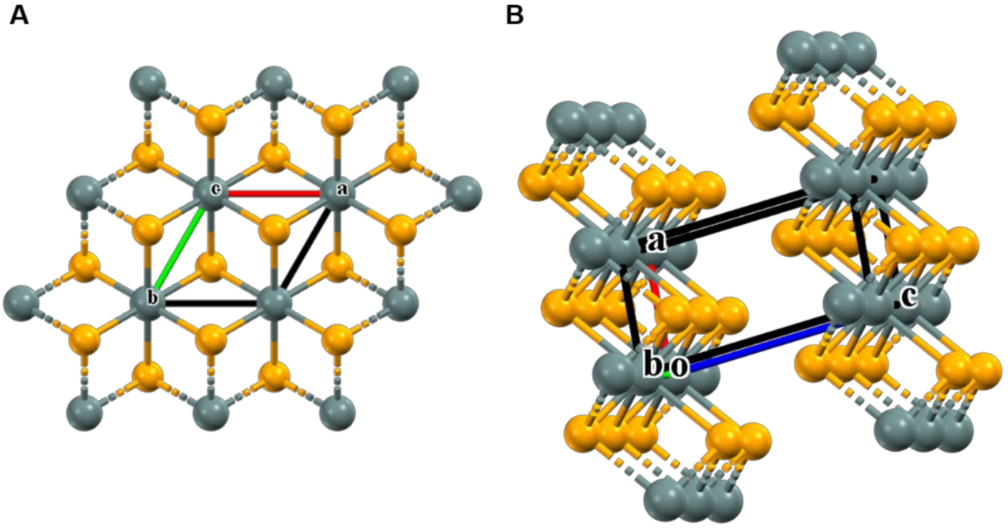


**Fig. S111. Visualization of the packed crystal structure of $SnSe_2$.** A) The packed crystal is visualized along c*. B) To better illustrate the 3D structure, packed cell is visualized employing free-style orientation.

Space group: P -3 m 1 (164)

Crystal system: Trigonal (Hexagonal axes)

Cell parameters: a=**3.81100 Å**, c= 6.13700 **Å**

Density [g·cm$^{-3}$] = 5.95100

Hamaker Constant [eV] = **1.42**

Molecular Mass [g·mol$^{-1}$] = 276.6

$f_{Pauling}$ = **0.083**

Band gap [eV] = 1.15

$$\varepsilon_\infty = \begin{pmatrix} 10.7 & & \\ & 10.7 & \\ & & 9.4 \end{pmatrix}$$

$$\varepsilon(0) = \begin{pmatrix} 23.2 & & \\ & 23.2 & \\ & & 10 \end{pmatrix}$$

$E_{total}^{theory\ (RPA)}$ [meV·Å$^{-2}$] = **N/A**

$E_{total}^{theory\ (corrected\ rVV10)}$ [meV·Å$^{-2}$] = **16.5**

$E_{total}^{empirical}$ [meV·Å$^{-2}$] = **14.9**

$E_{dispervise}^{empirical}$ [meV·Å$^{-2}$] = **13.6**

**Notes**:
Crystal structure is given in ref.[375] Band gap is reported in ref.[237] Dielectric constants and position of infrared bands are given in ref.[337]

# Crystal no. 89. SrFBr

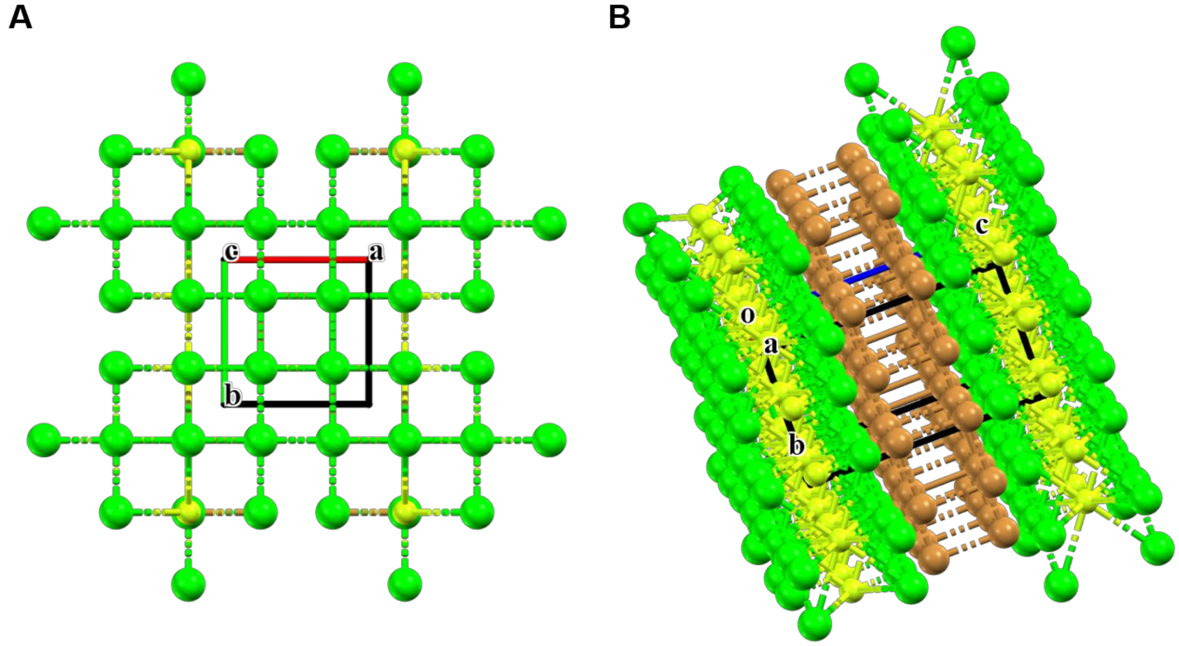


**Fig. S112. Visualization of the packed crystal structure of SrFBr.** A) The packed crystal is visualized along c*. B) To better illustrate the 3D structure, packed cell is visualized employing free-style orientation

Density [g·cm$^{-3}$] = 4.75

Molecular Mass [g·mol$^{-1}$] = 186.5

Band gap [eV] = 7.2

$$\varepsilon_\infty = \begin{pmatrix} 2.88 & & \\ & 2.88 & \\ & & N/A \end{pmatrix}$$

N/A

Hamaker Constant [eV] = **0.908**

$f_{Pauling} \approx$ **0.635**

$E_{total}$ $^{theory\ (RPA)}$ [meV·Å$^{-2}$] = **N/A**
$E_{total}$ $^{theory\ (corrected\ rVV10)}$ [meV·Å$^{-2}$] = **22.18**

$E_{total}$ $^{empirical}$ [meV·Å$^{-2}$] = **24.0**

$E_{dispervise}$ $^{empirical}$ [meV·Å$^{-2}$] = **8.74**

**Notes**:
Density is calculated from XRD data in ref.[338] Band gap is approximated from the optical density spectrum reported in ref.[376] Dielectric constant is given in ref.[342]

# Crystal no. 90. SrFCl

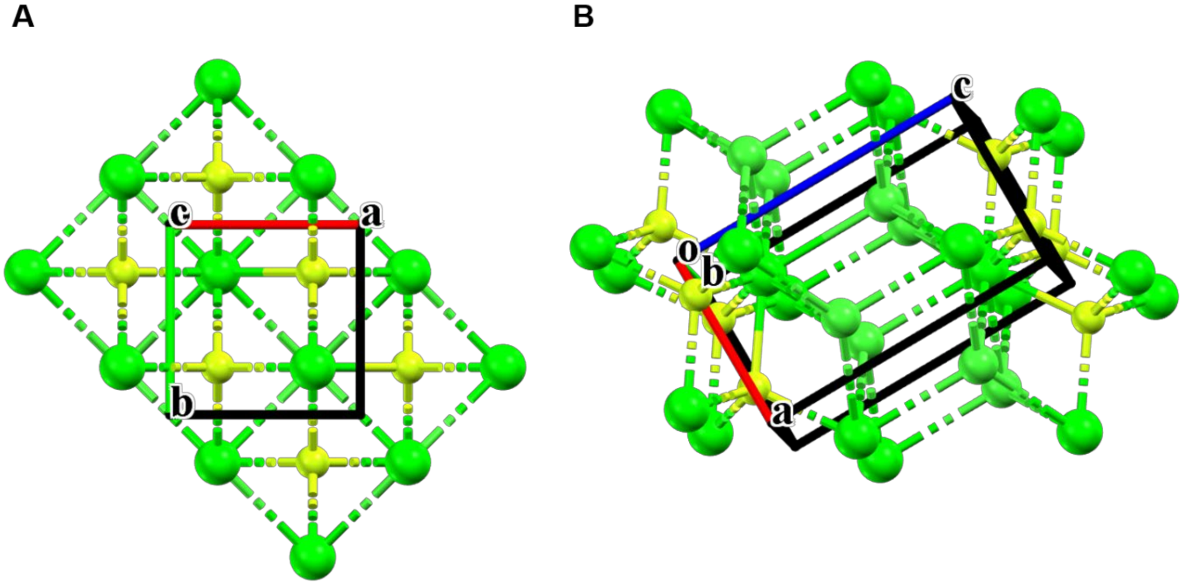


**Fig. S113. Visualization of the packed crystal structure of SrFCl.** A) The packed crystal is visualized along c*. B) To better illustrate the 3D structure, packed cell is visualized employing free-style orientation.

Space group: P 4/n m m (129)

Crystal system: Tetragonal

Cell parameters: a=**4.12590 Å**, c= **6.95790 Å**

Density [g·cm$^{-3}$] = 3.98400

Hamaker Constant [eV] = 0.77

Molecular Mass [g·mol$^{-1}$] = 142.1

$f_{Pauling}$ ≈ **0.705**

Band gap [eV] = 7

$$\varepsilon_\infty = \begin{pmatrix} 2.71 & & \\ & 2.71 & \\ & & 2.645 \end{pmatrix}$$

$$\varepsilon(0) = \begin{pmatrix} 8.2 & & \\ & 8.2 & \\ & & 7 \end{pmatrix}$$

$E_{total}$ $^{theory\ (RPA)}$ [meV·Å$^{-2}$] = **N/A**

$E_{total}$ $^{theory\ (corrected\ rVV10)}$ [meV·Å$^{-2}$] = **N/A**

$E_{total}$ $^{empirical}$ [meV·Å$^{-2}$] = **25.3**

$E_{dispervise}$ $^{empirical}$ [meV·Å$^{-2}$] = **7.45**

**Notes**:
Crystal structure is given in ref.[377] Band gap is approximated from the optical density spectrum reported in ref.[376] Dielectric constants are given in ref.[378] Ionicity refers to Sr-Cl bond's ionicity.

# Crystal no. 91. $TaS_2$

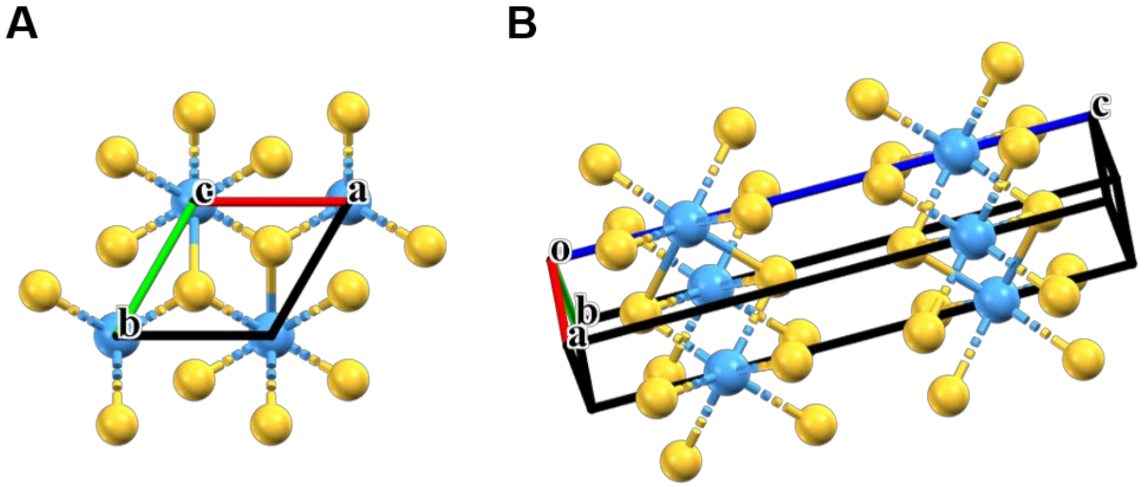


**Fig. S114. Visualization of the packed crystal structure of $TaS_2$.** A) The packed crystal is visualized along c*. B) To better illustrate the 3D structure, packed cell is visualized employing free-style orientation.

Density [$g \cdot cm^{-3}$] = 7.10

Molecular Mass [$g \cdot mol^{-1}$] = 245.1

Band gap [eV] = N/A

N/A

N/A

Hamaker Constant [eV] = **1.69**

$f_{Pauling}$ = **0.253**

$E_{total}^{theory\ (RPA)}$ [$meV \cdot Å^{-2}$] = **17.86**
$E_{total}^{theory\ (corrected\ rVV10)}$ [$meV \cdot Å^{-2}$] = **18.22**

$E_{total}^{empirical}$ [$meV \cdot Å^{-2}$] = **21.8**

$E_{dispervise}^{empirical}$ [$meV \cdot Å^{-2}$] = **16.3**

**Notes**:
Density is calculated form XRD data in ref.[379] Dielectric function of $TaS_2$ is given in section 12.

# Crystal no. 92. $TaSe_2$

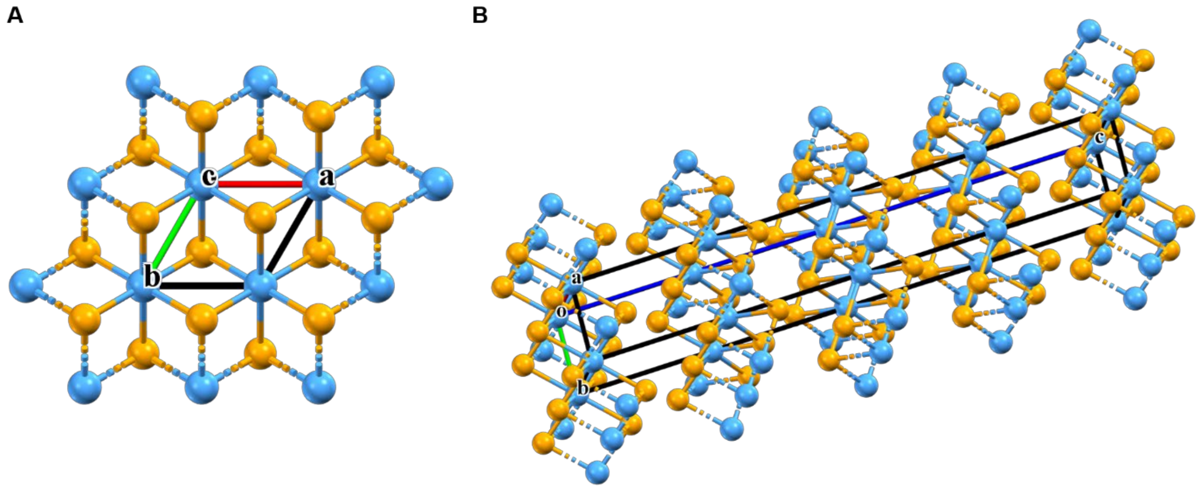


**Fig. S115. Visualization of the packed crystal structure of $TaSe_2$.** A) The packed crystal is visualized along c*. B) To better illustrate the 3D structure, packed cell is visualized employing free-style orientation.

Space group: P 63/m m c (194)

Crystal system: Hexagonal

Cell parameters: a=**3.43600 Å**, c= **25.39200 Å**

Density [g·cm$^{-3}$] = 8.65600

Hamaker Constant [eV] = **1.80**

Molecular Mass [g·mol$^{-1}$] = 338.9

$f_{Pauling}$ = **0.241**

Band gap [eV] = N/A

N/A

$E_{total}$ $^{theory\ (RPA)}$ [meV·Å$^{-2}$] = **19.44**
$E_{total}$ $^{theory\ (corrected\ rVV10)}$ [meV·Å$^{-2}$] = **19.21**

N/A

$E_{total}$ $^{empirical}$ [meV·Å$^{-2}$] = **22.9**

$E_{dispervise}$ $^{empirical}$ [meV·Å$^{-2}$] = **17.4**

**Notes**:
Crystal structure is given in ref.[380] Dielectric function of $TaSe_2$ is given in section 12.

# Crystal no. 93. $TcS_2$

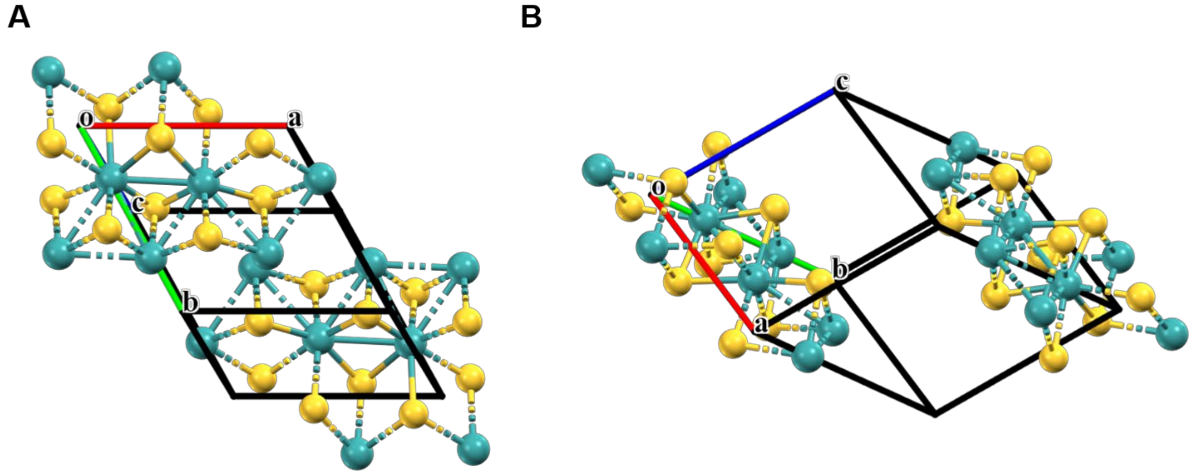


**Fig. S116. Visualization of the packed crystal structure of $TcS_2$.** A) The packed crystal is visualized along c*. B) To better illustrate the 3D structure, packed cell is visualized employing free-style orientation.

Density [g·cm$^{-3}$] = 5.05

Molecular Mass [g·mol$^{-1}$] = 162.1

Band gap [eV] = 1

$$\varepsilon_\infty = \begin{pmatrix} 16.3 & & \\ & 16.3 & \\ & & \approx 7 \end{pmatrix}$$

N/A

Hamaker Constant [eV] = **1.713**

$f_{Pauling}$ = **0.109**

$E_{total}$ $^{theory\ (RPA)}$ [meV·Å$^{-2}$] = **N/A**
$E_{total}$ $^{theory\ (rescaled\ VV10)}$ [meV·Å$^{-2}$] = **19.7**

$E_{total}$ $^{empirical}$ [meV·Å$^{-2}$] = **18.5**

$E_{dispervise}$ $^{empirical}$ [meV·Å$^{-2}$] = **16.5**

**Notes**:
Density is calculated form XRD data in ref.[381] Band gap is reported in ref.[382] Dielectric constant (in-plane) is approximated using Eq. (S1). Electronic dielectric constant (out-of-plane) is a crude estimation based on the level anisotropy observed for other transition metal dichalcogenides with similar structure.

# Crystal no. 94. Te

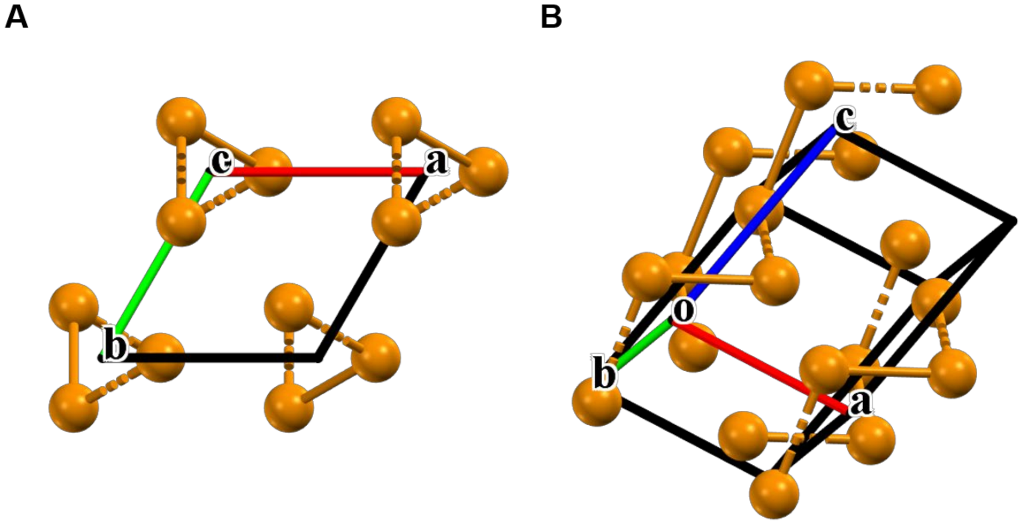


**Fig. S117. Visualization of the packed crystal structure of Te.** A) The packed crystal is visualized along c*. B) To better illustrate the 3D structure, packed cell is visualized employing free-style orientation.

Space group: P 31 2 1 (152)

Crystal system: Trigonal (Hexagonal axes)

Cell parameters: a=**4.45400 Å**, c= **5.92400 Å**

Density [g·cm$^{-3}$] = 6.24500

Hamaker Constant [eV] = **1.932**

Molecular Mass [g·mol$^{-1}$] = 127.6

$f_{Pauling}$ = **0.0**

Band gap [eV] = 0.33

$$\varepsilon_\infty = \begin{pmatrix} 23 & & \\ & 23 & \\ & & 38 \end{pmatrix}$$

$E_{total}^{theory\ (RPA)}$ [meV·Å$^{-2}$] = **N/A**

$E_{total}^{theory\ (corrected\ rVV10)}$ [meV·Å$^{-2}$] = **N/A**

$$\varepsilon(0) = \begin{pmatrix} 33.5 & & \\ & 33.5 & \\ & & 51.3 \end{pmatrix}$$

$E_{total}^{empirical}$ [meV·Å$^{-2}$] = **18.6**

$E_{dispervise}^{empirical}$ [meV·Å$^{-2}$] = **18.6**

**Notes**:
Crystal structure is given in ref.[383] Dielectric function of Te is given in section 12.

# Crystal no. 95. TiOBr

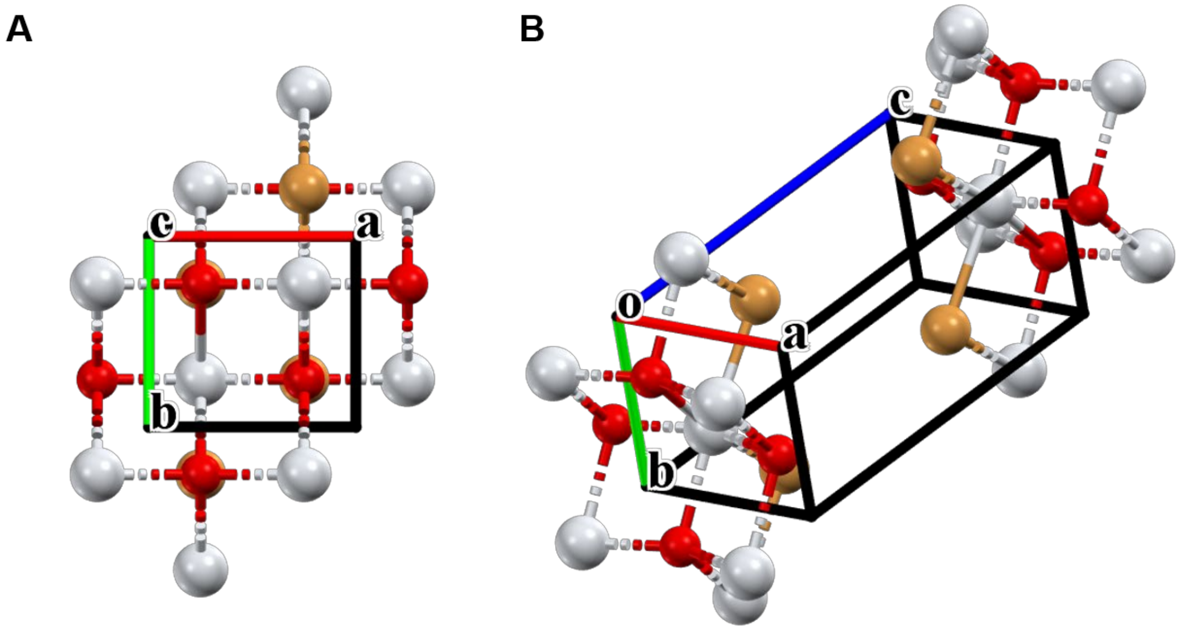


**Fig. S118. Visualization of the packed crystal structure of TiOBr.** A) The packed crystal is visualized along c*. B) To better illustrate the 3D structure, packed cell is visualized employing free-style orientation.

Space group: P m m n (59)

Crystal system: Orthorhombic

Cell parameters: a=**3.78700 Å**, b= **3.48700 Å** c= **8.52900 Å**

Density [g·cm$^{-3}$] = 4.23900

Hamaker Constant [eV] = 0.**794**

Molecular Mass [g·mol$^{-1}$] = 143.8

$f_{Pauling}$ = **0.495**

Band gap [eV] = 1.8

$$\varepsilon_\infty = \begin{pmatrix} 4.74 & & \\ & 4.54 & \\ & & N/A \end{pmatrix}$$

N/A

$E_{total}$ $^{theory (RPA)}$ [meV·Å$^{-2}$] = **N/A**

$E_{total}$ $^{theory (corrected rVV10)}$ [meV·Å$^{-2}$] = **13.27**

$E_{total}$ $^{empirical}$ [meV·Å$^{-2}$] = **15.0**

$E_{dispervise}$ $^{empirical}$ [meV·Å$^{-2}$] = **7.6**

**Notes**:

Crystal structure is given in ref.[384] Band gap is estimated from the transmission spectrum in ref.[385] Dielectric constants are reported in ref.[386] Anisotropy is ignored. Ionicity is the average ionicity of Ti-O and Ti-Br bonds.

# Crystal no. 96. TiOCl

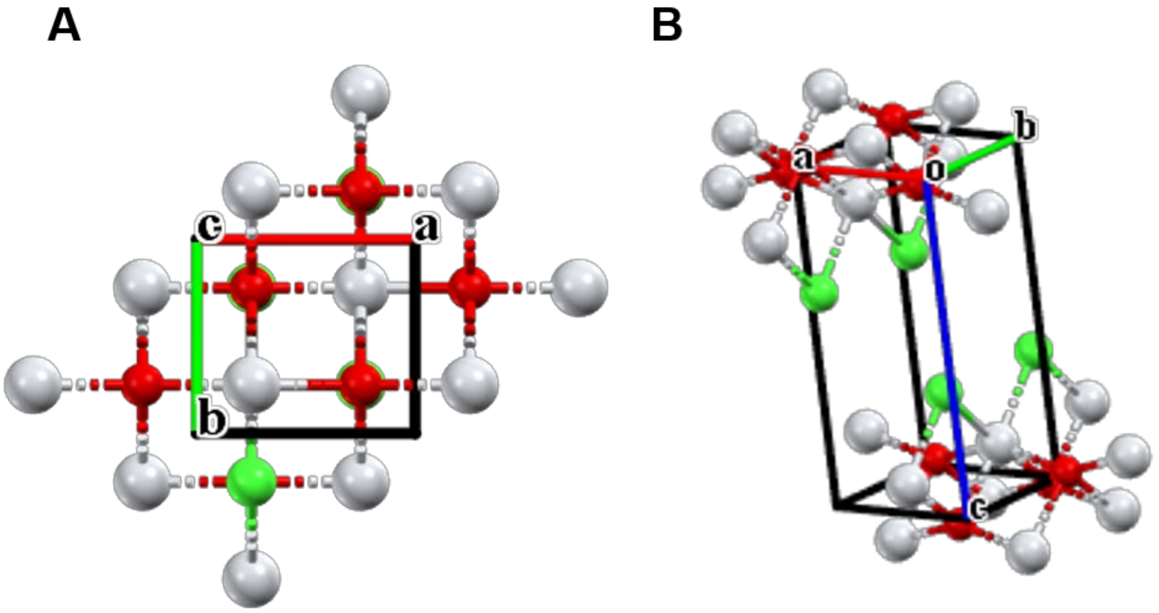


**Fig. S119. Visualization of the packed crystal structure of TiOCl.** A) The packed crystal is visualized along c*. B) To better illustrate the 3D structure, packed cell is visualized employing free-style orientation.

Space group: P m m n (59)

Crystal system: Orthorhombic

Cell parameters: a=**3.78600 Å**, b= **3.36100 Å** c= **8.04500 Å**

Density [$g \cdot cm^{-3}$] = 3.22200

Hamaker Constant [eV] = **0.68**

Molecular Mass [$g \cdot mol^{-1}$] = 99.32

$f_{Pauling}$ = **0.538**

Band gap [eV] = 1.9

$$\varepsilon_{\infty} = \begin{pmatrix} 4.1 & & \\ & 4.1 & \\ & & N/A \end{pmatrix}$$

$E_{total}^{theory\ (RPA)}$ [$meV \cdot Å^{-2}$] = **N/A**

$E_{total}^{theory\ (corrected\ rVV10)}$ [$meV \cdot Å^{-2}$] = **12.87**

$$\varepsilon(0) = \begin{pmatrix} 14.4 & & \\ & 9.9 & \\ & & N/A \end{pmatrix}$$

$E_{total}^{empirical}$ [$meV \cdot Å^{-2}$] = **14.2**

$E_{dispervise}^{empirical}$ [$meV \cdot Å^{-2}$] = **6.55**

**Notes**:
Crystal structure is given in ref.[387] Band gap and dielectric constants are reported in ref.[386] Anisotropy is ignored. Ionicity is the average ionicity of Ti-O and Ti-Cl bonds.

# Crystal no. 97. $TiS_2$

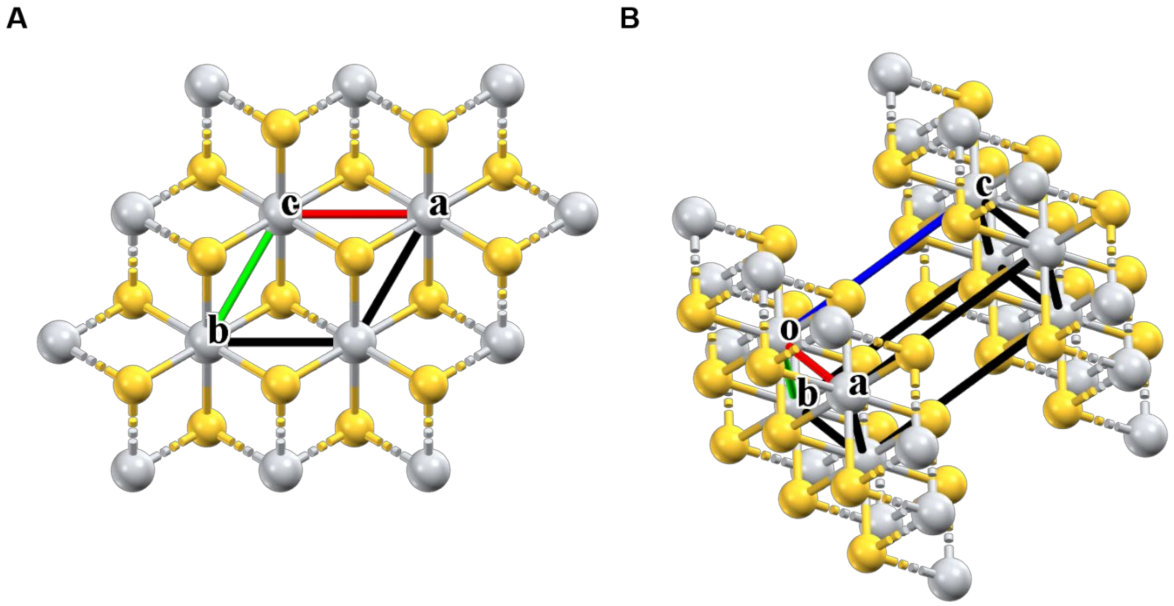


**Fig. S120. Visualization of the packed crystal structure of $TiS_2$.** A) The packed crystal is visualized along c*. B) To better illustrate the 3D structure, packed cell is visualized employing free-style orientation.

Space group: P -3 m 1 (164)

Crystal system: Trigonal (Hexagonal axes)

Cell parameters: a=**3.39700 Å**, c= **5.69100 Å**

Density [g·cm$^{-3}$] = 3.27000

Hamaker Constant [eV] = **1.57**

Molecular Mass [g·mol$^{-1}$] = 112.0

$f_{Pauling}$ = **0.237**

Band gap [eV] = N/A

N/A

$E_{total}^{theory (RPA)}$ [meV·Å$^{-2}$] = **18.88**

$E_{total}^{theory (corrected rVV10)}$ [meV·Å$^{-2}$] = **18.084**

N/A

$E_{total}^{empirical}$ [meV·Å$^{-2}$] = **19.8**

$E_{dispervise}^{empirical}$ [meV·Å$^{-2}$] = **15.1**

**Notes**:

Crystal structure is given in ref.[388] Dielectric function is estimated from the onset of the inter-band transition and the low frequency dielectric constant, as explained in our previous work.[16] The onset of the inter-band transition is assumed to be 0.5 eV.[105, 389] Low frequency dielectric constant is 19.4.[390] A Drude band is included in calculation of Hamaker constant with plasma and scattering frequencies of 1.04 and 0.183 eV, respectively.[390] This is equivalent to DC conductivity of 795 S·cm$^{-1}$ which is consistent with the measurements in ref.[391] Anisotropy is ignored.

# Crystal no. 98. $TiSe_2$

A B

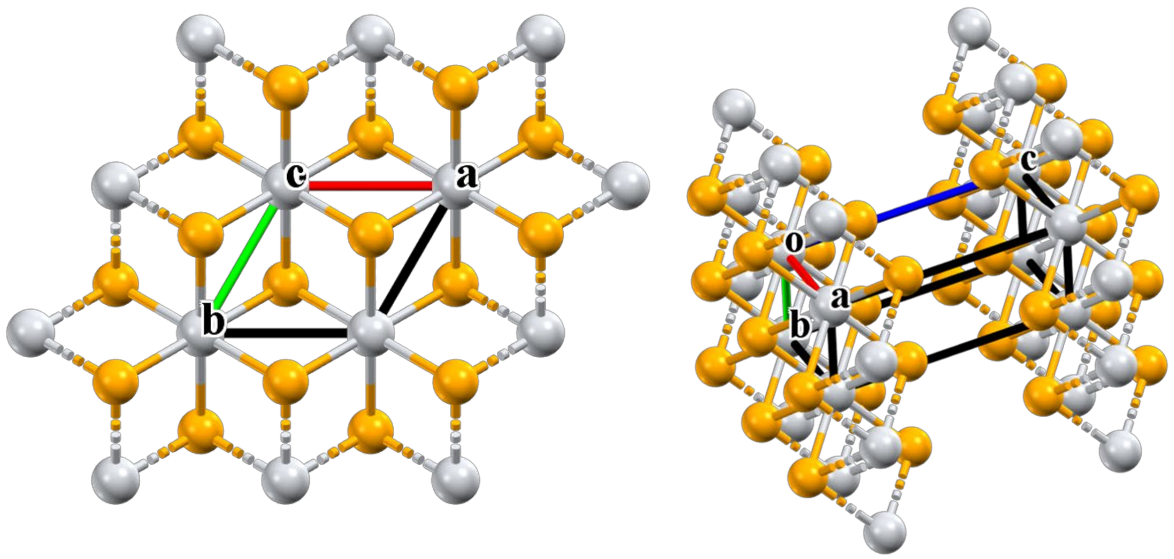


**Fig. S121. Visualization of the packed crystal structure of $TiSe_2$.** A) The packed crystal is visualized along c*. B) To better illustrate the 3D structure, packed cell is visualized employing free-style orientation.

| | |
|---|---|
| Space group: P -3 m 1 (164) | Crystal system: Trigonal (Hexagonal axes) |
| Cell parameters: a=**3.53300 Å**, c= **5.99500 Å** | |
| Density [g·cm$^{-3}$] = 5.27300 | Hamaker Constant [eV] = **1.72** |
| Molecular Mass [g·mol$^{-1}$] = 205.8 | $f_{Pauling}$ = **0.225** |
| Band gap [eV] = N/A | |
| N/A | $E_{total}$ $^{theory\ (RPA)}$ [meV·Å$^{-2}$] = **17.39**<br>$E_{total}$ $^{theory\ (corrected\ rVV10)}$ [meV·Å$^{-2}$] = **18.61** |
| N/A | $E_{total}$ $^{empirical}$ [meV·Å$^{-2}$] = **21.3**<br>$E_{dispervise}$ $^{empirical}$ [meV·Å$^{-2}$] = **16.5** |

**Notes**:
Crystal structure is given in ref.[388] Dielectric function is estimated from the onset of the inter-band transition and the low frequency dielectric constant, as explained in our previous work.[16] The onset of the inter-band transition is estimated to be 0.4 eV.[103] Low frequency dielectric constant is 29.[392] Drude band is included in calculation of Hamaker constant with plasma and scattering frequencies of 0.9 and 0.21 eV, respectively. This is equivalent to DC conductivity of 525 S·cm$^{-1}$ which is consistent with the measurements in ref.[393] Anisotropy is ignored.

# Crystal no. 99. $TiTe_2$

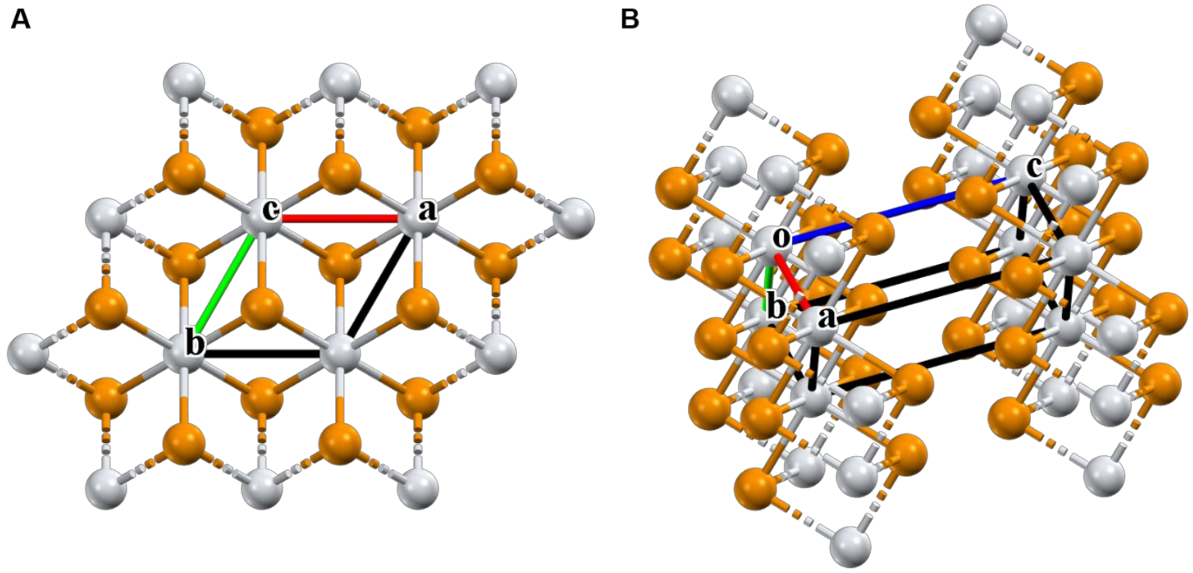


**Fig. S122. Visualization of the packed crystal structure of $TiTe_2$.** A) The packed crystal is visualized along c*. B) To better illustrate the 3D structure, packed cell is visualized employing free-style orientation.

Space group: P -3 m 1 (164) | Crystal system: Trigonal (Hexagonal axes)

Cell parameters: a=**3.77700 Å**, c= **6.49800 Å**

Density [$g \cdot cm^{-3}$] = 6.26900 | Hamaker Constant [eV] = **1.87**

Molecular Mass [$g \cdot mol^{-1}$] = 303.1 | $f_{Pauling}$ = **0.075**

Band gap [eV] = N/A

N/A | $E_{total}^{theory (RPA)}$ [$meV \cdot Å^{-2}$] = **19.76**
$E_{total}^{theory (corrected rVV10)}$ [$meV \cdot Å^{-2}$] = **19.93**

N/A | $E_{total}^{empirical}$ [$meV \cdot Å^{-2}$] = **19.5**

$E_{dispervise}^{empirical}$ [$meV \cdot Å^{-2}$] = **18.0**

**Notes**:
Crystal structure is given in ref.[394] Dielectric function of $TiTe_2$ is given in section 12.

# Crystal no. 100. $TlInSe_2$

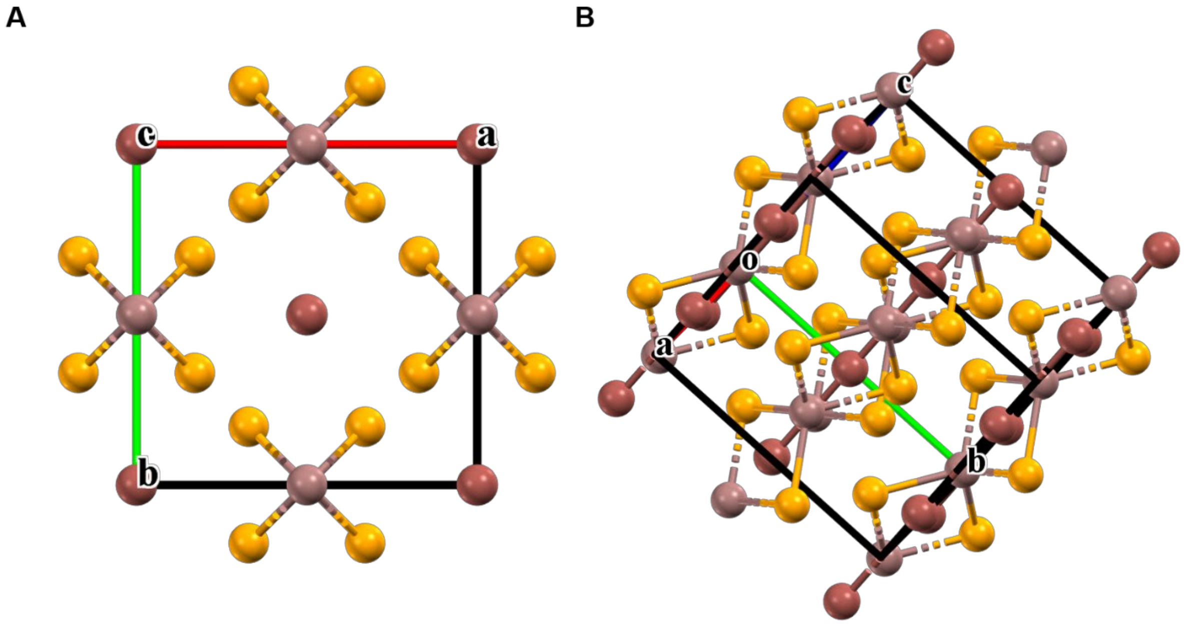


**Fig. S123. Visualization of the packed crystal structure of $TlInSe_3$.** A) The packed crystal is visualized along c*. B) To better illustrate the 3D structure, packed cell is visualized employing free-style orientation.

Space group: I 4/m c m (140)

Crystal system: Tetragonal

Cell parameters: a=**8.07500 Å**, c= **6.84700 Å**

Density [g·cm$^{-3}$] = 7.09800

Hamaker Constant [eV] = **1.34**

Molecular Mass [g·mol$^{-1}$] = 477.1

$f_{Pauling}$ = **0.138**

Band gap [eV] = 1.44

$$\varepsilon_\infty = \begin{pmatrix} 8.4 & & \\ & 8.4 & \\ & & 6 \end{pmatrix}$$

$E_{total}^{theory\ (RPA)}$ [meV·Å$^{-2}$] = **N/A**

$E_{total}^{theory\ (corrected\ rVV10)}$ [meV·Å$^{-2}$] = **N/A**

$$\varepsilon(0) = \begin{pmatrix} 11.6 & & \\ & 11.6 & \\ & & 7 \end{pmatrix}$$

$E_{total}^{empirical}$ [meV·Å$^{-2}$] = **15.0**

$E_{dispervise}^{empirical}$ [meV·Å$^{-2}$] = **12.9**

**Notes**:

Crystal structure is given in ref.[395] Note that this a chain-like structure. Band gap is reported in ref.[396] Dielectric constants along with the position of the infrared bands are reported in ref.[397] Ionicity is for the In-Se bond.

# Crystal no. 101. $V_2O_5$

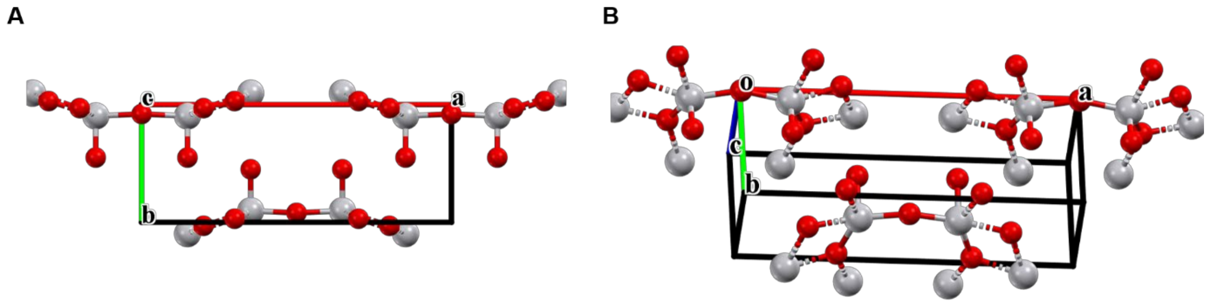


**Fig. S124. Visualization of the packed crystal structure of $V_2O_5$.** A) The packed crystal is visualized along c*. B) To better illustrate the 3D structure, packed cell is visualized employing free-style orientation.

Space group: P m n 21 (31)

Crystal system: Orthorhombic

Cell parameters: a=**11.48000 Å**, b= **4.36000 Å** c= **3.55000 Å**

Density [g·cm$^{-3}$] = 3.39900

Hamaker Constant [eV] = **0.883**

Molecular Mass [g·mol$^{-1}$] = 149.9

$f_{Pauling}$ = **0.559**

Band gap [eV] = 2.4

$$\varepsilon_\infty = \begin{pmatrix} 4.3 & & \\ & 4.5 & \\ & & 3.88 \end{pmatrix}$$

$E_{total}$ $^{theory\ (RPA)}$ [meV·Å$^{-2}$] = **N/A**

$E_{total}$ $^{theory\ (corrected\ rVV10)}$ [meV·Å$^{-2}$] = **19.01**

$$\varepsilon(0) = \begin{pmatrix} 20.1 & & \\ & 37.2 & \\ & & 6.48 \end{pmatrix}$$

$E_{total}$ $^{empirical}$ [meV·Å$^{-2}$] = **19.3**

$E_{dispervise}$ $^{empirical}$ [meV·Å$^{-2}$] = **8.5**

**Notes**:

Crystal structure is given in ref.[398] Band gap is reported in ref.[399] Electronic dielectric constants are reported in ref.[400] Note that these values are not consistent with the proposed values in ref.[401] but they are in better consistency with the infrared reflectivity measurements in ref.[402] Dielectric constants along with the position of infrared bands are reported in ref.[402]

# Crystal no. 102. $VSe_2$

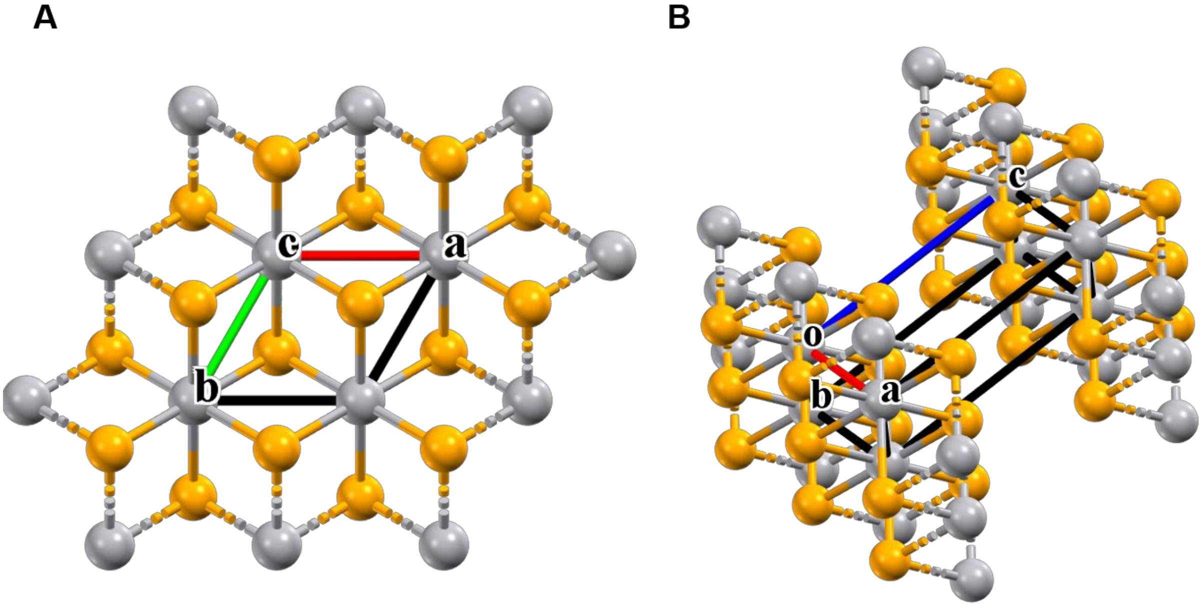


**Fig. S125. Visualization of the packed crystal structure of $VSe_2$.** A) The packed crystal is visualized along c*. B) To better illustrate the 3D structure, packed cell is visualized employing free-style orientation.

Space group: P -3 m 1 (164)

Crystal system: Trigonal (Hexagonal axes)

Cell parameters: a=**3.55500 Å**, c= **6.13400 Å**

Density [g·cm$^{-3}$] = 5.80000

Hamaker Constant [eV] = **1.881**

Molecular Mass [g·mol$^{-1}$] = 208.9

$f_{Pauling}$ = **0.191**

Band gap [eV] = N/A

N/A

$E_{total}^{theory\ (RPA)}$ [meV·Å$^{-2}$] = **22.26**
$E_{total}^{theory\ (corrected\ rVV10)}$ [meV·Å$^{-2}$] = **19.93**

N/A

$E_{total}^{empirical}$ [meV·Å$^{-2}$] = **22.4**

$E_{dispervise}^{empirical}$ [meV·Å$^{-2}$] = **18.1**

**Notes**:
Crystal strucure is given in ref.[403] Dielectric function of $VSe_2$ is given in section 12.

# Crystal no. 103. $WS_2$

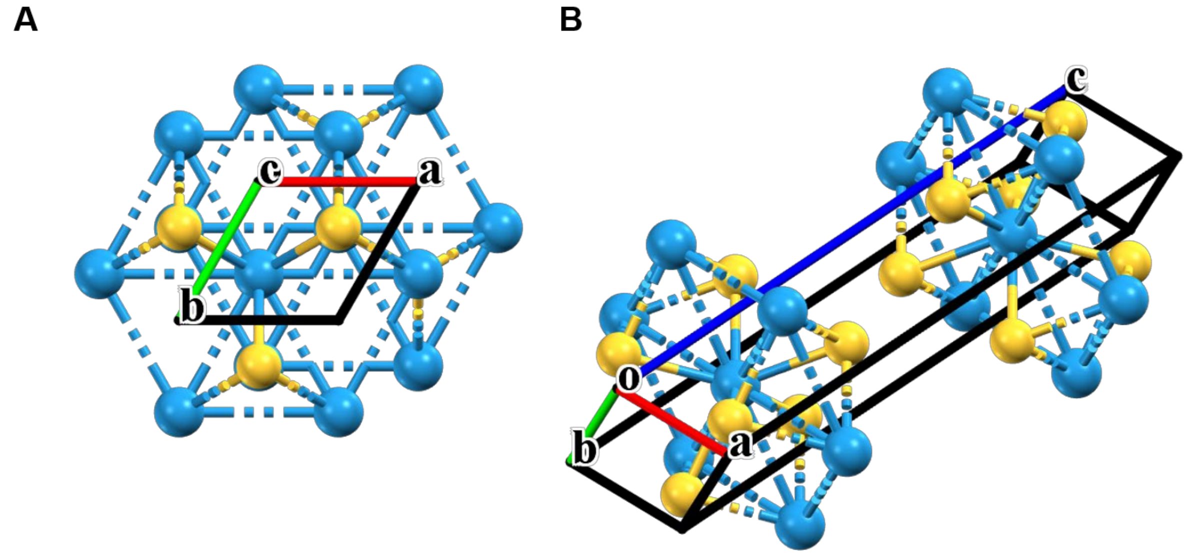


**Fig. S126. Visualization of the packed crystal structure of $WS_2$.** A) The packed crystal is visualized along c*. B) To better illustrate the 3D structure, packed cell is visualized employing free-style orientation.

Space group: P 63/m m c (194)

Crystal system: Hexagonal

Cell parameters: a=**3.18000 Å**, c= **12.50000 Å**

Density [g·cm$^{-3}$] = 7.52300

Hamaker Constant [eV] = **1.932**

Molecular Mass [g·mol$^{-1}$] = 248.0

$f_{Pauling}$ = **0.012**

Band gap [eV] = 1.8

$$\varepsilon_\infty = \begin{pmatrix} 12.5 & & \\ & 12.5 & \\ & & 6.2 \end{pmatrix}$$

$E_{total}$ $^{theory\ (RPA)}$ [meV·Å$^{-2}$] = **20.24**

$E_{total}$ $^{theory\ (corrected\ rVV10)}$ [meV·Å$^{-2}$] = **19.73**

$$\varepsilon(0) = \begin{pmatrix} 12.8 & & \\ & 12.8 & \\ & & \approx 6.2 \end{pmatrix}$$

$E_{total}$ $^{empirical}$ [meV·Å$^{-2}$] = **18.8**

$E_{dispervise}$ $^{empirical}$ [meV·Å$^{-2}$] = **18.6**

**Notes**:

Crystal structure is given in ref.[404] Dielectric function of $WS_2$ is given in our previous work.[16]

# Crystal no. 104. $WSe_2$

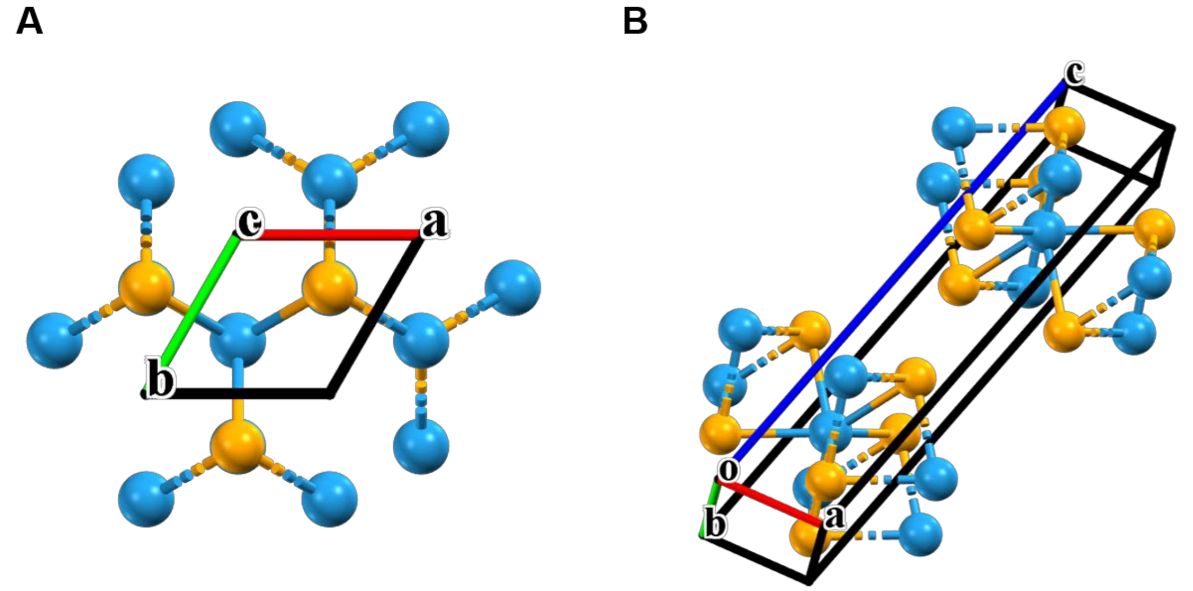


**Fig. S127. Visualization of the packed crystal structure of $WSe_2$.** A) The packed crystal is visualized along c*. B) To better illustrate the 3D structure, packed cell is visualized employing free-style orientation.

Space group: P 63/m m c (194)

Crystal system: Hexagonal

Cell parameters: a=**3.28200 Å**, c= **12.96000 Å**

Density [g·cm$^{-3}$] = 9.38900

Hamaker Constant [eV] = **1.90**

Molecular Mass [g·mol$^{-1}$] = 341.8

$f_{Pauling}$ = **0.009**

Band gap [eV] = 1.35

$$\varepsilon_{\infty} = \begin{pmatrix} 12.9 & & \\ & 12.9 & \\ & & 7.8 \end{pmatrix}$$

$$\varepsilon(0) = \begin{pmatrix} 13.3 & & \\ & 13.3 & \\ & & \approx 7.8 \end{pmatrix}$$

$E_{total}^{theory\ (RPA)}$ [meV·Å$^{-2}$] = **19.98**

$E_{total}^{theory\ (corrected\ rVV10)}$ [meV·Å$^{-2}$] = **19.8**

$E_{total}^{empirical}$ [meV·Å$^{-2}$] = **18.5**

$E_{dispervise}^{empirical}$ [meV·Å$^{-2}$] = **18.3**

**Notes**:

Crystal structure is given in ref.[405] Dielectric function of $WS_2$ is given in our previous work.[16]

# Crystal no. 105. ZrNCl

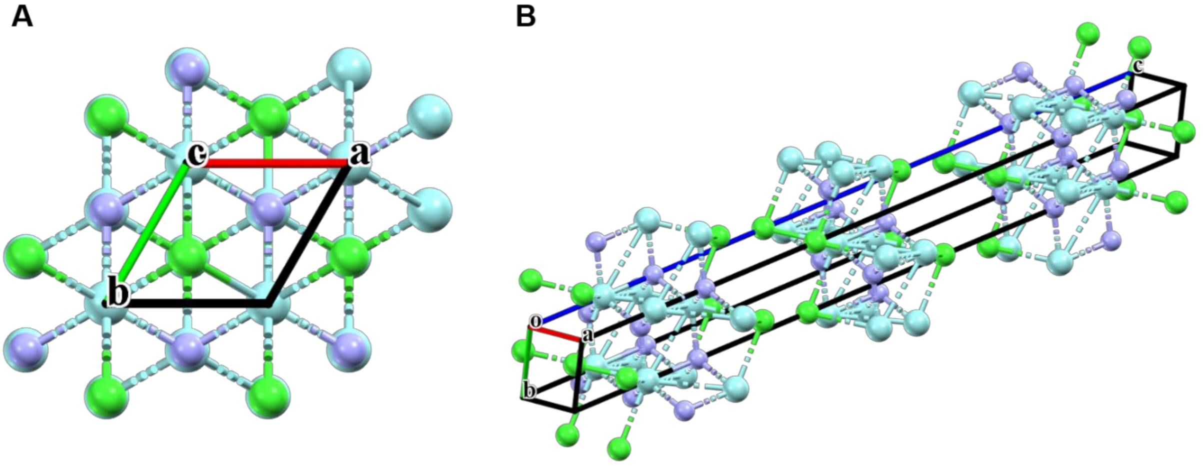


**Fig. S128. Visualization of the packed crystal structure of ZrNCl.** A) The packed crystal is visualized along c*. B) To better illustrate the 3D structure, packed cell is visualized employing free-style orientation.

Space group: R -3 m (166)

Crystal system: Trigonal (Hexagonal axes)

Cell parameters: a=**3.60388 Å**, c= **27.67189 Å**

Density [g·cm$^{-3}$] = 4.50300

Hamaker Constant [eV] = **1.0364**

Molecular Mass [g·mol$^{-1}$] = 140.7

$f_{Pauling}$ = **0.543**

Band gap [eV] = 3

$$\varepsilon_\infty = \begin{pmatrix} 4.4 & & \\ & 4.4 & \\ & & N/A \end{pmatrix}$$

N/A

$E_{total}$ $^{theory\ (RPA)}$ [meV·Å$^{-2}$] = **N/A**

$E_{total}$ $^{theory\ (corrected\ VV10)}$ [meV·Å$^{-2}$] = **16.62**

$E_{total}$ $^{empirical}$ [meV·Å$^{-2}$] = **21.8**

$E_{dispervise}$ $^{empirical}$ [meV·Å$^{-2}$] = **10.0**

**Notes**:

Crystal structure is given in ref.[406] Band gap and electronic dielectric constant are estimated from the reflectance spectrum in ref.[407] Anisotropy is ignored. Ionicity is the average ionicity of Zr-N and Zr-Cl bonds.

# Crystal no. 106. $ZrS_2$

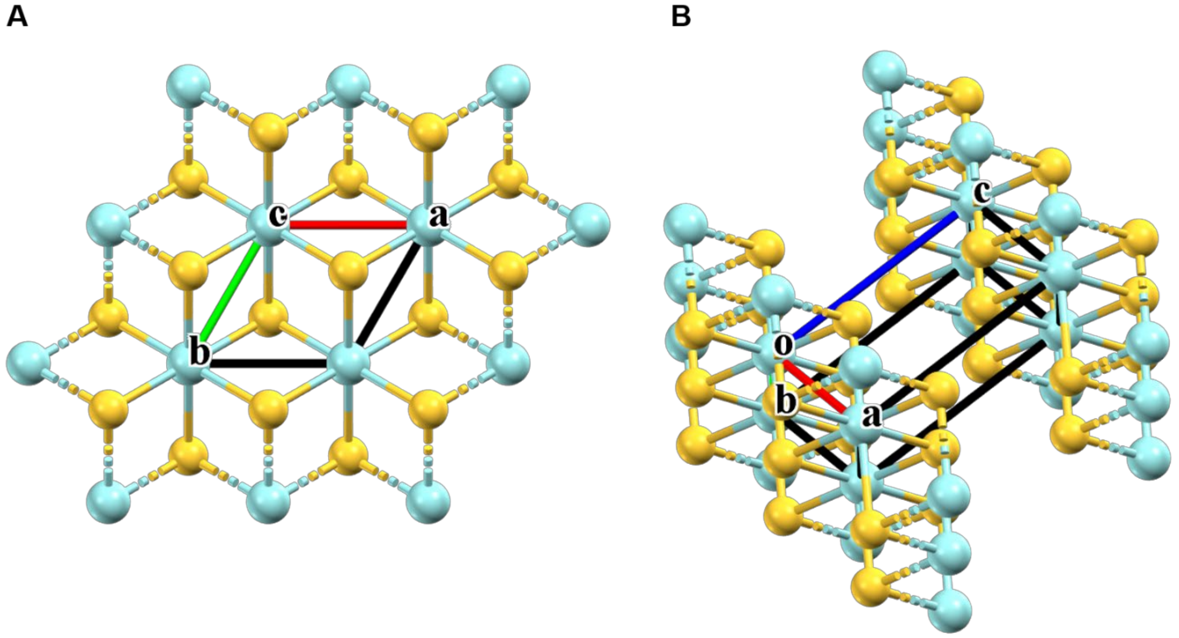


**Fig. S129. Visualization of the packed crystal structure of $ZrS_2$.** A) The packed crystal is visualized along c*. B) To better illustrate the 3D structure, packed cell is visualized employing free-style orientation.

Space group: P -3 m 1 (164)

Crystal system: Trigonal (Hexagonal axes)

Cell parameters: a=**3.63000 Å**, c= **5.85000 Å**

Density [g·cm$^{-3}$] = 3.86400

Hamaker Constant [eV] = **1.34**

Molecular Mass [g·mol$^{-1}$] = 155.35

$f_{Pauling}$ = **0.323**

Band gap [eV] = 1.7

$$\varepsilon_\infty = \begin{pmatrix} 8 & & \\ & 8 & \\ & & 6.1 \end{pmatrix}$$

$E_{total}^{theory\ (RPA)}$ [meV·Å$^{-2}$] = **16.98**

$E_{total}^{theory\ (corrected\ rVV10)}$ [meV·Å$^{-2}$] = **15.91**

$$\varepsilon(0) = \begin{pmatrix} 34.6 & & \\ & 34.6 & \\ & & 10.2 \end{pmatrix}$$

$E_{total}^{empirical}$ [meV·Å$^{-2}$] = **19.1**

$E_{dispervise}^{empirical}$ [meV·Å$^{-2}$] = **12.9**

**Notes**:

Crystal structure is given in ref.[404] Band gap and in-plane electronic dielectric constant are reported in ref.[408] Other elements of dielectric tensors are reported in ref.[292]

# Crystal no. 107. $ZrSe_2$

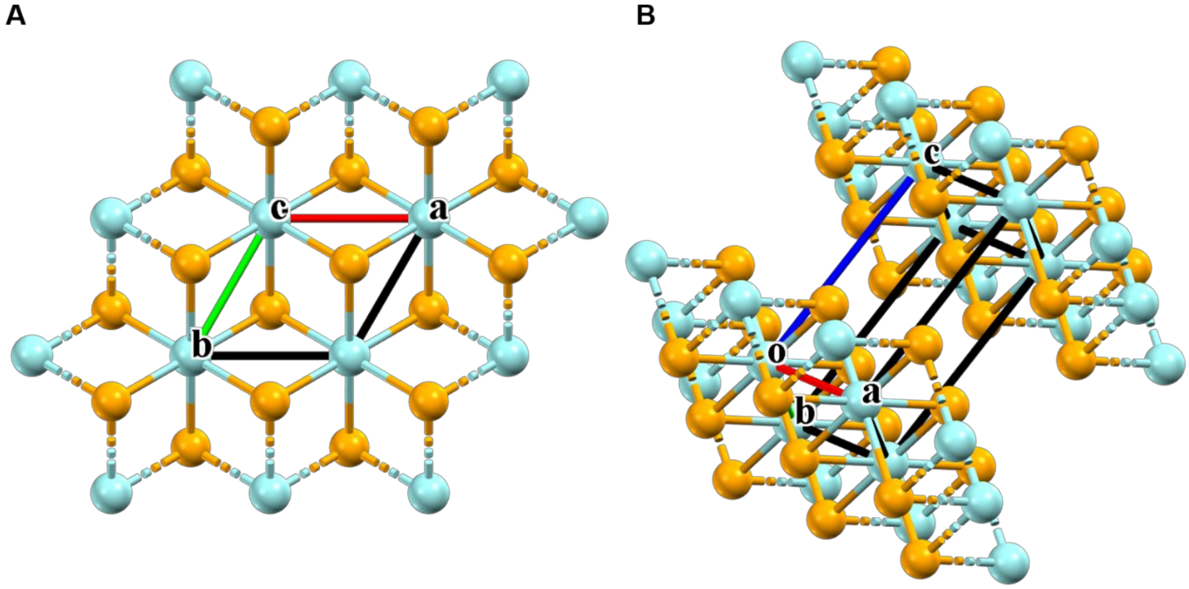


**Fig. S130. Visualization of the packed crystal structure of $ZrSe_2$.** A) The packed crystal is visualized along c*. B) To better illustrate the 3D structure, packed cell is visualized employing free-style orientation.

Space group: P -3 m 1 (164)

Crystal system: Trigonal (Hexagonal axes)

Cell parameters: a=**3.77100 Å**, c= **6.12900 Å**

Density [g·cm$^{-3}$] = 5.4810

Hamaker Constant [eV] = **1.41**

Molecular Mass [g·mol$^{-1}$] = 249.14

$f_{Pauling}$ = **0.311**

Band gap [eV] = 1.2

$$\varepsilon_\infty = \begin{pmatrix} 11 & & \\ & 11 & \\ & & 5.5 \end{pmatrix}$$

N/A

$E_{total}$ $^{theory\ (RPA)}$ [meV·Å$^{-2}$] = **18.53**

$E_{total}$ $^{theory\ (corrected\ rVV10)}$ [meV·Å$^{-2}$] = **16.5**

$E_{total}$ $^{empirical}$ [meV·Å$^{-2}$] = **19.7**

$E_{dispervise}$ $^{empirical}$ [meV·Å$^{-2}$] = **13.6**

**Notes**:

Crystal structure is given in ref.[338] Band gap is reported in ref.[409] Electronic dielectric constants are estimated by analysing the reflectance spectra reported in ref.[105] using RefFit software.[410] Note that the measurements are done at 77 K. There are some inconsistencies in reported values for the electronic dielectric constants of $ZrSe_2$. (See for instance refs.[411]). The cited electronic constant (in-plane) in our analysis is consistent with the measurements of Lee *et al*.[408] and Bell *et al*.[412]. It is also consistent with the predicted value based on the empirical Eqs. (5) and (S18), (*i.e.* 12.5).

References

[1] J. N. Israelachvili, *Intermolecular and surface forces*, Academic press, **2011**.
[2] M. J. Vold, *Journal of Colloid Science* **1961**, 16, 1.
[3] F. M. Fowkes, in *Contact Angle, Wettability, and Adhesion*, Vol. 43, AMERICAN CHEMICAL SOCIETY, 1964, 6.
[4] J. N. Israelachvili, *Journal of the Chemical Society, Faraday Transactions 2: Molecular and Chemical Physics* **1973**, 69, 1729; J. Mahanty, B. W. Ninham, *Dispersion forces*, Vol. 1, Academic Press, **1976**.
[5] P.-G. De Gennes, *Reviews of modern physics* **1985**, 57, 827.
[6] C. J. Van Oss, M. K. Chaudhury, R. J. Good, *Chemical reviews* **1988**, 88, 927.
[7] V. A. Parsegian, *Van der Waals forces: a handbook for biologists, chemists, engineers, and physicists*, Cambridge University Press, **2005**.
[8] J. Ángyán, J. Dobson, G. Jansen, T. Gould, *London dispersion forces in molecules, solids and nano-structures: an introduction to physical models and computational methods*, Royal Society of Chemistry, **2020**.
[9] S. Grimme, J. Antony, S. Ehrlich, H. Krieg, *The Journal of chemical physics* **2010**, 132, 154104.
[10] H. C. Hamaker, *physica* **1937**, 4, 1058.
[11] P. Slavíček, R. Kalus, P. Paška, I. Odvárková, P. Hobza, A. Malijevský, *The Journal of chemical physics* **2003**, 119, 2102.
[12] N. Alves de Lima, *The Journal of Chemical Physics* **2010**, 132, 014110.
[13] A. Tkatchenko, M. Scheffler, *Physical review letters* **2009**, 102, 073005.
[14] I. E. e. Dzyaloshinskii, E. M. Lifshitz, L. P. Pitaevskii, *Advances in Physics* **1961**, 10, 165.
[15] T. Gould, E. Gray, J. F. Dobson, *Physical Review B* **2009**, 79, 113402.
[16] M. Moazzami Gudarzi, S. H. Aboutalebi, *Science Advances* **2021**, 7, eabg2272.
[17] E. Zaremba, W. Kohn, *Physical Review B* **1976**, 13, 2270.
[18] V. G. Ruiz, W. Liu, E. Zojer, M. Scheffler, A. Tkatchenko, *Physical review letters* **2012**, 108, 146103.
[19] T. Gould, S. Lebègue, J. F. Dobson, *Journal of Physics: Condensed Matter* **2013**, 25, 445010; J. F. Dobson, T. Gould, S. Lebègue, *Physical Review B* **2016**, 93, 165436.
[20] S. Lebègue, J. Harl, T. Gould, J. Ángyán, G. Kresse, J. Dobson, *Physical review letters* **2010**, 105, 196401.
[21] T. Björkman, A. Gulans, A. V. Krasheninnikov, R. M. Nieminen, *Physical Review Letters* **2012**, 108, 235502.
[22] B. Cordero, V. Gómez, A. E. Platero-Prats, M. Revés, J. Echeverría, E. Cremades, F. Barragán, S. Alvarez, *Dalton Transactions* **2008**, 2832.
[23] S. Alvarez, *Dalton Transactions* **2013**, 42, 8617.
[24] S. S. Batsanov, *Inorg. Mater.* **2001**, 37, 871.
[25] L. Pauling, C. University, C. U. Press, *The Nature of the Chemical Bond and the Structure of Molecules and Crystals: An Introduction to Modern Structural Chemistry*, Cornell University Press, **1960**.
[26] A. C. Thompson, D. T. Attwood, E. M. Gullikson, M. R. Howells, J. B. Kortright, A. L. Robinson, J. H. Underwood, *X-Ray Data Booklet*, Center for X-ray Optics and Advanced Light Source, Berkeley, California **2009**.
[27] C. F. Matta, L. Massa, A. V. Gubskaya, E. Knoll, *Journal of Chemical Education* **2011**, 88, 67.
[28]
[29] R. J. Maurer, C. Freysoldt, A. M. Reilly, J. G. Brandenburg, O. T. Hofmann, T. Björkman, S. Lebègue, A. Tkatchenko, *Annual Review of Materials Research* **2019**, 49, 1.
[30] N. Mounet, M. Gibertini, P. Schwaller, D. Campi, A. Merkys, A. Marrazzo, T. Sohier, I. E. Castelli, A. Cepellotti, G. Pizzi, N. Marzari, *Nature Nanotechnology* **2018**, 13, 246.

[31] N. Mounet, M. Gibertini, P. Schwaller, D. Campi, A. Merkys, A. Marrazzo, T. Sohier, I. E. Castelli, A. Cepellotti, G. Pizzi, N. Marzari, (Ed: M. C. Archive), 2020.
[32] L. Pauling, *The Nature of the Chemical Bond*, Vol. 260, Cornell university press Ithaca, NY, **1960**.
[33] J. Phillips, *Reviews of Modern Physics* **1970**, 42, 317.
[34] L. Pauling, J. Phillips, *Phys. Today* **1971**, 24, 9.
[35] B. Szigeti, *Transactions of the Faraday Society* **1949**, 45, 155.
[36] D. Tabor, R. H. S. Winterton, *Proceedings of the Royal Society of London. A. Mathematical and Physical Sciences* **1969**, 312, 435.
[37] H. Krupp, *Advances in Colloid and Interface Science* **1967**, 1, 111.
[38] E. M. Lifshitz, M. Hamermesh, in *Perspectives in Theoretical Physics*, (Ed: L. P. Pitaevski), Pergamon, Amsterdam 1992.
[39] R. J. Gillespie, P. L. A. Popelier, *Chemical Bonding and Molecular Geometry*, Oxford Universiity Press, **2001**.
[40] J. A. Van Vechten, *Physical Review* **1969**, 182, 891.
[41] S. Wemple, M. DiDomenico Jr, *Physical Review B* **1971**, 3, 1338.
[42] Y. Morioka, I. Nakagawa, *Spectrochimica Acta Part A: Molecular Spectroscopy* **1978**, 34, 5.
[43] S.-i. Kondo, H. Matsumoto, *J. Phys. Soc. Jpn.* **1982**, 51, 1441.
[44] Y. O. Dovgii, I. V. Kityk, Y. M. Aleksandrov, V. N. Kolobanov, V. N. Makhov, V. V. Mikhailin, *J. Appl. Spectrosc.* **1985**, 43, 1168.
[45] I. Pollini, J. Thomas, R. Coehoorn, C. Haas, *Physical Review B* **1986**, 33, 5747.
[46] (Ed: N. I. o. S. a. Technology), Gaithersburg MD 2000.
[47] E. Gullikson, Vol. 2021, Lawrence Berkeley National Laboratory.
[48] H. Matsumoto, H. Nakagawa, S.-i. Kondo, **1983**.
[49] S.-i. Kondo, H. Matsumoto, *J. Phys. Soc. Jpn.* **1981**, 50, 3047.
[50] A. Jain, S. P. Ong, G. Hautier, W. Chen, W. D. Richards, S. Dacek, S. Cholia, D. Gunter, D. Skinner, G. Ceder, *APL materials* **2013**, 1, 011002.
[51] I. Pollini, G. Spinolo, *physica status solidi (b)* **1970**, 41, 691.
[52] B. Carricabruru, J. Ferre, R. Mamy, I. Pollini, J. Thomas, *Journal of Physics C: Solid State Physics* **1986**, 19, 4985.
[53] S. Kazim, M. Alì, S. Palleschi, G. D'Olimpio, D. Mastrippolito, A. Politano, R. Gunnella, A. Di Cicco, M. Renzelli, G. Moccia, O. A. Cacioppo, R. Alfonsetti, J. Strychalska-Nowak, T. Klimczuk, R. J Cava, L. Ottaviano, *Nanotechnology* **2020**, 31, 395706.
[54] A. Jain, S. P. Ong, G. Hautier, W. Chen, W. D. Richards, S. Dacek, S. Cholia, D. Gunter, D. Skinner, G. Ceder, K. A. Persson, *APL Materials* **2013**, 1, 011002.
[55] A. Borghesi, G. Guizzetti, F. Marabelli, L. Nosenzo, E. Reguzzoni, *Solid State Communications* **1984**, 52, 463.
[56] C.-W. Chen, T.-T. Tang, S.-H. Lin, J. Y. Huang, C.-S. Chang, P.-K. Chung, S.-T. Yen, C.-L. Pan, *JOSA B* **2009**, 26, A58.
[57] P. Soukiassian, J. Cazaux, J. Perrin, *physica status solidi (b)* **1974**, 66, 151.
[58] K. Kato, F. Tanno, N. Umemura, *Appl. Opt.* **2013**, 52, 2325.
[59] D. F. Edwards, in *Handbook of Optical Constants of Solids*, (Ed: E. D. Palik), Academic Press, Burlington 1997.
[60] L. Lajaunie, F. Boucher, R. Dessapt, P. Moreau, *Physical Review B* **2013**, 88, 115141.
[61] M. Itoh, K. Hayakawa, S. Oishi, *Journal of Physics: Condensed Matter* **2001**, 13, 6853.
[62] S. Deb, *Proceedings of the Royal Society of London. Series A. Mathematical and Physical Sciences* **1968**, 304, 211.
[63] G. Álvarez-Pérez, T. G. Folland, I. Errea, J. Taboada-Gutiérrez, J. Duan, J. Martín-Sánchez, A. I. Tresguerres-Mata, J. R. Matson, A. Bylinkin, M. He, *Advanced Materials* **2020**, 32, 1908176.
[64] S. Parkin, A. Beal, *Philosophical Magazine B* **1980**, 42, 627.
[65] M. Naito, S. Tanaka, *Journal of the Physical Society of Japan* **1982**, 51, 219.
[66] R. Manzke, G. Crecelius, J. Fink, R. Schöllhorn, *Solid State Communications* **1981**, 40, 103.
[67] P. J. S. Foot, B. A. Nevett, *physica status solidi (a)* **1986**, 93, 283.
[68] M. Piacentini, F. S. Khumalo, C. G. Olson, J. W. Anderegg, D. W. Lynch, *Chemical Physics* **1982**, 65, 289.

[69] S. Y. Kim, T. Y. Kim, L. J. Sandilands, S. Sinn, M.-C. Lee, J. Son, S. Lee, K.-Y. Choi, W. Kim, B.-G. Park, C. Jeon, H.-D. Kim, C.-H. Park, J.-G. Park, S. J. Moon, T. W. Noh, *Phys. Rev. Lett.* **2018**, 120, 136402.
[70] C.-T. Kuo, M. Neumann, K. Balamurugan, H. J. Park, S. Kang, H. W. Shiu, J. H. Kang, B. H. Hong, M. Han, T. W. Noh, J.-G. Park, *Scientific Reports* **2016**, 6, 20904.
[71] C. Lane, J.-X. Zhu, *Physical Review B* **2020**, 102, 075124.
[72] C. A. Belvin, E. Baldini, I. O. Ozel, D. Mao, H. C. Po, C. J. Allington, S. Son, B. H. Kim, J. Kim, I. Hwang, J. H. Kim, J.-G. Park, T. Senthil, N. Gedik, *Nature Communications* **2021**, 12, 4837.
[73] C. Xu, B. Li, W. Jiao, W. Zhou, B. Qian, R. Sankar, N. D. Zhigadlo, Y. Qi, D. Qian, F.-C. Chou, X. Xu, *Chemistry of Materials* **2018**, 30, 4823.
[74] R. Mamy, B. Thieblemont, L. Martin, F. Pradal, *Il Nuovo Cimento B (1971-1996)* **1977**, 38, 196.
[75] A. F. Fang, G. Xu, T. Dong, P. Zheng, N. L. Wang, *Scientific Reports* **2013**, 3, 1153.
[76] C. Ghiţă, L. Ghiţă, I. Baltog, M. Constantinescu, *physica status solidi (b)* **1980**, 102, 111.
[77] R. A. Abreu, *Physics Letters A* **1984**, 100, 375.
[78] T. Hayashi, K. Toyoda, M. Itoh, *Journal of the Physical Society of Japan* **1988**, 57, 1861.
[79] R. Frisenda, J. O. Island, J. L. Lado, E. Giovanelli, P. Gant, P. Nagler, S. Bange, J. M. Lupton, C. Schüller, A. J. Molina-Mendoza, L. Aballe, M. Foerster, T. Korn, M. Angel Niño, D. P. de Lara, E. M. Pérez, J. Fernandéz-Rossier, A. Castellanos-Gomez, *Nanotechnology* **2017**, 28, 455703.
[80] A. B. Buckman, N. H. Hong, D. Wilson, *J. Opt. Soc. Am.* **1975**, 65, 914.
[81] C. Haas, *Physica B+ C* **1981**, 105, 305.
[82] N. Dean, J. C. Petersen, D. Fausti, R. a. I. Tobey, S. Kaiser, L. Gasparov, H. Berger, A. Cavalleri, *Physical review letters* **2011**, 106, 016401.
[83] G. Lucovsky, W. Y. Liang, R. M. White, K. R. Pisharody, *Solid State Communications* **1976**, 19, 303.
[84] A. Beal, H. Hughes, W. Liang, *Journal of physics C: solid state physics* **1975**, 8, 4236.
[85] B. Ruzicka, L. Degiorgi, H. Berger, R. Gaál, L. Forró, *Physical review letters* **2001**, 86, 4136.
[86] A. König, R. Schuster, M. Knupfer, B. Büchner, H. Berger, *Physical Review B* **2013**, 87, 195119.
[87] M. Selders, P. Gspan, P. Grosse, *physica status solidi (b)* **1971**, 47, 519.
[88] P. Grosse, W. Richter, *physica status solidi (b)* **1970**, 41, 239.
[89] E. D. Palik, in *Handbook of Optical Constants of Solids*, (Ed: E. D. Palik), Academic Press, Burlington 1997.
[90] S. Tutihasi, G. G. Roberts, R. C. Keezer, R. E. Drews, *Physical Review* **1969**, 177, 1143.
[91] P. Bammes, R. Klucker, E. E. Koch, T. Tuomi, *physica status solidi (b)* **1972**, 49, 561.
[92] B. Sonntag, T. Tuomi, G. Zimmerer, *physica status solidi (b)* **1973**, 58, 101.
[93] R. S. Caldwell, H. Y. Fan, *Physical Review* **1959**, 114, 664.
[94] G. H. Sherman, *MEASUREMENT OF THE NONLINEAR SUSCEPTIBILITIES OF TELLURIUM, SELENIUM, CADMIUM-TELLURIDE, AND INDIUM-ANTIMONIDE AT 28.0 MICRONS*, University of Illinois at Urbana-Champaign, **1972**.
[95] M. N. Polyanskiy.
[96] S. Schöche, T. Hofmann, R. Korlacki, T. Tiwald, M. Schubert, *Journal of Applied Physics* **2013**, 113, 164102.
[97] N. Sbai, J. Perriere, B. Gallas, E. Millon, W. Seiler, M.-C. Bernard, *Journal of Applied Physics* **2008**, 104, 033529.
[98] T. Tiwald, M. Schubert, *Measurement of rutile TiO2 dielectric tensor from 0.148 to 33 um using generalized ellipsometry*, Vol. 4103, SPIE, **2000**.
[99] R. H. French, R. M. Cannon, L. K. DeNoyer, Y. M. Chiang, *Solid State Ionics* **1995**, 75, 13.
[100] J. R. DeVore, *J. Opt. Soc. Am.* **1951**, 41, 416.
[101] P. B. Allen, N. Chetty, *Physical Review B* **1994**, 50, 14855.
[102] M. Zhang, X. Wang, A. Rahman, Q. Zeng, D. Huang, R. Dai, Z. Wang, Z. Zhang, *Applied Physics Letters* **2018**, 112, 041907.
[103] D. L. Greenaway, R. Nitsche, *Journal of Physics and Chemistry of Solids* **1965**, 26, 1445.
[104] X. Feng, J. Henke, C. Morice, C. J. Sayers, E. Da Como, J. van Wezel, E. van Heumen, *arXiv preprint arXiv:2101.03048* **2021**.

[105] S. C. Bayliss, W. Y. Liang, *Journal of Physics C: Solid State Physics* **1985**, 18, 3327.
[106] A. Gustinetti, G. Campagnoli, *Journal of Physics C: Solid State Physics* **1981**, 14, L609.
[107] A. Kis, K. Jensen, S. Aloni, W. Mickelson, A. Zettl, *Physical Review Letters* **2006**, 97, 025501.
[108] R. Zhang, Z. Ning, Y. Zhang, Q. Zheng, Q. Chen, H. Xie, Q. Zhang, W. Qian, F. Wei, *Nature Nanotechnology* **2013**, 8, 912.
[109] L. X. Benedict, N. G. Chopra, M. L. Cohen, A. Zettl, S. G. Louie, V. H. Crespi, *Chemical Physics Letters* **1998**, 286, 490.
[110] L. A. Girifalco, R. A. Lad, *The Journal of Chemical Physics* **1956**, 25, 693.
[111] Z. Li, Y. Wang, A. Kozbial, G. Shenoy, F. Zhou, R. McGinley, P. Ireland, B. Morganstein, A. Kunkel, S. P. Surwade, L. Li, H. Liu, *Nature Materials* **2013**, 12, 925.
[112] J. Abrahamson, *Carbon* **1973**, 11, 337.
[113] N. Mounet, N. Marzari, *Physical Review B* **2005**, 71, 205214.
[114] R. Zacharia, H. Ulbricht, T. Hertel, *Physical Review B* **2004**, 69, 155406.
[115] H. Ulbricht, G. Moos, T. Hertel, *Physical review letters* **2003**, 90, 095501.
[116] Z. Liu, J. Z. Liu, Y. Cheng, Z. Li, L. Wang, Q. Zheng, *Physical Review B* **2012**, 85, 205418.
[117] C. Qu, W. Cao, B. Liu, A. Wang, F. Xie, M. Ma, W. Shan, M. Urbakh, Q. Zheng, *The Journal of Physical Chemistry C* **2019**, 123, 11671.
[118] E. Koren, E. Lörtscher, C. Rawlings, A. W. Knoll, U. Duerig, *Science* **2015**, 348, 679.
[119] W. Wang, S. Dai, X. Li, J. Yang, D. J. Srolovitz, Q. Zheng, *Nature Communications* **2015**, 6, 7853.
[120] M. Xia, C. Liang, Z. Cheng, R. Hu, S. Liu, *Physical Chemistry Chemical Physics* **2019**, 21, 1217.
[121] J. Wang, D. C. Sorescu, S. Jeon, A. Belianinov, S. V. Kalinin, A. P. Baddorf, P. Maksymovych, *Nature Communications* **2016**, 7, 13263.
[122] C. D. van Engers, N. E. A. Cousens, V. Babenko, J. Britton, B. Zappone, N. Grobert, S. Perkin, *Nano Letters* **2017**, 17, 3815.
[123] P. Li, Z. You, T. Cui, *Sensors and Actuators A: Physical* **2014**, 217, 56.
[124] M. R. Roenbeck, X. Wei, A. M. Beese, M. Naraghi, A. o. Furmanchuk, J. T. Paci, G. C. Schatz, H. D. Espinosa, *ACS Nano* **2014**, 8, 124.
[125] R. Menzel, A. Lee, A. Bismarck, M. S. P. Shaffer, *Langmuir* **2009**, 25, 8340.
[126] T. Arai, Y. Tominaga, S. Asai, M. Sumita, *Journal of Polymer Science Part B: Polymer Physics* **2005**, 43, 2568.
[127] X. Zhang, D. Yang, P. Xu, C. Wang, Q. Du, *Journal of Materials Science* **2007**, 42, 7069.
[128] J.-F. Dai, G.-J. Wang, C.-K. Wu, *Chromatographia* **2014**, 77, 299.
[129] J. Weippert, J. Hauns, J. Bachmann, A. Böttcher, X. Yao, B. Yang, A. Narita, K. Müllen, M. M. Kappes, *The Journal of chemical physics* **2018**, 149, 194701.
[130] H. K. Christenson, *The Journal of Physical Chemistry* **1993**, 97, 12034.
[131] A. I. Bailey, S. M. Kay, *Proceedings of the Royal Society of London. Series A. Mathematical and Physical Sciences* **1967**, 301, 47.
[132] J. Schultz, K. Tsutsumi, J.-B. Donnet, *Journal of Colloid and Interface Science* **1977**, 59, 277.
[133] H. K. Christenson, *Journal of Colloid and Interface Science* **1988**, 121, 170.
[134] K.-T. Wan, B. Lawn, *Acta Metallurgica et Materialia* **1990**, 38, 2073.
[135] K. T. Wan, D. T. Smith, B. R. Lawn, *Journal of the American Ceramic Society* **1992**, 75, 667.
[136] H. Keller-Rudek, G. K. Moortgat, R. Sander, R. Sörensen, *Earth Syst. Sci. Data* **2013**, 5, 365.
[137] W. Prichard, W. Orville-Thomas, *Transactions of the Faraday Society* **1963**, 59, 2218.
[138] B. Jańczuk, W. Wójcik, A. Zdziennicka, *Journal of Colloid and Interface Science* **1993**, 157, 384.
[139] C. J. Drummond, D. Y. Chan, *Langmuir* **1997**, 13, 3890.
[140] M. Taniguchi, J. S. Lindsey, *Photochemistry and photobiology* **2018**, 94, 290.
[141] C. Wohlfarth, Springer-Verlag Berlin Heidelberg.
[142] R. Kumar, S. Asad Ali, A. H. Naqvi, H. S. Virk, U. De, D. K. Avasthi, R. Prasad, *Indian Journal of Physics* **2009**, 83, 969.
[143] J. Flores-Mijangos, V. Beltrán-López, *Appl. Opt.* **2003**, 42, 592.

[144] K. A. M. dos Santos, P. A. Z. Suarez, J. C. Rubim, *Polymer Degradation and Stability* **2005**, 90, 34.
[145] A. A. Serkov, H. V. Snelling, S. Heusing, T. M. Amaral, *Scientific Reports* **2019**, 9, 1773.
[146] X. Zhang, J. Qiu, J. Zhao, X. Li, L. Liu, *Journal of Quantitative Spectroscopy and Radiative Transfer* **2020**, 252, 107063.
[147] Y. Ozaki, I. Tanabe, *Analyst* **2016**, 141, 3962.
[148] M. Moazzami Gudarzi, S. H. Aboutalebi, 2021.
[149] E. Yousif, M. Abdallh, H. Hashim, N. Salih, J. Salimon, B. M. Abdullah, Y.-F. Win, *International Journal of Industrial Chemistry* **2013**, 4, 4.
[150] C. D. Keefe, L. A. Donovan, *Journal of Molecular Structure* **2001**, 597, 259.
[151] C. D. Keefe, E. Butler, E. Gillis, J. L. MacDonald, *Spectrochimica Acta Part A: Molecular and Biomolecular Spectroscopy* **2007**, 67, 125.
[152] F. M. Fowkes, *Industrial & Engineering Chemistry* **1964**, 56, 40.
[153] M. R. Querry, MISSOURI UNIV-KANSAS CITY, 1985.
[154] S. Handschuh-Wang, Y. Chen, L. Zhu, X. Zhou, *ChemPhysChem* **2018**, 19, 1584.
[155] T. A. Hamdalla, T. A. Hanafy, A. E. Bekheet, *Journal of Spectroscopy* **2015**, 2015, 204867.
[156] I. Bodurov, I. Vlaeva, A. Viraneva, T. Yovcheva, S. Sainov, *power electronics*, 2, 1.
[157] H. Schonhorn, G. Baker, F. Bates, *Journal of Polymer Science: Polymer Physics Edition* **1985**, 23, 1555.
[158] H. Fujimoto, K. Kamiya, J. Tanaka, M. Tanaka, *Synthetic Metals* **1985**, 10, 367.
[159] C. R. Fincher, M. Ozaki, M. Tanaka, D. Peebles, L. Lauchlan, A. J. Heeger, A. G. MacDiarmid, *Physical Review B* **1979**, 20, 1589.
[160] T. Nowlin, D. F. Smith Jr, *Journal of Applied Polymer Science* **1980**, 25, 1619.
[161] Q. He, J.-Q. Liu, B. Yang, X. Chen, C.-S. Yang, *Surface and Coatings Technology* **2014**, 252, 120.
[162] Y. Li, L. Su, C. Shou, C. Yu, J. Deng, Y. Fang, *Scientific Reports* **2013**, 3, 2865.
[163] I. Savva, A. S. Kalogirou, A. Chatzinicolaou, P. Papaphilippou, A. Pantelidou, E. Vasile, E. Vasile, P. A. Koutentis, T. Krasia-Christoforou, *RSC Advances* **2014**, 4, 44911.
[164] T. A. F. König, P. A. Ledin, J. Kerszulis, M. A. Mahmoud, M. A. El-Sayed, J. R. Reynolds, V. V. Tsukruk, *ACS Nano* **2014**, 8, 6182.
[165] S. Habaue, R. Ito, K. Okumura, Y. Takamushi, *Journal of Polymers* **2015**, 2015, 478729.
[166] E. Fekete, J. Móczó, B. Pukánszky, *Journal of colloid and interface science* **2004**, 269, 143.
[167] J. Kuczynski, E. Papirer, *European polymer journal* **1991**, 27, 653.
[168] K. Davis, R. Yarbrough, M. Froeschle, J. White, H. Rathnayake, *RSC Advances* **2019**, 9, 14638.
[169] W. L. Bond, *Journal of Applied Physics* **1965**, 36, 1674.
[170] R. J. Collins, D. A. Kleinman, *Journal of Physics and Chemistry of Solids* **1959**, 11, 190.
[171] D. M. Roessler, W. C. Walker, *Physical Review* **1967**, 159, 733.
[172] R. HANNA, *Journal of the American Ceramic Society* **1965**, 48, 376.
[173] C. M. Miller, Texas A & M University.
[174] M. R. Querry, *Optical constants of minerals and other materials from the millimeter to the ultraviolet*, Chemical Research, Development & Engineering Center, US Army Armament …, **1987**.
[175]
[176] D. D. S. Meneses, B. Rousseau, P. Echegut, P. Simon, *Applied Spectroscopy* **2007**, 61, 1390.
[177] E. Papirer, E. Brendlé, H. Balard, J. Dentzer, *Journal of materials science* **2000**, 35, 3573.
[178] W. Wu, G. H. Nancollas, *Advances in colloid and interface science* **1999**, 79, 229.
[179] H. H. Li, *Journal of Physical and Chemical Reference Data* **1980**, 9, 161.
[180] J. Barth, R. Johnson, M. Cardona, D. Fuchs, A. M. Bradshaw, *Physical Review B* **1990**, 41, 3291.
[181] W. Kaiser, W. G. Spitzer, R. H. Kaiser, L. E. Howarth, *Physical Review* **1962**, 127, 1950.
[182] R. L. Amey, R. H. Cole, *The Journal of Chemical Physics* **1964**, 40, 146.
[183] M. S. Anderson, C. A. Swenson, *Journal of Physics and Chemistry of Solids* **1975**, 36, 145.
[184] W. F. Chan, G. Cooper, X. Guo, G. R. Burton, C. E. Brion, *Physical Review A* **1992**, 46, 149.
[185] E. Papirer, J.-M. Perrin, B. Siffert, G. Philipponneau, *Journal of colloid and interface science* **1991**, 144, 263.

[186] J. Shen, Y. He, J. Wu, C. Gao, K. Keyshar, X. Zhang, Y. Yang, M. Ye, R. Vajtai, J. Lou, *Nano letters* **2015**, 15, 5449.
[187] P. K. Sharma, K. Hanumantha Rao, *Advances in Colloid and Interface Science* **2002**, 98, 341.
[188] G. Carron, *Acta Crystallogr.* **1963**, 16, 338.
[189] J.-B. Vaney, J. Carreaud, G. Delaizir, A. Piarristeguy, A. Pradel, E. Alleno, J. Monnier, E. Lopes, A. Gonçalves, A. Dauscher, *Journal of Materials Chemistry C* **2016**, 4, 2329.
[190] J. Trotter, *Zeitschrift für Kristallographie-Crystalline Materials* **1965**, 121, 81.
[191] Springer-Verlag Berlin Heidelberg.
[192] Springer-Verlag Berlin Heidelberg.
[193] H. Boller, *Monatshefte für Chemie / Chemical Monthly* **1973**, 104, 916.
[194] C. Chen, M. Wang, J. Wu, H. Fu, H. Yang, Z. Tian, T. Tu, H. Peng, Y. Sun, X. Xu, J. Jiang, N. B. M. Schröter, Y. Li, D. Pei, S. Liu, S. A. Ekahana, H. Yuan, J. Xue, G. Li, J. Jia, Z. Liu, B. Yan, H. Peng, Y. Chen, *Science Advances* **2018**, 4, eaat8355.
[195] K. Łukaszewicz, J. Stępień-Damm, A. Pietraszko, A. Kajokas, J. Grigas, *Pol. J. Chem.* **1999**, 73, 541.
[196] A. Cantarero, J. Martinez-Pastor, A. Segura, A. Chevy, *Applied Physics A* **1988**, 45, 125.
[197] A. Cantarero, J. Martinez-Pastor, A. Segura, A. Chevy, *Physical Review B* **1987**, 35, 9586.
[198] B. Gardes, G. Brun, J.-C. Tedenac, *Eur. J. Solid State Inorg. Chem.* **1989**, 26, 221.
[199] M. Hervieu, C. Michel, B. Domenges, Y. Laligant, A. Lebail, G. Ferey, B. Raveau, *Mod. Phys. Lett. B* **1988**, 2, 491.
[200] J. Hwang, T. Timusk, G. Gu, *Journal of Physics: Condensed Matter* **2007**, 19, 125208.
[201] S. Nakajima, *J. Phys. Chem. Solids* **1963**, 24, 479.
[202] C. Lee, T.-H. An, E. E. Gordon, H. S. Ji, C. Park, J.-H. Shim, Y. S. Lim, M.-H. Whangbo, *Chem. Mater.* **2017**, 29, 2348.
[203] L.-D. Zhao, J. He, D. Berardan, Y. Lin, J.-F. Li, C.-W. Nan, N. Dragoe, *Energy & Environmental Science* **2014**, 7, 2900.
[204] H. Hiramatsu, H. Yanagi, T. Kamiya, K. Ueda, M. Hirano, H. Hosono, *Chemistry of Materials* **2008**, 20, 326.
[205] D. Berardan, J. Li, E. Amzallag, S. Mitra, J. Sui, W. Cai, N. Dragoe, *Materials* **2015**, 8.
[206] H. Brœkken, *Zeitschrift für Kristallographie-Crystalline Materials* **1930**, 74, 67.
[207] U. Schwarz, A. Wosylus, M. Schmidt, L. Akselrud, A. Ormeci, M. Hanfland, V. Hermann, C. Kuntscher, *Inorganics* **2019**, 7, 143.
[208] M.-H. Du, D. J. Singh, *Physical Review B* **2010**, 82, 045203.
[209] Springer-Verlag Berlin Heidelberg.
[210] F. Bannister, *Mineralogical magazine and journal of the Mineralogical Society* **1935**, 24, 49.
[211] Springer-Verlag Berlin Heidelberg.
[212] Z. Deng, D. Chen, B. Peng, F. Tang, *Crystal Growth & Design* **2008**, 8, 2995.
[213] T. Unuma, T. Sasaki, K. Yamaki, A. Irie, H. Ishida, T. Kato, *OSA Continuum* **2020**, 3, 2646.
[214] Z.-Y. Zhao, Q.-L. Liu, W.-W. Dai, *Scientific Reports* **2016**, 6, 31449.
[215] mindat.
[216] Springer-Verlag Berlin Heidelberg.
[217] E. Dönges, *Z. anorg. allg. Chem* **1951**, 265, 6.
[218] C. Martin, A. V. Suslov, S. Buvaev, A. F. Hebard, P. Bugnon, H. Berger, A. Magrez, D. B. Tanner, *EPL (Europhysics Letters)* **2016**, 116, 57003.
[219] A. Shevelkov, E. Dikarev, R. Shpanchenko, B. Popovkin, *Journal of Solid State Chemistry* **1995**, 114, 379.
[220] C. Martin, A. Suslov, S. Buvaev, A. Hebard, P. Bugnon, H. Berger, A. Magrez, D. Tanner, *Physical Review B* **2014**, 90, 201204.
[221] A. Akrap, J. Teyssier, A. Magrez, P. Bugnon, H. Berger, A. B. Kuzmenko, D. Van Der Marel, *Physical Review B* **2014**, 90, 035201.
[222] J.-J. Ying, V. V. Struzhkin, Z.-Y. Cao, A. F. Goncharov, H.-K. Mao, F. Chen, X.-H. Chen, A. G. Gavriliuk, X.-J. Chen, *Physical Review B* **2016**, 93, 100504.
[223] A. Tomokiyo, T. Okada, S. Kawano, *Japanese Journal of Applied Physics* **1977**, 16, 291.
[224] K. Ishizaka, M. S. Bahramy, H. Murakawa, M. Sakano, T. Shimojima, T. Sonobe, K. Koizumi, S. Shin, H. Miyahara, A. Kimura, K. Miyamoto, T. Okuda, H. Namatame, M. Taniguchi, R.

Arita, N. Nagaosa, K. Kobayashi, Y. Murakami, R. Kumai, Y. Kaneko, Y. Onose, Y. Tokura, *Nature Materials* **2011**, 10, 521.
[225] M. Tran, J. Levallois, P. Lerch, J. Teyssier, A. Kuzmenko, G. Autes, O. Yazyev, A. Ubaldini, E. Giannini, D. Van Der Marel, *Physical review letters* **2014**, 112, 047402.
[226] P. Lošťák, J. Horák, A. Vaško, N. t. Dich, *physica status solidi (a)* **1980**, 59, 311.
[227] L. Wu, J. Yang, M. Chi, S. Wang, P. Wei, W. Zhang, L. Chen, J. Yang, *Scientific Reports* **2015**, 5, 14319; L. Wu, J. Yang, S. Wang, P. Wei, J. Yang, W. Zhang, L. Chen, *Physical Review B* **2014**, 90, 195210.
[228] O. Hassel, *Norsk Geologisk Tidsskrift* **1927**, 9, 266.
[229] P. Trucano, R. Chen, *Nature* **1975**, 258, 136.
[230] C. C. Stoumpos, D. H. Cao, D. J. Clark, J. Young, J. M. Rondinelli, J. I. Jang, J. T. Hupp, M. G. Kanatzidis, *Chemistry of Materials* **2016**, 28, 2852.
[231] K. Leng, L. Wang, Y. Shao, I. Abdelwahab, G. Grinblat, I. Verzhbitskiy, R. Li, Y. Cai, X. Chi, W. Fu, P. Song, A. Rusydi, G. Eda, S. A. Maier, K. P. Loh, *Nature Communications* **2020**, 11, 5483.
[232] W. R. Busing, H. A. Levy, *The Journal of Chemical Physics* **1957**, 26, 563.
[233]
[234] J. W. Anthony, R. A. Bideaux, K. W. Bladh, M. C. Nichols, *Handbook of Mineralogy*, Mineralogical Society of America, Chantilly, VA 20151-1110, USA **2010**.
[235] M. R.S., *Zeitschrift für Kristallographie* **1962**, 117, 309.
[236] Y. De Haan, *Bur. Stand.(US), Spec. Publ* **1969**, 301, 233.
[237] B. Evans, in *Optical and Electrical Properties*, Springer, 1976.
[238] F. Boucher, M. Evain, R. Brec, *Acta Crystallographica Section B: Structural Science* **1995**, 51, 952.
[239] C. Calareso, V. Grasso, L. Silipigni, *Journal of Applied Physics* **1997**, 82, 6228.
[240] C. Malliakas, S. J. Billinge, H. J. Kim, M. G. Kanatzidis, *Journal of the American Chemical Society* **2005**, 127, 6510.
[241] A. Sacchetti, L. Degiorgi, T. Giamarchi, N. Ru, I. Fisher, *Physical Review B* **2006**, 74, 125115.
[242] S. Kalasina, N. Phattharasupakun, T. Maihom, V. Promarak, T. Sudyoadsuk, J. Limtrakul, M. Sawangphruk, *Scientific Reports* **2018**, 8, 12192.
[243] H. Grime, J. A. Santos, *Zeitschrift für Kristallographie-Crystalline Materials* **1934**, 88, 136.
[244] Y. Sakisaka, T. Ishii, T. Sagawa, *Journal of the Physical Society of Japan* **1974**, 36, 1365.
[245] I. Pollini, J. Thomas, A. Lenselink, *Physical Review B* **1984**, 30, 2140.
[246] V. Carteaux, D. Brunet, G. Ouvrard, G. Andre, *Journal of Physics: Condensed Matter* **1995**, 7, 69.
[247] H. Ji, R. A. Stokes, L. D. Alegria, E. C. Blomberg, M. A. Tanatar, A. Reijnders, L. M. Schoop, T. Liang, R. Prozorov, K. S. Burch, N. P. Ong, J. R. Petta, R. J. Cava, *Journal of Applied Physics* **2013**, 114, 114907.
[248] H. Braekken, *Nor. Vidensk. Selsk. Forh* **1932**, 5, 42.
[249] G. Guizzetti, L. Nosenzo, I. Pollini, E. Reguzzoni, G. Samoggia, G. Spinolo, *Physical Review B* **1976**, 14, 4622.
[250] N. Wooster, *Zeitschrift für Kristallographie-Crystalline Materials* **1930**, 74, 363.
[251] M. Bykov, E. Bykova, L. Dubrovinsky, M. Hanfland, H.-P. Liermann, S. van Smaalen, *Scientific reports* **2015**, 5, 1.
[252] T. Zhang, Y. Wang, H. Li, F. Zhong, J. Shi, M. Wu, Z. Sun, W. Shen, B. Wei, W. Hu, X. Liu, L. Huang, C. Hu, Z. Wang, C. Jiang, S. Yang, Q.-m. Zhang, Z. Qu, *ACS Nano* **2019**, 13, 11353.
[253] M. Wang, J. Zhang, Z. Wang, C. Wang, S. van Smaalen, H. Xiao, X. Chen, C. Du, X. Xu, X. Tao, *Advanced Optical Materials* **2020**, 8, 1901446.
[254] R. Diehl, C.-D. Carpentier, *Acta Crystallographica Section B: Structural Crystallography and Crystal Chemistry* **1977**, 33, 1399.
[255] R. A. Susilo, B. G. Jang, J. Feng, Q. Du, Z. Yan, H. Dong, M. Yuan, C. Petrovic, J. H. Shim, D. Y. Kim, B. Chen, *npj Quantum Materials* **2020**, 5, 58.
[256] J. Beck, *Zeitschrift für anorganische und allgemeine Chemie* **1990**, 585, 157.

[257] E. J. Telford, A. H. Dismukes, K. Lee, M. Cheng, A. Wieteska, A. K. Bartholomew, Y.-S. Chen, X. Xu, A. N. Pasupathy, X. Zhu, C. R. Dean, X. Roy, *Advanced Materials* **2020**, 32, 2003240.
[258] A. V. Arakcheeva, G. Chapuis, M. Meyer, *Zeitschrift für Kristallographie - Crystalline Materials* **2001**, 216, 199.
[259] J. Gu, G. Yan, Y. Lian, Q. Mu, H. Jin, Z. Zhang, Z. Deng, Y. Peng, *RSC Advances* **2018**, 8, 25802.
[260] J. Haberecht, H. Borrmann, R. Kniep, *Zeitschrift für Kristallographie-New Crystal Structures* **2001**, 216, 544.
[261] I. Pollini, G. Benedek, J. Thomas, *Physical Review B* **1984**, 29, 3617.
[262] R. Wyckoff, *Crystal structures* **1963**, 1, 239.
[263] J. Thomas, I. Pollini, *Physical Review B* **1985**, 32, 2522.
[264] M. Goldsztaub, *CR Hebd. Seances Acad. Sci* **1934**, 198, 667.
[265] Y. Zeng, P. Gu, Z. Zhao, B. Zhang, Z. Lin, Y. Peng, W. Li, W. Zhao, Y. Leng, P. Tan, T. Yang, Z. Zhang, Y. Song, J. Yang, Y. Ye, K. Tian, Y. Hou, *Advanced Materials* **2022**, 34, 2108847.
[266] W. Klingen, G. Eulenberger, H. Hahn, *Zeitschrift für anorganische und allgemeine Chemie* **1973**, 401, 97.
[267] P. J. S. Foot, J. Suradi, P. A. Lee, *Materials Research Bulletin* **1980**, 15, 189.
[268] R. Brec, D. Schleich, G. Ouvrard, A. Louisy, J. Rouxel, *Inorganic Chemistry* **1979**, 18, 1814.
[269] D. Fenske, H. G. von Schnering, *Angewandte Chemie International Edition in English* **1983**, 22, 407.
[270] V. Kucek, C. Drasar, J. Navratil, L. Benes, P. Lostak, *Journal of Crystal Growth* **2013**, 380, 72.
[271] C. Drasar, V. Kucek, L. Benes, P. Lostak, M. Vlcek, *Journal of Solid State Chemistry* **2012**, 193, 42.
[272] R. Wyckoff, *Crystal structures* **1963**, 1, 85.
[273] T. McMath, J. Irwin, *physica status solidi (a)* **1976**, 38, 731.
[274] J. F. Molloy, M. Naftaly, Y. M. Andreev, G. V. Lanskii, I. N. Lapin, A. I. Potekaev, K. A. Kokh, A. V. Shabalina, A. V. Shaiduko, V. A. Svetlichnyi, *CrystEngComm* **2014**, 16, 1995.
[275] L. I. Tatarinova, Y. K. Auleitner, Z. G. Pinsker, *Kristallografiya* **1956**, 1, 537.
[276] M. Julien-Pouzol, S. Jaulmes, M. Guittard, F. Alapini, *Acta Crystallographica Section B: Structural Crystallography and Crystal Chemistry* **1979**, 35, 2848.
[277] J. F. Sánchez-Royo, A. Segura, V. Muñoz, *physica status solidi (a)* **1995**, 151, 257.
[278] D. G. Mead, L. Genzel, *Infrared Physics* **1978**, 18, 555.
[279] A. Wilms, R. Kniep, *Zeitschrift für Naturforschung B* **1981**, 36, 1658.
[280] R. Kniep, A. Wilms, H. J. Beister, *Materials Research Bulletin* **1983**, 18, 615.
[281] T. Wadsten, *Acta Chem. Scand.* **1967**, 21, 593.
[282] C. S. Jung, D. Kim, S. Cha, Y. Myung, F. Shojaei, H. G. Abbas, J. A. Lee, E. H. Cha, J. Park, H. S. Kang, *Journal of Materials Chemistry A* **2018**, 6, 9089.
[283] J. W. Rau, C. Kannewurf, *Physical Review B* **1971**, 3, 2581.
[284] W. Zachariasen, *Physical Review* **1932**, 40, 917.
[285] H. C. Hsueh, J. X. Li, C. H. Ho, *Advanced Optical Materials* **2018**, 6, 1701194.
[286] A. M. Elkorashy, *Physica B: Condensed Matter* **1989**, 159, 171.
[287] A. Okazaki, *Journal of the Physical Society of Japan* **1958**, 13, 1151.
[288] C. R. Kannewurf, R. J. Cashman, *Journal of Physics and Chemistry of Solids* **1961**, 22, 293.
[289] A. Elkorashy, *physica status solidi (b)* **1988**, 149, 747.
[290] J. M. Chamberlain, S. S. Sirbegovic, P. M. Nikolic, *Journal of Physics C: Solid State Physics* **1974**, 7, L150.
[291] L. E. Conroy, K. C. Park, *Inorganic Chemistry* **1968**, 7, 459.
[292] T. Iwasaki, N. Kuroda, Y. Nishina, *Journal of the Physical Society of Japan* **1982**, 51, 2233.
[293] D. T. Hodul, M. J. Sienko, *Inorganic Chemistry* **1981**, 20, 3655.
[294] H. Vaterlaus, F. Levy, *Journal of Physics C: Solid State Physics* **1985**, 18, 2351.
[295] A. Laturia, M. L. Van de Put, W. G. Vandenberghe, *npj 2D Materials and Applications* **2018**, 2, 1.
[296] G. Lucovsky, R. M. White, J. A. Benda, J. F. Revelli, *Physical Review B* **1973**, 7, 3859.
[297] A. Hussain Reshak, S. Auluck, *Physica B: Condensed Matter* **2005**, 363, 25.

[298] S. Mangelsen, P. Naumov, O. Barkalov, S. Medvedev, W. Schnelle, M. Bobnar, S. Mankovsky, S. Polesya, C. Näther, H. Ebert, *Physical Review B* **2017**, 96, 205148.
[299] J. M. Bijvoet, A. Claassen, A. Karssen, *Proceedings of the Koninklijke Nederlandse Academie van Wetenschappen* **1926**, 29, 529.
[300] J. Biellmann, B. Prevot, *Infrared Physics* **1980**, 20, 99.
[301] K. Osamura, Y. Murakami, Y. Tomiie, *Journal of the Physical Society of Japan* **1966**, 21, 1848.
[302] F. Lyu, Y. Sun, Q. Yang, B. Tang, M. Li, Z. Li, M. Sun, P. Gao, L.-H. Ye, Q. Chen, *Nanotechnology* **2020**, 31, 315711.
[303] C. Julien, M. Eddrief, M. Balkanski, A. Chevy, *Physical Review B* **1992**, 46, 2435.
[304] K. Schubert, E. Dörre, E. Günzel, *Naturwissenschaften* **1954**, 41, 448.
[305] A. F. Qasrawi, N. M. Gasanly, *Solid State Communications* **2010**, 150, 325.
[306] P. Gomes da Costa, R. G. Dandrea, R. F. Wallis, M. Balkanski, *Physical Review B* **1993**, 48, 14135.
[307] J. Camassel, P. Merle, H. Mathieu, A. Chevy, *Physical Review B* **1978**, 17, 4718.
[308] N. M. Gasanly, B. M. Yavadov, V. I. Tagirov, E. A. Vinogradov, *physica status solidi (b)* **1978**, 89, K43.
[309] R. Kniep, A. Wilms, *Materials Research Bulletin* **1980**, 15, 763.
[310] R. Kniep, A. Wilms, J. Beister, K. Syassen, *Zeitschrift für Naturforschung B* **1981**, 36, 1520.
[311] G. Sawitzki, D. Müller, H. Hahn, *Materials Research Bulletin* **1980**, 15, 753.
[312] S. Guggenheim, Y.-H. Chang, A. F. Koster van Groos, *American Mineralogist* **1987**, 72, 537.
[313] J. W. Gruner, *Zeitschrift für Kristallographie-Crystalline Materials* **1934**, 88, 412.
[314] R. Frisenda, Y. Niu, P. Gant, M. Muñoz, A. Castellanos-Gomez, *npj 2D Materials and Applications* **2020**, 4, 38.
[315] D. Barthelmy, in *Mineralogy Database*, Vol. 2021.
[316] L. Desgranges, G. Calvarin, G. Chevrier, *Acta Crystallographica Section B: Structural Science* **1996**, 52, 82.
[317] A. Suslu, K. Wu, H. Sahin, B. Chen, S. Yang, H. Cai, T. Aoki, S. Horzum, J. Kang, F. M. Peeters, S. Tongay, *Scientific Reports* **2016**, 6, 20525.
[318] Vol. 2021, Hudson Institute of Mineralogy.
[319] W. Busing, *Trans. Am. Cryst. Assoc* **1970**, 6, 57.
[320] S. Kinno, R. Onaka, *Journal of the Physical Society of Japan* **1980**, 49, 1379.
[321] S. T. Pantelides, *Physical Review Letters* **1975**, 35, 250.
[322] W. Klingen, R. Ott, H. Hahn, *Zeitschrift für anorganische und allgemeine Chemie* **1973**, 396, 271.
[323] R. F. Frindt, D. Yang, P. Westreich, *Journal of Materials Research* **2005**, 20, 1107.
[324] A. Wiedenmann, J. Rossat-Mignod, A. Louisy, R. Brec, J. Rouxel, *Solid State Communications* **1981**, 40, 1067.
[325] V. Grasso, L. Silipigni, *J. Opt. Soc. Am. B* **1999**, 16, 132.
[326] N. Wooster, *Zeitschrift für Kristallographie-Crystalline Materials* **1931**, 80, 504.
[327] R. G. Dickinson, L. Pauling, *Journal of the American Chemical Society* **1923**, 45, 1466.
[328] L. C. Towle, V. Oberbeck, B. E. Brown, R. E. Stajdohar, *Science* **1966**, 154, 895.
[329] D. Puotinen, R. Newnham, *Acta Crystallographica* **1961**, 14, 691.
[330] G. Hägg, N. Schönberg, *Ark. Kemi* **1954**, 7, 371.
[331] F. Kadijk, F. Jellinek, *Journal of the Less Common Metals* **1971**, 23, 437.
[332] R. Cairns, E. Ott, *Journal of the American Chemical Society* **1933**, 55, 527.
[333] Y. Qi, H. Qi, J. Li, C. Lu, *Journal of Crystal Growth* **2008**, 310, 4221.
[334] T. Marcopoulos, M. Economou, *American Mineralogist* **1981**, 66, 1020.
[335] J. A. A. Ketelaar, *Zeitschrift für Kristallographie - Crystalline Materials* **1934**, 88, 26.
[336] C. R. Ronda, G. J. Arends, C. Haas, *Physical Review B* **1987**, 35, 4038.
[337] C. Haas, *Physica B+C* **1981**, 105, 305.
[338] B. E. Taylor, J. Steger, A. Wold, *Journal of Solid State Chemistry* **1973**, 7, 461.
[339] S. Tengner, *Zeitschrift für anorganische und allgemeine Chemie* **1938**, 239, 126.
[340] W. Nieuwenkamp, J. Bijvoet, *Zeitschrift für Kristallographie-Crystalline Materials* **1932**, 82, 157.

[341] A. Eijkelenkamp, K. Vos, *physica status solidi (b)* **1976**, 76, 769.
[342] M. Sieskind, J. C. Boulou, A. Fettouhi, D. Ayachour, *Materials Research Bulletin* **2000**, 35, 1897.
[343] W. Nieuwenkamp, J. Bijvoet, *Zeitschrift für Kristallographie-Crystalline Materials* **1932**, 81, 469.
[344] P. Terpstra, H. G. K. Westenbrink, *Proceedings of the Koninklijke Nederlandse Academie van Wetenschappen* **1926**, 29, 431.
[345] F. Gronvold, H. Haraldsen, A. Kjekshus, *ACTA CHEMICA SCANDINAVICA* **1960**, 14, 1879.
[346] C. Mankai, G. Martinez, O. Gorochov, *Physical Review B* **1977**, 16, 4666.
[347] S. Furuseth, K. Selte, A. Kjekshus, *ACTA CHEMICA SCANDINAVICA* **1965**, 19, 257.
[348] J. He, W. Jiang, X. Zhu, R. Zhang, J. Wang, M. Zhu, S. Wang, Y. Zheng, L. Chen, *Physical Chemistry Chemical Physics* **2020**, 22, 26383.
[349] F. Ghasemi, R. Taghavimendi, A. Bakhshayeshi, *Optical and Quantum Electronics* **2020**, 52, 1.
[350] G. Kliche, *Journal of Solid State Chemistry* **1985**, 56, 26.
[351] B. Ghosh, F. Alessandro, M. Zappia, R. Brescia, C.-N. Kuo, C. S. Lue, G. Chiarello, A. Politano, L. S. Caputi, A. Agarwal, *Physical Review B* **2019**, 99, 045414.
[352] Amit, R. K. Singh, N. Wadehra, S. Chakraverty, Y. Singh, *Physical Review Materials* **2018**, 2, 114202; O. Pavlosiuk, D. Kaczorowski, *Scientific Reports* **2018**, 8, 11297.
[353] K. Friemelt, L. Kulikova, L. Kulyuk, A. Siminel, E. Arushanov, C. Kloc, E. Bucher, *Journal of Applied Physics* **1996**, 79, 9268.
[354] N. A. Pike, A. Dewandre, B. Van Troeye, X. Gonze, M. J. Verstraete, *Physical Review Materials* **2018**, 2, 063608.
[355] N. W. Alcock, A. Kjekshus, *ACTA CHEMICA SCANDINAVICA* **1965**, 19, 79.
[356] C. H. Ho, P. C. Liao, Y. S. Huang, T. R. Yang, K. K. Tiong, *Journal of Applied Physics* **1997**, 81, 6380.
[357] H. Jiao, X. Qin, Y. Li, G. Wang, *Infrared Physics & Technology* **2021**, 116, 103752.
[358] H. Hillebrecht, T. Ludwig, G. Thiele, *Zeitschrift für anorganische und allgemeine Chemie* **2004**, 630, 2199.
[359] S. Sinn, C. H. Kim, B. H. Kim, K. D. Lee, C. J. Won, J. S. Oh, M. Han, Y. J. Chang, N. Hur, H. Sato, B.-G. Park, C. Kim, H.-D. Kim, T. W. Noh, *Scientific Reports* **2016**, 6, 39544.
[360] M. J. Buerger, S. B. Hendricks, *Zeitschrift für Kristallographie - Crystalline Materials* **1938**, 98, 1.
[361] M. Vuković, Z. Branković, D. Poleti, A. Rečnik, G. Branković, *Journal of sol-gel science and technology* **2014**, 72, 527.
[362] E. Dönges, *Zeitschrift für anorganische und allgemeine Chemie* **1950**, 263, 280.
[363] S. A. Semiletov, *Kristallografiya* **1956**, 1, 403.
[364] R. Sehr, L. R. Testardi, *Journal of Physics and Chemistry of Solids* **1962**, 23, 1219.
[365] H. T. Langhammer, M. Stordeur, H. Sobotta, V. Riede, *physica status solidi (b)* **1982**, 109, 673.
[366] S. V. Dordevic, M. S. Wolf, N. Stojilovic, H. Lei, C. Petrovic, *Journal of Physics: Condensed Matter* **2013**, 25, 075501.
[367] J. Trotter, T. Zobel, *Zeitschrift für Kristallographie - Crystalline Materials* **1966**, 123, 67.
[368] I. Lefebvre, M. Lannoo, G. Allan, A. Ibanez, J. Fourcade, J. C. Jumas, E. Beaurepaire, *Physical Review Letters* **1987**, 59, 2471.
[369] Springer-Verlag Berlin Heidelberg.
[370] W. Hofmann, *Zeitschrift für Kristallographie - Crystalline Materials* **1935**, 92, 161.
[371] P. M. Nikolić, S. S. Vujatović, O. H. Hughes, C. J. Doran, J. M. Chamberlain, presented at *Proceedings of the Twelfth International Conference on the Physics of Semiconductors*, Wiesbaden, 1974//, **1974**.
[372] R. E. Banai, L. A. Burton, S. G. Choi, F. Hofherr, T. Sorgenfrei, A. Walsh, B. To, A. Cröll, J. R. S. Brownson, *Journal of Applied Physics* **2014**, 116, 013511.
[373] I. Oftedal, *Norsk Geologisk Tidsskrift* **1926**, 9, 225.

[374] L. A. Burton, T. J. Whittles, D. Hesp, W. M. Linhart, J. M. Skelton, B. Hou, R. F. Webster, G. O'Dowd, C. Reece, D. Cherns, D. J. Fermin, T. D. Veal, V. R. Dhanak, A. Walsh, *Journal of Materials Chemistry A* **2016**, 4, 1312.
[375] G. Busch, C. Fröhlich, F. Hulliger, E. Steigmeier, *Helv. Phys. Acta* **1961**, 34, 359.
[376] E. Nicklaus, *physica status solidi (a)* **1979**, 53, 217.
[377] M. Sauvage, *Acta Crystallographica Section B: Structural Crystallography and Crystal Chemistry* **1974**, 30, 2786.
[378] Z.-L. Lv, H.-L. Cui, X.-H. Li, H. Wang, G.-F. Ji, *The European Physical Journal B* **2017**, 90, 1.
[379] F. J. Di Salvo, G. Hull Jr, L. Schwartz, J. Voorhoeve, J. Waszczak, *The Journal of Chemical Physics* **1973**, 59, 1922.
[380] E. Bjerkelund, A. Kjkshus, *Acta Chem Scand* **1967**, 21, 513.
[381] H.-J. Lamfers, A. Meetsma, G. Wiegers, J. De Boer, *J. Alloys Compd.* **1996**, 241, 34.
[382] J. C. Wildervanck, F. Jellinek, *Journal of the Less Common Metals* **1971**, 24, 73.
[383] A. J. Bradley, *The London, Edinburgh, and Dublin Philosophical Magazine and Journal of Science* **1924**, 48, 477.
[384] H. G. V. Schnering, M. Collin, M. Hassheider, *Zeitschrift für anorganische und allgemeine Chemie* **1972**, 387, 137.
[385] C. A. Kuntscher, M. Klemm, S. Horn, M. Sing, R. Claessen, *The European Physical Journal Special Topics* **2009**, 180, 29.
[386] R. Rückamp, Universität zu Köln, 2006.
[387] E. Snigireva, S. Troyanov, V. Rybakov, *ZHURNAL NEORGANICHESKOI KHIMII* **1990**, 35, 1945.
[388] I. Oftedal, *Zeitschrift für Physikalische Chemie* **1928**, 134U, 301.
[389] M. N. Gjerding, R. Petersen, T. G. Pedersen, N. A. Mortensen, K. S. Thygesen, *Nature communications* **2017**, 8, 1.
[390] R. Sudharsanan, K. K. Bardhan, B. P. Clayman, J. C. Irwin, *Solid State Communications* **1987**, 62, 563.
[391] C. Julien, J. Ruvalds, A. Virosztek, O. Gorochov, *Solid State Communications* **1991**, 79, 875.
[392] H. P. Vaterlaus, S. Ansermet, M. Py, F. Lévy, *Solid State Communications* **1980**, 35, 925.
[393] R. H. Friend, D. Jerome, A. D. Yoffe, *Journal of Physics C: Solid State Physics* **1982**, 15, 2183.
[394] Y. Arnaud, M. Chevreton, *Journal of Solid State Chemistry* **1981**, 39, 230.
[395] D. Müller, G. Eulenberger, H. Hahn, *Zeitschrift fuer anorganische und allgemeine Chemie* **1973**, 398, 207.
[396] A. E. Bakhyshov, M. F. Agaeva, A. M. Darvish, *physica status solidi (b)* **1979**, 91, K31.
[397] N. M. Gasanly, A. F. Goncharov, B. M. Dzhavadov, N. N. Melnik, V. I. Tagirov, E. A. Vinogradov, *physica status solidi (b)* **1979**, 92, K139.
[398] A. Ketelaar, *Zeitschrift für Kristallographie-Crystalline Materials* **1936**, 95, 9.
[399] S. Xie, E. Iglesia, A. T. Bell, *The Journal of Physical Chemistry B* **2001**, 105, 5144.
[400] N. Kenny, C. R. Kannewurf, D. H. Whitmore, *Journal of Physics and Chemistry of Solids* **1966**, 27, 1237.
[401] J. Taboada-Gutiérrez, G. Álvarez-Pérez, J. Duan, W. Ma, K. Crowley, I. Prieto, A. Bylinkin, M. Autore, H. Volkova, K. Kimura, T. Kimura, M. H. Berger, S. Li, Q. Bao, X. P. A. Gao, I. Errea, A. Y. Nikitin, R. Hillenbrand, J. Martín-Sánchez, P. Alonso-González, *Nature Materials* **2020**, 19, 964.
[402] P. Clauws, J. Vennik, *physica status solidi (b)* **1976**, 76, 707.
[403] E. Hoschek, W. Klemm, *Zeitschrift für anorganische und allgemeine Chemie* **1939**, 242, 49.
[404] R. W. G. Wyckoff, *Structure of Crystals*, The Chemical Catalog Company, New York **1931**.
[405] W. Schutte, J. De Boer, F. Jellinek, *Journal of Solid State Chemistry* **1987**, 70, 207.
[406] S. Y. Istomin, J. Köhler, A. Simon, *Physica C: Superconductivity* **1999**, 319, 219.
[407] T. Takano, Y. Kasahara, T. Oguchi, I. Hase, Y. Taguchi, Y. Iwasa, *Journal of the Physical Society of Japan* **2011**, 80, 023702.
[408] P. A. Lee, G. Said, R. Davis, T. H. Lim, *Journal of Physics and Chemistry of Solids* **1969**, 30, 2719.
[409] M. Moustafa, T. Zandt, C. Janowitz, R. Manzke, *Physical Review B* **2009**, 80, 035206.

[410] A. B. Kuzmenko, *Review of Scientific Instruments* **2005**, 76, 083108.
[411] M. Ratajack, C. Kannewurf, J. Revelli, J. Wagner, *Physical Review B* **1978**, 17, 4674; S. M. Oliver, J. J. Fox, A. Hashemi, A. Singh, R. L. Cavalero, S. Yee, D. W. Snyder, R. Jaramillo, H.-P. Komsa, P. M. Vora, *Journal of Materials Chemistry C* **2020**, 8, 5732.
[412] M. Bell, W. Liang, *Advances in physics* **1976**, 25, 53.
